# *In silico *miRNA prediction in metazoan genomes: balancing between sensitivity and specificity

**DOI:** 10.1186/1471-2164-10-204

**Published:** 2009-04-30

**Authors:** Ate van der Burgt, Mark WJE Fiers, Jan-Peter Nap, Roeland CHJ van Ham

**Affiliations:** 1 Applied Bioinformatics, Plant Research International, Wageningen University & Research Centre, PO Box 16, 6700 AA Wageningen, The Netherlands; 2 Laboratory of Bioinformatics, Wageningen University, Dreijenlaan 3, 6703 HA Wageningen, The Netherlands; 3 Centre for BioSystems Genomics 2012 (CBSG2012), PO Box 98, 6700 AB Wageningen, The Netherlands; 4 Current address: New Zealand Institute for Plant & Food Research Ltd, Private Bag 4704, Christchurch, New Zealand

## Abstract

**Background:**

MicroRNAs (miRNAs), short ~21-nucleotide RNA molecules, play an important role in post-transcriptional regulation of gene expression. The number of known miRNA hairpins registered in the miRBase database is rapidly increasing, but recent reports suggest that many miRNAs with restricted temporal or tissue-specific expression remain undiscovered. Various strategies for *in silico *miRNA identification have been proposed to facilitate miRNA discovery. Notably support vector machine (SVM) methods have recently gained popularity. However, a drawback of these methods is that they do not provide insight into the biological properties of miRNA sequences.

**Results:**

We here propose a new strategy for miRNA hairpin prediction in which the likelihood that a genomic hairpin is a true miRNA hairpin is evaluated based on statistical distributions of observed biological variation of properties (descriptors) of known miRNA hairpins. These distributions are transformed into a single and continuous outcome classifier called the *L *score. Using a dataset of known miRNA hairpins from the miRBase database and an exhaustive set of genomic hairpins identified in the genome of *Caenorhabditis elegans*, a subset of 18 most informative descriptors was selected after detailed analysis of correlation among and discriminative power of individual descriptors. We show that the majority of previously identified miRNA hairpins have high *L *scores, that the method outperforms miRNA prediction by threshold filtering and that it is more transparent than SVM classifiers.

**Conclusion:**

The *L *score is applicable as a prediction classifier with high sensitivity for novel miRNA hairpins. The *L-*score approach can be used to rank and select interesting miRNA hairpin candidates for downstream experimental analysis when coupled to a genome-wide set of *in silico*-identified hairpins or to facilitate the analysis of large sets of putative miRNA hairpin loci obtained in deep-sequencing efforts of small RNAs. Moreover, the in-depth analyses of miRNA hairpins descriptors preceding and determining the *L *score outcome could be used as an extension to miRBase entries to help increase the reliability and biological relevance of the miRNA registry.

## Background

MicroRNAs (miRNAs) are ~21-nucleotide (nt) short, single stranded RNA molecules involved in post-transcriptional regulation of gene expression [1]. They are present in higher eukaryotes and some viral genomes [2]. Because the miRNA and small-interfering RNA (siRNA) pathways partly overlap, current understanding of miRNA biogenesis has gained from advances made in the field of RNA interference. Mature, functional miRNAs develop from degenerate palindromic repeats with a characteristic hairpin-like secondary structure [1,3,4]. Initially, experimental identification of miRNAs was achieved through direct cloning and sequencing of small RNAs [5,6]. However, such relatively low-throughput screenings were biased towards abundantly or ubiquitously expressed miRNAs [6] and missed many miRNAs with restricted temporal or tissue-specific expression patterns [1]. Recently, strategies using PCR [7], microarrays [8,9] or ultra high-throughput sequencing [10,11] have expanded the list of known miRNAs. Many of these show tissue-specific expression [9,12] or appear to be species-specific [12,13]. In both *Arabidopsis thaliana *[14,15] and *Caenorhabditis elegans *[10,16], for example, high-throughput sequencing of small RNAs shows moderate overlap in detected miRNAs between experiments in each species, indicating that also in well studied genomes many new miRNAs remain to be discovered. In addition, the observation that many miRNA loci exhibit compelling hairpin structures on both sense and antisense strands led to the discovery of anti-sense miRNA transcription [17]. Antisense miRNA transcription and processing yield distinct mature miRNAs. This contributes to the functional diversification of miRNA genes for a considerable fraction of the known miRNA loci [17,18].

Various methods for the *in silico *prediction of miRNAs have been developed to aid in experimental studies of miRNA discovery [19,20]. These methods generally consider the hairpin-like secondary structure of the miRNA precursor, the miRNA hairpin, as the most important characteristic of a miRNA gene. They use RNA secondary structure prediction (RSSP) algorithms, such as RNAfold [21] or Mfold [22], to predict the secondary structure and thermodynamic stability of the RNA hairpin structures. Current bioinformatics approaches for the prediction of miRNAs [19,20] generally include three steps: (1) genome-wide prediction of hairpin structures; (2) filtering or scoring of those hairpins on the basis of their similarity in physical and sequence features to known miRNA hairpins and (3) experimental validation of putative candidates.

A common approach for the first step is to search for hairpin structures using a sliding window and perform RSSP on each window [5,8,23,24]. An improvement in overall calculation time over this approach is to first identify degenerate palindromic sequences and to analyse only these further with RSSP [25,26]. Unfortunately, these approaches detect vast numbers of hairpin structures in complete eukaryotic genomes. Depending on the method used, 1E3 to 4E3 hairpins per Mb of genomic sequence are found, resulting in about 1E7 hairpins identified in the human genome [8,24-27]. The challenge is, therefore, to devise an appropriate filtering method to separate the chaff from the wheat.

Different criteria for filtering candidate miRNA sequences have been proposed, generally with the aim to reduce the search space and/or to increase the specificity of prediction [20]. Evolutionary conservation is considered an important feature of the hairpin sequence [1] and analysis thereof is often used to identify and focus comparisons on the conserved non-coding sequence space in different genomes [5,23]. An evolutionary approach known as phylogenetic shadowing has been used for combined selection and filtering of miRNA candidates [28]. This study revealed a characteristic camel-shaped conservation pattern of putatively orthologous miRNAs that was useful as a criterion for finding conserved miRNA candidates in primate genomes. Other filtering criteria include intragenomic matching of candidate miRNAs and their potential targets [29], evidence for expression, thresholds on structural properties of hairpins, e.g. minimal folding energy (MFE), absence of repetitive or low-complexity sequences, occurrence in introns or intergenic regions [11], or proximity to known miRNA loci [2,24,30].

Stringent filtering is performed to attain high specificity, that is, to minimize the number of false positive predictions of miRNA genes [20,25]. Obviously, maximizing specificity increases the number of false negative predictions, that is, a decreased sensitivity [31]. This implies that with stringent filtering, relevant miRNAs will be missed. The success of any filtering procedure depends however on the validity of the underlying assumptions. For example, filtering on evolutionary conservation will miss species-specific or fast evolving miRNAs [12] and low-complexity filtering is likely to miss miRNAs originating from transposable elements [32,33]. Several recent methods employ machine learning techniques such as a Support Vector Machine (SVM) [24,27,34,35] for classification. Such SVMs evaluate differences in hairpin properties between true-positive and true-negative examples of miRNAs for a given taxon to generate a prediction classifier. SVMs have been successfully used for miRNA gene prediction [24,34,35] and for the prediction of 5' Drosha processing sites in miRNA hairpins [26,11]. Although the SVM approach is claimed to outperform earlier methods [35], SVM-based classifications combine many features in a single kernel function and therefore do not provide direct insight into the biological significance of these features. Such insight can only be obtained through expert analyses and dedicated feature selection procedures [19]. Moreover, the set of true-negatives which is required for training an SVM is often very difficult to define.

Here, we present an innovative strategy for miRNA prediction that focuses on attaining optimal sensitivity. We define and combine 40 different filtering criteria and, using a set of genomic hairpins identified in the genome of *Caenorhabditis elegans*, show that 18 of these characteristics capture the biological variation of miRNA features present in sets of known miRNA hairpins. These 18 criteria are used to establish a combined likelihood score *L *that assesses the likelihood that a predicted hairpin structure in a genome contains a genuine miRNA. *L *is a continuous classifier that allows user-adjustable thresholds for sensitivity and specificity in *ab initio *miRNA prediction and miRNA analysis. Good performance of *L *for large sets of hairpins from the genomes of *C. elegans *and four viruses demonstrates the added value of the new analytical strategy for future miRNA discovery and selection.

## Results

We have developed and evaluated a new computational strategy for the prediction of candidate miRNAs in DNA sequences. The new approach focuses on high sensitivity in an initial hairpin detection step, followed by a flexible, user-adjustable procedure to balance sensitivity with specificity and selectivity. For all hairpin sequences from an input sequence, a miRNA likelihood score *L *is calculated, given an underlying scoring model based on descriptors of the physical and sequence characteristics of miRNAs (Table 1; [see Additional file 1]). The performance of the strategy was assessed by retrieval of known miRNAs from hairpin structures identified in the genome of *C. elegans*. Appropriate scoring models were derived for various taxonomic sets of known miRNAs.

**Table 1:** Subset of 18 most informative miRNA hairpin descriptors

**Descriptor**	**Explanation**	**Bound**. ^**a**^	**Type **^**b**^	**Discriminative power **^**c**^	**K **^**d**^
bulgeRatio	ratio asymmetrical bulges vs. stem length	↑	str	1.45	0.416
dP	adjusted base pairing propensity (dP)	↓	str	2.28	0.417
largest bulge	longest bulge in stem (nt)	↑	str	1.74	0.343
longest match-stretch	longest match-stretch in stem (nt)	↓	str	1.20	0.336
Looplength	central loop length (nt)	↑	str	1.17 (u)	0.191
max match count	matches in 24 nt	↓	str	2.75	0.477
MFEahl index [39]	MFEahl corrected for GC- Content	↓	str	4.75	0.706
Q [37,39]	Normalized Shannon entropy (Q)	↑	str	3.01	0.844
stem length	stem length	↑↓	str	1.29 (l)	0.404
GAsurplusCU	surplus of GA over CU in sequence	↑↓	seq	1.12 (u) 1.05 (l)	0.195
GsurplusC	surplus of G over C in sequence	↓	seq	1.12	0.995
polyA	longest poly-A stretch (nt)	↑	seq	1.58	0.834
polyNucHairpin	longest mono-nucleotide stretch (nt) in the hairpin	↑	seq	1.64	0.846
polyU	longest poly-U stretch (nt)	↑	seq	1.53	0.540
SCS-di	Di-nucleotide Sequence Complexity (-)	↑	seq	1.77	0.557
SCS-mono	Mono-nucleotide Sequence Complexity (-)	↓	seq	1.60	0.317
GU-match contribution	ratio of GU-matches vs. all matches	↑	mix	1.28	0.173
MFEahl (dG) [37,39]	MFE Adjusted for hairpin length	↓	mix	13.33	0.742

A detailed explanation of all 40 descriptors is given in the additional information [see Additional files 1 and 2].

^a ^Boundary; indication of extreme tail of descriptor distribution that was transformed into *S *< 1 fraction. Symbols denote: ↓ lower tail; ↑ upper tail, and; ↓↑ both tails.

^b ^Type; descriptor based on structural (str), sequence (seq) or both structural and sequence (mix) properties of the hairpin.

^c ^Discriminative power; expressed at 95% sensitivity, measured on the taxonomic set Metazoa (3,902 miRNA hairpins, positives) and genomic hairpins in *C. elegans *(3,526,115 hairpins, negatives). * Discriminative power of descriptor 'Z' was measured on 25,599 identified hairpins in four viruses.

^d ^K; highest Cohen's kappa coefficient [41] with another descriptor, measured on the taxonomic set Metazoa with *S *< 1 cut-off of 95%.

### Descriptor data fit

**Figure 1:**
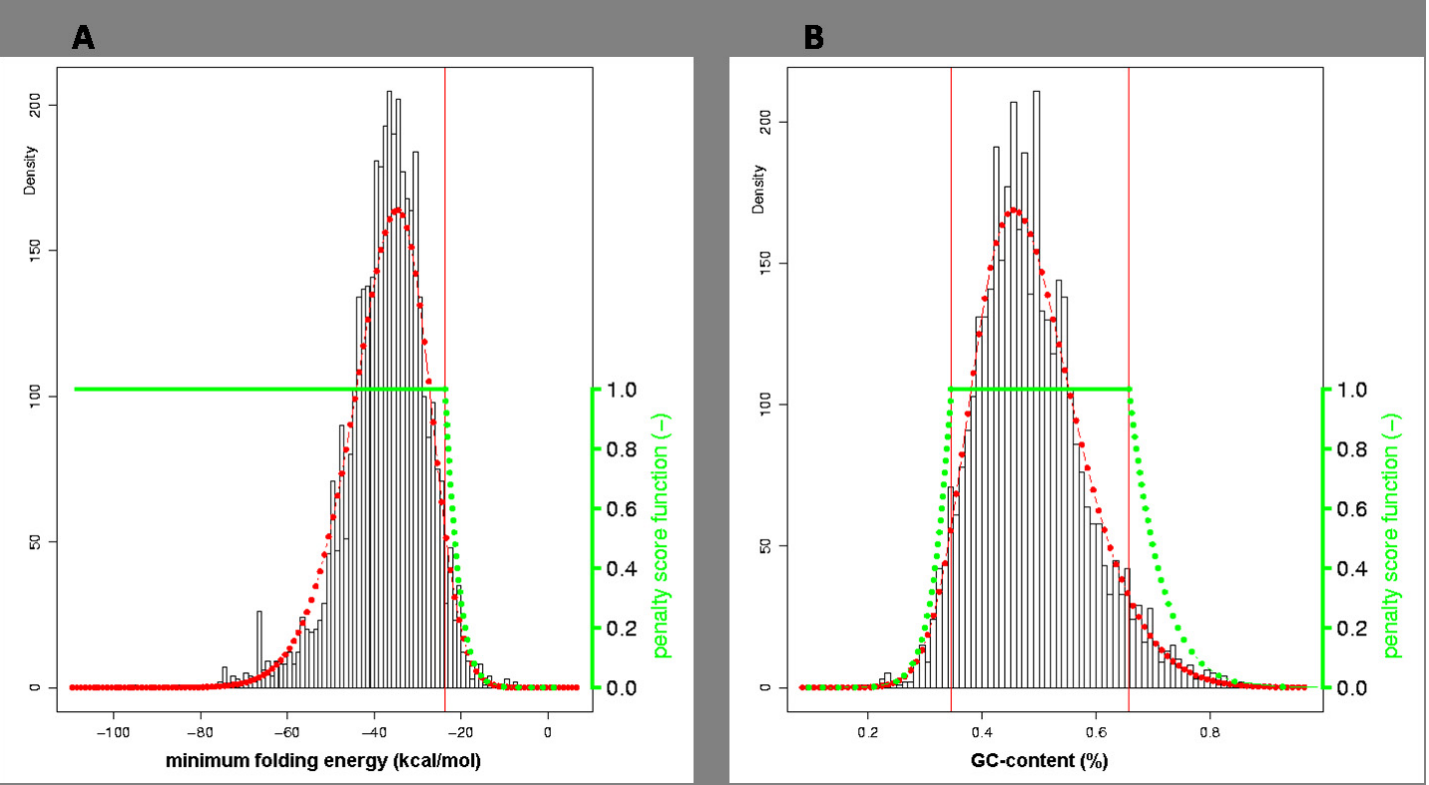
**Data fit and likelihood distribution function for two descriptors**. Frequency distribution (black bars), SN-fitted distribution (red curve) and likelihood distribution function (LDF) (green curve) for descriptors MFE (A) and GC-content (B) of the taxonomic set Metazoa (3,902 miRNA hairpins). Red vertical lines mark the upper and lower 5% tails of the distribution.

Most of the descriptors used for miRNA characteristics have been proposed in previous studies [36-39], but a few are, to the best of our knowledge, for the first time defined in this study, for example 'GAsurplusCU' (Table 1). In all cases except one, the empirical data showed a good fit to a skew-normal (SN) probability distribution [40] according to a Chi-square goodness-of-fit test (*p *≤ 1.4E-5; [see Additional file 2]). As example, the frequency distribution, fitted SN distribution and the transformed likelihood distribution function (LDF) are shown for the descriptors MFE and GC content in Figure 1. The only exception to an SN probability distribution fit was found for the descriptor 'P', which is the *p*-value of the MFE of randomized sequence [38]. This fitted best to an exponential distribution corrected for zero values. Results of the data fit for all descriptors from the taxonomic set Metazoa are listed in Additional file 1.

### Likelihood score *S *for miRNA hairpin descriptors

The CDF of the fitted distribution was transformed into an LDF with outcome *S*. The range of *S *(between 0 and 1) has an *S *< 1 and an *S *= 1 fraction which are separated by the cut-off value derived from the 95% confidence interval of the descriptor's CDF. The *S *< 1 fraction contains miRNAs hairpins with descriptor values in the tail(s) of the distribution. These have a low probability of occurring in true miRNA hairpins. The *S *= 1 fraction contains miRNA hairpins with values in the remainder of the distribution and corresponds to likely properties of miRNA hairpins. Descriptors were treated differently with respect to the transformation of the tails of the CDF (Table 1; [see Additional file 2]). For example, for the descriptor "minimal folding energy" (MFE) of a miRNA, there is in principle no need to impose a lower bound, even though the fitted distribution (Figure 1A) indicates that very low MFE values occur rarely in known miRNAs. The LDF of the descriptor MFE assigns a score *S *= 1 for all values below -23.72 kcal/mol. Higher MFEs are penalized proportional to the LDF and therefore assigned a score S < 1. MFE is an example of a descriptor where the *S *< 1 fraction represents the upper 5% tail of the confidence interval. For other descriptors, the *S *< 1 fraction is represented by the lower 5% tail of the distribution (e.g. match ratio) or by both the lower 5% and the upper 5% tail (e.g. GC-content, Figure 1B). Table 1 and Additional file 1 list the unlikely tails for each descriptor.

### Correlation of descriptors

The 40 descriptors here defined were either based on the sequence of the hairpin, the structure of the hairpin or on a combination of both (Table 1; [see Additional file 1]). Correlations among these were obvious, for instance, between stem length or GC-content on the one hand and the MFE of a hairpin on the other hand. Correlated descriptors will overemphasize the importance of a more general feature of a miRNA hairpin and affect the usefulness of *L*. To assess the correlation among descriptors in their *S *< 1 fractions, we calculated Cohen's kappa coefficient *κ *[41] for all 780 possible pairs of descriptors, using the miRNA hairpins of the taxonomic set Metazoa [see Additional file 3]. For each descriptor the most strongly correlated descriptor, as determined by the highest observed *κ *is listed in Table 1 (column *κ*) and Additional file 3. The highest *κ *was found between descriptors 'GCratio' and 'GsurplusC' (*κ *= 0.995), followed by 'D' and 'Q' (*κ *= 0.844) and the pair 'P' and 'Z' (*κ *= 0.784).

### Discriminative power of descriptors

**Figure 2:**
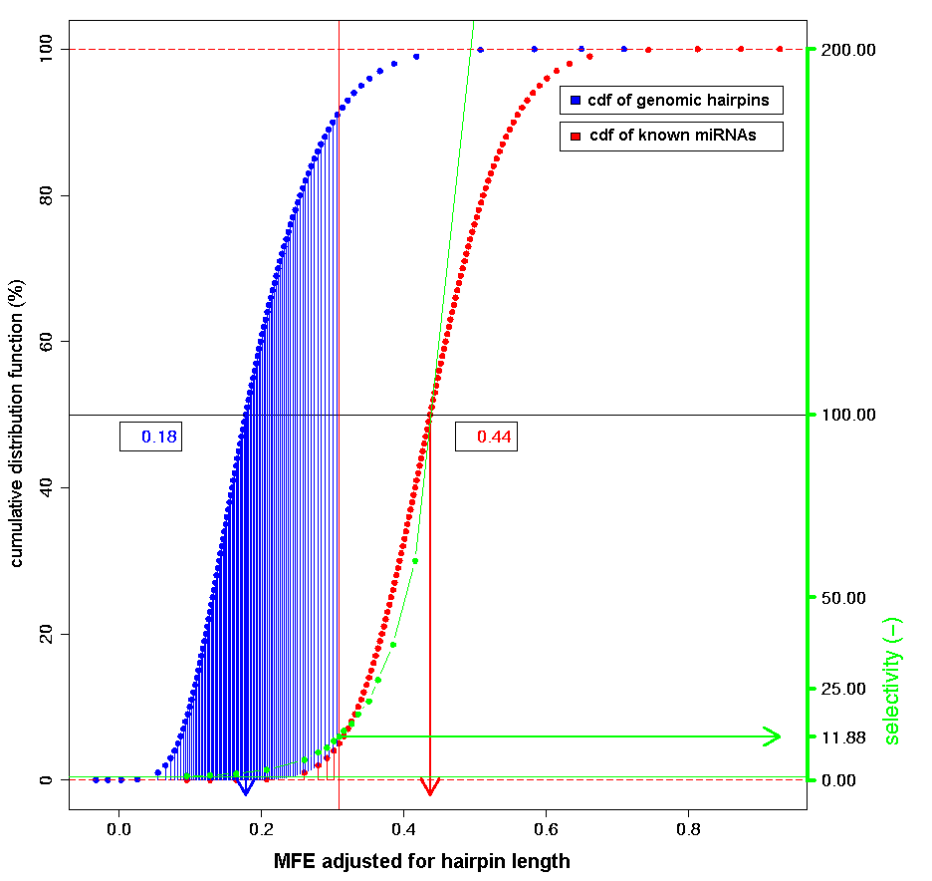
**Discriminative power of the descriptor MFEahl**. Red curve represents the CDF of the descriptor MFEahl for the taxonomic set Metazoa (3,902 miRNA hairpins). Blue curve represents the CDF of the SN-fitted distribution of the same descriptor in case of 100,000 randomly selected hairpins from the *C. elegans *genome. Green curve represents the discriminative power, calculated as sensitivity/(1.0-specificity). The fraction of hairpins in the *S *< 1 fraction is shaded (*S *< 1 cut-off at 95% of the CDF of known miRNA hairpins). The discriminative power at 95% sensitivity is shown by a green arrow (13.33). SN-fitted means are shown by red (0.44) and blue (0.18) arrows.

A discriminative descriptor contributes to the separation of true miRNA hairpins from non-miRNA hairpins. The discriminative power of a descriptor was defined as the ratio of percentages of miRNA hairpins and genomic hairpins that comply with a threshold set to the descriptor's limiting value between *S *= 1 and *S *< 1 of the LDF (95% of the CDF). A discriminative power smaller than 1.0 implies that relatively more miRNA hairpins are rejected than genomic hairpins. Higher values are obtained for descriptor values that are typically encountered in miRNA hairpins, but that are less common in collections of genomic, predominantly non-miRNA hairpins. The most discriminative descriptor was MFEahl (13.33) and least discriminative were polyC, polyCstem, polyGstem (0.95) (Table 1; [see Additional file 2]). Figure 2 illustrates the discriminative power of descriptor MFEahl and reveals a substantial difference between the SN-fitted CDFs of miRNA hairpins and randomly selected genomic hairpins. Only 7% of genomic hairpins complied with the criterion of an MFEahl of 0.314, representing 95% of the CDF of metazoan miRNA hairpins. The opposite was true for the least discriminative descriptors: for example for polyC, 99% of the genomic hairpins versus 96% of the metazoan miRNA hairpins had a longest polyC stretch smaller than five (not shown).

### Delimiting a subset of most informative descriptors

The correlation and discriminative power of descriptors were used to select a non-redundant subset of most informative descriptors. Descriptors that either correlated with a more selective descriptor, using a threshold for correlation of *κ*>0.4, or that had a discriminative power smaller than 1.1 were omitted. This resulted in a subset of 18 descriptors, seven of which were sequence related, nine structure related, and two descriptors with mixed properties (Table 1). Remarkably, the descriptors 'GC-content' and the MFE randomization descriptors 'P' [38] and 'Z' [37], which are often used in miRNA prediction studies, were not included in the subset [see Additional file 1]. The latter two ranked among the most selective descriptors, but were excluded because of their strong correlation with the most selective descriptor MFEahl index, in which the MFE is adjusted for hairpin length and GC-content [see Additional file 3].

### Assessment of scoring model performance

The 18 most selective descriptors (Table 1) were used to define and analyze different scoring models. The CDF of the fitted distribution of all 18 descriptors was transformed into an LDF with outcome *S *and the *L *score for a given miRNA sequence was calculated as the product of all *S *values. Scoring model performance, defined as the power to distinguish (potential) miRNA hairpins from other (or random) genomic hairpins, was compared for different models that were built using varying settings for five parameters (see below). Scoring model performance was measured as AUC performance and, where appropriate, with selectivity measured at two values of sensitivity (95% and 75%), using genomic hairpins in *C. elegans *and the collection of miRNAs hairpins in the taxonomic set Metazoa.

#### (1) Size of taxonomic set

**Figure 3:**
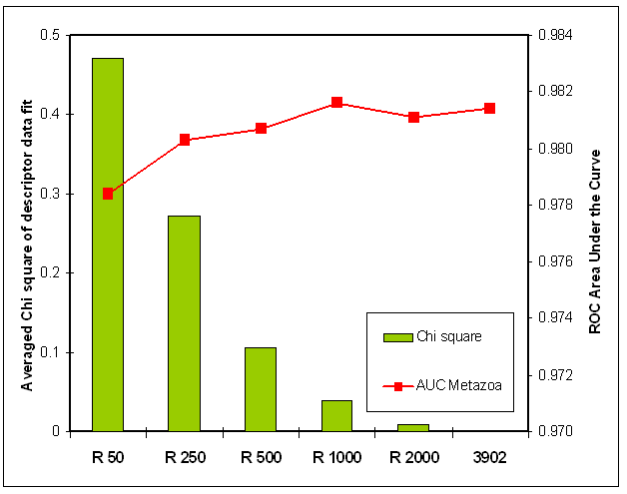
**Accuracy of fit and scoring model performance depends on the size of the input set**. AUC performance (red line) and average Chi-square accuracy of fit of 40 descriptors (green bars), using six scoring models that were based on varying sizes of the input set. Input-set sizes are indicated with a prefix 'R' and comprised 50, 250, 500, 1000, 2000, and the complete set (3,902) of metazoan miRNA hairpins. The smaller sets were compiled by randomly selecting miRNA hairpins from the complete set. This was repeated 50 times for each set. The accuracy of fit was then calculated by averaging Chi-square test statistics over all 40 descriptors and the 50 randomly selected subsets of each indicated size. Both AUC performance and Chi-square statistics show a strong dependency on Input-set size.

As expected, scoring models based on small taxonomic sets had a less accurate data fit, as shown by increasing Chi-square statistics for decreasing set size (Figure 3). Gain in goodness-of-fit and AUC performance saturated with increasing set size. The data presented in Figure 3 indicate that a minimal set size of a thousand miRNA hairpins is required for an accurate data fit (average *p*(Chi-square) < 0.05). Performance in terms of selectivity appeared to increase beyond this set size, suggesting that prediction performance can be further improved by using larger taxonomic sets.

#### (2) Composition of taxonomic set

**Figure 4:**
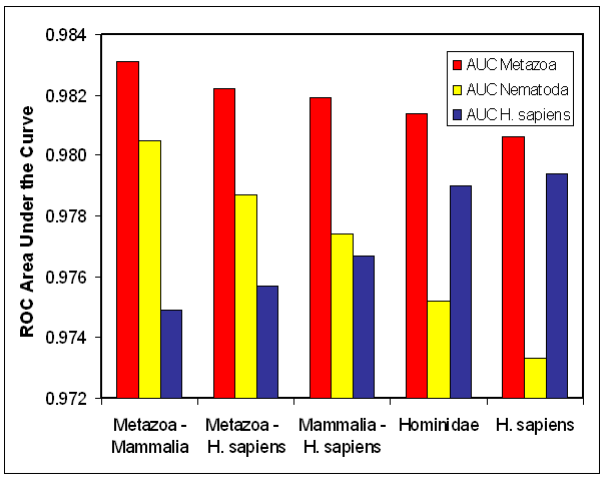
**Scoring model performance depends on the taxonomic distance of the input set**. AUC performance of five different scoring models that vary in the distance of the taxonomic input set. Area under the ROC curves is measured for the taxonomic sets Metazoa (red), Nematoda (yellow) and *H. sapiens *(blue) versus 200,000 randomly selected hairpins from the set of 3,526,115 *C. elegans *hairpins. Scoring models "X – Y" have as taxonomic input set all miRNA hairpins from set X after removal of set Y. From these subsets (and the set Hominidae) 781 miRNA hairpins have been randomly selected. The results presented for the random subsets are averages from 50 independent repeats.

Performance was found to depend on the evolutionary distance between the species contained in a scoring model's taxonomic set and the species for which (miRNA) hairpins are scored by that scoring model. When five taxonomic sets were constructed that comprised equally sized sets of miRNA hairpins from taxa with a decreasing evolutionary distance and diversity relative to human (Figure 4), these showed increasing AUC performance on the miRNA hairpins from human. The opposite trend, *i.e*. a decrease in AUC performance of the same five scoring models, was observed for miRNA hairpins from Nematoda and Metazoa. Seemingly small differences in AUC values translate to substantial differences in genome-wide counts of positive hairpins. When choosing an arbitrary miRNA detection sensitivity of 75%, the difference between an AUC of 0.9831 (red bar Metazoa- Mammalia in Figure 4) and 0.9806 (red bar *Homo sapiens *in Figure 4) results in 6,749 or 17.6% fewer remaining genomic hairpins (38,383 and 31,634 hairpins, respectively). The results confirm that a scoring model based on a set that is taxonomically closest to the organism for which the miRNA hairpins are scored, performs best. However, it is noteworthy that taxonomic sets that do not contain human miRNA hairpins (*e.g*. Mammalia excluding *H. sapiens*, Figure 4) can yield scoring models with a good performance for identifying human miRNAs.

#### (3) Composition of descriptor subset

**Figure 5:**
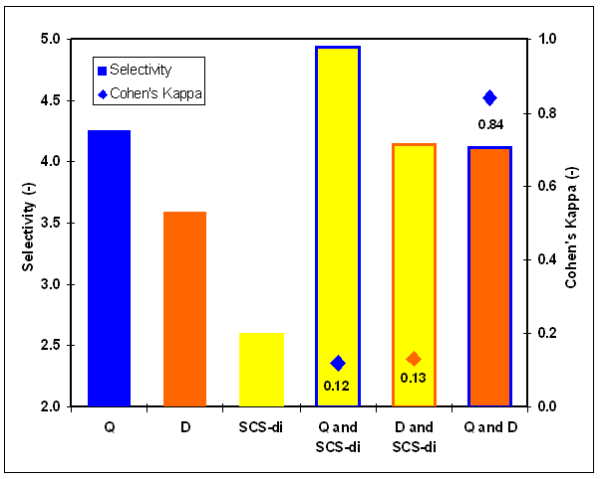
**Scoring model performance depends on the correlation of descriptors**. Discriminative power of three individual and three pairs of descriptors. For the descriptor pairs, Cohen's kappa coefficients are also given. Selectivity is expressed at 95% sensitivity on the set of all metazoan miRNA hairpins; specificity is measured on the set of 3,526,115 hairpins in the genome of *C. elegans*.

Selection of informative descriptors was found to be a crucial step in the development of scoring models, and three factors that affected scoring model performance were therefore analyzed in detail, i.e. the number of descriptors selected (quantity), discriminative power of descriptors (quality) and correlation between descriptors. Correlation of structural properties that describe RNA molecules is well-known [37]. In a miRNA prediction method such correlations can strongly influence the prediction accuracy and should therefore be dealt with cautiously. Figure 5 illustrates the effect of highly correlated descriptors on scoring model performance, using the strongly correlated descriptors 'D' and 'Q' (*κ *= 0.84) and a third descriptor 'SCS-di' that has a weak correlation with both 'D' and 'Q' (*κ *= 0.13 and 0.12, respectively). Scoring models containing pairs of uncorrelated descriptors had a higher selectivity value than the individual descriptors. In contrast, the scoring model based on the strongly correlated descriptors 'D' and 'Q' gave a lower discriminative power than one based on the most discriminative, individual descriptor 'Q' (Figure 5). Although this decrease (-0.14) seems small, the combined effect over all correlated descriptors will have a considerable effect in terms of the absolute number of genomic hairpins that are penalized. It illustrates the necessity of selecting a descriptor subset with as little pair-wise correlation as possible in the development of appropriate scoring models.

#### (4) Parameterization of the LDF

**Figure 6:**
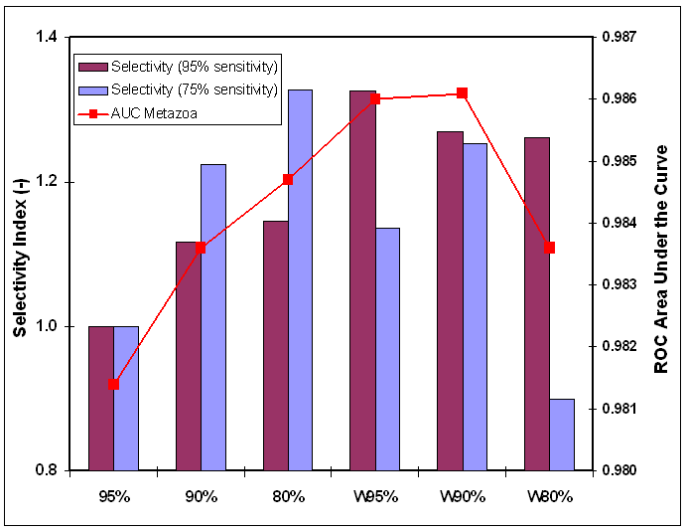
**Scoring model performance depends on LDF parameterization and weighting of descriptors**. AUC performance and selectivity of six different scoring models that vary in parameterization of the LDF (95-90-80%) and have no weighted (weight = 1.0) or weighted individual descriptors (W). Weights were adjusted to the square root of the descriptor's discriminative power as measured at a sensitivity of 95% (Table 1). The square root was taken to prevent disproportionate influence of descriptors with high discriminative power. All models have the same input set (3,902 metazoan miRNA hairpins) and are based on the previously selected set of 18 descriptors. Selectivity is expressed at 95% (purple) and 75% (blue) sensitivity on the set of all metazoan miRNA hairpins; specificity is measured on the set of 3,526,115 hairpins in the genome of *C. elegans*. Relative values of selectivity are presented with the initial scoring model taken as index (selectivity of 12.6 at 95% and 74.1 at 75% sensitivity).

The effect of parameterization on performance was assessed by lowering the default cut-off value (default 95% of the CDF) to 90% and 80% before use in calculation of the transformed likelihood distribution score *S*. The parameterization caused the number of miRNA hairpins included in the *S *< 1 fractions to double (90%) or quadruplicate (80%) and increased the penalization of hairpins relative to the default cut-off value. Figure 6 shows that increased AUC performance and selectivity values were obtained by lowering the cut-off value compared to the reference model, with selectivity indexed for the reference model. The increase is explained by the fact that most descriptors gain in discriminative power at a decrease in sensitivity (see Figure 2).

#### (5) Weighting of descriptors

The effect of weighting individual descriptors was studied by assigning weights to each of the 18 previously selected descriptors. Weights were equal to the square root of a descriptor's discriminative power as measured at a sensitivity of 95% and ranged from 1.06 for GAsurplusCU to 3.65 for MFEahl (Table 1; [see Additional file 1]). Figure 6 shows that a scoring model with weighted descriptors (W95% and W90%) had a significantly increased AUC performance and selectivity index relative to their unweighted models (95% and 90%). Obviously, when weighted on the basis of their discriminative power, descriptors with a high discriminative power contribute stronger to the overall *L *score than descriptors with low discriminative power. As such, a better separation between miRNA hairpins and random genomic hairpins is accomplished.

Finally, combinations of weighting and varying the parameterization of descriptors were tested (models W90% and W80%, Fig. 6). Scoring model W90% had the highest AUC performance on metazoan miRNA hairpins of all tested models. When weighting and parameterization were compared, weighting showed a stronger effect on selectivity measured at 95% sensitivity, whereas parameterization had a stronger effect on selectivity at 75% sensitivity. This implies that both variables have a distinct effect on the shape of the ROC curve.

### Building optimal scoring models

**Figure 7:**
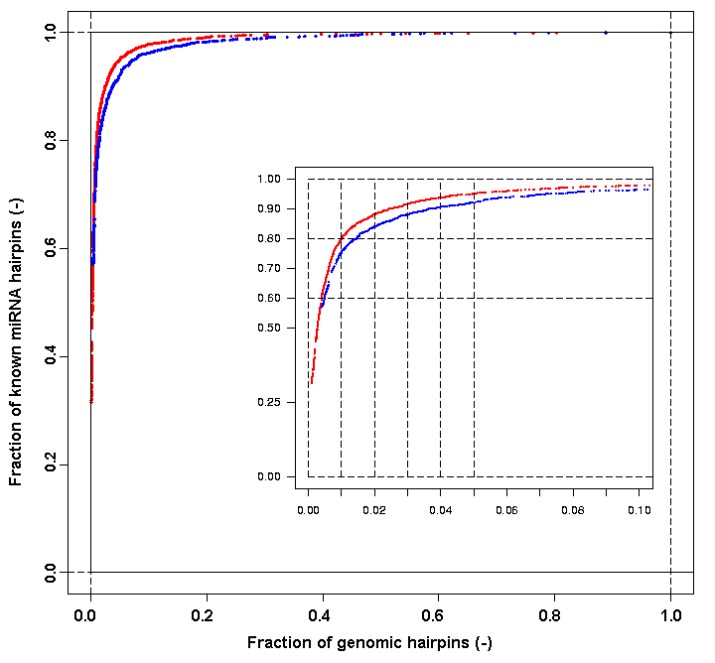
**ROC-curve of the *L-score *classifier of two different scoring models**. ROC curve of the *L-score *classifier of the final scoring model Metazoa (red) and the initial model without weighting and default parameterization (blue). True positives are measured on the taxonomic set Metazoa (3,902 miRNA hairpins), false positives on 500,000 randomly selected genomic hairpins from *C. elegans*.

The data collected and analyses presented allowed the selection of optimal scoring models, with maximal discriminative power to distinguish true miRNA hairpins from other genomic (or random) hairpins for any given case. In general, significant increases in selectivity were gained by descriptor weighting and parameterization. In terms of choice for a specific scoring model, the taxonomic input set should be sufficiently large and taxonomically as close as possible to the organism of interest. In total, 23 scoring models were built, based on 23 distinct taxonomic sets and the subset of 18 most informative descriptors, with the LDF parameterized at 90% of the CDF and using individual weighting of descriptors. Weighting and parameterization at 90% of the CDF resulted in the highest AUC performance. Table 2 shows the performance gain of these optimal scoring models relative to their non-optimized counterparts. Figure 7 shows the ROC curves for the final and initial scoring model Metazoa. The final scoring model Metazoa was subjected to a 10-fold cross-validation for benchmarking and yielded an AUC of 0.9732. The arbitrary cut-off for *L *of 1.0e-4 classifies 87.3% (1,774/2,033) of miRNA hairpins correctly as positive and 97.0% of all genomic hairpins of *C. elegans *as negative. The difference in performance with the non cross-validated AUC performance (0.9874) is due in part to the much smaller taxonomic input set used. The latter was obtained by clustering all Metazoan miRNA hairpins on the basis of sequence similarity. Nearly identical hairpin sequences can have subtle variation in some descriptor values and thereby accurately represent the fact that miRNAs occur in families. Therefore, and to maintain a classifier as selective as possible, we recommend to use the non-clustered variant of the scoring model.

**Table 2:** Scoring model performance

**Scoring model description**	# ^a^	Wt ^b^	LDF ^c^	Chi – Square ^d^	AUC^e^	AUC ^e^	AUC ^e^	Selectivity ^f^	
					Nematoda	*H. sapiens*	Metazoa	95%	75%
**Default models **^**g**^									
Metazoa	3,902	-	0.95	9.95e-7	0.9760	0.9764	0.9814	12.57	74.06
*H. sapiens*	781	-	0.95	0.090	0.9733	0.9794	0.9806	11.04	68.90
*C. elegans*	131	-	0.95	0.405	0.9747	0.9638	0.9735	7.73	41.43
**Optimized models **^**g**^									
Metazoa	3902	Y	0.90	9.95e-7	0.9813	0.9848	0.9874	21.92	105.7
*H*. sapiens	781	Y	0.90	0.090	0.9798	0.9870	0.9871	21.93	87.36
*C. elegans*	131	Y	0.90	0.405	0.9817	0.9775	0.9835	16.65	75.34

^a ^Number of miRNAs in taxonomic set

^b ^-; No weighting (weight = 1), Y; weighting individual descriptors by the square root of their discriminative power (Table 1)

^c ^LDF parameterized at 95% or 90% of the CDF

^d ^Goodness-of-fit is evaluated by averaging Chi-square test statistics of all 40 descriptors

^e ^Area under the curve of ROC curves measured on the taxonomic sets of known miRNA hairpins from Nematodes (211 miRNA hairpins), *H. sapiens *(781) and Metazoa (3,902) versus 200,000 randomly selected genomic hairpins from *C. elegans*.

^f ^Selectivity expressed at 95% and 75% sensitivity, with sensitivity measured on the taxonomic set of Metazoa, specificity measured on set of 3,526,115 *C. elegans *genomic hairpins.

^g ^Scorings models composed of the subset of 18 most informative descriptors

### Combined likelihood score *L *for miRNA hairpin descriptors

**Figure 8:**
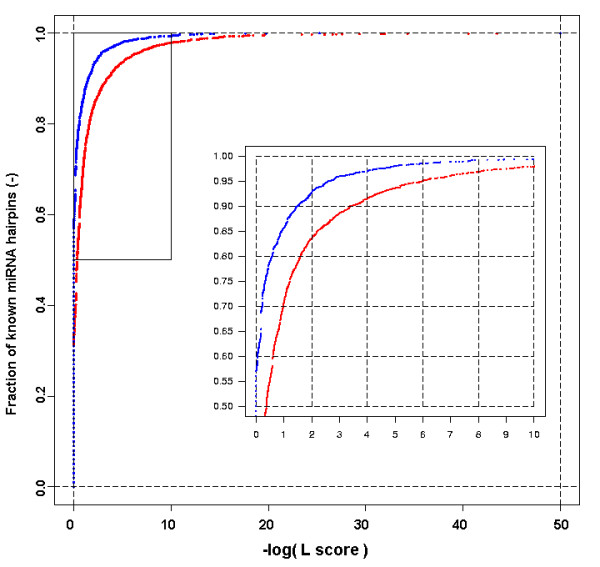
**Cumulative *L-score *plot of two different scoring models**. Ratio of miRNA hairpins in the taxonomic set Metazoa (3,902) that have an *L *score of at least a certain value. Data are shown for the final scoring models Metazoa (red) and the initial model without weighting and default parameterization (blue).

**Figure 9:**
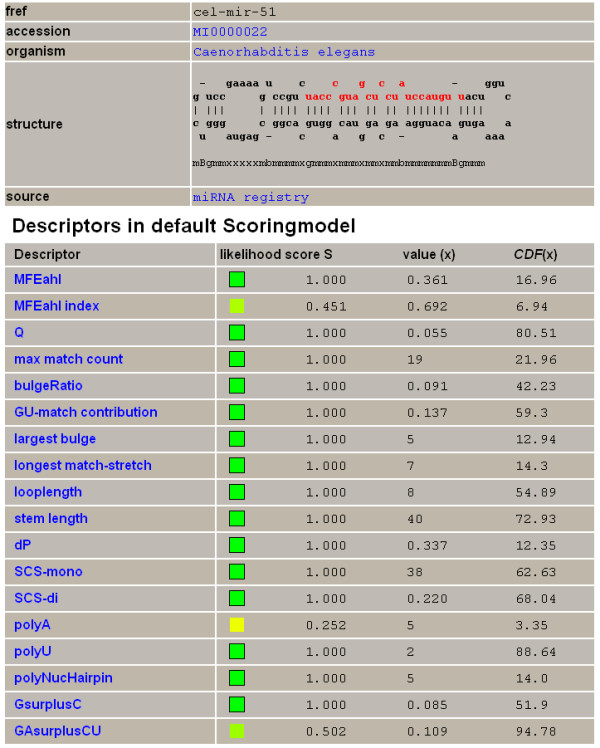
**Detailed descriptor analyses report for cel-mir-38**. Detailed report for the observed descriptor values of cel-mir-38 in the scoring model Metazoa (*L *score = 0.057). For each descriptor, a color-coded representation of the likelihood score S, the actual value of *S*, the actual observed descriptor value and the position of this value in the CDF of the descriptor are given. Descriptors MFEahl index, polyA and GAsurplusCU are in the S<1 fraction outside 90% of the CDF.

The combined *L *score for a given miRNA hairpin was calculated as the product of all *S *values of descriptors considered in a scoring model. In Figure 8, the distribution of the resulting *L *scores for all miRNA hairpins from the taxonomic set 'Metazoa' is given for two different scoring models in a cumulative *L-*score plot. For the scoring model Metazoa (red), 31% of the known metazoan miRNA hairpins had an *L *score of 1.0. This means that for 1,227 miRNA hairpins, the *S *score of the LDF of each of the 18 descriptors was 1.0. A cumulative *L *score plot can be used to select a desired level of sensitivity: an arbitrarily chosen sensitivity of 90% is reached at an *L *of 0.0004 for the optimized scoring model Metazoa (red) and at an *L *of 0.032 for the initial scoring model Metazoa (blue). This hundred-fold difference is caused by adjusted parameterization and weighting of descriptors in the first model, resulting in a higher overall penalization. This example illustrates that the *L *score is a relative measure that depends on the scoring model. For all 3,984 miRNA hairpins used in this study, *L *scores were calculated for all 23 scoring models [see Additional file 4], which were all based on the subset of 18 descriptors. Figure 9 shows an example of a detailed descriptor report for cel-mir-51 for scoring model Metazoa. The report shows the individual descriptor values, positions of these values in the CDF of the descriptors and the transformed *S *scores. The resulting *L *score equals 0.057 due to the fact that three descriptors fall outside the 90% range of the CDF. Data for all 91,632 combinations of miRNAs (3,984) and scoring models (23) are available in the accompanying web document *μRNALL *[42]

### Comparison of the *L *score approach to threshold filtering

Comparison of our *L *score method to binary threshold filtering on miRNA hairpin descriptors showed superior performance of the *L *classifier (Table 3). For sets of miRNA and genomic hairpins that were filtered at the bordering value between the *S *= 1 and *S *< 1 fractions for all 18 descriptors, values for the performance parameters sensitivity, specificity and selectivity were obtained. Next, we kept either sensitivity or specificity constant, assessed at which *L *score this parameter was equalled and compared the other performance parameters. In both cases, our scoring model approach outperformed threshold filtering. When fixed at sensitivity, the scoring model approach achieved 17% better. Sensitivity was measured on all metazoan miRNA hairpins (3,902 hairpins) instead of on *C. elegans *miRNA hairpins (132 hairpins) because of the inaccuracy caused by the limited number of miRNAs in the *C. elegans *set (data not shown).

**Table 3:** Comparison of threshold filtering of miRNA hairpins with the L-score classifier

**Method**	**Sensitivity** **% (number)**	**Specificity** **% (number)**	**Selectivity **^**c**^	***L *score **^**a**^
Threshold filtering	56.8 (2,216)	99.57 (15,049)	133	-
Scoring model Metazoa				
Fixed at specificity	62.0 (2,421)	**99.57 (15,048)**	145	0.221
Fixed at sensitivity	**56.8 (2,216)**	99.64 (12,829)	156	0.280

^a ^Sensitivity measured on the taxonomic set Metazoa (3,902 miRNA hairpins)

^b ^Specificity measured on the set of 3,526,115 genomic hairpins in *C. elegans*

^c ^Selectivity calculated with sensitivity on metazoan miRNAs and specificity measured on *C. elegans *genomic hairpins

^d ^minimal *L *score of the scoring model Metazoa for which the performance of threshold filtering performance is equalled

### Predicting hairpins from a genomic sequence

We developed a procedure for predicting hairpin structures from genomic sequences using Vmatch [43]. The algorithm detects degenerate palindromic repeats and is therefore able to recover known miRNA hairpins with very high sensitivity. To benchmark the procedure, we predicted hairpin structures in the genomes *C. elegans *and four viruses: Epstein-Barr virus (EBV), Mareks disease virus (MDV), Human cytomegalovirus (HCMV) and Kaposi sarcoma-associated herpesvirus (KSHV). The number of recovered, known miRNA hairpins, number of identified hairpins and percentage and absolute number of non-overlapping hairpins on unique loci are given for three different *L *score criteria for the scoring model Metazoa, for both miRNA and genomic hairpins (Table 4).

**Table 4:** Identified (miRNA) hairpins in genomes of C. elegans and four viruses

Organism	Identified miRNA hairpins ^a^	Identified genomic hairpins	miRNA hairpins % (number) ^b^			genomic hairpin loci % (number) ^b^		
			L = 1.0	L ≥ 0.05	L ≥ 1e-5	L = 1.0	L ≥ 0.05	L ≥ 1e-5
*C. elegans*	128/132	3,526,115	34 (45)	71 (94)	89 (117)	0.1 (3,110)	0.6 (21,313)	2.8 (98,309)
EBV	23/23	6,182	35 (8)	87 (20)	100 (23)	0.3 (20)	2.6 (162)	13 (793)
MDV	8/8	5,374	25 (2)	100 (8)	100 (8)	0.3 (15)	1.3 (69)	6.1 (329)
HCMV	10/11 ^c^	9,747	40 (4)	90 (9)	100 (10)	0.3 (30)	2.5 (247)	12 (1,147)
KSHV	12/13 ^d^	4,296	17 (2)	83 (10)	92 (11)	0.5 (20)	2.2 (94)	10 (433)

^a ^Identified miRNA hairpins/number of known miRNA hairpins in genome(s) in miRBase version 9.0 [44].

^b ^Percentage of miRNA or genomic hairpin loci that have at least a certain *L*-score for scoring model Metazoa (absolute numbers between brackets).

^c ^MiRNA hcmv-mir-UL148D could not be mapped on the genomic sequence of HCMV [EMBL: X17043].

^d ^MiRNA kshv-mir-K12-10b could not be mapped on the genomic sequence of KSHV [EMBL: U75698HCMV].

In the four viral genomes, 25,599 hairpins were identified, including all 55 known miRNAs hairpins of these viruses. In *C. elegans*, 3,526,115 hairpins were predicted and only four out of 132 known miRNAs hairpins were missed (cel-mir-262, cel-mir-260, cel-mir-272 and cel-mir-256). When benchmarking the performance of the hairpin identification with the miRBase entries [44] of all metazoan miRNA hairpins, 3,803 out of 3,902 (97.5%) miRNA hairpins were recovered. The hairpin prediction algorithm is independent of sequence context (data not shown). This benchmark is therefore an estimate of the algorithm's good performance on metazoan genomes. Similar performance has been reported for other edit-distance based hairpins detection methods [26]. On a genome scale, all these methods yield around 10,000–20,000 hairpins per single stranded Mb of sequence. These hairpins are predominantly overlapping and nested.

### Analyses of miRNA hairpin candidates in viral genomes

The data in Table 4 show that only 1.3–2.6% of the indentified hairpin loci in four viral genomes have a high *L *score (*L *≥ 0.05). This corresponds to 69–247 loci per genome. In addition, 83–100% of all known miRNA hairpins comply with this threshold for *L*. Out of 6,182 hairpins predicted in the genome of EBV, only 23 hairpins, originating from 20 unique loci, had an *L *score of 1.0. Ten of these were experimentally validated miRNA loci [2] [see Additional file 5]. Further support for our miRNA prediction and scoring method comes from recently discovered miRNAs in the MDV genome [45] that were not included in miRBase 9.0. All five novel miRNAs (mdv1-mir-M9 to mdv1-mir-M13) were present in the set of here predicted hairpins and three of these had *L *score of 1.0 [see Additional file 6]. The 15 unique loci in MDV with an *L *score of 1.0 collapsed into eight unique sequences due to a large inverted repeat. Out of these eight, all five loci that did not overlap with annotated exons corresponded to known miRNA loci.

The two examples show that most hairpins in viral genomes with high *L *score are true miRNA hairpins and that the absolute number of hairpin loci with high *L *scores is small. This allowed us to manually examine the remaining, non-miRNA loci with high *L *scores, using additional filtering criteria for genomic location such as proximity to known miRNAs [2] and intronic position [44]. Among the remaining loci in MDV with *L *≥ 0.05, one appeared to be located in the transcribed strand of an intron and two others closely flanked (0.3 kb) the mdv1-mir-M1 gene in the same orientation [see Additional file 6]. Similarly, out of the remaining ten EBV loci with *L *= 1.0, two candidate miRNAs were located directly upstream and amidst a cluster of eleven known miRNAs in an intronic region of the BART gene [46] [see Additional file 5].

### Mining the *C. elegans *genome for putative miRNA hairpins

Using the scoring model Metazoa, the genome of *C. elegans *was searched for potentially novel miRNA hairpins. For the set of 3,525,115 genomic hairpins, a cut-off value *L *≥ 0.05 resulted in a reduction of the number of candidate miRNA loci to 21,158 (0.6%). For *L *= 1, only 3,099 hairpins loci remained, but the sensitivity measured from retrieval of known miRNA hairpins was only 34%. Nevertheless, this shows that filtering criteria in addition to the *L *score are required prior to experimental evaluation of candidate miRNAs. Such criteria may include the use of annotation data or genomic context. It has been suggested, for example, that metazoan miRNAs do not overlap with exons [11]. Indeed, in the annotation used here (Ensembl build 150), manual inspection of the genomic position of 132 known miRNAs of *C. elegans *showed that only two hairpins (2%) overlap with annotated exons (cel-mir-354, cel-mir-356, Table 5). Another very selective criterion is similarity to a known (metazoan) mature miRNA. We used this criterion such that similarity should be present in the stem of the hairpin and cover at least a 19 nt overlap with at most three mismatches. This resulted in a reduction to only 937 hairpin loci (Table 5). Another property of many miRNAs is their clustered occurrence, with 45 from 132 miRNAs in *C. elegans *separated by less than 5 kb [10]. This criterion limited the number of hairpin loci to 15,514 (Table 5). An example of a less selective criterion is removing all hairpins that fall in highly repetitive genomic areas. A catalogue of repeat regions can be obtained by an algorithm as Tandem repeats finder [47]. Additional selection can be accomplished with threshold filtering using the same (or a subset of) descriptors already used in our scoring model. Besides translating miRNA hairpin properties into a statistic for relative rating, as done in calculating the *L *score, a descriptor can be used as a binary decision criterion: below the threshold, the hairpin is rejected as potential candidate; above it is included. Which descriptor(s) to use for further filtering is in the hands of the individual researcher and may depend on the data studied. In the following, we provide two examples of such filtering using seven individual descriptors: four based on structure (stem length <= 55, loop length <= 40, largest bulge <= 8, max match count >= 17) and three based on sequence complexity (polyNucHairpin <= 8, SCS-mono >= -10, SCS-di <= 0.40). Except for stem length, all individual thresholds fall within their *S *< 1 fractions and separately reject at most three *C. elegans *miRNA hairpins. This ensemble of thresholds excludes low-complexity sequences, captures palindromic repeats with limited degeneracy and should comprise the vast majority of miRNAs. In total, only six *C. elegans *miRNA hairpins fail this filter and the same sensitivity (96%) is achieved on all metazoan miRNA hairpins. Only 30% of the set of genomic hairpins in *C. elegans *passes this filter, representing a selectivity of 3.3 (data not shown).

**Table 5:** Mining the C. elegans genome for putative miRNA hairpins

	Rejected miRNA hairpins ^a^	Clustered ^b^ (miRNAs/loci)		Similar ^b^ (miRNAs/loci)	
5 kb flanking sequence	NA	132	15,514	-	-
Similarity to mature miRNA ^c^	NA	-	-	132	937
No overlap with exons ^d^	2	130	11,197	130	706
L = 1.0	87	45	80	-	-
L >= 1e-5	15	-	-	116	197
Exclude TRF overlap ^e^	0	45	74	-	-
Filter on 7 descriptors	6	-	-	116	162

Exclude known miRNA loci	132	0	20	0	64
similarity positioned correctly ^f^	18	-	-	0	41

^a ^Rejected *C. elegans *miRNA hairpins by this step alone

^b ^Remaining cumulative number of *C. elegans *miRNA hairpins; remaining cumulative number of genomic hairpin loci

^c ^Similarity to a metazoan mature miRNA of at least 19 nt length with at most three mismatches

^d ^Cel-mir-354 is fully located in the final exon of Y105E8A.16; cel-mir-356 is largely located in the 3'utr of ZK652.2, but overlaps 6 nt with the final exon

^e ^At most 40 nt overlap with a repeat identified by Tandem Repeats Finder [47]

^f ^Similarity with mature miRNA may have at most 3 nt overlap with hairpin loop coordinates and at least 8 nt of the stem must separate the similarity from the end of the hairpin. Criteria are set according to the biological model of miRNA maturation from hairpins [3].

**Figure 10:**
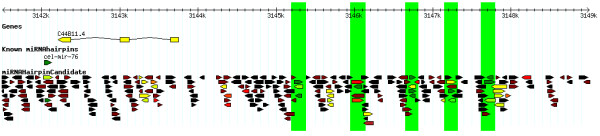
**A cluster of candidate miRNA hairpins in *C. elegans *3 kb upstream of cel-mir-76**. Five candidate miRNA hairpin loci with *L *score = 1 on chromosome III of *C. elegans*, selected by the filtering protocol Clustered. Loci are marked by green bars. Three out of five loci have hairpins with *L *score = 1 on both strands (positive strand: 3145224–3145336, 3146698–3146781, 3147197–3147283 and 3147660–3147798; negative strand: 3145240–3145320, 3145991–3146089, 3146703–3146775 and 3147690–3147767). The *L *score of genomic hairpins is indicated by a color gradient that ranges from dark green (*L *= 1) over yellow (*L *= 1e-4) and red (*L *= 5e-7) to black (*L *= 0).

**Figure 11:**
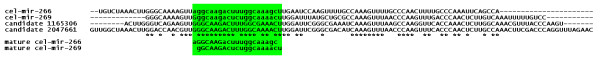
**Candidate miRNA hairpins in *C. elegans *closely related to cel-mir-266 and cel-mir-269**. ClustalW alignment of the hairpin sequences of cel-mir-266, cel-mir-269 and the genomic hairpins 1,165,306 (chr I, 1733470..1733572 (+), *L *score = 0.030, 12^th ^intron of F54F11.2) and 2,047,661 (chr II, 13515555..13515672 (+), *L *score = 7.2E-3, 7^th ^intron of Y71G12B.11). The position of the mature miRNA sequences of cel-mir266 and cel-mir-269 (in lowercase) is projected on the sequences in green. Lowest two lines show again the mature miRNA sequences of cel-mir-266 (MIMAT0000325) and cel-mir-269 (MIMAT0000322), with their seed sequence in uppercase.

In the following examples, filtering on *L *score was combined with filtering on either genomic context or on a similarity threshold to known metazoan mature miRNAs (we refer to these protocols as "Clustered" and "Similar"). Goal of these filtering protocols was to achieve sets feasibly sized for manual inspection and/or laboratory evaluation. The protocols comply with characteristics of miRNAs mentioned above: occurrence in clusters and presence in families. "Clustered" and "Similar" resulted in lists of 20 and 64 candidate miRNA loci [see Additional files 7 and 8], respectively, that were manually inspected. The most compelling cases among these are presented in Figure 10 and Figure 11. Figure 10 shows a cluster of hairpins of which several have *L *= 1, starting 3 kb downstream of cel-mir-76. Figure 11 shows two conspicuous hairpins that share similarity with the two known mature miRNAs cel-mir-269 and cel-mir-266 (hairpins 1,165,306 and 2,047,661, with *L *scores of 0.030 and 0.007, respectively). The multiple alignment of the four hairpin sequences revealed the characteristic camel-shaped conservation pattern that is often observed between related miRNAs [28]: high or perfect conservation in the stem of the pre-miRNA hairpins, low conservation in the hairpin loop and the up- and downstream stem sequences. The 5' seed sequences of both mature miRNAs (position two to seven) are exactly conserved in the novel hairpins, suggesting that hairpins 1,165,306 and 2,047,661 are likely members of the miRNA families to which cel-mir-269 and cel-mir-266 belong [10]. Furthermore, candidate 1,165,306 is located in the 12th intron of the gene F54F11.2, for which the *C. elegans *unigene set (build 28) [48] provided evidence of transcription. Cel-mir-269 and cel-mir-266 were predicted by comparative computational approaches and confirmed by a PCR amplification protocol, but their precise mature miRNA ends are unknown [36,44]. Recent high-throughput sequencing of miRNAs from *C. elegans *[10,16] could not confirm the existence of both miRNAs.

The data presented for the filtering protocols "Clustered" and "Similar" (Table 5) show that combined filtering on *L *score, genomic context and threshold filtering allows for compilation of a priority list of candidate miRNAs that is amenable to manual inspection and experimental verification. Apart from filtering on genomic clustering or similarity to known miRNAs, filtering on *L *score attains the largest data reduction. This shows that the *L *score was important in compilation of the priority list and demonstrates the added value of our approach for *in silico *miRNA prediction.

## Discussion

We here present a new computational strategy for the *in silico *prediction of miRNA hairpins and show the applicability of the method for predicting new candidate miRNA hairpins in four viral genomes and in the genome of *C. elegans*. While using the latter as an example for model construction, the *L *score method as here optimized for *C. elegans *is well usable for other metazoan genomes. However, further improvement of performance of the method on different taxonomic groups can be achieved by constructing dedicated scoring models [see Additional file 9]. Our strategy aims at minimizing the number of false negative predictions (optimal sensitivity), rather than at minimizing the number of false positive predictions (optimal specificity), as proposed in previous studies. Focusing on sensitivity rather than specificity should help uncover new classes of miRNA molecules in biological systems. Hairpins in genomic sequences are identified with the help of an adjusted suffix-tree based method. When the performance of the hairpin prediction was benchmarked on sets of all known miRNAs hairpins from viruses and Metazoa, all 55 viral miRNAs were recovered (100%), 128 of 132 (97%) *C. elegans *miRNAs were recovered and 3,803 out of 3,902 (97.5%) metazoan miRNAs were shown to be recoverable when considered in their genomic context. Similar performance was reported for another edit distance-based hairpin identification method [26].

Four *C. elegans *miRNAs (cel-mir-262, cel-mir-260, cel-mir-272 and cel-mir-256) remained undetected due to the absence of a stringently base-pairing area in their stems. They all had extremely poor *L *scores (2.7e-41, 2.5e-27, 3.5e-20 and 3.8e-10), ranking first, third, fourth and eight among the *C. elegans *miRNA hairpins with lowest *L *scores. None of the four was found in recent high-throughput sequencing datasets, which otherwise retrieved the vast majority of known *C. elegans *miRNAs [10,16]. This suggests that these four may not be genuine miRNAs. If so, the reported performance of our hairpin prediction method is underestimated.

The biological variation and evolutionary diversity of various properties of miRNA hairpins were captured in a likelihood score *L*, based on statistics derived from accurately fitted (generally skewed normal) distributions of hairpin characteristics derived from known miRNA hairpins. In total 40 hairpin characteristics were defined and analyzed. The *L *score is a measure for a single hairpin sequence: a descriptor that captures evolutionary conservation in another species is not included. Although conservation has proven to be an extremely selective miRNA detection criterion [28], it conflicts with the aim to maximize sensitivity, because of the existence of species-specific miRNAs. The strategy was evaluated on genomic hairpins and known miRNAs from the *C. elegans *genome. Although details of analyses and results are likely to differ when applied to other genomes and other negative sets of genomic hairpins, major trends and results were shown to be similar [see Additional file 9].

Based on analyses of correlation and discriminative power, 18 hairpin characteristics were identified as most selective. Comparison of the 18 descriptor scoring model with a binary threshold filtering protocol using the same 18 descriptors (Table 3) shows that a 17% higher selectivity is achieved with the *L *score strategy. Binary decision thresholds on all or even a few descriptors can easily result in a major decrease of sensitivity. In the strategy developed here, *L *< 1 represents individual sequences that have one or more descriptors with *S *< 1, indicating that these descriptors have a relatively low probability of occurrence in miRNA hairpins because they occur in the tail(s) of their respective distribution. When a descriptor value falls outside the observed range of biological variation, the continuous likelihood score *L *allows for the compensation of an unlikely score for a single descriptor by likely scores for other descriptors. In such a case, the sequence is not *a priori *rejected as a miRNA hairpin candidate. The *L *score thus allows for more deviations from 'genuine' miRNA characteristics than binary selection (yes/no) on the basis of the same characteristics. This is an important improvement over the use of pre-defined thresholds for filtering on single or multiple descriptors published previously [19]. Assigning scores to descriptors, as opposed to binary selection on pre-defined thresholds, has also been used in previous work. For example, MIRscan [5,6] employs an heuristic score assignment to seven features and assigns weights based on the relative entropy between known miRNA hairpins and genomic hairpins. PalGrade [8] uses a statistical distribution by arbitrarily binning the ordered vector of descriptor values. SVM kernels achieve descriptor scoring and weighting as part of the SVM [35]. Novel to the approach here developed is that the score assignment is based entirely on the statistical evaluation of the variation in physical and sequence properties observed in known miRNAs and no binary selection prior to building a scoring model is used.

The *L *score is a relative measure of the likelihood that a given hairpin is a miRNA hairpin candidate. An important parameter contributing to useful *L *scores is the number of miRNAs used to derive the discriminating statistics of the individual characteristics. A minimum of about a thousand miRNA hairpins is required, but results indicate that the larger the available data set, the better the scoring model captures the variation in miRNA hairpin characteristics and performs. In the context of machine learning, the input miRNA set could be considered a training set, although in the *L *score derivation no formal 'training' is included. It is noteworthy that the evolutionary distance between the species contained in the taxonomic set for a scoring model influences *L *considerably. In general, the scoring model based on the set that is taxonomically closest to the organism being analyzed performs best. This indicates that over a wide range of characteristics, miRNA hairpins within (related) species are substantially more alike than miRNA hairpin sequences between less-related species. This should be taken into account when searching for similarity between miRNA hairpins from distantly related species. However, we observed that, for example, taxonomic sets that do not contain human miRNA hairpins can yield scoring models that accurately identify human miRNA hairpins. In addition, descriptor weighting and parameterization appeared to considerably influence the performance of a scoring model. The analyses presented allow the selection of optimal scoring models, with maximal discriminative power to distinguish true miRNA hairpins from other genomic (or random) hairpins for a selected set and a given data set. However, each set of data, for example in case of a new genome sequence, will require its own analysis to build an optimal scoring model.

Whereas the analyses started with 40 descriptors, based on extensive correlation and selectivity analyses, a subset model based on 18 descriptors was performing better than the model comprising all 40 descriptors. In view of the importance of the taxonomic composition of the set used in the model, it should be pointed out, that this may reflect a taxonomic bias for metazoan sequences that may not be valid for other taxonomic groups, such as, for example plant miRNA hairpins. In the set of 18 most informative descriptors selected, it is remarkable that three descriptors that are generally considered important are not represented: GC content and the MFE randomization descriptors P and Z. GC-content is widely used as a pre-filtering step of *in silico *miRNA prediction methods [36], but is not among the 18 descriptors used in the final scoring models. The GC-content showed the highest variability in the (skewed-normal) mean over different taxonomic sets (data not shown), reflecting the apparently large variation in GC-content found among miRNA hairpins from different species. Any scoring model that includes the fitted distribution of GC-content would disqualify hairpin structures in species containing miRNAs with relatively high or low GC-content. Also the descriptors P and Z, based on MFE randomization shown to be significantly lower for miRNAs hairpins than for randomized sequences [37,38], were not among the18 descriptors selected. Although P and Z ranked among the most selective descriptors, they were excluded because of their strong correlation [see Additional file 3] with the most selective descriptor MFEahl index, in which the MFE is adjusted for hairpin length and GC-content.

SVMs have similar aims as the *L*-score strategy and perform well in miRNA classification [35]. In assessing and comparing the performance of different methods, however, several caveats should be considered. First, methods that use evolutionary conservation perform well on conserved miRNAs [25,11] but fail to detect species-specific or fast evolving miRNAs [12]. The importance of the latter should not be underestimated. Second, the particular data set(s) on which the performance is achieved is important to the evaluation and comparison of the results from different methods. Unfortunately, there is no benchmark set of both positive and negative examples of miRNA hairpins available. Many methods are tailored on a specific organism and are likely to perform best in that exact context. Assembling a set of true negative sequences is particularly challenging: hairpins in non-coding RNAs, e.g. the set of tRNAs as used in [25], are likely to possess different and more diverse hairpin features than miRNA hairpins, whereas a set of genomic hairpins might contain *bona fide *miRNA hairpins. SVMs explicitly require negative examples for training and testing, whereas the *L *scoring method uses such a set only for benchmarking purposes. Third, data on prediction performance are not reported consistently in literature. Our method enables reporting of AUC performance as well as sensitivity and specificity values over the entire range of the ROC curve. Comparison with binary classifiers from other methods therefore requires transformation of the continuous outcome to a binary outcome by choosing an arbitrary threshold for *L *and using the associated sensitivity and specificity values as measure of the performance.

With these caveats in mind, we compared the performance of our method to three leading SVM-based methods *miPred *[35], RNAmicro [25] and miRNA SVM [26] Note that RNAmicro is based on multiple sequence alignments. Using different positive and negative datasets, these methods report the following values of sensitivity and specificity; 1) *MiPred*: 86.69% and 97.68%, using 323 human miRNAs as positive and 646 human genomic hairpins as negative set; 2) *MiPred*: 87.65% and 97.75%, using 1,918 Metazoan, non-human miRNAs as positive and 3,836 human genomic hairpins as negative set; 3) RNAmicro: 90% and 99%, using 147 Metazoan miRNA hairpin alignments as positive and 383 shuffled miRNA hairpin and tRNA alignments as negative set, and; 4) miRNA SVM: 90% and 95%, using 322 human miRNAs as positive and 3,000 random human genomic hairpins as negative set. These performances compare well to the values of 87.26% and 97.02% obtained in the 10 fold-cross validated performance of our *L *score model, using 203 Metazoan miRNA hairpins as positive and 200,000 randomly selected genomic hairpins from *C. elegans *as negative set. The performance of the *L *scoring model is most similar to that of *miPred*, which does not include sequence conservation as a parameter. An analysis of the performance of our model on sets of genomic hairpins other than those derived for the *C. elegans *genome is provided as Additional file [see Additional file 9].

A major challenge of any SVM is understanding its behavior in, for example, a biological context. While SVMs are known to produce classifiers that perform well in case of unseen data [49], SVMs are essentially black-box classifiers. This makes it difficult to judge the relative importance of individual descriptors or to translate results in biologically relevant understanding. As all parameters are embedded in the kernel function of the SVM, SVM classifiers are also difficult to adjust, although they do not require the pre-selection of parameters required for the *L *score strategy here presented. Classifier selection based on the detailed descriptor analysis presented here may improve future SVM approaches. A key advantage of the *L *score strategy over SVMs is that the contribution of individual descriptors to a scoring model can be analyzed in a straightforward way by adding or removing a descriptor or changing the parameterization or weight of descriptors. This way the scoring model becomes better tailored to the biologist's needs in a particular research environment.

## Conclusion

At the laboratory bench, the criterion that is of most interest is simply how many putative hairpins should be evaluated by experimentation. With current developments in microarray analysis and high-throughput sequencing, the numbers of potential candidates that can be screened with relative ease will increase dramatically. Still large numbers of new miRNAs may be identified. Yet, for the time being, the individual laboratory would like to see as little putative candidates as possible with as high a success rate as feasible. The highest *L *score is 1, implying that the given hairpin scores are maximal for all descriptors in the model. In the 100 Mb large genome of *C. elegans*, still 3,110 hairpin loci remain that cannot be ranked further on the basis of *L*. It implies that relatively large numbers of genomic hairpins (3,110 loci from 3,526,115 hairpins, i.e. 0.09%; see Table 4) comply with all miRNA hairpin descriptors, whereas it is unlikely that they all generate mature miRNAs, given the relative small number of 132 currently known *C. elegans *miRNAs. It is likely that this situation will occur in most genomic contexts. If so, several strategies are open. The model parameters could be adjusted, so that the individual descriptor is less likely to get the maximal score. This way, the *L *score approach will convert to more traditional threshold filtering. Given that the analysis requires high sensitivity, it would however be more advantageous to incorporate more biological expert knowledge in the selection process, such as the presence of the hairpin in an intron, sequence similarity to known miRNAs, etc. When following this strategy, one has to be aware that the number of novel miRNAs that could be discovered is constrained by the filtering on genomic context. For example, in the set of 132 miRNAs in *C. elegans *(miRBase 9.0), 87 occur in singletons when clustered on distance, 56 have no other family representative and 102 are not located in an intron [10]. These numbers can thus be considered as indicative for the fraction of true miRNAs that will remain concealed when filtering on genomic context. With the filtering protocols ("Clustered" and "Similar") we show that a combination of filtering on *L *score, genomic context and threshold filtering allows for compilation of a priority list of manageable size for manual inspection and further experimentation.

In addition to good performance in comparison with other leading (SVM-based) methods and a user-defined selectivity, an additional advantage of the *L *score approach over threshold filtering and support vector machine classifiers is that the prior analysis of taxonomically defined sets and fitted distributions, correlations, and discriminative power of descriptors gives detailed insight in the behavior of a scoring model and can accommodate expert knowledge. It should therefore appeal to the experimental biologist, despite the fairly time-consuming construction of a suitable scoring model.

The scoring model proposed here is independent of the hairpin prediction step and can therefore be coupled to any *in silico *or experimental miRNA prediction method. It can facilitate the analysis of large sets of putative miRNA hairpin loci obtained in deep-sequencing efforts of small RNAs [10,14-16]. The *L *score approach can be used to rank and select interesting miRNA hairpin candidates for downstream experimental analysis in search for novel miRNAs. Moreover, our in-depth analyses of known miRNA hairpins from miRBase [44], our detailed descriptor analyses (Figure 9) and the *L *score approach here presented are likely to increase the reliability and evidence of miRBase entries and will help to further increase the biological relevance of the miRBase repository.

## Methods

### Sequence and annotation data

The complete set of 3,498 non-plant miRNA hairpin sequences were retrieved from the web resource miRBase version 9.0 [44]. In addition, 474 miRNA hairpin sequences from human and chimpanzee [12] and 18 from *C. elegans *[10] were obtained from the supplementary material of the respective publications. Secondary structures of the sequences were predicted using RNAfold version 1.6 [21] with the constrained folding option (-C) used to position the mature miRNA sequence(s) in the stem of the hairpin. The hairpin structure of six sequences, three from miRBase and three from the human/chimpanzee set [12], deviated considerably from the predicted characteristics of miRNA hairpins. These six sequences were therefore excluded from all subsequent analyses [see Additional file 10]. The resulting 3,984 miRNA hairpin sequences were included in this study, 3,902 from Metazoa and 82 from virus genomes. Sequence and annotation of the *C. elegans *genome (build 150) was obtained from Ensembl [50]*C. elegans *unigenes (build 28) were downloaded from NCBI UniGene [48]. Viral genome data for the Epstein-Barr virus [EMBL: AJ507799], Human cytomegalovirus [EMBL: X17403], Kaposi sarcoma-associated herpesvirus [EMBL: U75698] and Mareks disease virus [EMBL: AF243438] were obtained from EMBL [51].

### Informatics and statistics

Supplemental data are available through the Additional data files and the accompanying web document *μRNALL*, which can be downloaded at [42]. All statistical analyses were performed using the package R [52], as integrated in python through Rpy [53].

### Definition of miRNA descriptors

A set of 40 potentially discriminative features of miRNA hairpins, hereafter referred to as descriptors, was defined based on the set of 3,984 miRNA hairpins. The descriptors include both physical and sequence characteristics of miRNA hairpins [Table 1; see Additional files 1 and 2]. A subset of descriptors is given in Table 1. To take the evolutionary diversity of the descriptors into account in the statistical analyses, miRNA sequences were divided in hierarchically organized subsets based on their taxonomic relationships. Taxonomic sets that comprised at least 100 sequences were used for analysis. In total, 23 taxonomic sets were defined [see Additional file 4], including one set containing all metazoan miRNA sequences, eleven sets representing metazoan taxa and eleven species-specific sets. The virus set was not used because it contained only 82 sequences. Unless stated otherwise, all results presented in this paper use the combined taxonomic set 'Metazoa' (3,902 miRNAs).

### Individual likelihood score *S *for each descriptor

For all 3,902 sequences, descriptor values were calculated and their distributions within each of the 23 taxonomic sets were fitted to an appropriate probability distribution. Goodness-of-fit was determined by a Chi-square test. For each distribution, the probability that the descriptor takes a value less than or equal to a specified value was calculated as the cumulative distribution function (CDF) and transformed into a likelihood distribution function (LDF). For the LDF, a default cut-off value was set at 0.05, corresponding to the 95% confidence interval of the fitted distribution of the descriptor. For each descriptor, values of the CDF above the cut-off value were transformed to the LDF likelihood score *S *= 1. Values below the cut-off were transformed to the likelihood score *S *= (CDF/cut-off). Table 1 and Additional file 2 list for each descriptor whether the lower tail of the CDF, upper tail or both were transformed. As a result of this transformation, each descriptor in the taxonomic set has a likelihood distribution *S *comprising an *S *< 1 and an *S *= 1 fraction. *S *= 1 indicates a descriptor for which characteristics of the individual sequence are in 95% of the distribution.

### Likelihood score *L *for the combined descriptor values

To obtain a single metric for a given taxonomic set, the likelihood scores *S *for all descriptors were multiplied to obtain the combined likelihood score *L*. The ensemble of likelihood scores *S *for a given set of hairpin sequences is referred to as the scoring model. *L *is the outcome of the scoring model and functions as classifier for miRNA hairpin sequences. *L *ranges between 0 and 1 and represents the likelihood of a hairpin sequence to be a true miRNA hairpin given the underlying descriptors used in the scoring model. It is possible to incorporate additional expert knowledge in the scoring model by assigning a relative weight to the *S *score of an individual descriptor. In the default setting reported here, no difference between descriptors is made (assigned weight = 1). An *L *score of 1.0 for a hairpin sequence indicates that *S *= 1 for each descriptor of the set. *L *is only affected by descriptors with a value *S *< 1.

### Correlation and discriminative power of descriptors

To prevent potential over-penalization of hairpin sequences when combining correlated descriptors, we determined the independence (orthogonality) of all descriptors in the *S *< 1 fraction by calculating Cohen's kappa [41] for each combination of descriptors. The value *κ *= 0 indicates that there is no more correlation between descriptors than expected by chance alone, and *κ *= 1 indicates that the descriptors are fully dependent. The discriminative power of a descriptor, i.e. its ability to distinguish true miRNA hairpins from non-miRNA hairpins, was calculated as the ratio of percentages of miRNA hairpins and genomic hairpins that comply with a given threshold for this descriptor. As threshold the descriptor's limiting value between *S *= 1 and *S *< 1 of the LDF was chosen (95% of the CDF). Discriminative power was calculated using known miRNA hairpins from the taxonomic set Metazoa and genomic hairpins from a set of 3,526,115 hairpins identified in *C. elegans *(see section Identification of putative miRNA hairpin structures). It is calculated with the formula for selectivity (see below), but for the sake of clarity we will here use the term 'discriminative power' for the performance of a single descriptor and the term 'selectivity' for the performance of a scoring model.

### Descriptor selection and model evaluation

To select a subset of descriptors that was most informative for the combined assessment of miRNA hairpins by the *L *classifier, descriptors that either correlated with a more discriminative descriptor (*κ*>0.4) or that showed low discriminative power (< 1.1) were discarded from the initial set. The resulting subset was used to evaluate the impact of different settings of variables. For all models, *L *scores were calculated for 100,000 randomly selected hairpins from the *C. elegans *genome. We evaluated (1) the effect of the size of the input set, which refers to the number of miRNA hairpins in a given taxonomic set; (2) the impact of evolutionary distance between taxa; (3) the impact of different combinations of descriptors in a scoring model; (4) the effect of parameterization of descriptors and (5) the effect of weighting of descriptors.

### Performance of *L*

The performance of the outcome classifier *L *of scoring models was measured in two ways. First, by the area under a receiver operating characteristic (ROC) curve [54]. Trapezoids were constructed as approximation of the Area Under the Curve (AUC). Unless described otherwise, ROC curves were made for the taxonomic set of metazoan miRNA hairpins (3,902) versus 200,000 randomly selected hairpins from the *C. elegans *genome. Second, sensitivity, specificity, and selectivity were calculated for each scoring model from the counts of true and false positive and negative cases (TP, FP, TN, and FN, respectively) in the following way:



TP and FN were counted from taxonomic sets of known miRNA hairpins, TN and FP were determined as a fraction of genome-wide identified hairpins. Although these sets of genomic hairpins contained an unknown number of true miRNAs (so FN and TP), this number was expected to be sufficiently small to be ignored. For uniform comparison, we benchmarked selectivity at discrete values of sensitivity (95% and/or 75%). Discrete points on the ROC curve correspond to pairs of sensitivity and specificity values, and as such describe the shape of the curve.

The performance of the classifier *L *was compared with sensitivity, specificity and selectivity of threshold filtering on descriptors of miRNA and genomic hairpins. For the 18 most informative descriptors, the threshold used did represent the same cut-off value between the *S *= 1 and *S *< 1 fraction of the LDF, at 95% of the CDF at the side(s) of the distribution as listed in Table 1. This cut-off was used as a binary decision criterion: below the threshold, the (miRNA) hairpin was included; above it was rejected.

A 10-fold cross-validation was performed on 200,000 randomly chosen genomic hairpins and repeated ten times. As input set, a non-redundant variant of the taxonomic set Metazoa was constructed. This involved clustering miRNA hairpins with identical mature miRNA seed sequences and an overall hairpin sequence identity larger than 90%. All but a single representative for each cluster were then removed, yielding a subset of 2,033 sequences.

### Identification of putative miRNA hairpin structures

The suffix-tree based tool VMatch [43] was used to identify small genomic hairpin structures in the genomes of *C. elegans *and four viruses, using a sliding window of 1,000 nt with an overlap of 200 nt. The latter value exceeds the length of the largest known metazoan miRNA hairpin (153 nt). Each sequence window was stored as a VMatch database (index) and its reverse complement was used as query sequence in a VMatch search for degenerate palindromic sequences, allowing GU-base pairing. Parameter settings that allowed exhaustive retrieval of known miRNA hairpins were found empirically (data not shown). Such a palindromic sequence consists of two inverse complementary sequences for the stem, at a physical distance representing the loop of a putative hairpin. Palindromes were discarded if the distance was larger than 50 nt, which represents the upper limit of loop size in the vast majority of metazoan miRNAs. Overlapping palindromes were merged if they had at most 8 non-overlapping nucleotides on either side. With these parameter settings, only a small number of known miRNA hairpins was missed. The remaining set of palindromic sequences was used for secondary structure prediction using RNAfold version 1.6 with the constrained folding option (-C) to enforce the stem structure in the folding of the molecule [21]. All hairpin structures were filtered for five threshold values: (1) minimal hairpin length = 45 nt; (2) minimal number of base pairs in the stem = 15; (3) minimal number of paired bases in the most stringently paired window of 24 positions in the hairpin stem = 15; (4) maximum length of a bulge in the stem = 29 nt; (5) minimal ratio of the number of paired positions divided by all positions in the stem (match-ratio) = 0.45.

### Grouping identified genomic hairpins into unique loci

Many of the genomic hairpins identified were overlapping or nested. Such hairpins were grouped into unique loci when the centers of their loops were less than 20 nt apart, regardless of the strand on which the hairpins were located.

## Abbreviations

AUC: Area Under the (ROC) Curve; CDF: cumulative distribution function; EBV: Epstein-Barr Virus; HCMV: Human cytomegalovirus; KSHV: Kaposi sarcoma-associated herpesvirus; LDF: likelihood distribution function; MDV: Mareks disease Virus; MFE: minimal folding energy (kcal/mol); miRNA: microRNA; ROC: Receiver Operating Characteristic; RSSP: RNA secondary structure prediction; siRNA: small-interfering RNA; SN: Skewed Normal distribution; SVM: Support Vector Machine.

## Authors' contributions

AvdB designed the study, performed the programming and analyses, drafted the manuscript and added all additional analyses for the revision. MF participated in the design of the study and supported the computational work. JPN and RvH contributed to the design of the study, supervised and participated in the preparation of the manuscript. All authors were involved in discussions, have read and approved the final manuscript.

## Supplementary Material

Additional file 1 **Data fit of descriptors**. Results of the data fit for 40 descriptors from the taxonomic set Metazoa (3,902 miRNA hairpins). Click here for file

Additional file 2 **Detailed explanation of descriptors**. A set of 40 potentially discriminative features of miRNA hairpins, referred to as descriptors, was defined and includes both physical and sequence characteristics of miRNA hairpins. Click here for file

Additional file 3 **Descriptor interdependency**. Correlation among descriptors in their S<1 fractions, assessed by Cohen's kappa coefficient *κ *of all 780 possible pairs of descriptors, using the miRNA hairpins of the taxonomic set Metazoa. Click here for file

Additional file 4 **Taxonomic sets with at least 100 miRNA sequences**. MiRNA sequences were divided in hierarchically organized subsets based on their taxonomic relationships. A total of 23 taxonomic sets comprised at least 100 sequences and were used for analysis. Click here for file

Additional file 5 **Hairpins identified in Epstein-Barr Virus**. Details of 23 hairpins with *L *score = 1.0, identified in Epstein-Barr Virus [EMBL: AJ507799] for the scoring model Metazoa. Click here for file

Additional file 6 **Hairpins identified in Mareks disease Virus**. Details of 18 hairpins with *L *>= 0.30 identified in Mareks Disease Virus [EMBL: AF243438] for the scoring model Metazoa. Click here for file

Additional file 7 **Hairpin loci in *C. elegans *obtained by the filtering protocol "Clustered"**. Filtering on *L *score was combined with filtering on genomic context, a protocol referred to as "Clustered". Click here for file

Additional file 8 **Hairpin loci in *C. elegans *obtained by the filtering protocol "Similar"**. Filtering on *L *score was combined with filtering on a similarity threshold to known metazoan mature miRNAs, a protocol referred to as "Similar". Click here for file

Additional file 9 **Performance of the scoring model Metazoa**. Analysis of the performance of the scoring model Metazoa on sets of genomic hairpins other than those derived for the *C. elegans *genome. Click here for file

Additional file 10 **MiRNAs that excluded from the analyses**. The hairpin structure of six sequences deviated considerably from the predicted characteristics of miRNA hairpins and were excluded from all subsequent analyses. Click here for file
