# Supplementary material for: In silico miRNA prediction in metazoan genomes: balancing between sensitivity and specificity
Source: BMC Genomics. 2009 Apr 30;10:204. doi: 10.1186/1471-2164-10-204 (PMC2688010; doi:10.1186/1471-2164-10-204)
Supplement: Additional file 1 — Data fit of descriptors. Results of the data fit for 40 descriptors from the taxonomic set Metazoa (3,902 miRNA hairpins). [file 1471-2164-10-204-S1.pdf]

Additional File 2: Data fit of 40 descriptors from the taxonomic set* Metazoa *(3902 miRNA hairpins).

Descriptor data is fitted to a Skew-Normal (SN) distribution with parameters location, scale and shape (*Azzalini and Capitanio 1999*). The `normal` mean of the data is given too.
* Fit-parameter *Lambda* of an exponential distribution corrected for existence of zero-values (descriptor P).

descriptor	Location	Shape	Scale	Mean	Chisquare
MFEahl index	0.745	2.18	0.253	0.926	0.0
MFEasl index	1.70	2.57	0.630	2.17	0.0
MFEindex	61.10	1.75	24.64	78.05	0.0
MaxDiBaseRatio	0.032	5.69	0.080	0.094	0.0
MFE	-27.70	-2.17	13.50	-37.36	0.0
MFEahl	0.365	1.53	0.115	0.442	5.54e-10
MFEasl	0.845	1.72	0.273	1.03	5.05e-12
P	24.96 *	None	None	None	None
Z	-5.90	0.600	2.27	-4.97	0.0
GAsurplusCU	-7.68e-02	1.13	0.096	-1.96e-02	0.0
GC-content	0.384	2.28	0.140	0.486	1.39e-05
GCratio	0.866	3.78	0.467	1.21	7.66e-15
GsurplusC	0.011	0.928	0.131	0.082	5.59e-08
GU-match contribution	0.088	1.72	0.102	0.159	0.0
minimal base occurence	0.223	-4.08	0.053	0.182	5.27e-09
polyA	1.69	3.39	1.56	2.87	0.0
polyAstem	1.66	2.81	1.42	2.73	0.0
polyC	1.61	3.53	1.44	2.71	0.0
polyCstem	1.56	3.37	1.43	2.63	0.0
polyG	1.76	7.33	1.51	2.94	0.0

descriptor	Location	Shape	Scale	Mean	Chisquare
polyGstem	1.74	6.98	1.49	2.90	0.0
polyNucHairpin	2.73	4.88	1.54	3.93	0.0
polyNucStem	2.67	3.52	1.45	3.78	0.0
polyU	2.02	2.58	1.59	3.20	0.0
polyUstem	1.86	2.77	1.56	3.03	0.0
SCS-di	0.195	3.77	0.061	0.241	0.0
SCS-mono	42.98	-9.83e-01	17.94	32.94	7.03e-08
bulgeRatio	0.020	3.79	0.089	0.088	0.0
D	0.013	6.19	0.036	0.040	0.0
dP	0.390	-1.18	0.034	0.369	0.0
gapratio	0.147	-6.85e-01	0.037	0.131	0.0
hairpin length	70.56	2.10	19.69	84.63	0.0
largest bulge	1.43	5.20	2.35	3.24	0.0
longest match-stretch	6.13	5.08	5.56	10.33	0.0
max match count	21.57	-1.71	2.10	20.12	0.0
match ratio stem	0.828	-1.15	0.084	0.777	0.0
looplength	2.95	569.23	8.43	7.95	0.0
Q	0.031	7.30	0.100	0.104	0.0
stem length	30.27	1.64	8.89	36.30	0.0
stem symmetry	-1.75e-04	857.09	0.042	0.027	0.0

MFEahl index

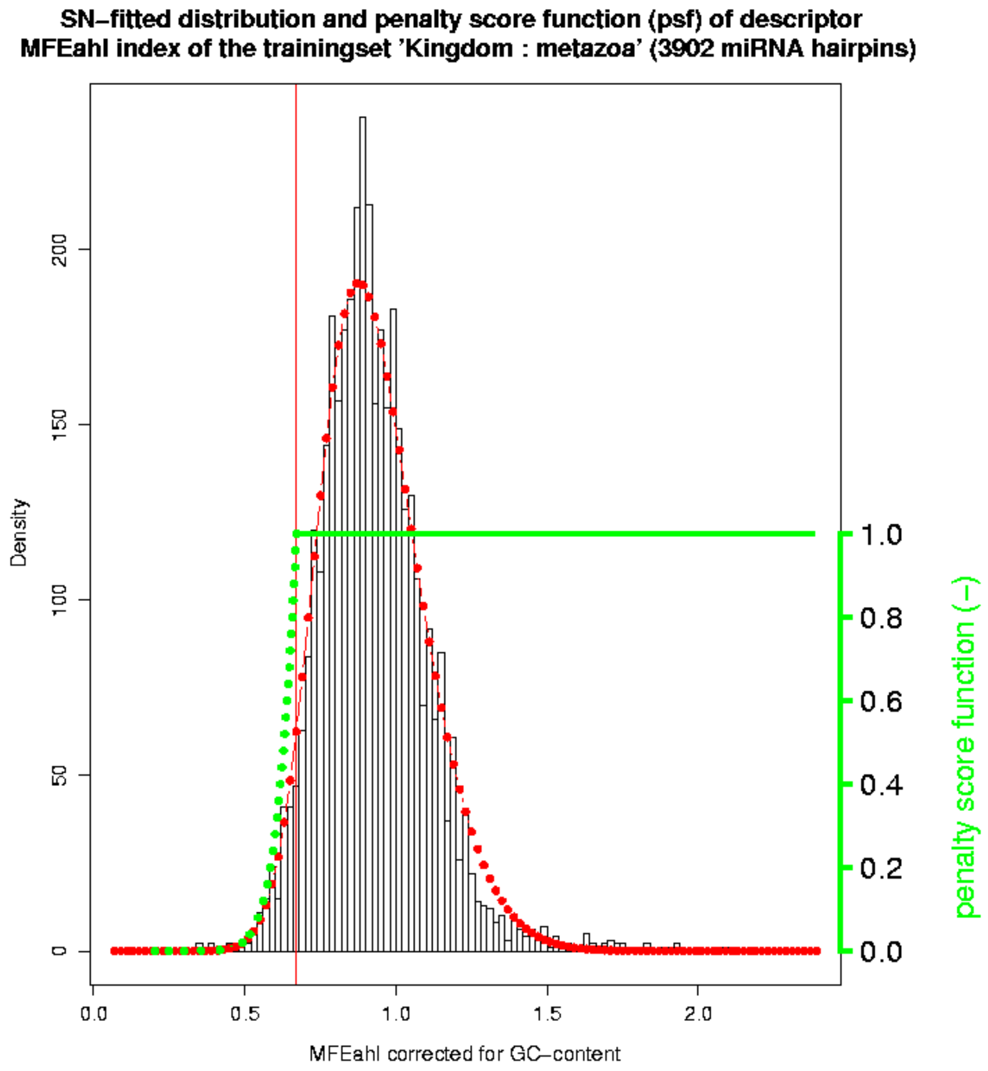

QQ-plot

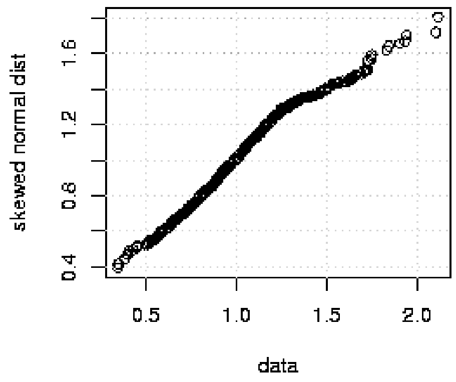

**Chisquare:**	0.0
**Location:**	0.744535
**Scale:**	0.253056
**Shape:**	2.17643
**(Normal) mean:**	0.925786
**Observed max:**	2.112
**Observed min:**	0.343478
**S<1 cut-off (95%):**	0.67036

MFEasl index

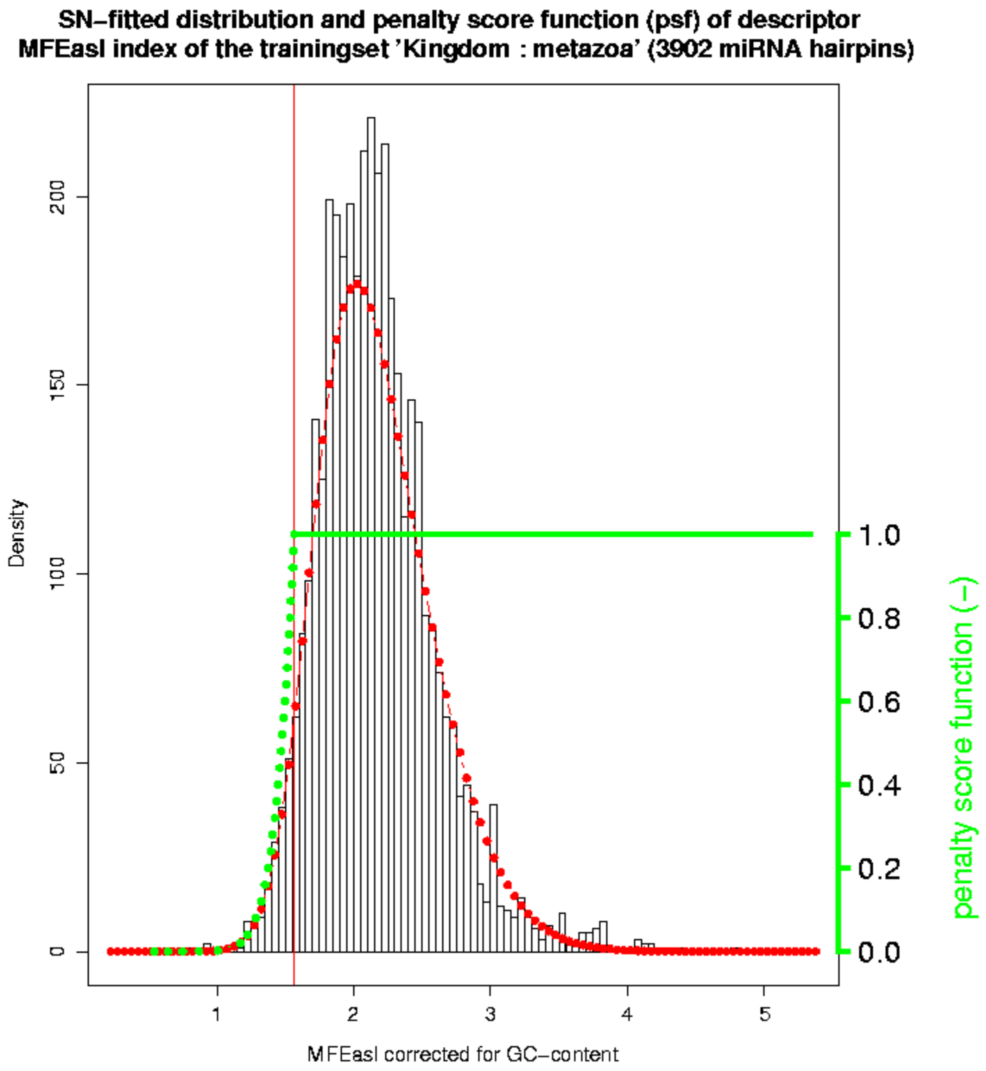

QQ-plot

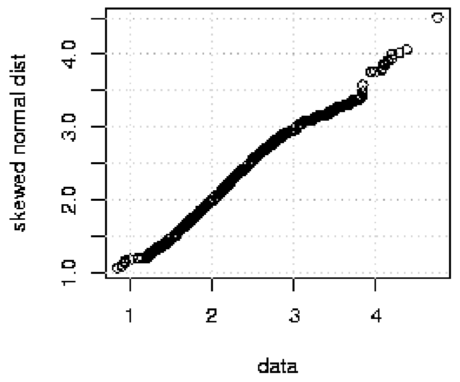

**Chisquare:**	0.0
**Location:**	1.70486
**Scale:**	0.630493
**Shape:**	2.57055
**(Normal) mean:**	2.16775
**Observed max:**	4.752
**Observed min:**	0.844444
**S<1 cut-off (95%):**	1.56589

MFEindex

SN–fitted distribution and penalty score function (psf) of descriptor MFEindex of the trainingset 'Kingdom : metazoa' (3902 miRNA hairpins)

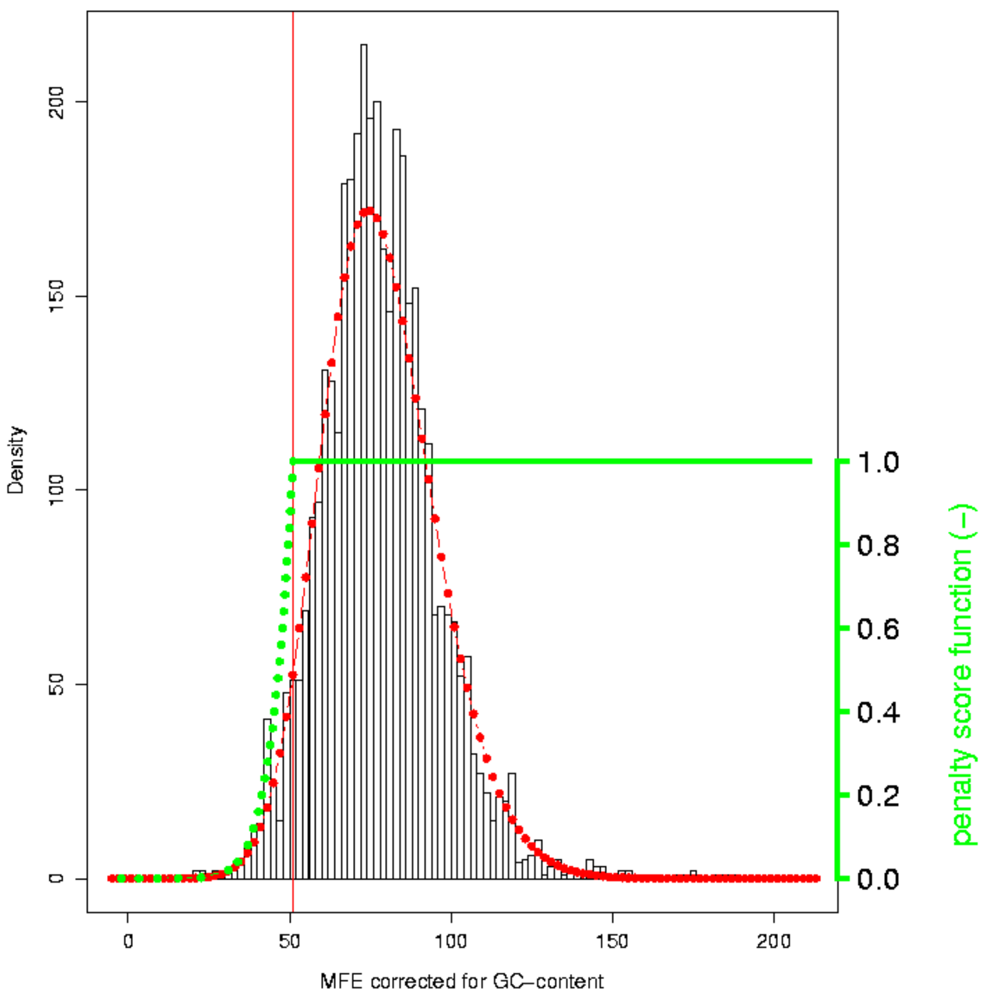

QQ-plot

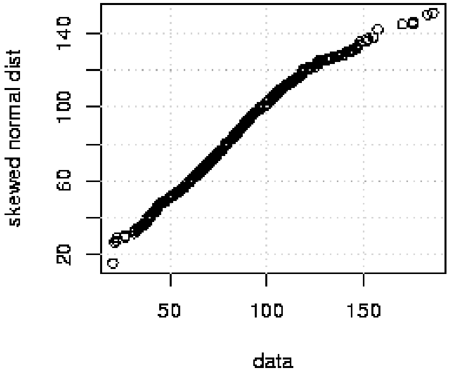

**Chisquare:**	0.0
**Location:**	61.1007
**Scale:**	24.6399
**Shape:**	1.74921
**(Normal) mean:**	78.0512
**Observed max:**	186.171
**Observed min:**	20.5615
**S<1 cut-off (95%):**	51.0978

GU-match contribution

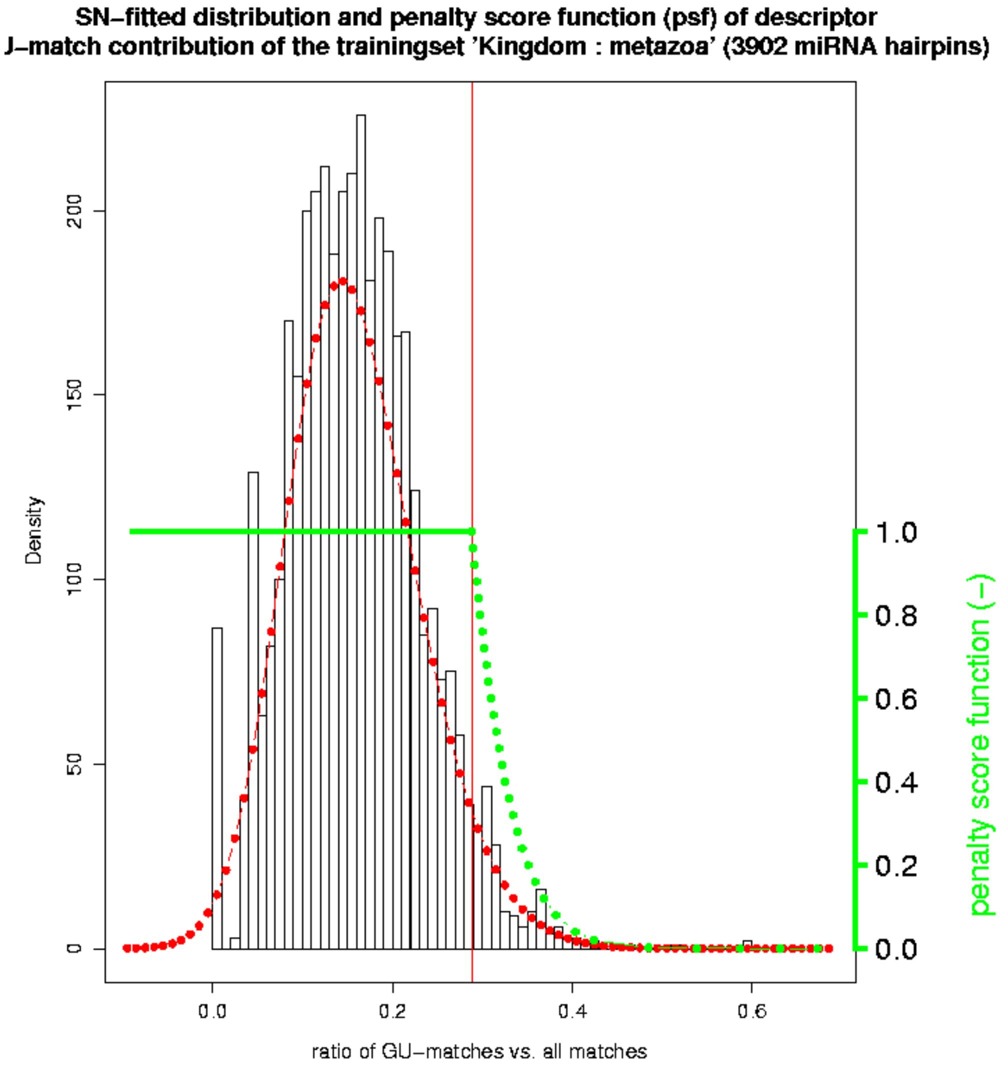

QQ-plot

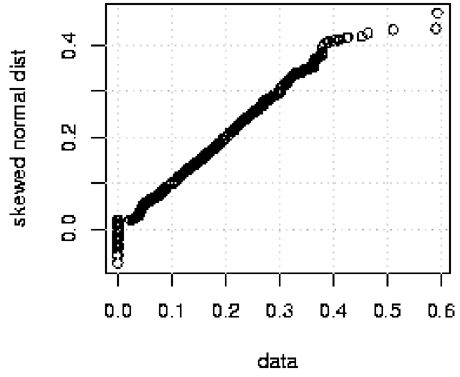

**Chisquare:**	0.0
**Location:**	0.0883499
**Scale:**	0.101917
**Shape:**	1.71873
**(Normal) mean:**	0.158559
**Observed max:**	0.594609
**Observed min:**	0.0
**S<1 cut-off (95%):**	0.288099

MFE

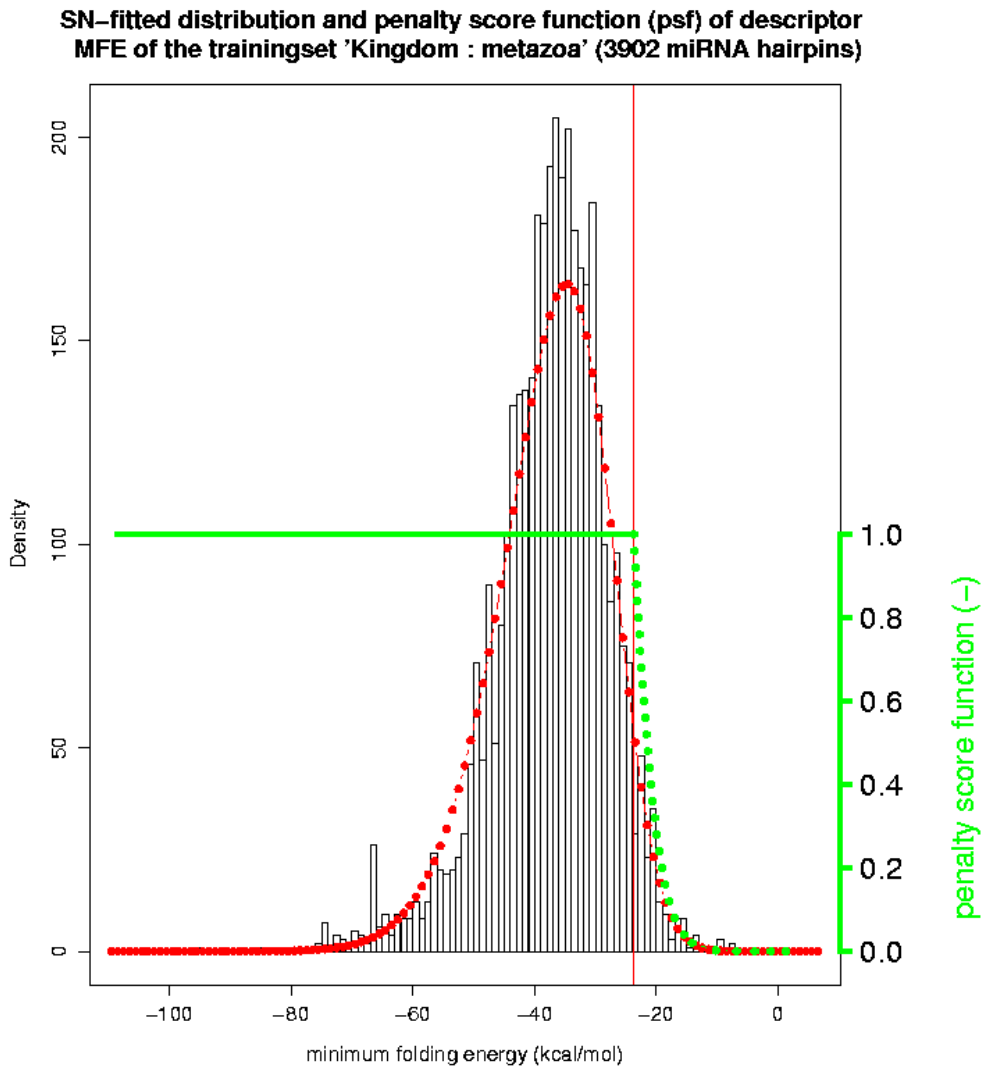

QQ-plot

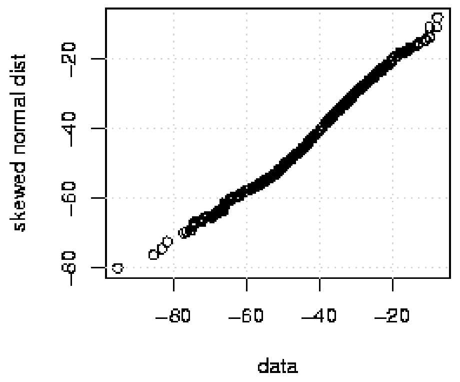

**Chisquare:**	0.0
**Location:**	-27.7019
**Scale:**	13.4971
**Shape:**	-2.16693
**(Normal) mean:**	-37.3639
**Observed max:**	-7.6
**Observed min:**	-95.4
**S<1 cut-off (95%):**	-23.7178

MFEahl

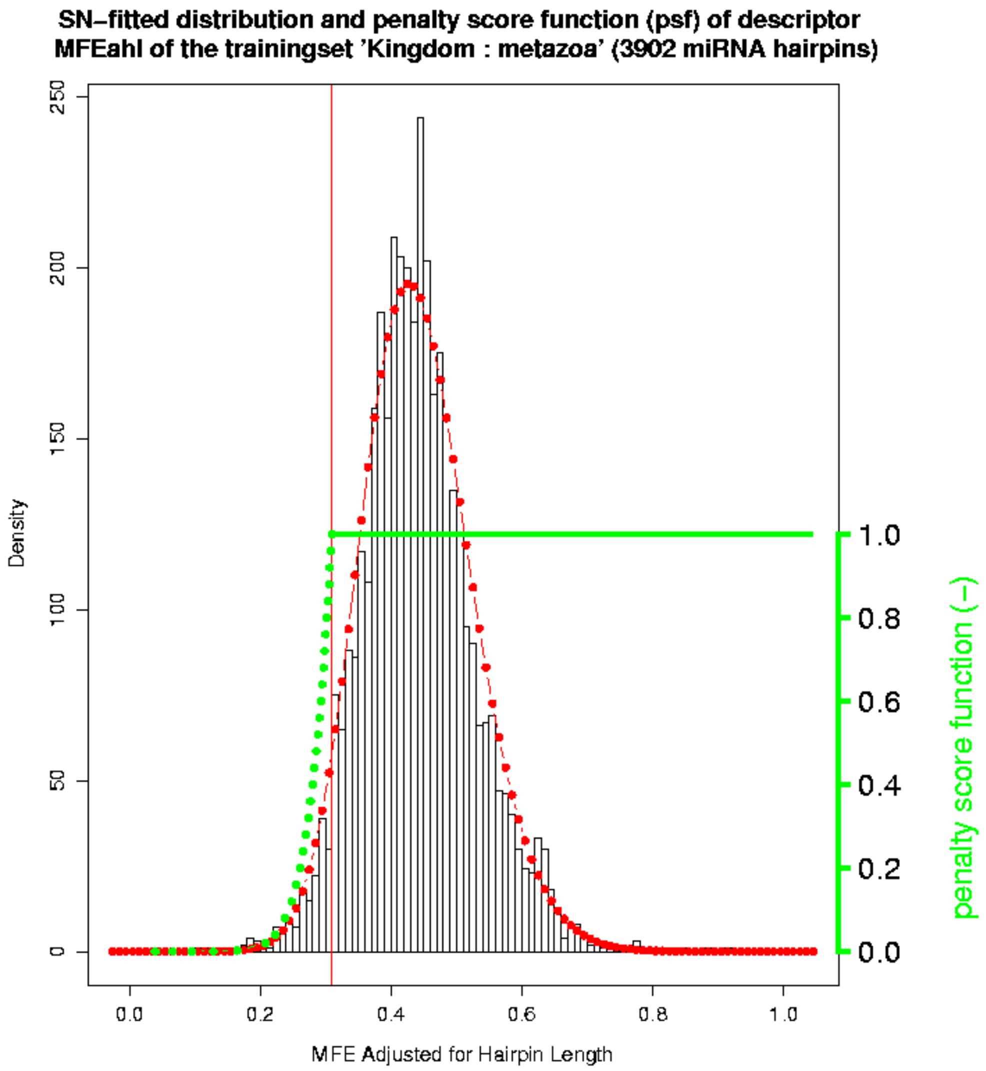

QQ-plot

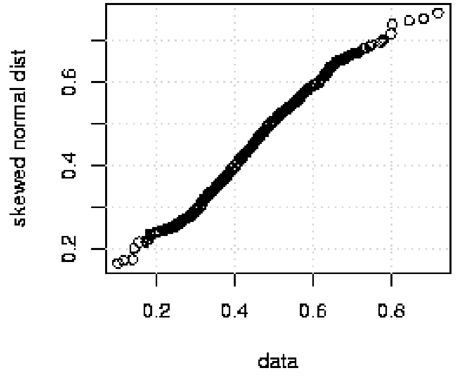

**Chisquare:**	5.54375e-10
**Location:**	0.365177
**Scale:**	0.11512
**Shape:**	1.53494
**(Normal) mean:**	0.441833
**Observed max:**	0.918519
**Observed min:**	0.101282
**S<1 cut-off (95%):**	0.309681

MFEasl

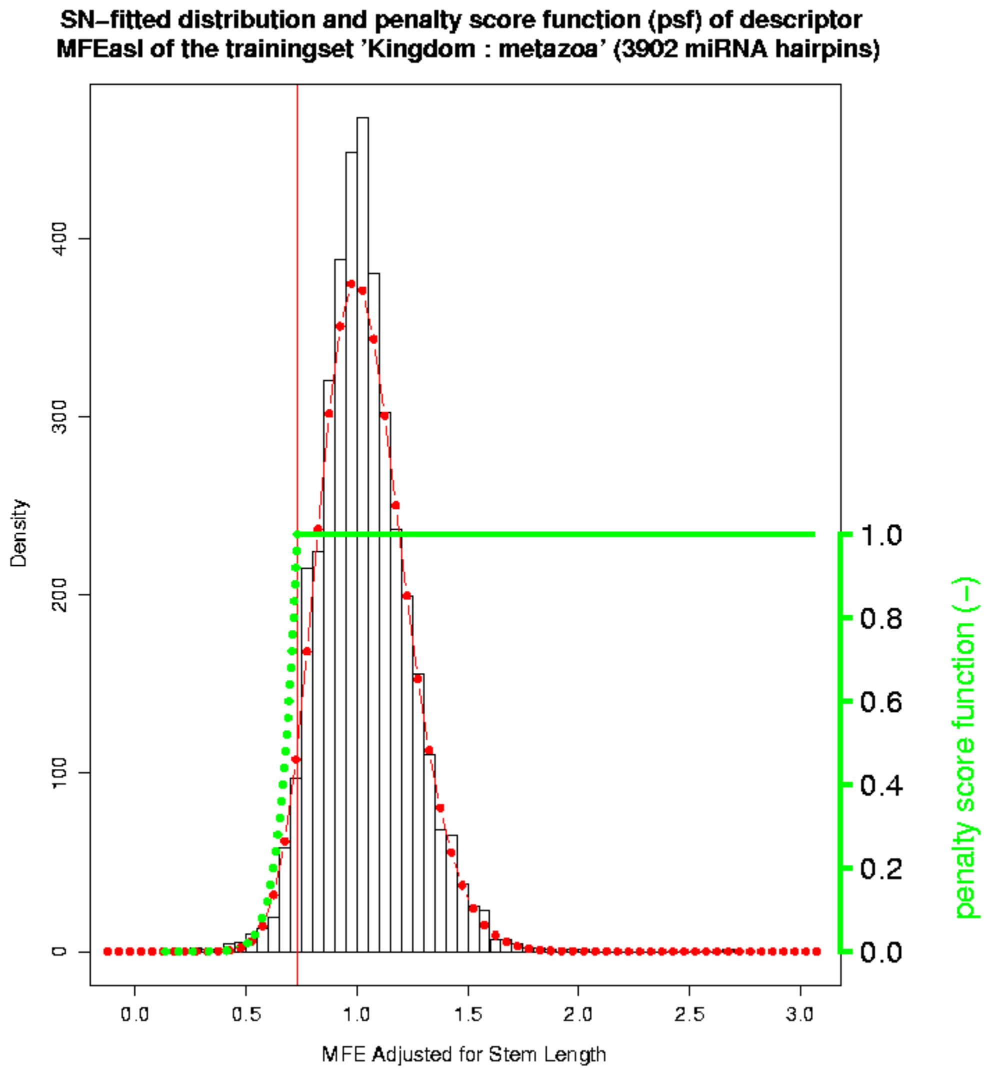

QQ-plot

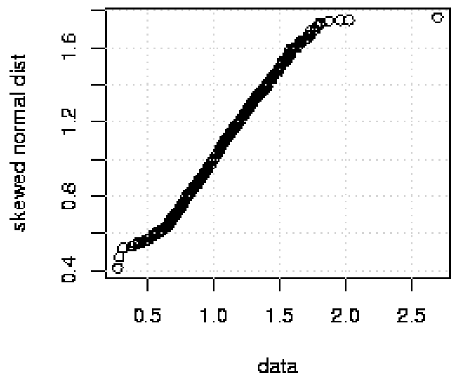

**Chisquare:**	5.04619e-12
**Location:**	0.845142
**Scale:**	0.273289
**Shape:**	1.72176
**(Normal) mean:**	1.03273
**Observed max:**	2.69286
**Observed min:**	0.281481
**S<1 cut-off (95%):**	0.731766

Z

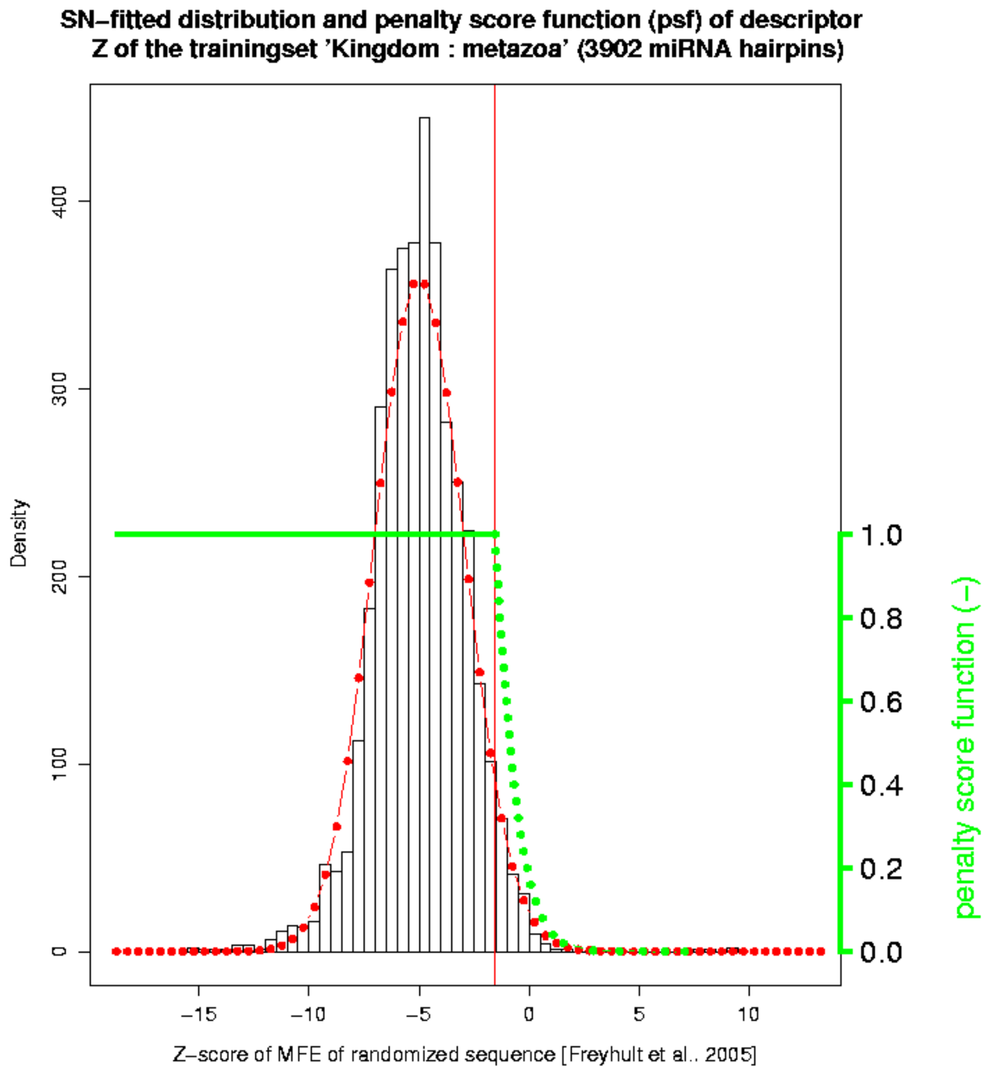

QQ-plot

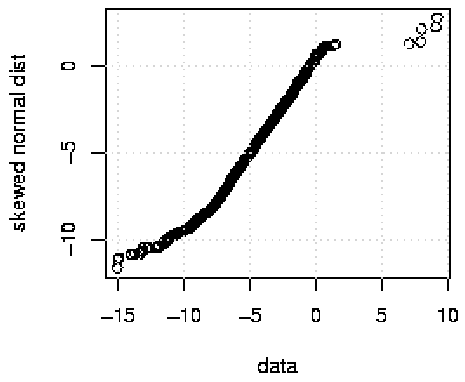

**Chisquare:**	0.0
**Location:**	-5.89667
**Scale:**	2.26638
**Shape:**	0.599787
**(Normal) mean:**	-4.9672
**Observed max:**	9.235
**Observed min:**	-15.068
**S<1 cut-off (95%):**	-1.54438

GAsurplusCU

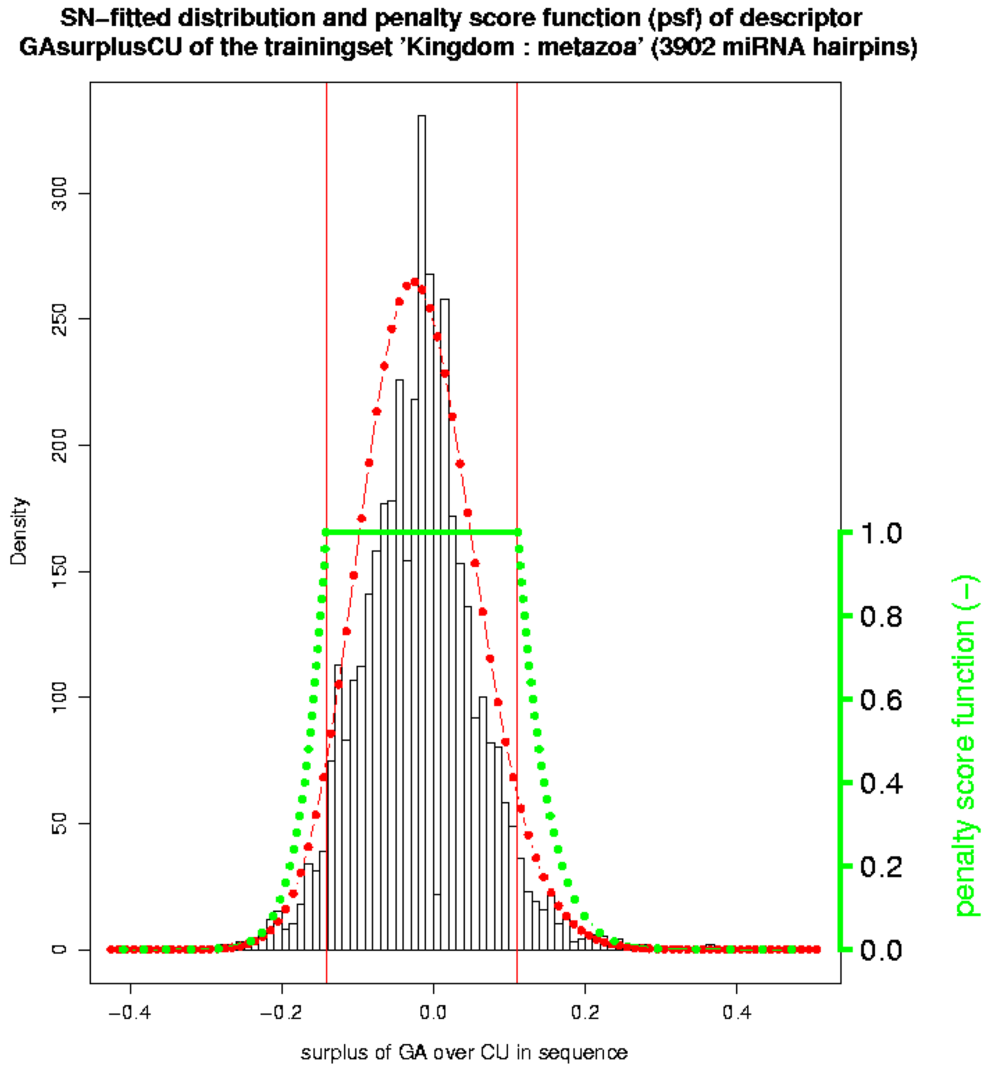

QQ-plot

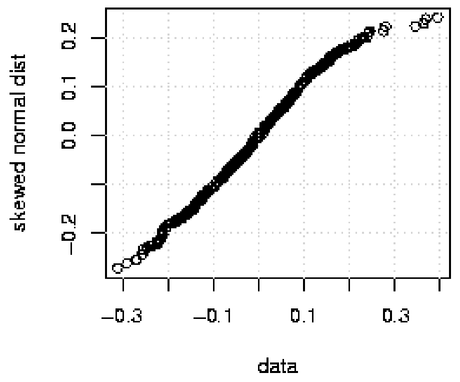

**Chisquare:**	0.0
**Location:**	-0.0767883
**Scale:**	0.095809
**Shape:**	1.13371
**(Normal) mean:**	-0.0195647
**Observed max:**	0.3939
**Observed min:**	-0.3111
**S<1 cut-off (95%):**	0.110752
**S<1 cut-off (95%):**	-0.141576

GC-content

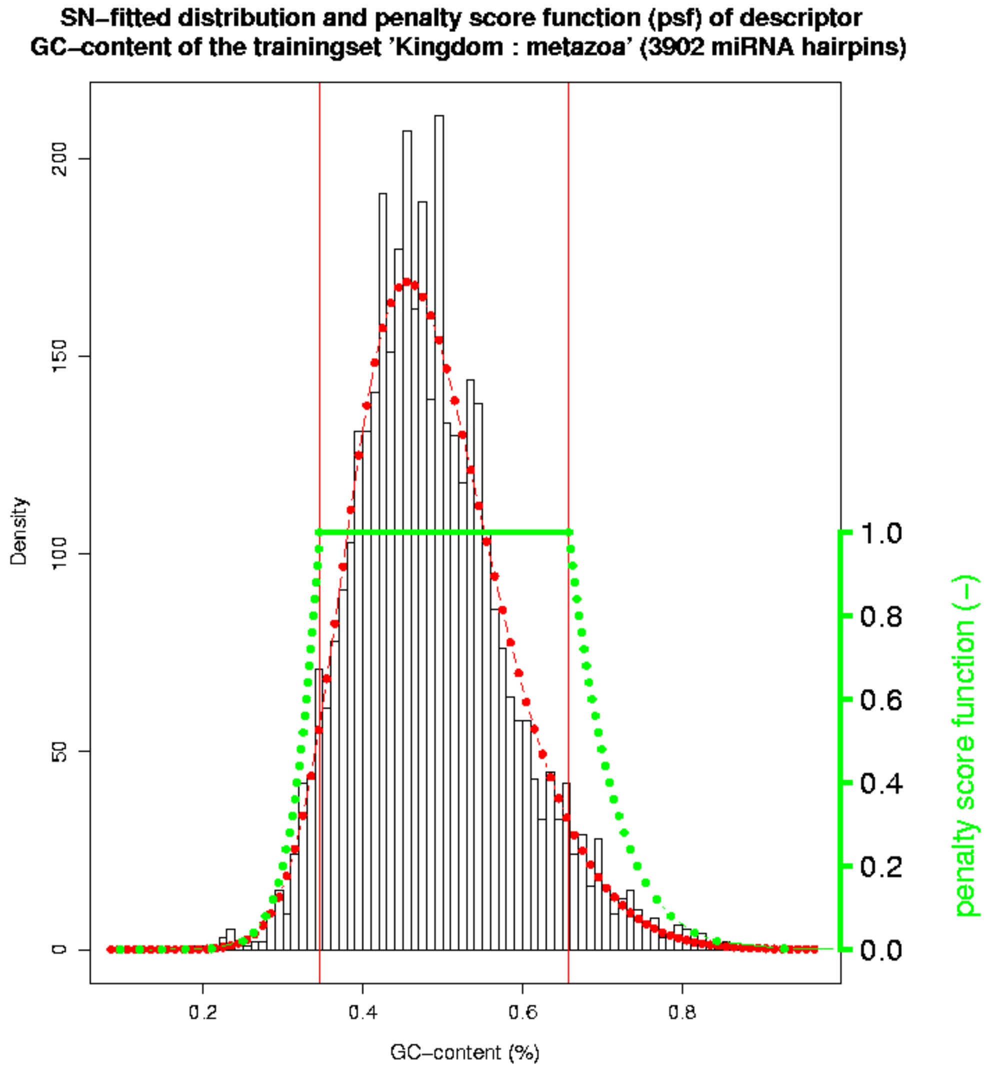

QQ-plot

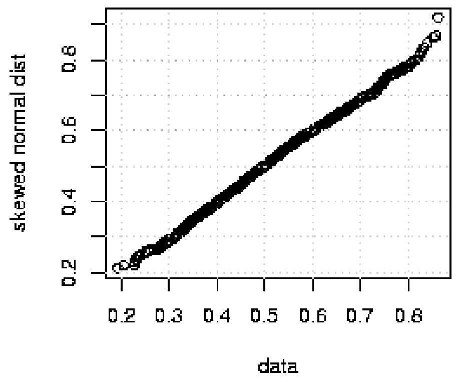

**Chisquare:**	1.38619e-05
**Location:**	0.383866
**Scale:**	0.13968
**Shape:**	2.27646
**(Normal) mean:**	0.485548
**Observed max:**	0.8625
**Observed min:**	0.1932
**S<1 cut-off (95%):**	0.657634
**S<1 cut-off (95%):**	0.345824

GCratio

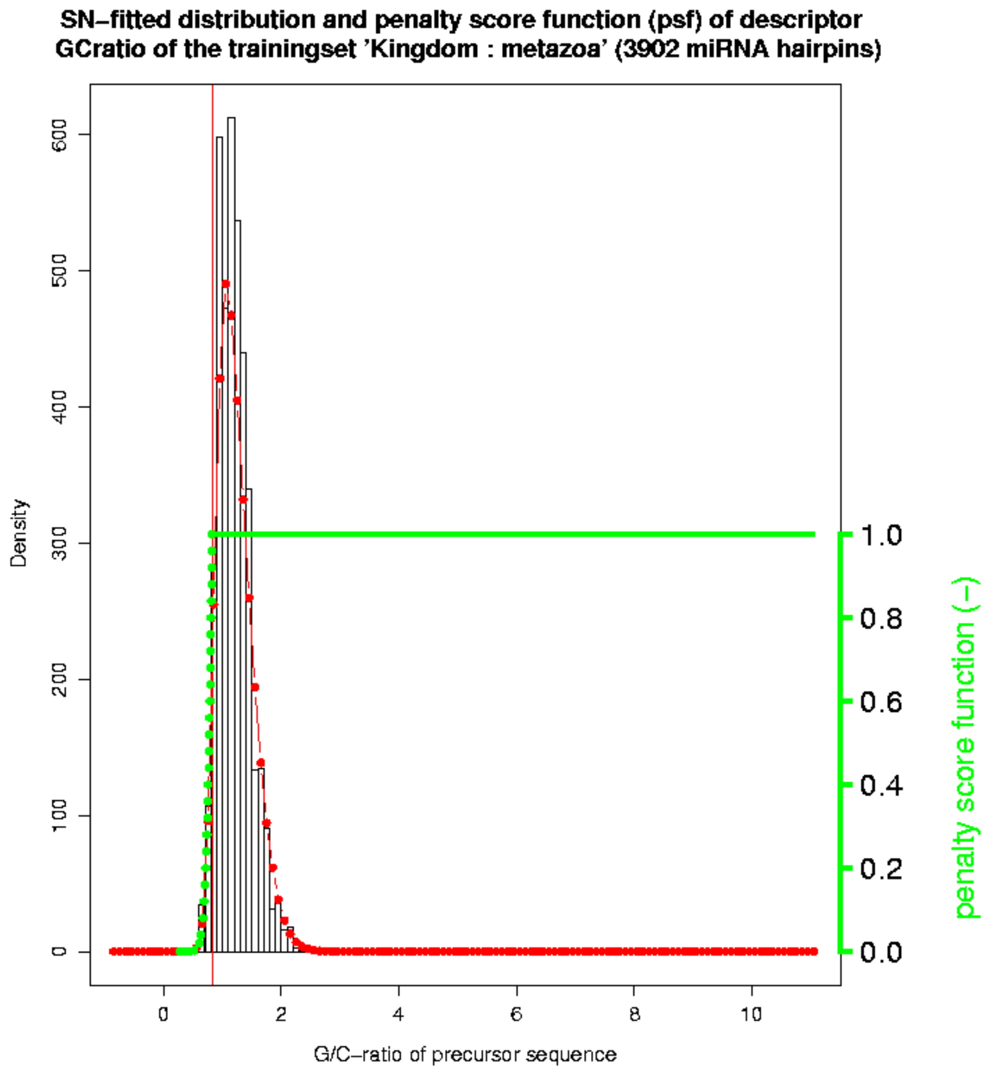

QQ-plot

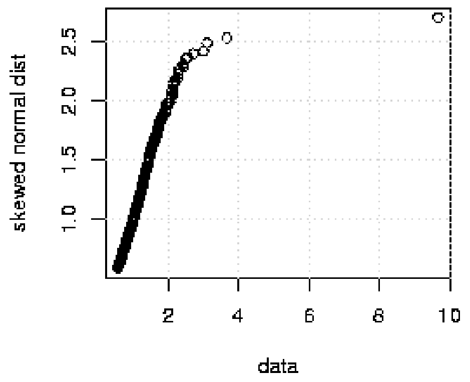

**Chisquare:**	7.66054e-15
**Location:**	0.865937
**Scale:**	0.466545
**Shape:**	3.78372
**(Normal) mean:**	1.21302
**Observed max:**	9.6667
**Observed min:**	0.5745
**S<1 cut-off (95%):**	0.821993

GsurplusC

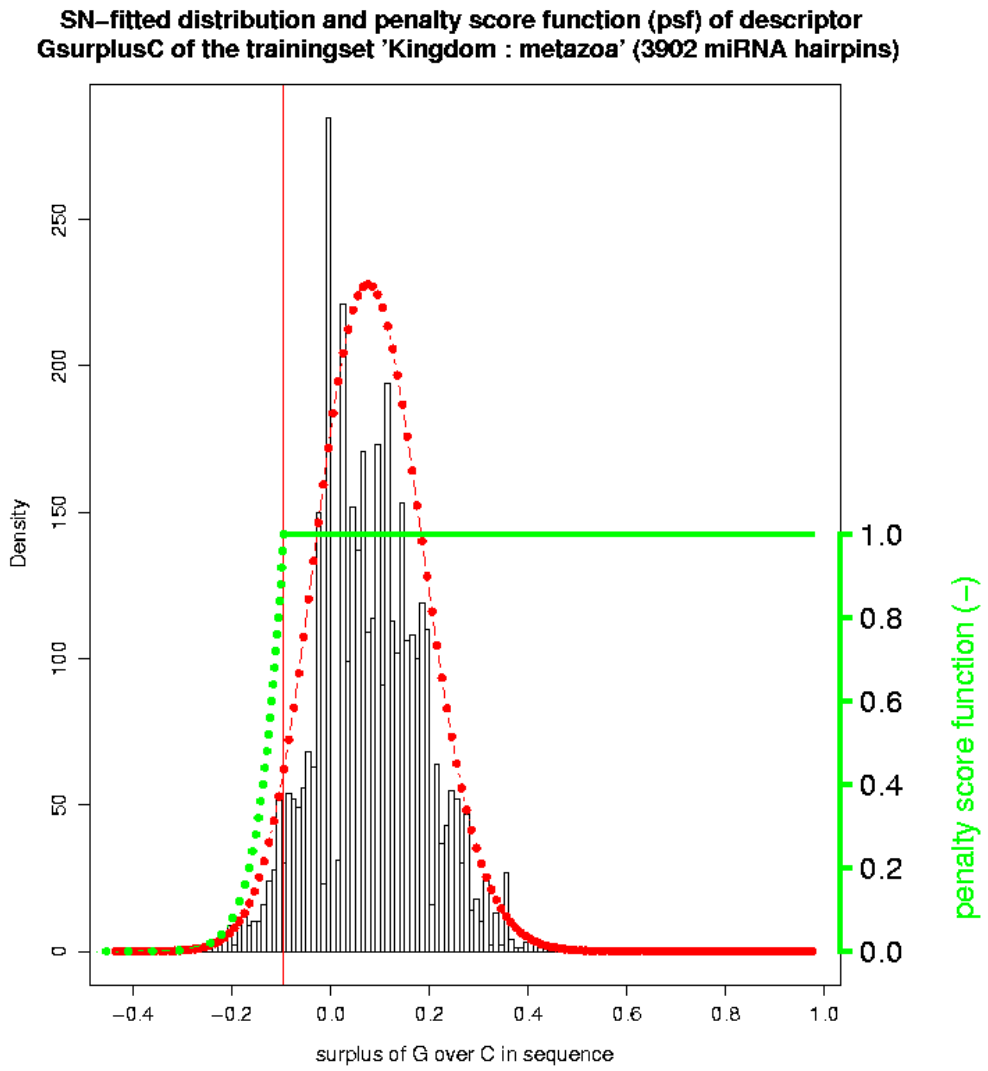

QQ-plot

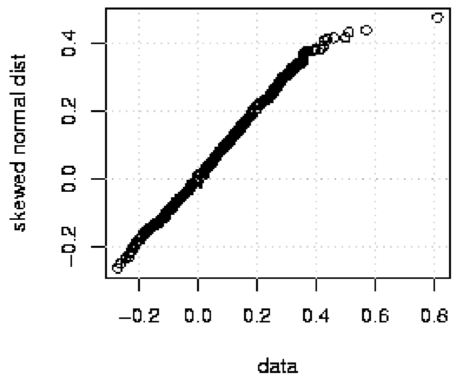

**Chisquare:**	5.58931e-08
**Location:**	0.0108803
**Scale:**	0.130924
**Shape:**	0.928368
**(Normal) mean:**	0.0819377
**Observed max:**	0.8125
**Observed min:**	-0.2703
**S<1 cut-off (95%):**	-0.095113

MaxDiBaseRatio

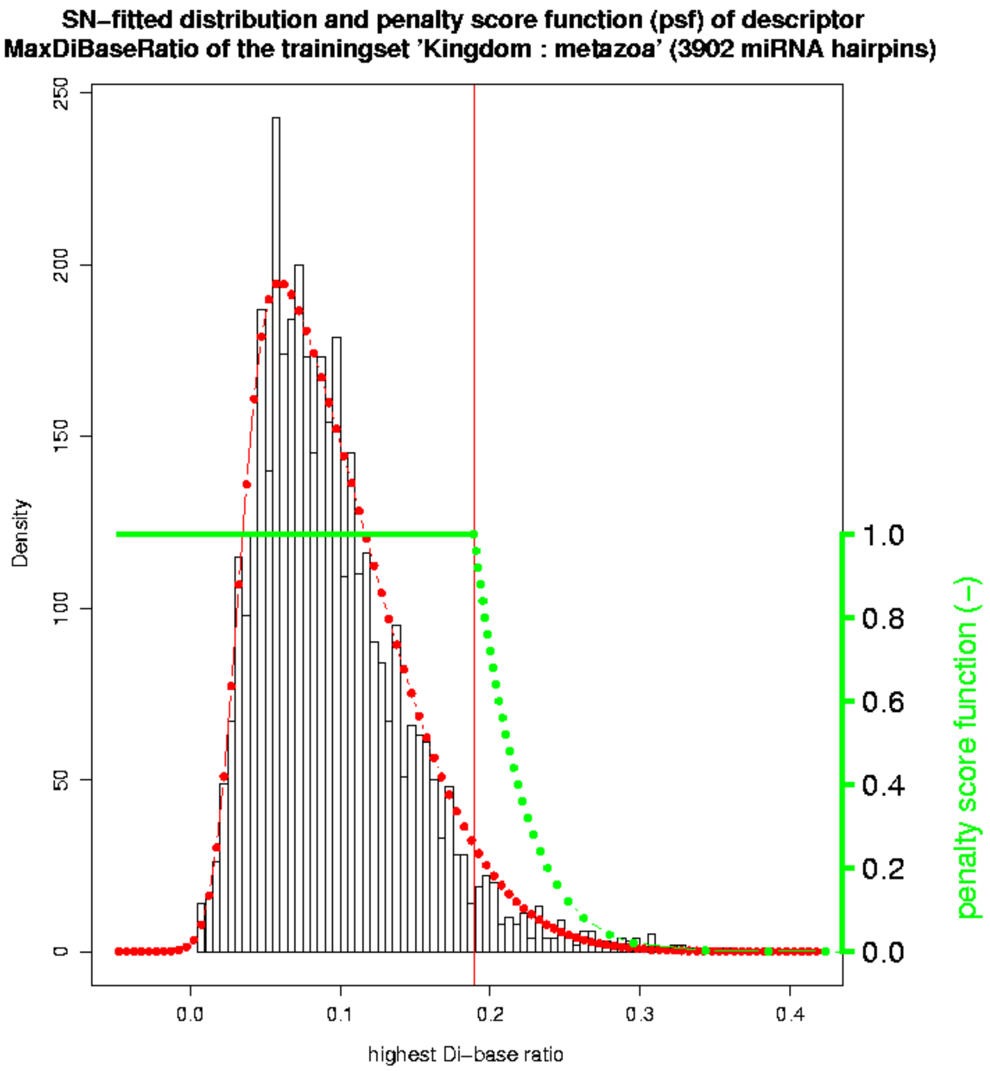

QQ-plot

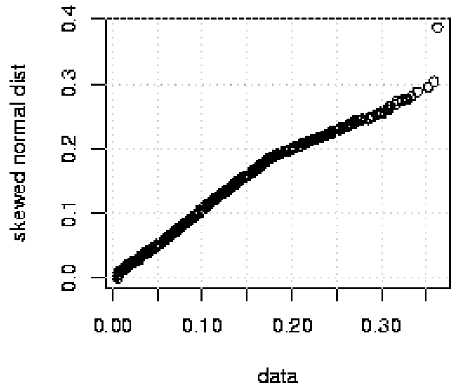

**Chisquare:**	0.0
**Location:**	0.0323537
**Scale:**	0.0799498
**Shape:**	5.68686
**(Normal) mean:**	0.0942557
**Observed max:**	0.3625
**Observed min:**	0.006
**S<1 cut-off (95%):**	0.189052

minimal base occurence

SN–fitted distribution and penalty score function (psf) of descriptor
[illegible]imal base occurence of the trainingset 'Kingdom : metazoa' (3902 miRNA hairpins)

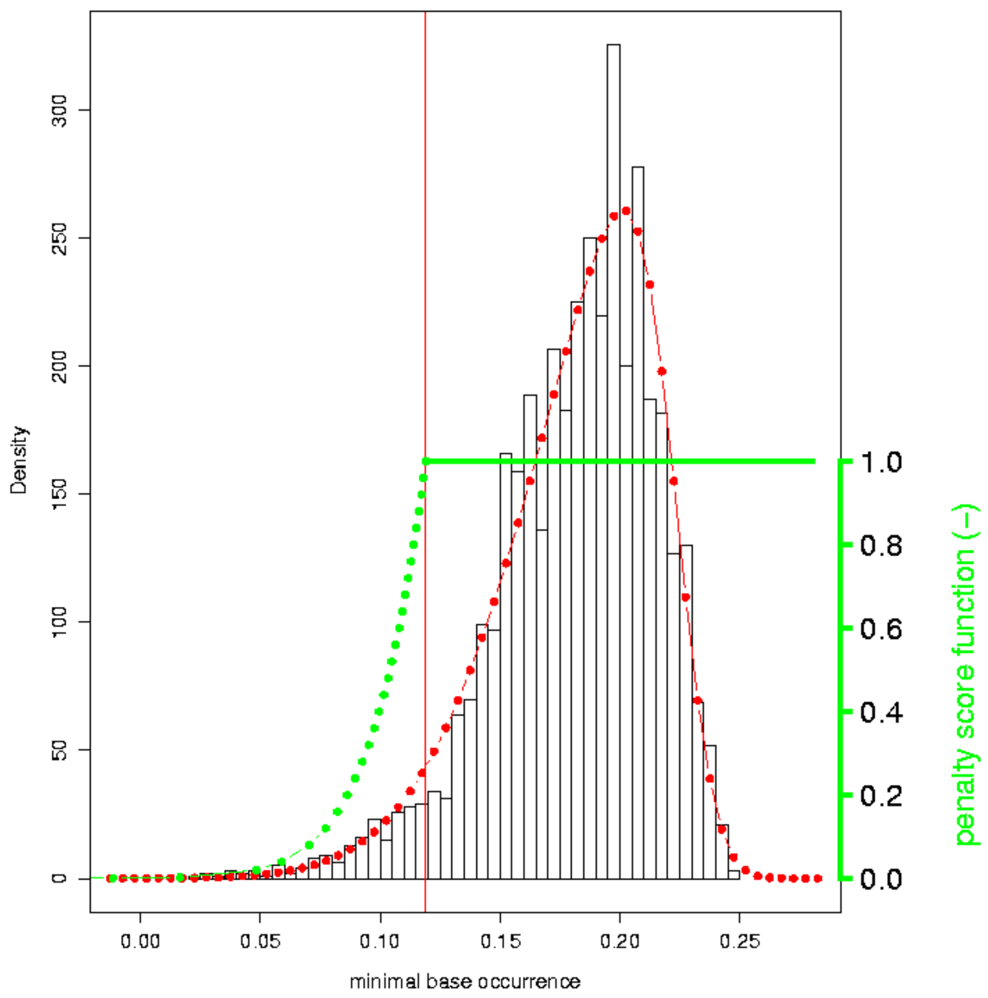

QQ-plot

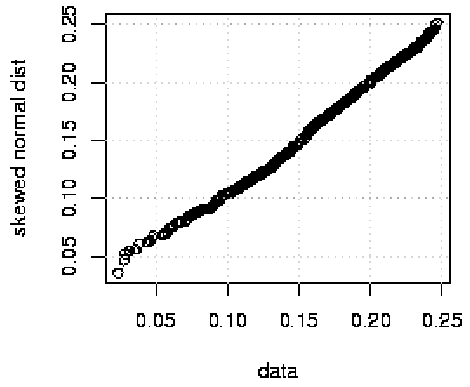

**Chisquare:**	5.26947e-09
**Location:**	0.223177
**Scale:**	0.0531415
**Shape:**	-4.08112
**(Normal) mean:**	0.182043
**Observed max:**	0.2469
**Observed min:**	0.0238
**S<1 cut-off (95%):**	0.119022

polyA

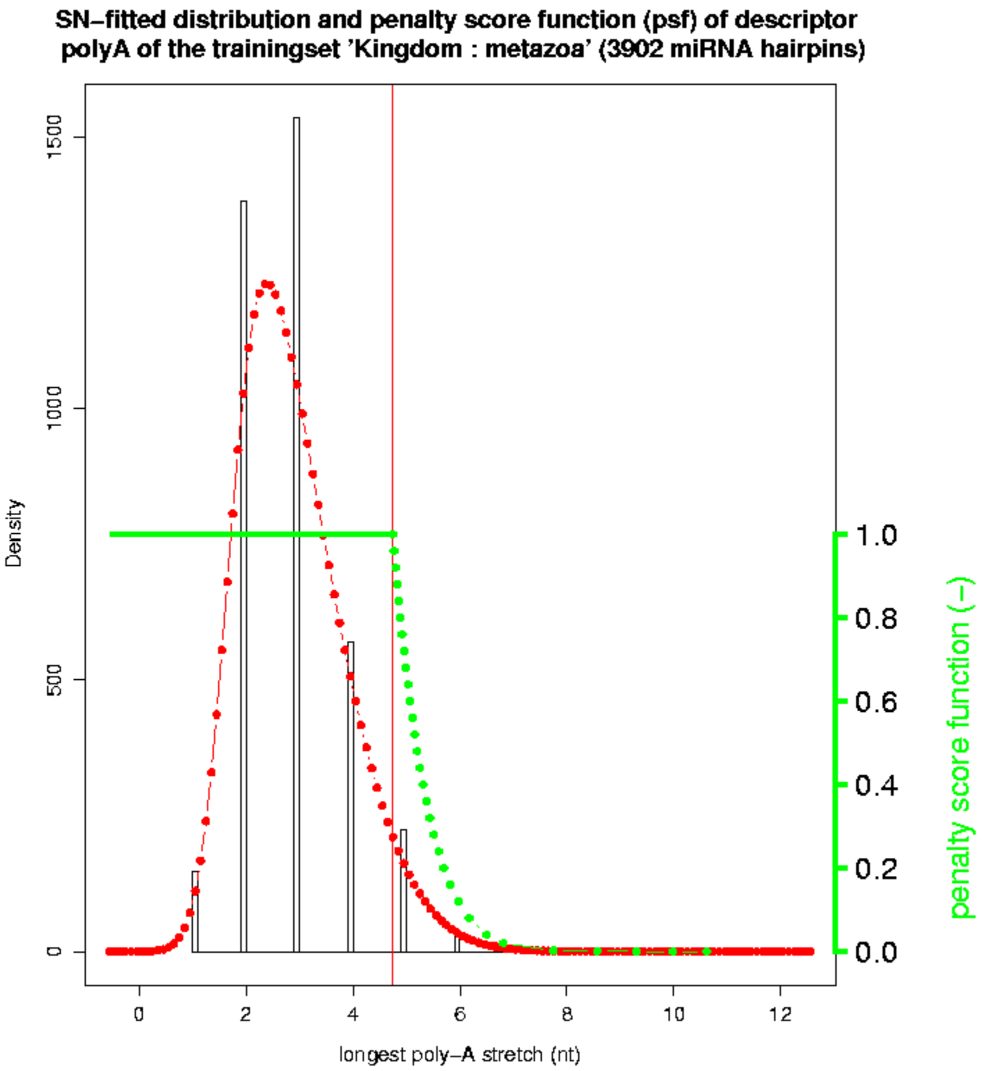

QQ-plot

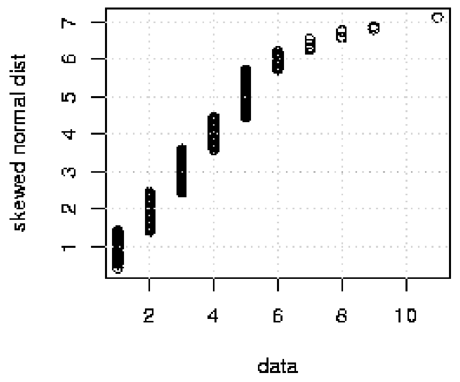

**Chisquare:**	0.0
**Location:**	1.68659
**Scale:**	1.55805
**Shape:**	3.38658
**(Normal) mean:**	2.8716
**Observed max:**	11.0
**Observed min:**	1.0
**S<1 cut-off (95%):**	4.7403

polyAstem

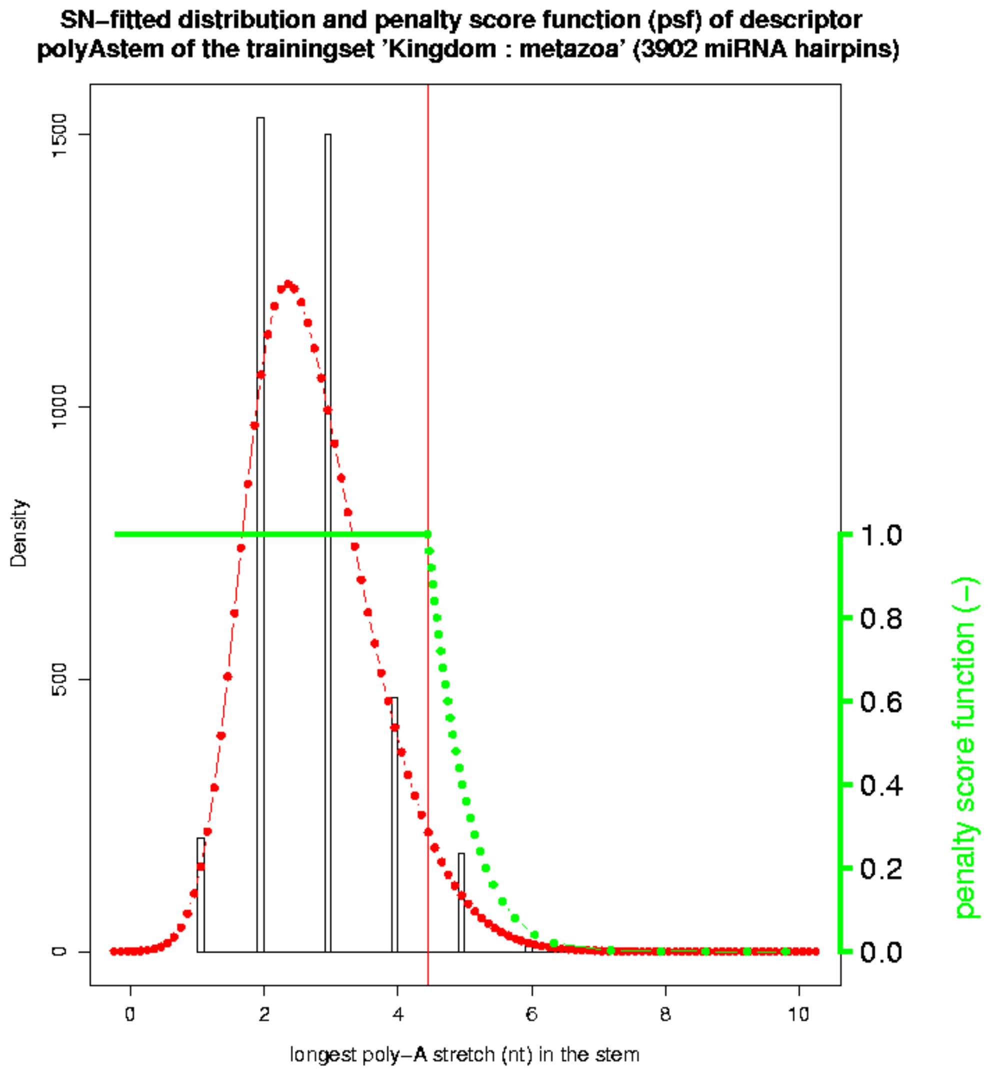

QQ-plot

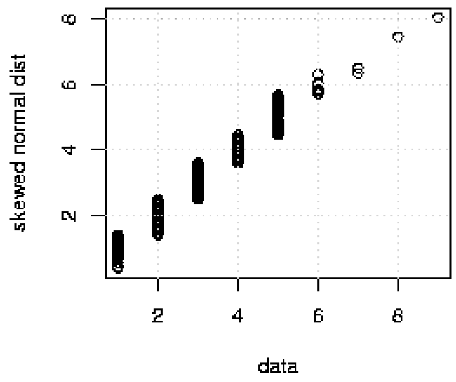

**Chisquare:**	0.0
**Location:**	1.66128
**Scale:**	1.41939
**Shape:**	2.808
**(Normal) mean:**	2.72501
**Observed max:**	9.0
**Observed min:**	1.0
**S<1 cut-off (95%):**	4.44324

polyC

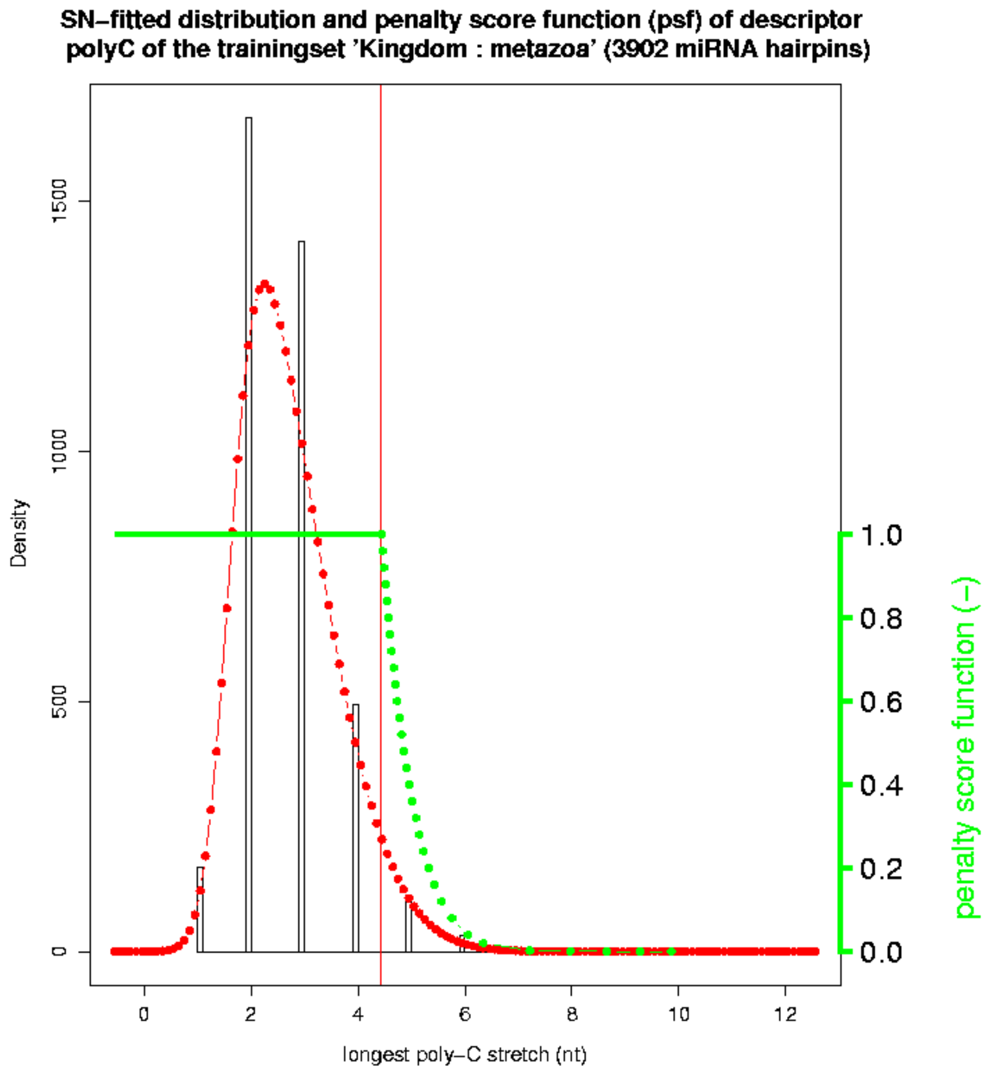

QQ-plot

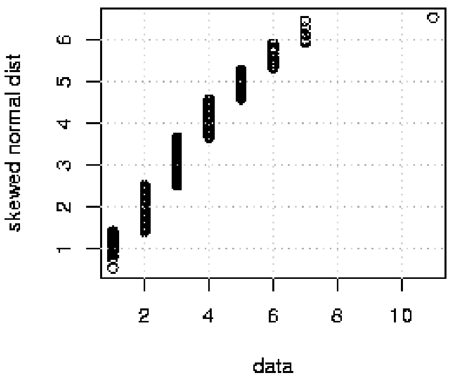

**Chisquare:**	0.0
**Location:**	1.61237
**Scale:**	1.43933
**Shape:**	3.53117
**(Normal) mean:**	2.70528
**Observed max:**	11.0
**Observed min:**	1.0
**S<1 cut-off (95%):**	4.4334

polyCstem

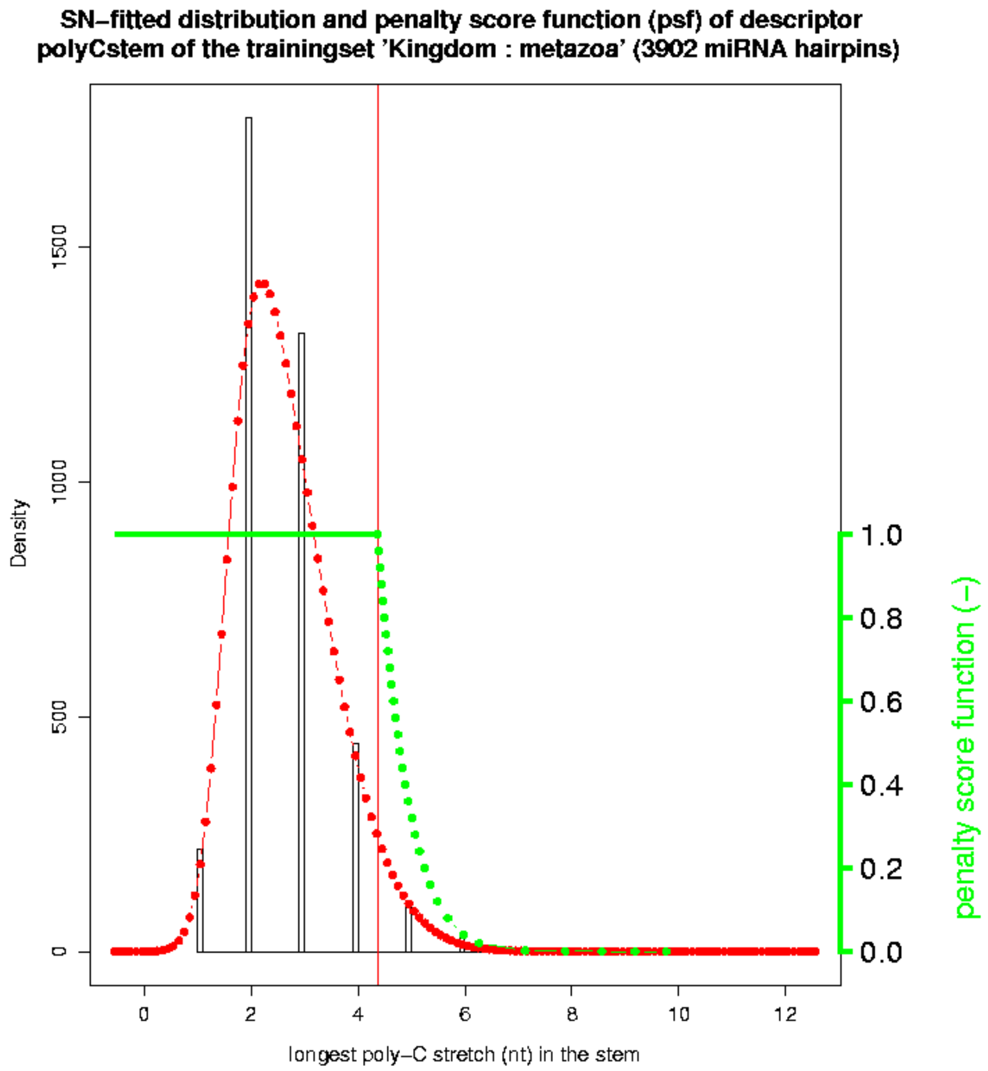

QQ-plot

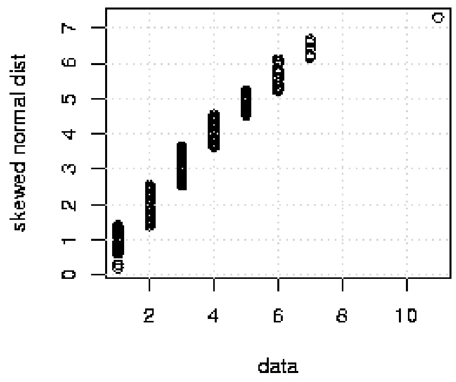

**Chisquare:**	0.0
**Location:**	1.55511
**Scale:**	1.43219
**Shape:**	3.3689
**(Normal) mean:**	2.63378
**Observed max:**	11.0
**Observed min:**	1.0
**S<1 cut-off (95%):**	4.36216

polyG

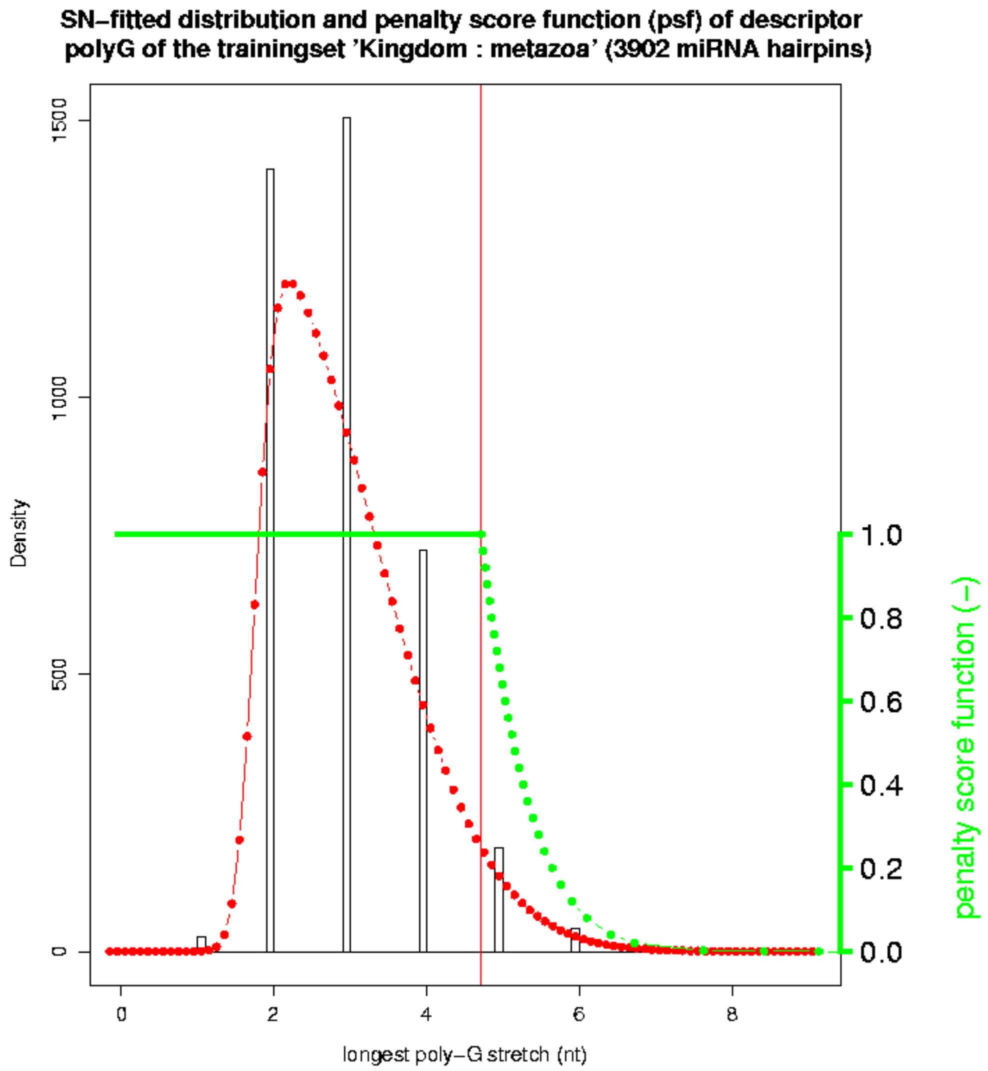

QQ-plot

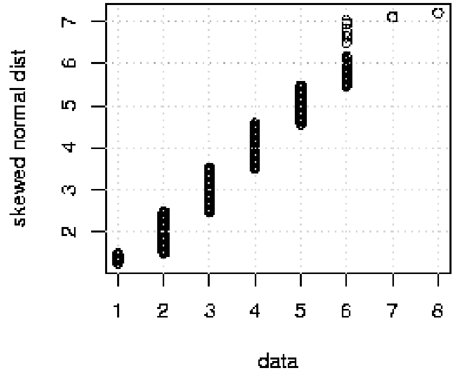

**Chisquare:**	0.0
**Location:**	1.75592
**Scale:**	1.50793
**Shape:**	7.32535
**(Normal) mean:**	2.94029
**Observed max:**	8.0
**Observed min:**	1.0
**S<1 cut-off (95%):**	4.71142

polyGstem

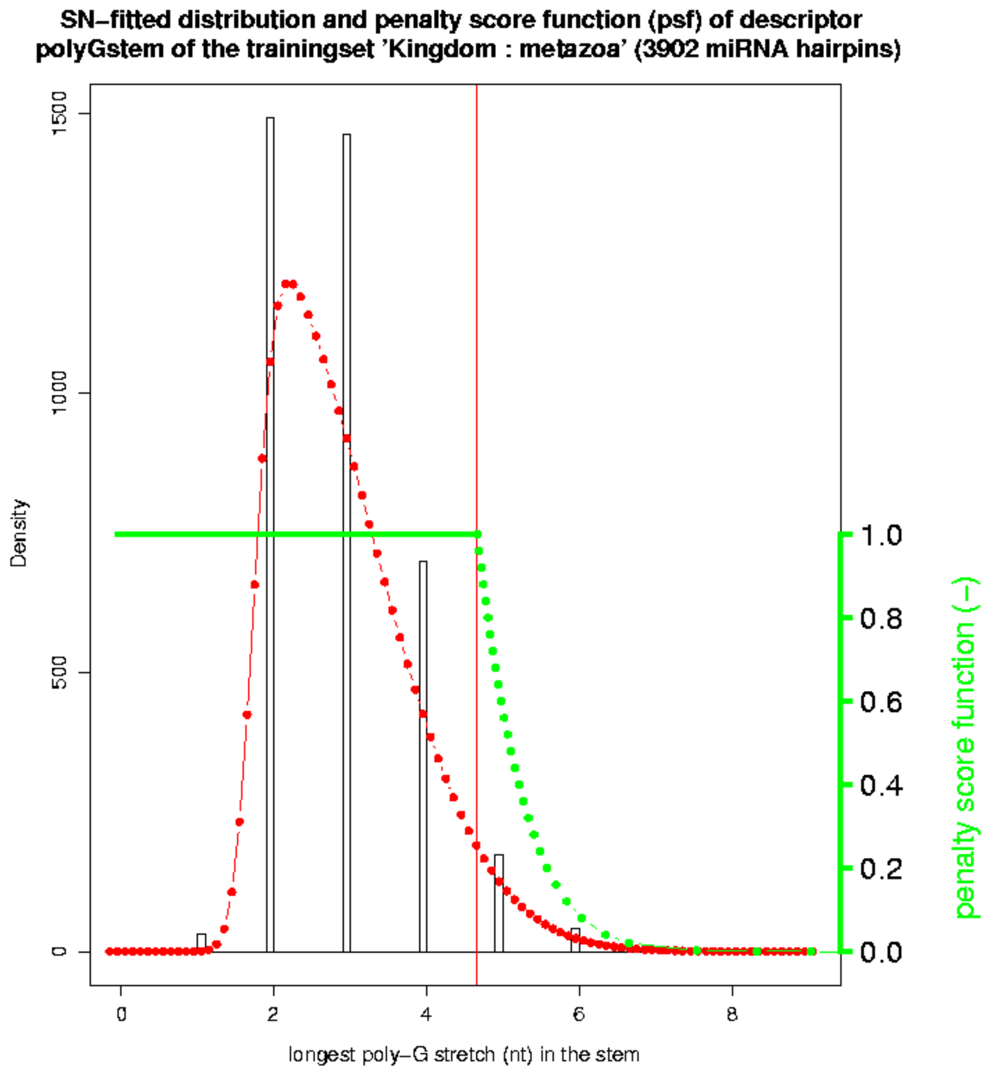

QQ-plot

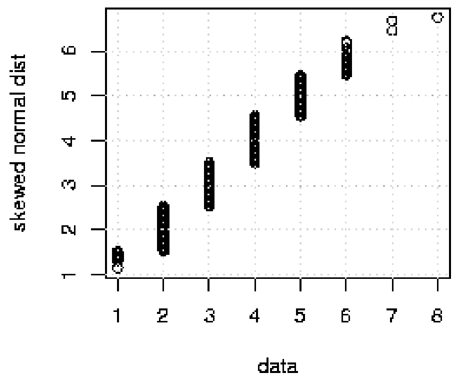

**Chisquare:**	0.0
**Location:**	1.74226
**Scale:**	1.48927
**Shape:**	6.98274
**(Normal) mean:**	2.9039
**Observed max:**	8.0
**Observed min:**	1.0
**S<1 cut-off (95%):**	4.66117

polyNucHairpin

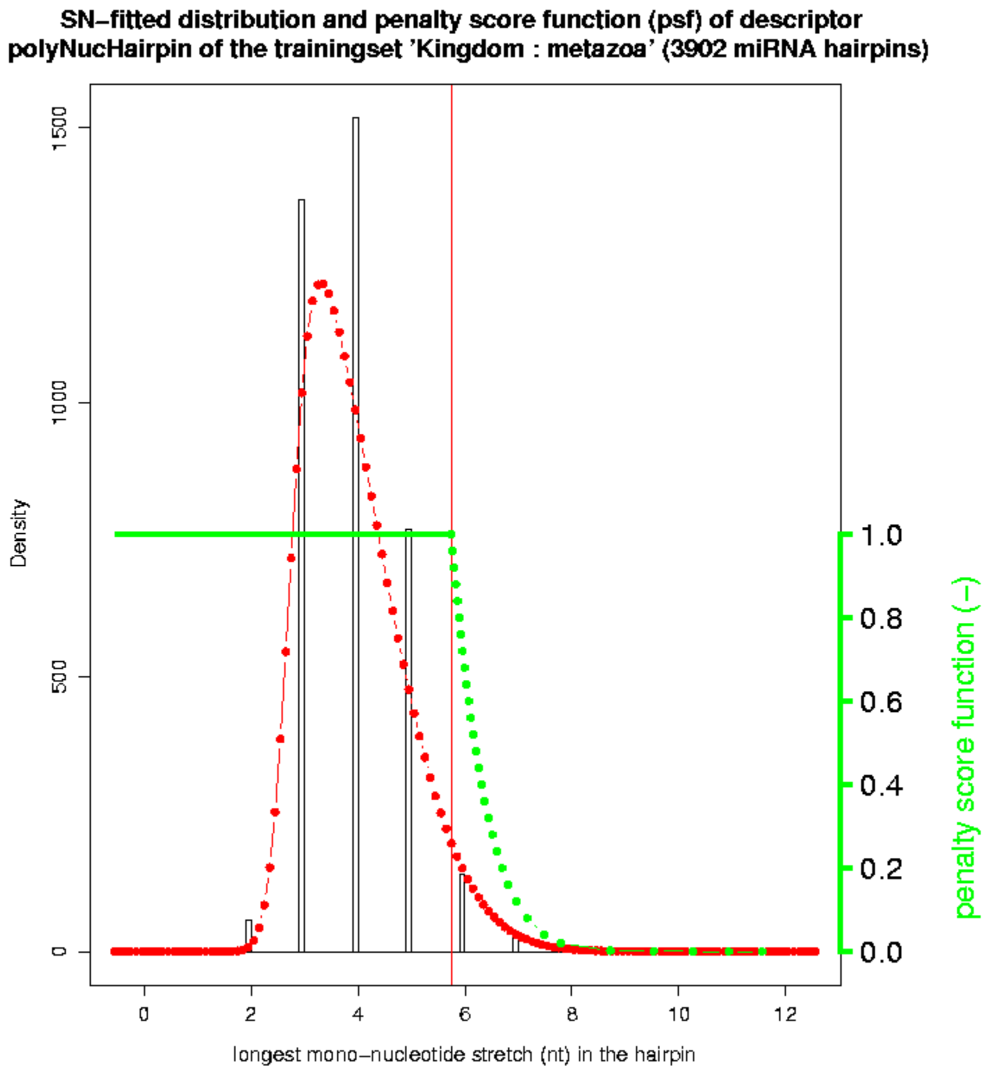

QQ-plot

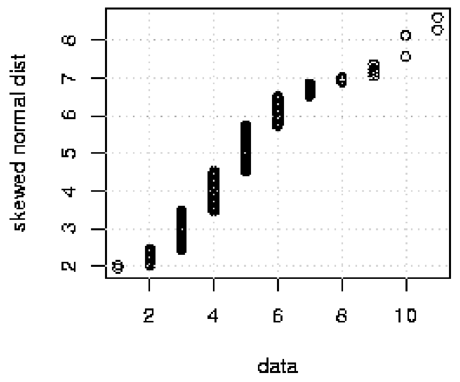

**Chisquare:**	0.0
**Location:**	2.72665
**Scale:**	1.5404
**Shape:**	4.87675
**(Normal) mean:**	3.93106
**Observed max:**	11.0
**Observed min:**	1.0
**S<1 cut-off (95%):**	5.74577

polyNucStem

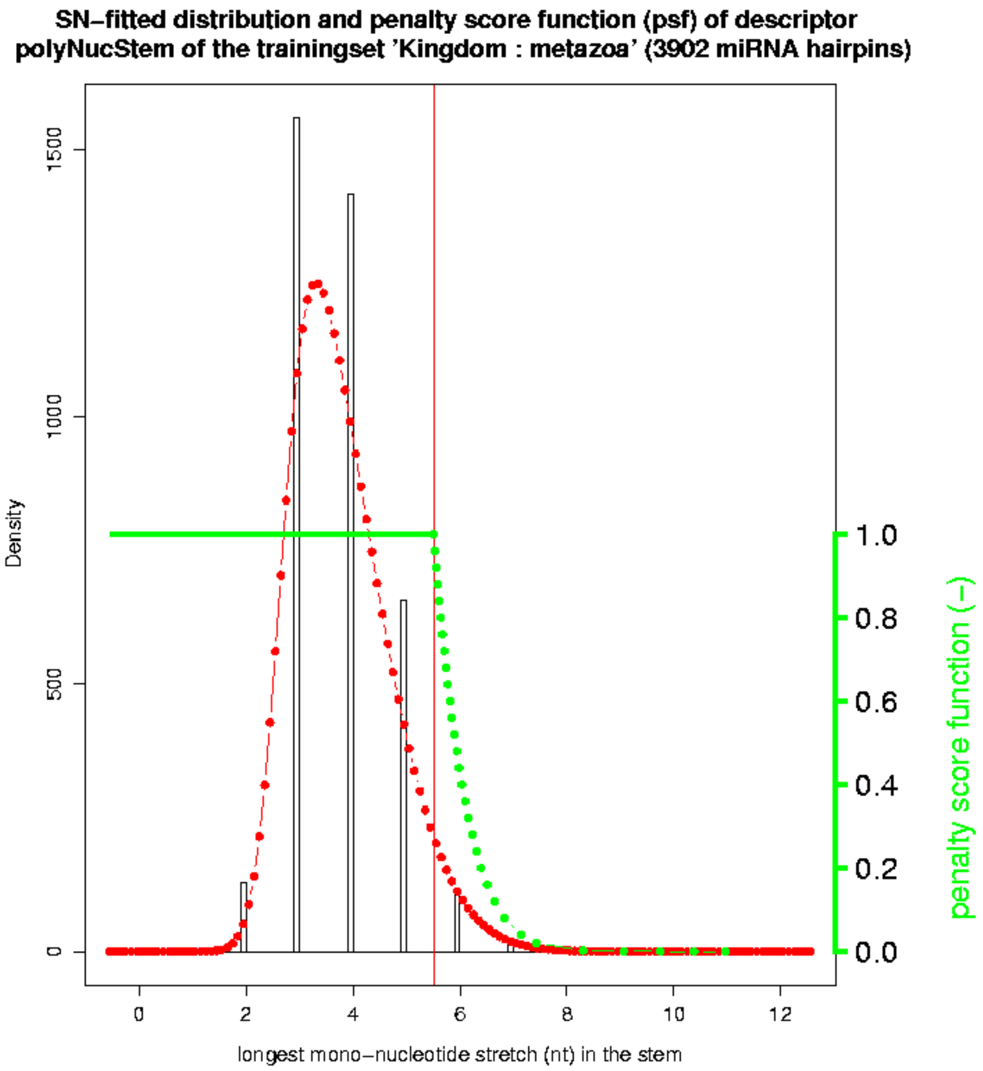

QQ-plot

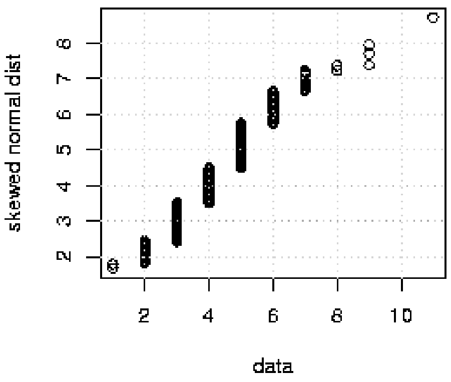

**Chisquare:**	0.0
**Location:**	2.67064
**Scale:**	1.44741
**Shape:**	3.52401
**(Normal) mean:**	3.78139
**Observed max:**	11.0
**Observed min:**	1.0
**S<1 cut-off (95%):**	5.50751

polyU

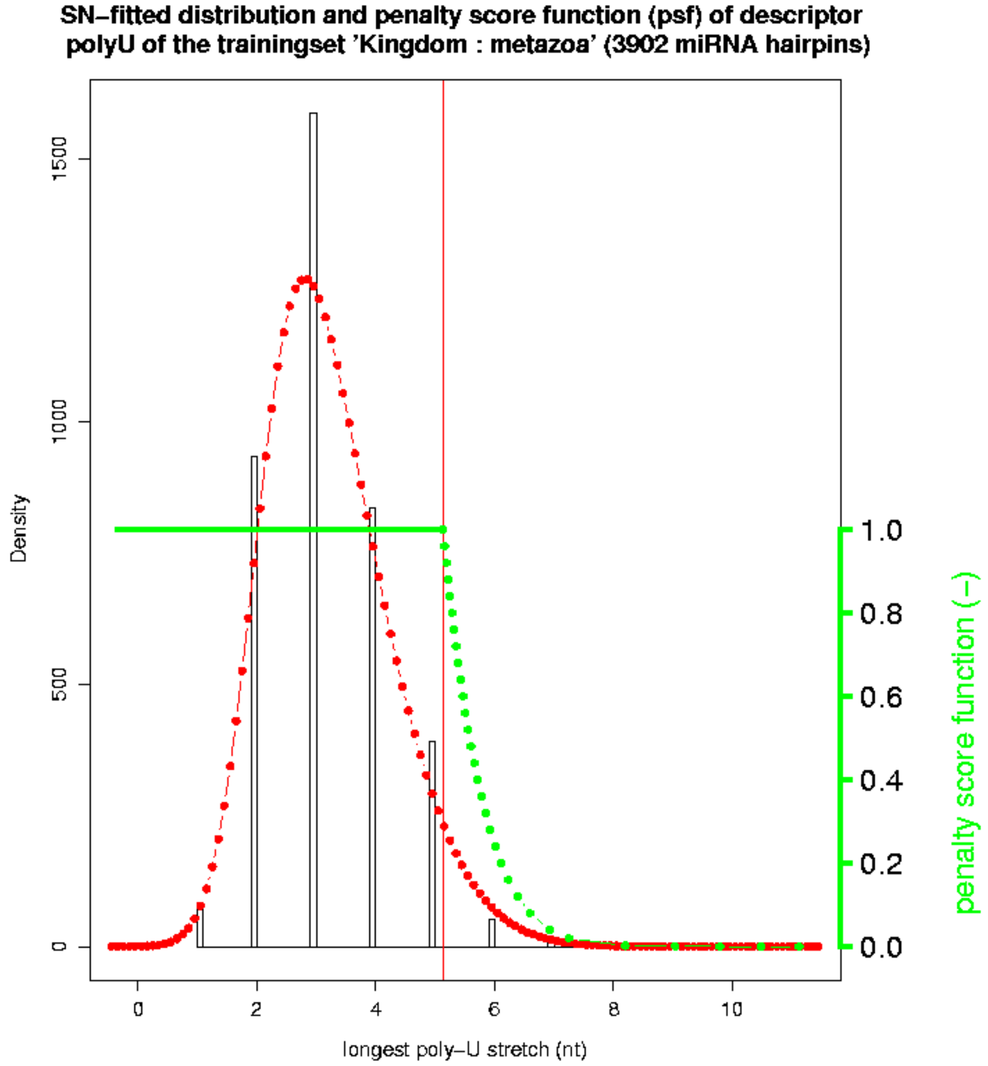

QQ-plot

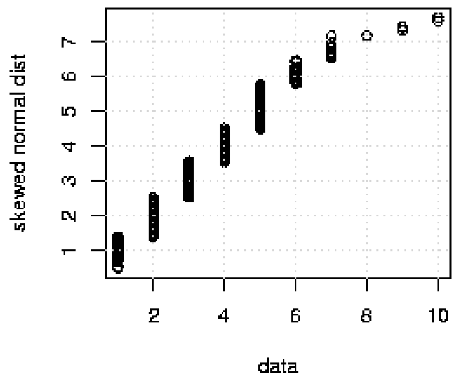

**Chisquare:**	0.0
**Location:**	2.0161
**Scale:**	1.58976
**Shape:**	2.58261
**(Normal) mean:**	3.19605
**Observed max:**	10.0
**Observed min:**	1.0
**S<1 cut-off (95%):**	5.13197

polyUstem

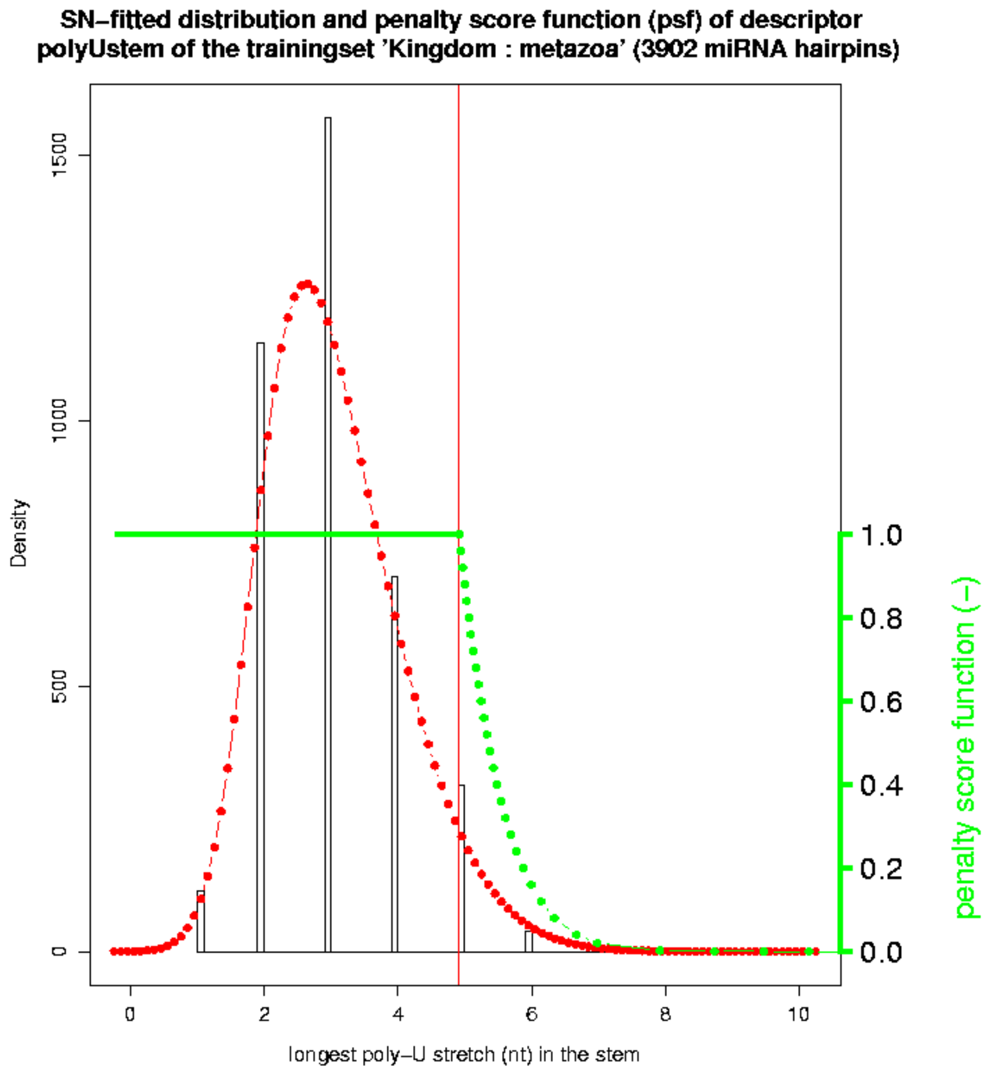

QQ-plot

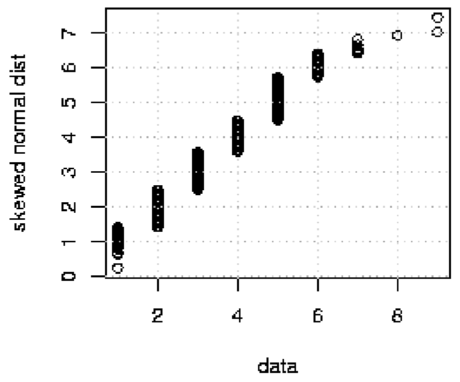

**Chisquare:**	0.0
**Location:**	1.86499
**Scale:**	1.55504
**Shape:**	2.77036
**(Normal) mean:**	3.03127
**Observed max:**	9.0
**Observed min:**	1.0
**S<1 cut-off (95%):**	4.91282

SCS-di

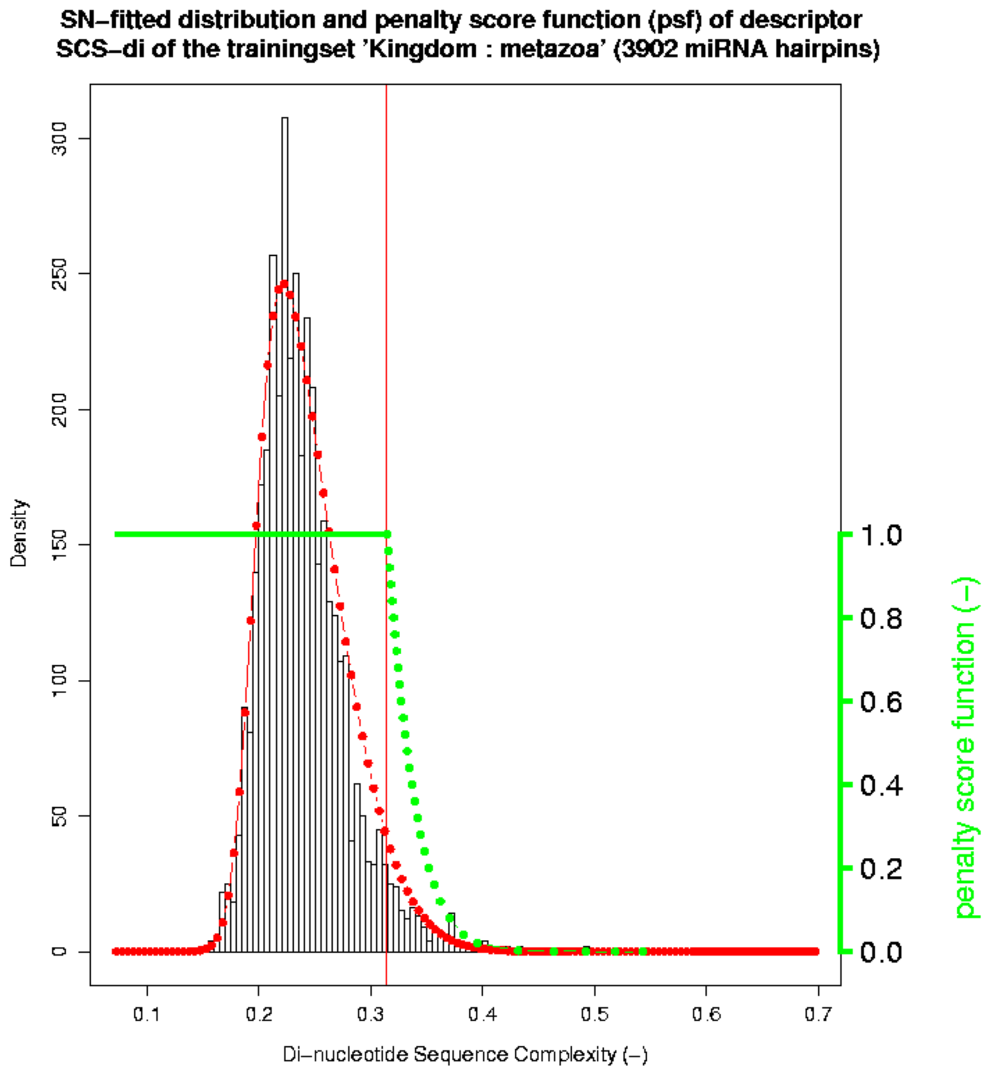

QQ-plot

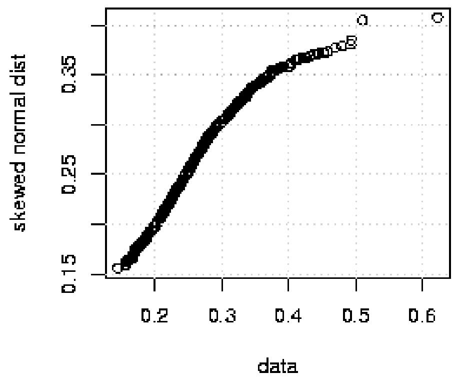

**Chisquare:**	0.0
**Location:**	0.195423
**Scale:**	0.0606903
**Shape:**	3.76599
**(Normal) mean:**	0.240969
**Observed max:**	0.623
**Observed min:**	0.145
**S<1 cut-off (95%):**	0.314374

SCS-mono

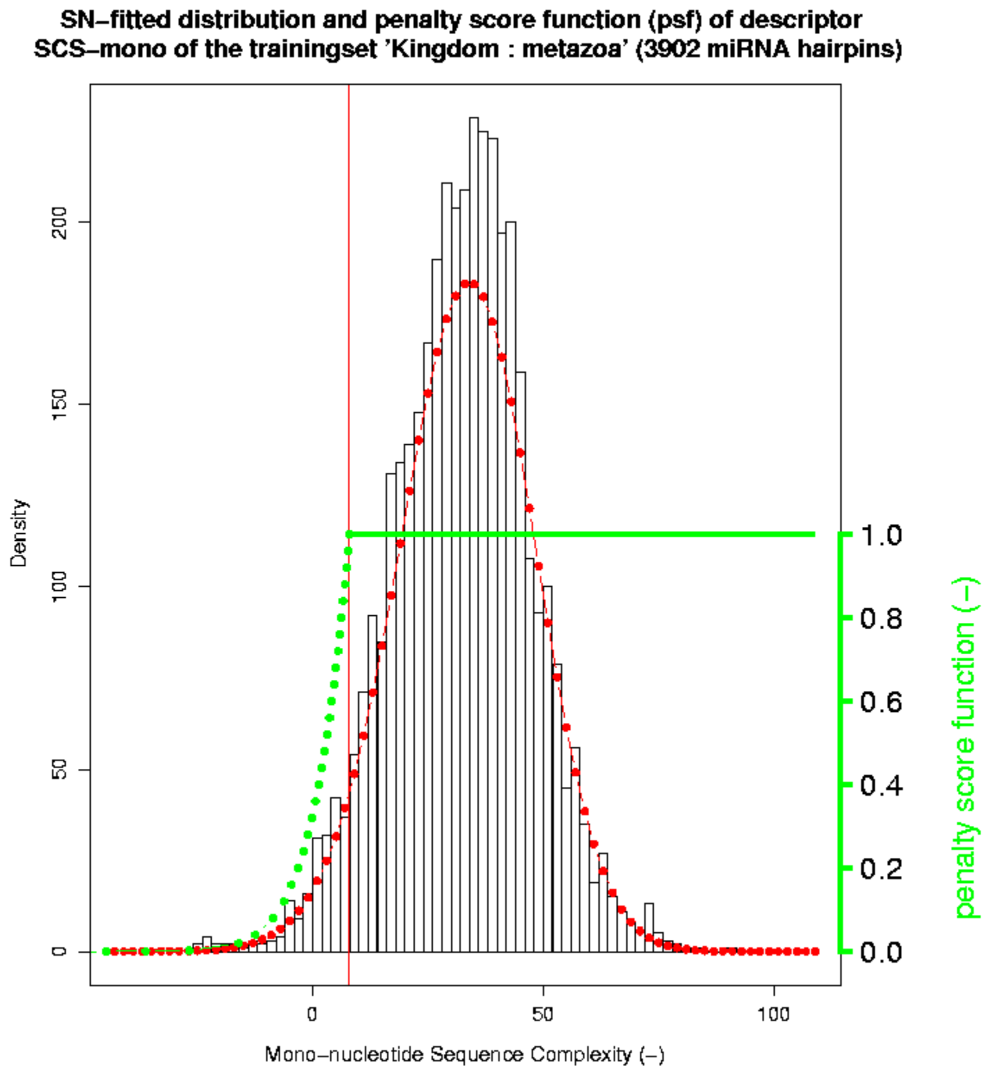

QQ-plot

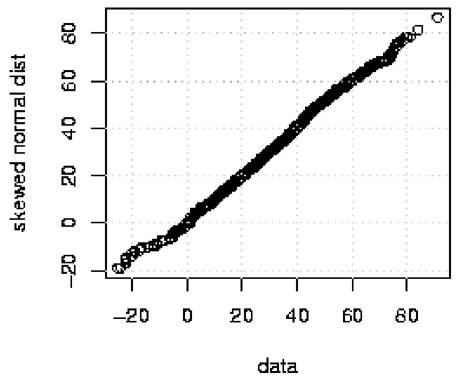

**Chisquare:**	7.02569e-08
**Location:**	42.9751
**Scale:**	17.936
**Shape:**	-0.983258
**(Normal) mean:**	32.9439
**Observed max:**	91.0
**Observed min:**	-25.0
**S<1 cut-off (95%):**	7.92857

bulgeRatio

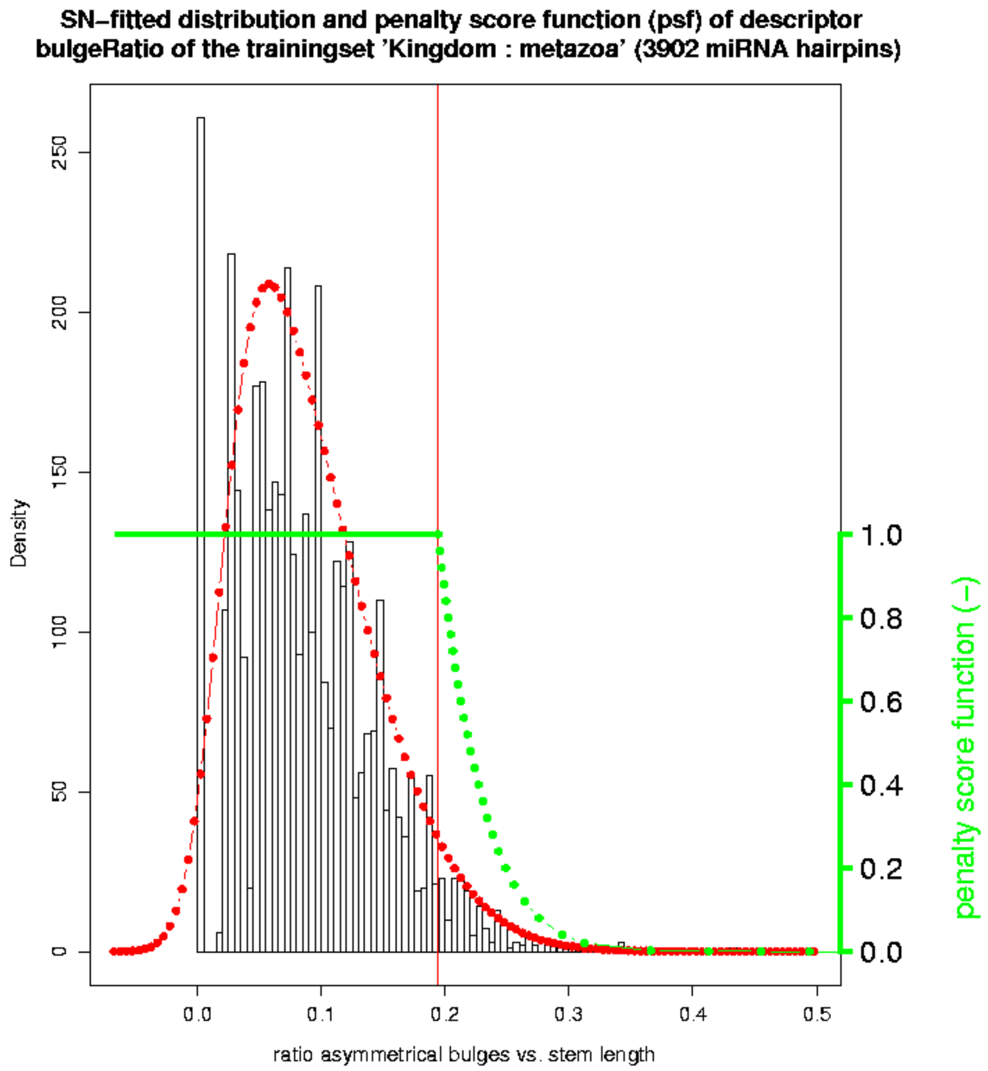

QQ-plot

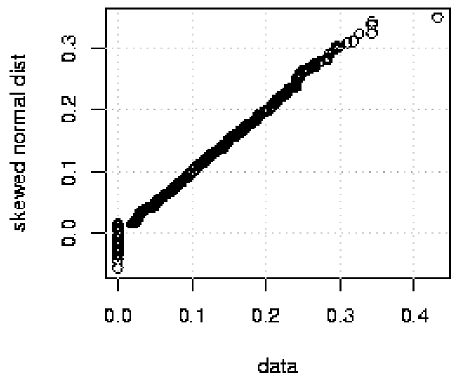

**Chisquare:**	0.0
**Location:**	0.019558
**Scale:**	0.0890496
**Shape:**	3.7891
**(Normal) mean:**	0.0884691
**Observed max:**	0.4318
**Observed min:**	0.0
**S<1 cut-off (95%):**	0.194092

D

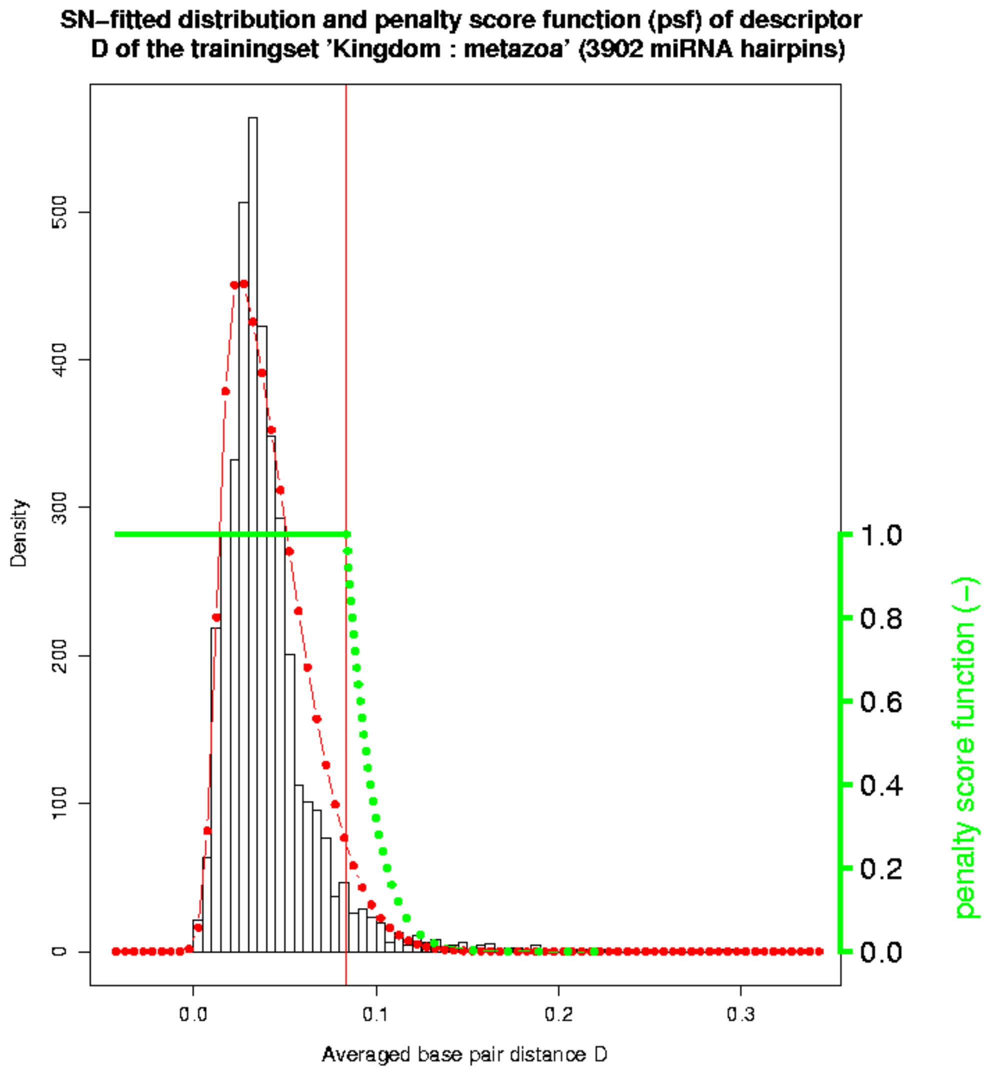

QQ-plot

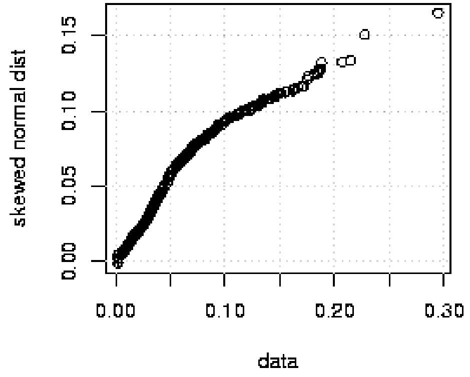

**Chisquare:**	0.0
**Location:**	0.0130984
**Scale:**	0.0360043
**Shape:**	6.1902
**(Normal) mean:**	0.0399119
**Observed max:**	0.295
**Observed min:**	0.0022
**S<1 cut-off (95%):**	0.0836655

dP

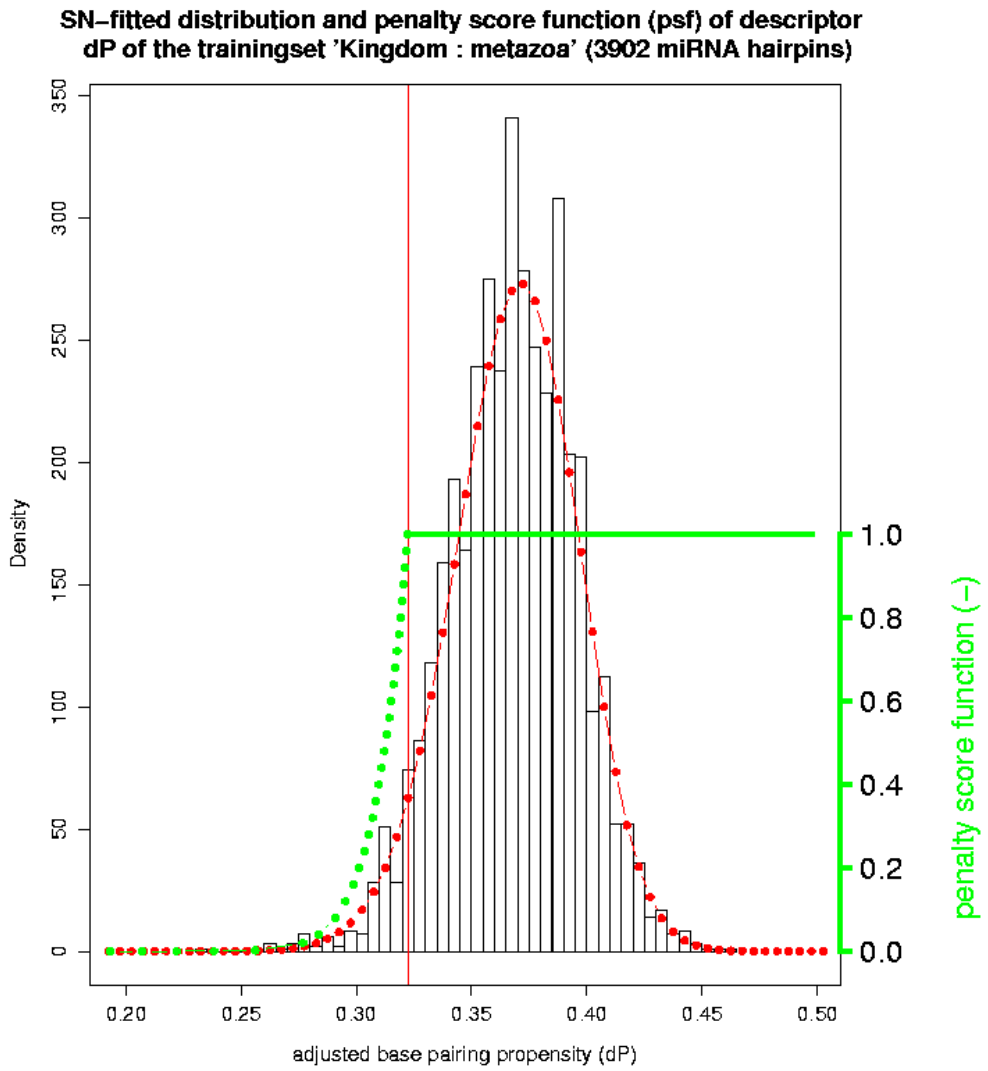

QQ-plot

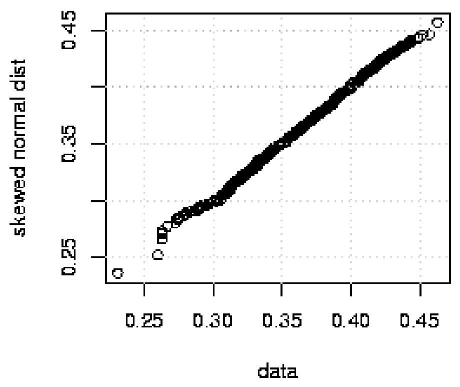

**Chisquare:**	0.0
**Location:**	0.389509
**Scale:**	0.0342635
**Shape:**	-1.17835
**(Normal) mean:**	0.368672
**Observed max:**	0.463
**Observed min:**	0.231
**S<1 cut-off (95%):**	0.322419

gapratio

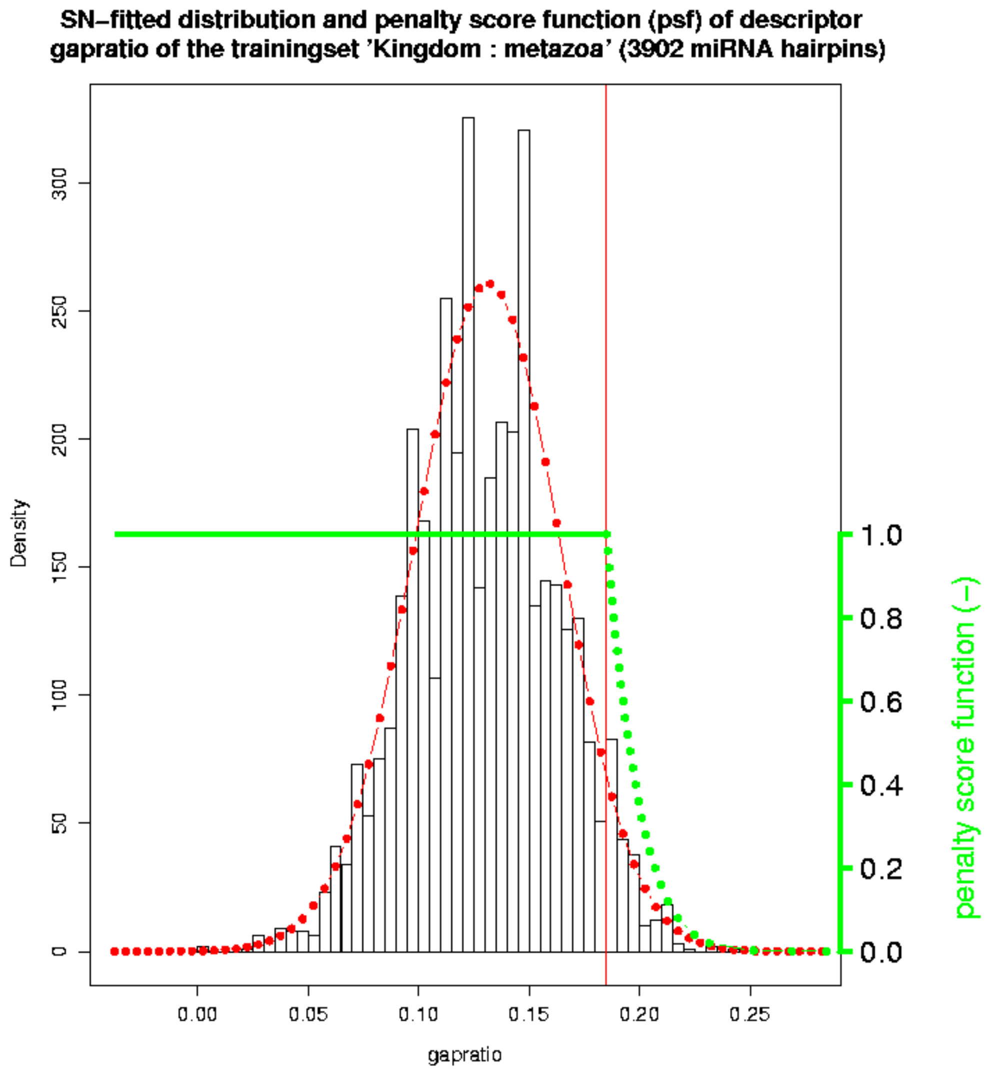

QQ-plot

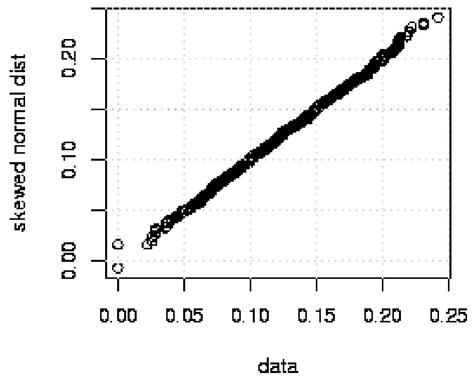

**Chisquare:**	0.0
**Location:**	0.147387
**Scale:**	0.0374218
**Shape:**	-0.684979
**(Normal) mean:**	0.130505
**Observed max:**	0.242
**Observed min:**	0.0
**S<1 cut-off (95%):**	0.184914

hairpin length

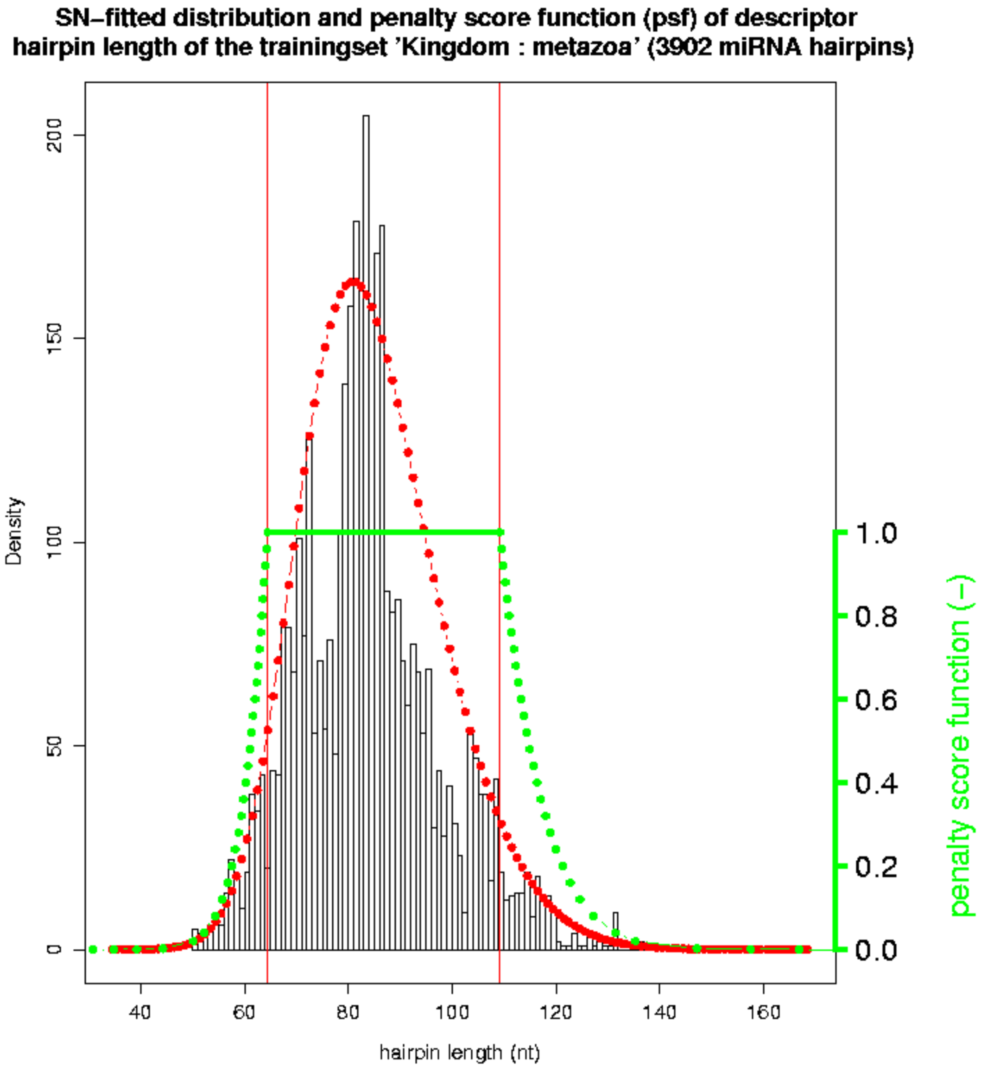

QQ-plot

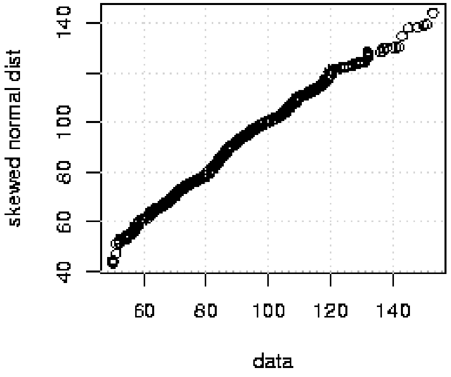

**Chisquare:**	0.0
**Location:**	70.5582
**Scale:**	19.6889
**Shape:**	2.09728
**(Normal) mean:**	84.6343
**Observed max:**	153.0
**Observed min:**	50.0
**S<1 cut-off (95%):**	109.148
**S<1 cut-off (95%):**	64.4372

largest bulge

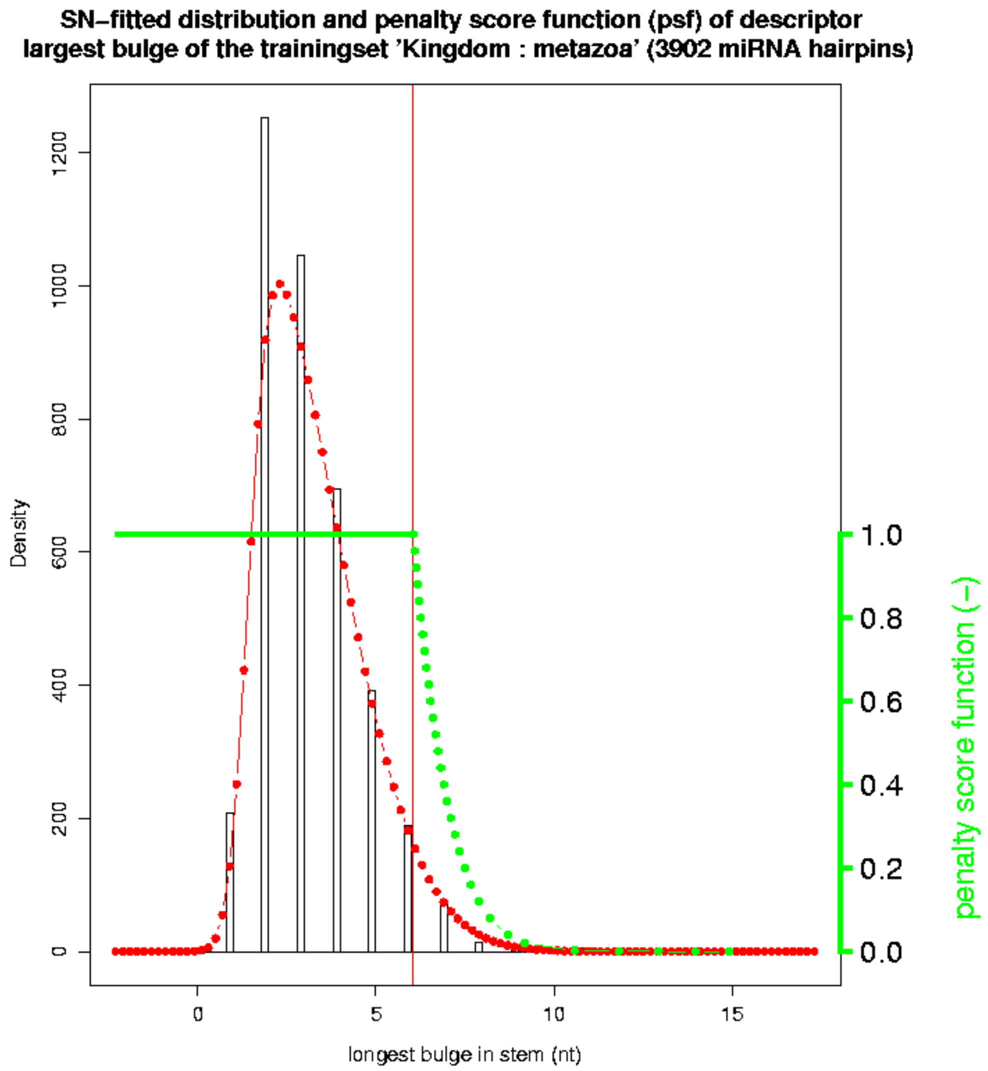

QQ-plot

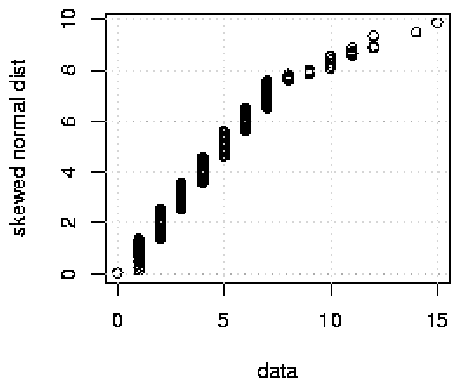

**Chisquare:**	0.0
**Location:**	1.43358
**Scale:**	2.35157
**Shape:**	5.20435
**(Normal) mean:**	3.23783
**Observed max:**	15.0
**Observed min:**	0.0
**S<1 cut-off (95%):**	6.04257

longest match-stretch

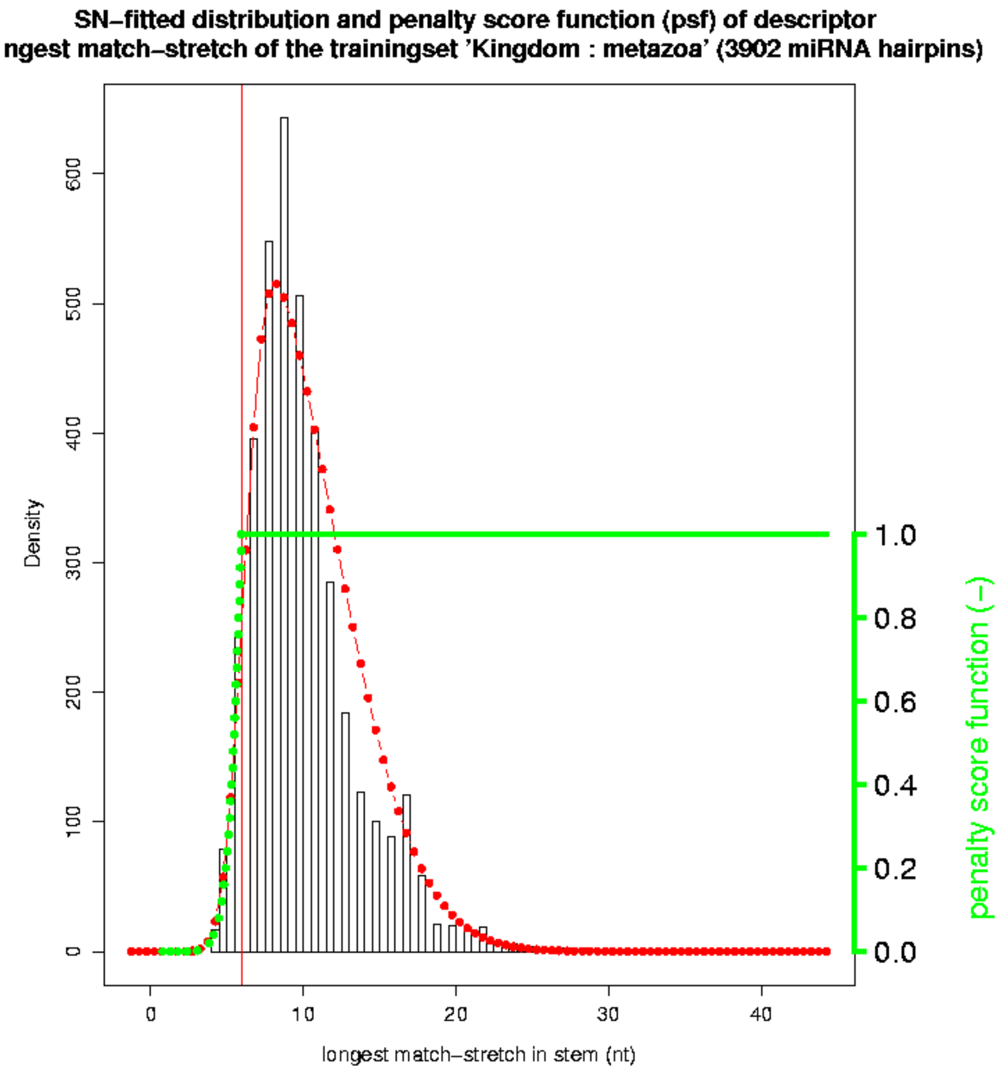

QQ-plot

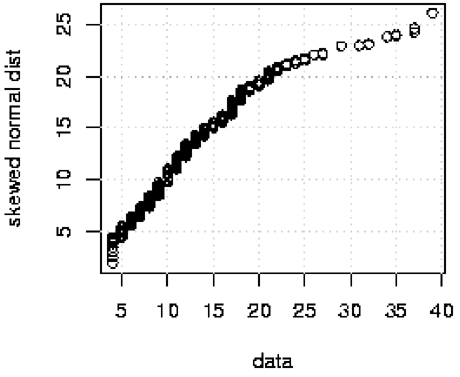

**Chisquare:**	0.0
**Location:**	6.12766
**Scale:**	5.55791
**Shape:**	5.08126
**(Normal) mean:**	10.3344
**Observed max:**	39.0
**Observed min:**	4.0
**S<1 cut-off (95%):**	5.95116

looplength

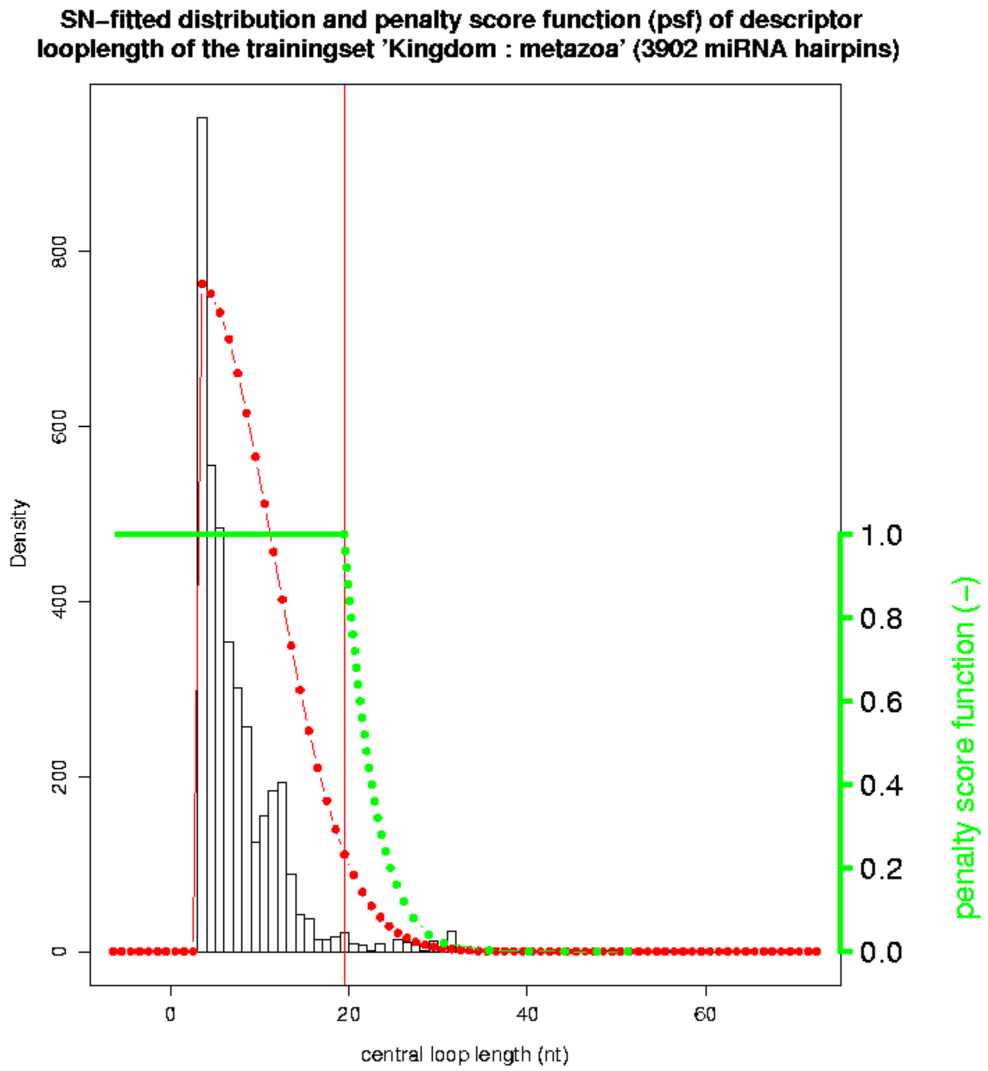

QQ-plot

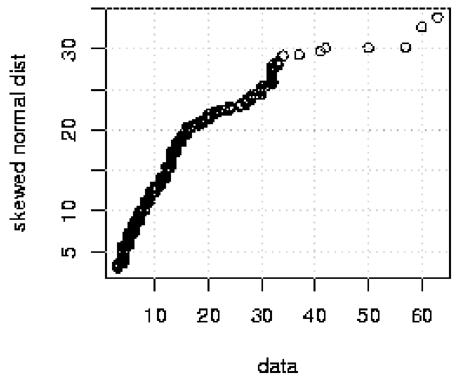

**Chisquare:**	0.0
**Location:**	2.94955
**Scale:**	8.42558
**Shape:**	569.229
**(Normal) mean:**	7.94823
**Observed max:**	63.0
**Observed min:**	3.0
**S<1 cut-off (95%):**	19.4634

match ratio stem

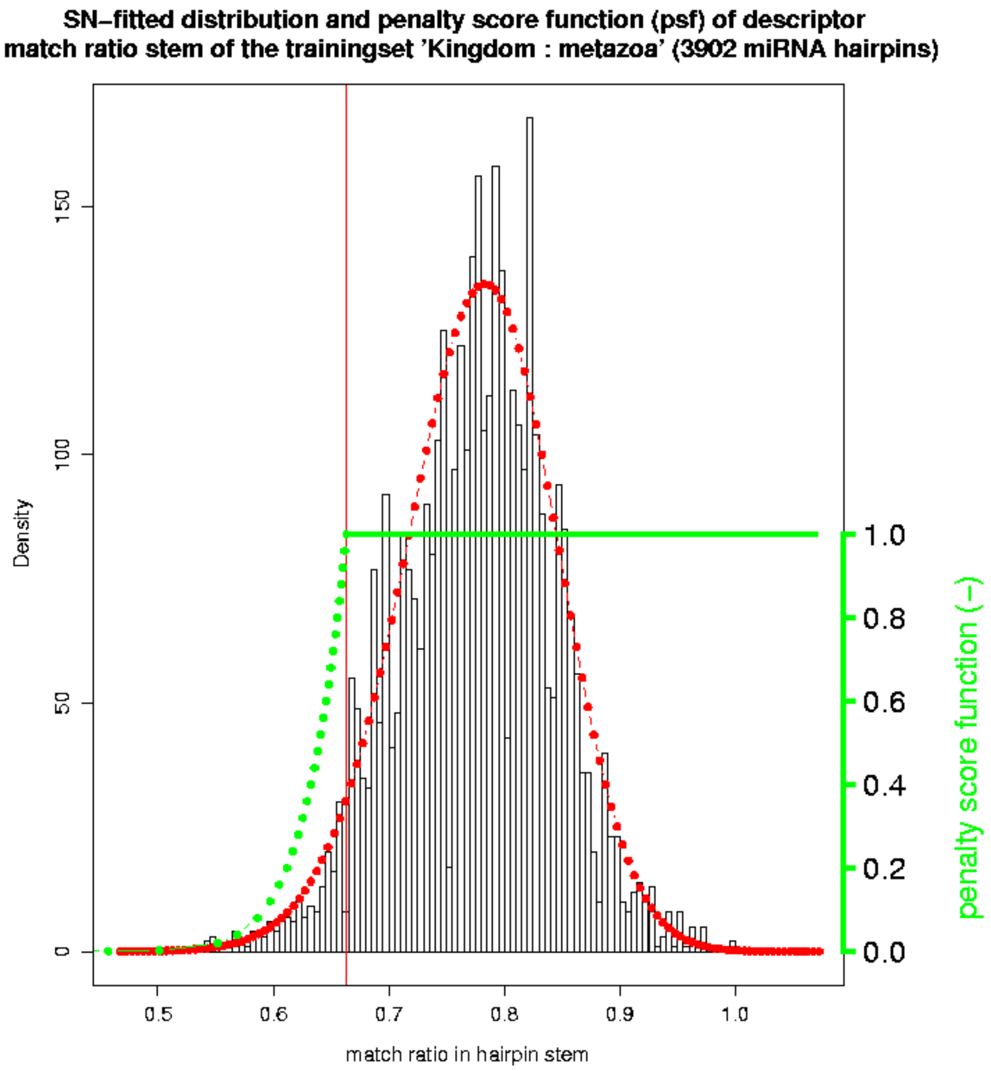

QQ-plot

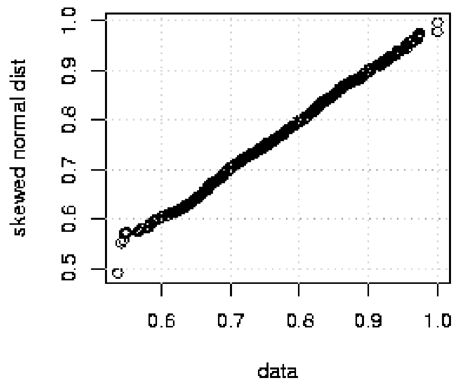

**Chisquare:**	0.0
**Location:**	0.827555
**Scale:**	0.0838567
**Shape:**	-1.15456
**(Normal) mean:**	0.776958
**Observed max:**	1.0
**Observed min:**	0.537
**S<1 cut-off (95%):**	0.663386

max match count

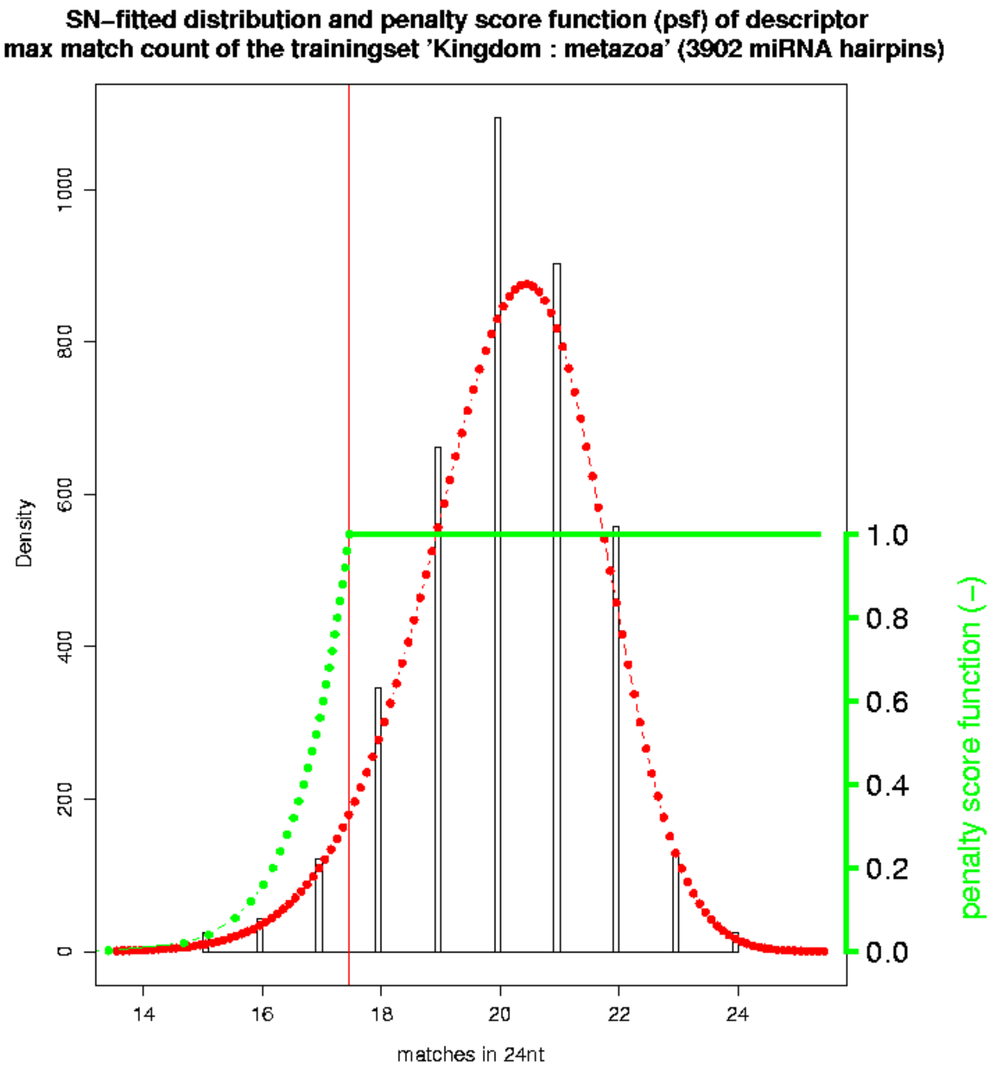

QQ-plot

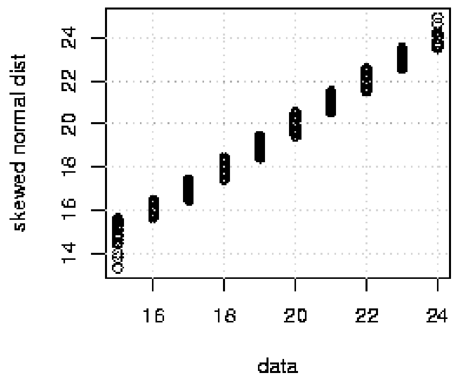

**Chisquare:**	0.0
**Location:**	21.5666
**Scale:**	2.09586
**Shape:**	-1.70829
**(Normal) mean:**	20.1248
**Observed max:**	24.0
**Observed min:**	15.0
**S<1 cut-off (95%):**	17.4589

Q	

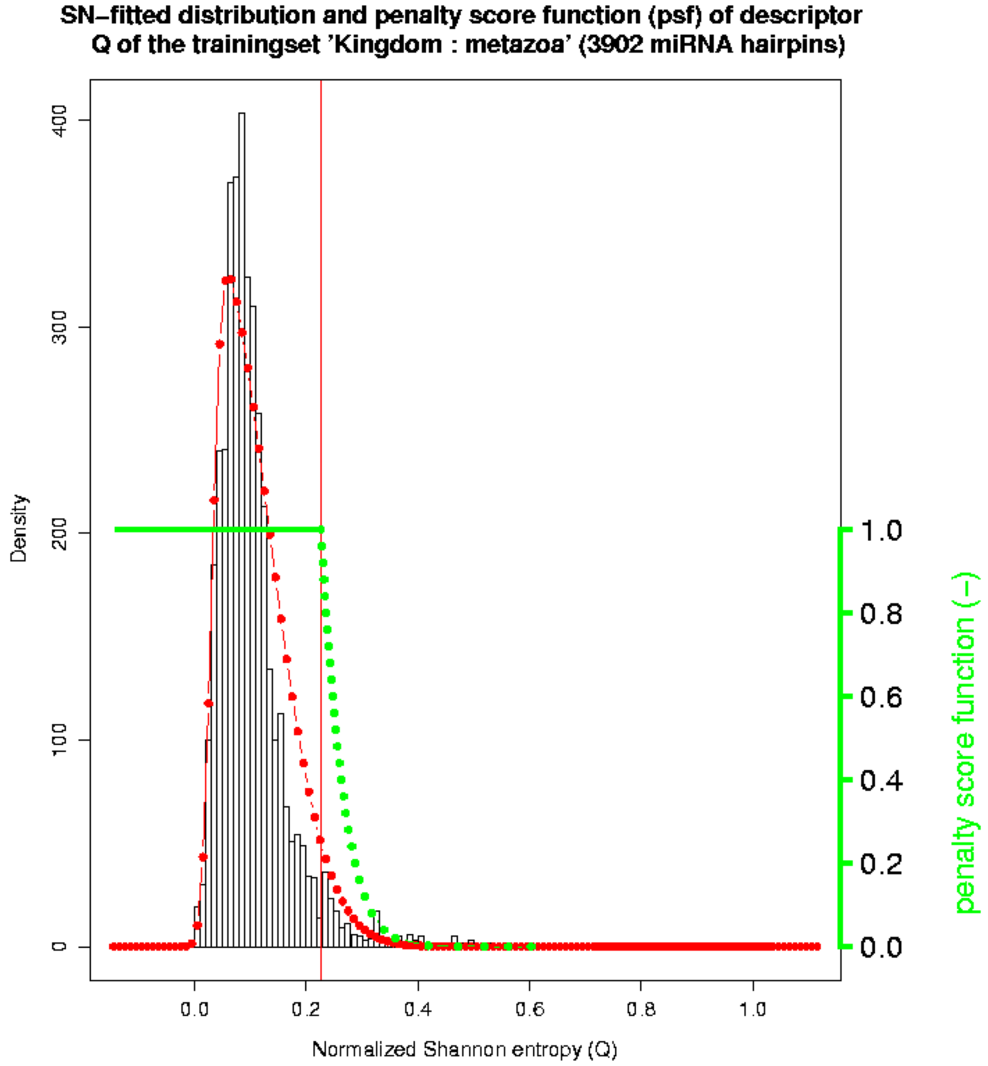

QQ-plot

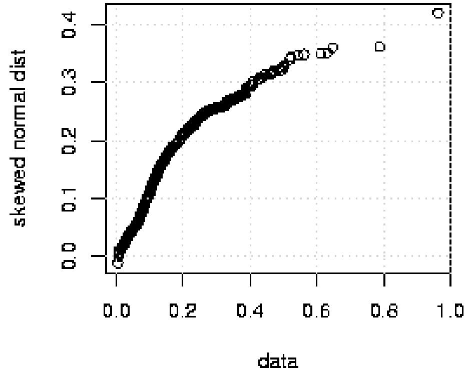

**Chisquare:**	0.0
**Location:**	0.0305664
**Scale:**	0.0998147
**Shape:**	7.30345
**(Normal) mean:**	0.103986
**Observed max:**	0.9629
**Observed min:**	0.0054
**S<1 cut-off (95%):**	0.2262

stem length

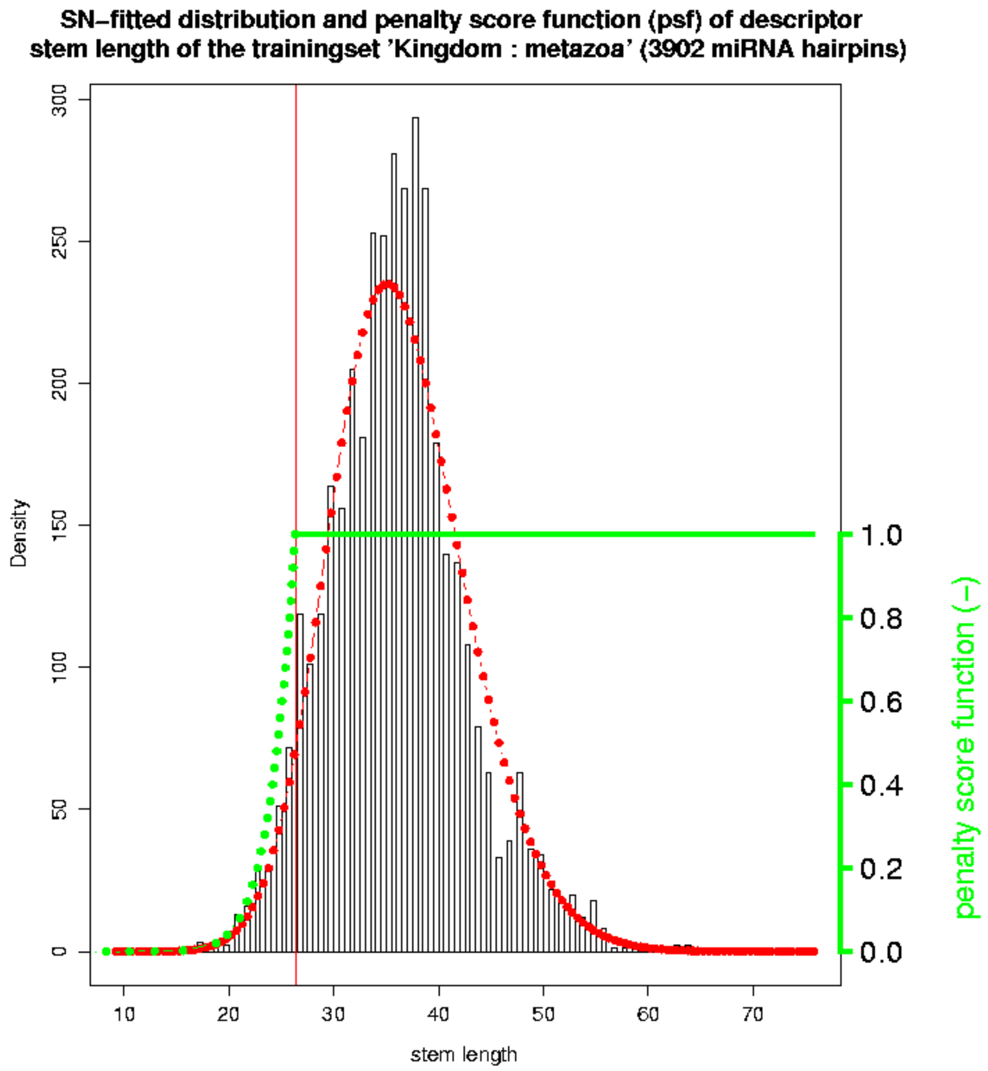

QQ-plot

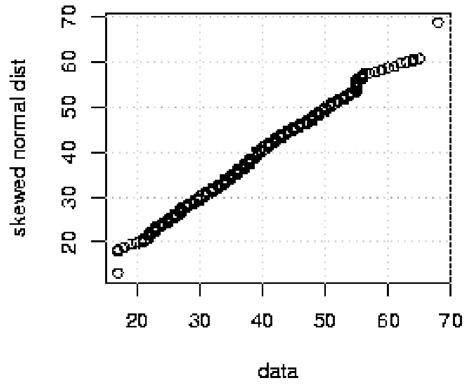

**Chisquare:**	0.0
**Location:**	30.2712
**Scale:**	8.88948
**Shape:**	1.63807
**(Normal) mean:**	36.3027
**Observed max:**	68.0
**Observed min:**	17.0
**S<1 cut-off (95%):**	26.329

stem symmetry

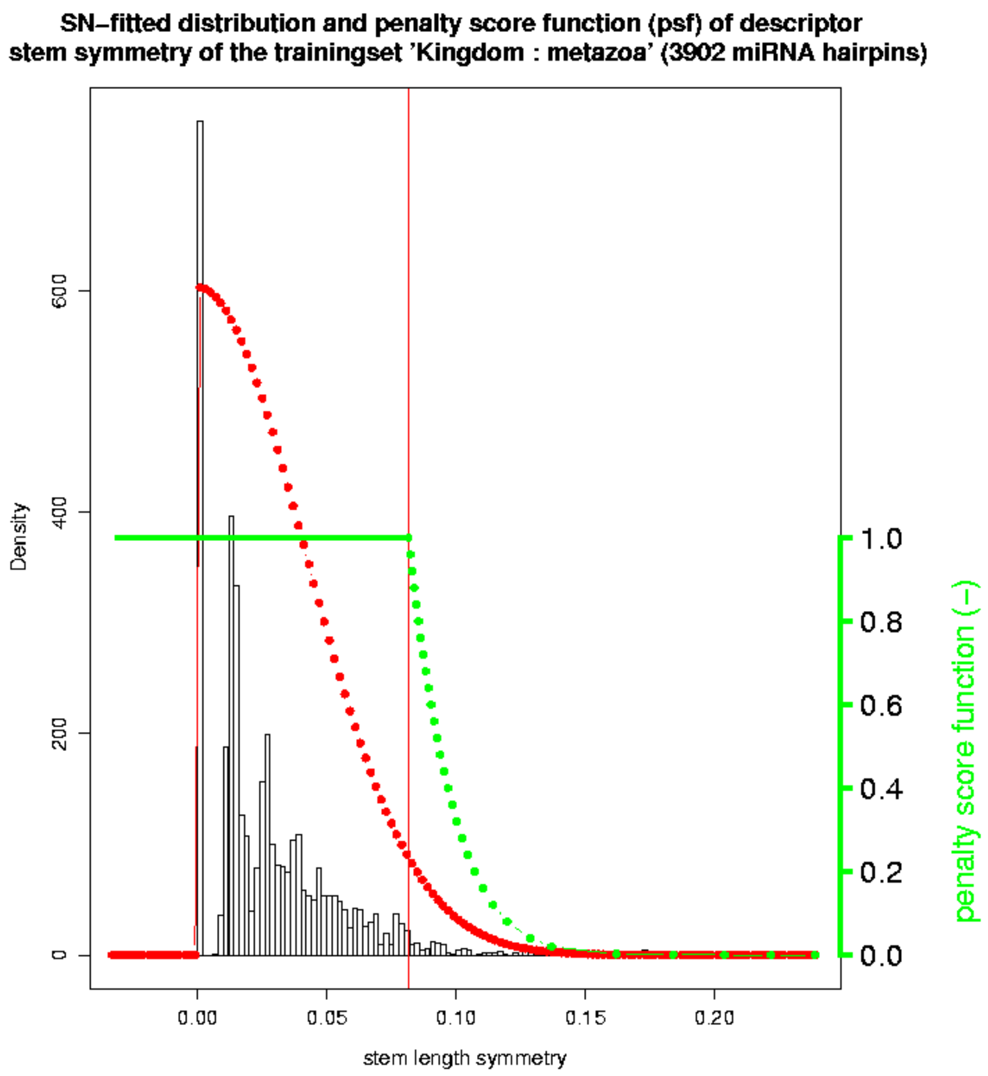

QQ-plot

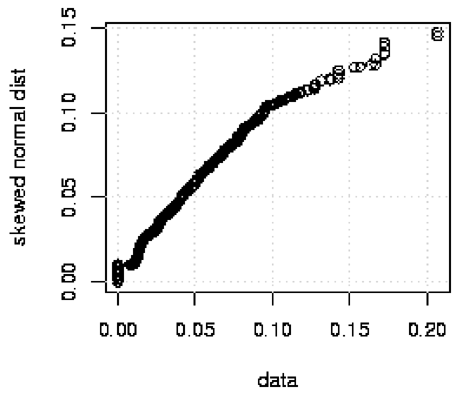

**Chisquare:**	0.0
**Location:**	-0.000174961
**Scale:**	0.0417007
**Shape:**	857.091
**(Normal) mean:**	0.0272546
**Observed max:**	0.2069
**Observed min:**	0.0
**S<1 cut-off (95%):**	0.0815569
