# Supplementary material for: In silico miRNA prediction in metazoan genomes: balancing between sensitivity and specificity
Source: BMC Genomics. 2009 Apr 30;10:204. doi: 10.1186/1471-2164-10-204 (PMC2688010; doi:10.1186/1471-2164-10-204)
Supplement: Additional file 2 — Detailed explanation of descriptors. A set of 40 potentially discriminative features of miRNA hairpins, referred to as descriptors, was defined and includes both physical and sequence characteristics of miRNA hairpins. [file 1471-2164-10-204-S2.pdf]

Additional File 1: Detailed explanation of descriptors.

**MFEahl index**	**MFEahl corrected for GC-content**	
**explanation**	MFE adjusted for hairpin length (L) and divided by GC content (GC).<br>MFE / (L * GC)	
**datarange**	[> 0.0 .. ∞]	theoretical range
	[0.343 .. 2.45]	observed on all miRNAs (4835)
	[0.343 .. 2.11]	observed on metazoan miRNAs (3896)
	[0.383 .. 2.45]	observed on viridiplantae miRNAs (857)

**MFEasl index**	**MFEasl corrected for GC-content**	
**explanation**	MFE adjusted for stem length (Ls) and divided by GC content (GC).<br>MFE / (Ls * GC)	
**datarange**	[> 0.0 .. ∞]	theoretical range
	[0.844 .. 8.42]	observed on all miRNAs (4835)
	[0.844 .. 4.75]	observed on metazoan miRNAs (3896)
	[1.13 .. 8.42]	observed on viridiplantae miRNAs (857)

**MFEindex**	**MFE corrected for GC-content**	
**explanation**	MFE of the hairpin divided by the GC content	
**datarange**	[> 0.0 .. ∞]	theoretical range
	[20.56 .. 570.18]	observed on all miRNAs (4835)
	[20.56 .. 186.17]	observed on metazoan miRNAs (3896)
	[30.47 .. 570.18]	observed on viridiplantae miRNAs (857)

**GU-match contribution**	**ratio of GU-matches vs. all matches**	
**explanation**	fraction of GU-matches relative to the match_ratio in the stem	
**datarange**	[0.0 .. 1.0]	theoretical range
	[0.0 .. 0.595]	observed on all miRNAs (4835)
	[0.0 .. 0.595]	observed on metazoan miRNAs (3896)
	[0.0 .. 0.551]	observed on viridiplantae miRNAs (857)

looplength	central loop length (nt)
explanation	Length of the central loop op the hairpin precursor (nt)
datarange	

[3 .. ∞]	theoretical range
[3 .. 360]	observed on all miRNAs (4835)
[3 .. 63]	observed on metazoan miRNAs (3896)
[3 .. 360]	observed on viridiplantae miRNAs (857)

stem length	stem length
explanation	length of the hairpin stem
datarange	

[15 .. ∞]	theoretical range
[17 .. 146]	observed on all miRNAs (4835)
[17 .. 68]	observed on metazoan miRNAs (3896)
[18 .. 146]	observed on viridiplantae miRNAs (857)

**MFE**	**minimum folding energy (kcal/mol)**
**explanation**	The Minimal Free Energy of folding (kcal/mol) or MFE of a hairpin structure is a thermodynamical measure for its stability. It can be calculated by programs as RNAfold (*Hofacker 2003*)and Mfold (*Zuker 2003*), that predict the most stable secondary structure for a RNA sequence. Because MFE is a negative number, increasingly lower values of MFE relate to increasingly more stable structures.
**datarange**	[-∞ .. < 0.0] — theoretical range<br>[-229.80 .. -7.60] — observed on all miRNAs (4835)<br>[-95.40 .. -7.60] — observed on metazoan miRNAs (3896)<br>[-229.80 .. -9.60] — observed on viridiplantae miRNAs (857)

[-∞ .. < 0.0]	theoretical range
[-229.80 .. -7.60]	observed on all miRNAs (4835)
[-95.40 .. -7.60]	observed on metazoan miRNAs (3896)
[-229.80 .. -9.60]	observed on viridiplantae miRNAs (857)

**MFEahl**	**MFE Adjusted for Hairpin Length**
**explanation**	Folding energy normalized for hairpin length. It is obtained by dividing MFE by the length (L) of the hairpin. Because longer hairpins tends to have lower MFE, MFEahl corrects for low MFEs due to long hairpin length.<br>This quantity is also known under the name dG (*Freyhult e.a. 2005*)
**datarange**	

[> 0.0 .. 1.0]	theoretical range
[0.101 .. 0.919]	observed on all miRNAs (4835)
[0.101 .. 0.919]	observed on metazoan miRNAs (3896)
[0.112 .. 0.838]	observed on viridiplantae miRNAs (857)

**MFEasl**	**MFE Adjusted for Stem Length**
**explanation**	MFE of the hairpin divided by the length of the stem (Ls) of the hairpin
**datarange**	[> 0.0 .. ∞] — theoretical range<br><br>[0.281 .. 3.65] — observed on all miRNAs (4835)<br>[0.281 .. 2.69] — observed on metazoan miRNAs (3896)<br>[0.356 .. 3.65] — observed on viridiplantae miRNAs (857)

**P**	**P-value of MFE of randomized sequence**
**explanation**	The P-value is the fraction of sequences in a set ***Xshuffled*** of shuffled sequences having a MFE lower than that of the start sequence (*Bonnet et al. 2004, Freyhult et al. 2005*). With ***M*** as the fraction with a lower MFE, and ***N*** the total of shuffled sequences, the P-value is defined as:<br><br>p(x) = M / (N +1)<br><br>1.000 shuffled sequences where generated using a dinucleotide frequency preserving procedure (*Bonnet et al. 2004*).
**datarange**	[0.0 .. 1.0] — theoretical range<br><br>[0.0 .. 0.957] — observed on all miRNAs (4835)<br>[0.0 .. 0.957] — observed on metazoan miRNAs (3896)<br>[0.0 .. 0.553] — observed on viridiplantae miRNAs (857)

**Z**	**Z-score of MFE of randomized sequence**
**explanation**	The Z-score is the number of standard deviations by which the MFE of the sequence deviates from the mean MFE of the set ***Xshuffled*** of shuffled sequences (*Le and Maizel 1989, Freyhult et al. 2005*). It is defined as:<br><br>Z(x) = (MFE(x) - < Xshuffled(x) >) / sd(Xshuffled(x))<br><br>1.000 shuffled sequences where generated using a dinucleotide frequency preserving procedure (*Bonnet et al. 2004*).
**datarange**	

[-∞ .. ∞]	theoretical range
[-29.18 .. 9.23]	observed on all miRNAs (4835)
[-15.07 .. 9.23]	observed on metazoan miRNAs (3896)
[-29.18 .. 7.93]	observed on viridiplantae miRNAs (857)

**dP**	**adjusted base pairing propensity (dP)**
**explanation**	Adjusted base pairing propensity (dP) measures the total number of base pairs present in the RNA secondary structure S (*Schultes et al., 1999*) divided by the length L in nucleotides. It removes the bias that a long sequence tends to have more base pairs. dP ranges [0.0, 0.5], 0.0 for no base pair interactions and 0.5 for maximum of L/2 base pairs (*Loong and Mishra 2007*).
**datarange**	

[0.0 .. 0.5]	theoretical range
[0.123 .. 0.480]	observed on all miRNAs (4835)
[0.231 .. 0.463]	observed on metazoan miRNAs (3896)
[0.123 .. 0.480]	observed on viridiplantae miRNAs (857)

GAsurplusCU	**surplus of GA over CU in sequence**
**explanation**	Just as GsurplusC, GAsurplusCU measures the relative abundance of Guanines and Adenosines in a sequence relative to the number of Cytosines and Uracils. With ***g***, ***c***, ***a*** and ***u*** as the number of Guanines, Cytosines, Adenosines and Uracils in the sequence, GAsurplusCU is calculated by:<br><br>GAsurplusCU = $(g + a - c - u) / (g + a + c + u)$<br><br>Perfect palindromic repeats often fold into a hairpin structure. When ignoring the central loop of prefect palindromes, the number of Guanines and Adenosines will equal the number of Cytosines and Uracils, resulting in a GAsurplusCU of 0.0.<br><br>MiRNAs are non-perfect palindromes with central loops and high incidence of bulges and mismatches. However, the tendency of having a near-zero GAsurplusCU, remains. Deviation of zero correlates with high incidence of unpaired Guanines and/or Adenonines (positive), or Cytosines and/or Uracils (negative). Because a large fraction of unpaired bases is energetically unfavorable, it is not typically observed on miRNAs.
**datarange**	[-1.0 .. 1.0] theoretical range<br><br>[-3.68e-01 .. 0.394] observed on all miRNAs (4835)<br>[-3.11e-01 .. 0.394] observed on metazoan miRNAs (3896)<br>[-3.68e-01 .. 0.233] observed on viridiplantae miRNAs (857)

[-1.0 .. 1.0]	theoretical range
[-3.68e-01 .. 0.394]	observed on all miRNAs (4835)
[-3.11e-01 .. 0.394]	observed on metazoan miRNAs (3896)
[-3.68e-01 .. 0.233]	observed on viridiplantae miRNAs (857)

GC-content	GC-content (%)
explanation	GC-content of the hairpin precursor sequence. Theoretically, GC-content ranges from 0.0 (100% AU) to 1.0 (100% GC). Extreme low and high GC-contents are rarely observed on known miRNA hairpins.
datarange	[0.0 .. 1.0] theoretical range
	[0.193 .. 0.863] observed on all miRNAs (4835)
	[0.193 .. 0.863] observed on metazoan miRNAs (3896)
	[0.240 .. 0.765] observed on viridiplantae miRNAs (857)

GCratio	G/C-ratio of precursor sequence
explanation	G/C-ratio of precursor hairpin sequence, as used before in RNA sequence analysis (*Freyhult et al. 2005*). Of all base-ratios, G/C-ratio is the single (somewhat) informative in terms of selectivity. In palindromic repeats that fold into hairpins, G/C-ratios much lower than 1.0 correlate with high incidence of unpaired Cytosines. Because this is energetically unfavorable, it is rarely observed on miRNAs. On the contrary, high G/C-ratios can be caused by high incidence of GU-basepairing.<br><br>Major disadvantage of this descriptor, is that G/C ratio runs to infinity because of a zero division in case no Cytosines are present in the (candidate) miRNA sequence. Expressing content on G and C as the descriptor GsurplusC solves the zero division.
datarange	[0 .. ∞] theoretical range
	[0.449 .. 9.67] observed on all miRNAs (4835)
	[0.575 .. 9.67] observed on metazoan miRNAs (3896)
	[0.449 .. 4.33] observed on viridiplantae miRNAs (857)

**GsurplusC**	**surplus of G over C in sequence**
**explanation**	Surplus of G over C is an alternative representation of the G/C-ratio of a sequence, as used before in RNA sequence analysis (*Freyhult et al. 2005*). With ***g*** and ***c*** as the number of Guanines and Cytosines in the sequence, GsurplusC is calculated by:<br><br>GsurplusC = (***g*** - ***c***) / (***g*** + ***c***)<br><br>In palindromic repeats that fold into hairpins, negative GsurplusC correlates with high incidence of unpaired Cytosines. Because this is energetically unfavorable, it is rarely observed on miRNAs. On the contrary, positive GsurplusC can be caused by high incidence of GU-basepairing.
**datarange**	

[-1.0 .. 1.0]	theoretical range
[-3.80e-01 .. 0.812]	observed on all miRNAs (4835)
[-2.70e-01 .. 0.812]	observed on metazoan miRNAs (3896)
[-3.80e-01 .. 0.625]	observed on viridiplantae miRNAs (857)

**hairpin length**	**hairpin length (nt)**
**explanation**	Length of the total hairpin precursor (nt).
**datarange**	

[45 .. ∞]	theoretical range
[50 .. 451]	observed on all miRNAs (4835)
[50 .. 153]	observed on metazoan miRNAs (3896)
[56 .. 451]	observed on viridiplantae miRNAs (857)

MaxDiBaseRatio	highest Di-base ratio
explanation	MaxDiBaseRatio counts the two most occuring bases in a sequence, divided by the length L of the sequence and corrected by minus 0.5. The correction of 0.5 represents the theoretical minimal ratio of any two most occurring bases. MaxDiBaseRatio ranges [0.0, 0.5], 0.0 for a sequence with equal AUGC occurences and 0.5 for a sequence consisting out of only one or two different bases. Because miRNA precursors tends to have a *complex* sequence composition, overrepresentation of one or two bases is rare, causing high values for MaxDiBaseRatio to be observed rarely.
datarange	[0.0 .. 0.5] — theoretical range
	[0.006 .. 0.362] — observed on all miRNAs (4835)
	[0.006 .. 0.362] — observed on metazoan miRNAs (3896)
	[0.008 .. 0.265] — observed on viridiplantae miRNAs (857)

minimal base occurence	minimal base occurrence
explanation	Minimal base ratio measures the number of the least occuring base in the sequence, divided by the length L. Minimal base ratio ranges [0.0, 0.25], 0.0 for a sequence lacking any of the four bases, 0.25 for a sequence with equal amounts of each base.
datarange	[0.0 .. 0.25] — theoretical range
	[0.024 .. 0.247] — observed on all miRNAs (4835)
	[0.024 .. 0.247] — observed on metazoan miRNAs (3896)
	[0.072 .. 0.246] — observed on viridiplantae miRNAs (857)

**polyA**	**longest poly-A stretch (nt)**
**explanation**	PolyA counts the longest continious stretch of Adenosine bases (nt) in the complete hairpin sequence. In practice, PolyA in miRNA hairpins ranges from 1 (not even di-Adenosines in the sequence) to hardly ever above 8. In the rare case that a large polyA-track is present, it is very often (partially) located in the central loop of the hairpin.
**datarange**	

[0 .. << L/2]	theoretical range
[1 .. 14]	observed on all miRNAs (4835)
[1 .. 11]	observed on metazoan miRNAs (3896)
[1 .. 14]	observed on viridiplantae miRNAs (857)

**polyAstem**	**longest poly-A stretch (nt) in the stem**
**explanation**	PolyA counts the longest continious stretch of Adenosine bases (nt) in the two stems of the hairpin. In practice, PolyAstem ranges from 1 (not even di-Adenosines in the stem sequences) to hardly ever above 6. In the rare case that a large polyA-track is present in the hairpin sequence, it is very often (partially) located in the central loop of the hairpin, and therefore extreme high values for polyAstem are even more rare than for polyA.
**datarange**	

[0 .. << L/2]	theoretical range
[1 .. 14]	observed on all miRNAs (4835)
[1 .. 9]	observed on metazoan miRNAs (3896)
[1 .. 14]	observed on viridiplantae miRNAs (857)

polyC	longest poly-C stretch (nt)
explanation	PolyCstem counts the longest continious stretch of Cytosine bases (nt) in the two stems of the hairpin. In practice, PolyCstem ranges from 1 (not even di-Cytosines in the stem sequences) to hardly ever above 6. In the rare case that a large polyC-track is present in the hairpin sequence, it is very often (partially) located in the central loop of the hairpin, and therefore extreme high values for polyCstem are even more rare than for polyC.
datarange	[0 .. << L/2] — theoretical range<br>[1 .. 11] — observed on all miRNAs (4835)<br>[1 .. 11] — observed on metazoan miRNAs (3896)<br>[1 .. 7] — observed on viridiplantae miRNAs (857)

polyCstem	longest poly-C stretch (nt) in the stem
explanation	PolyCstem counts the longest continious stretch of Cytosine bases (nt) in the two stems of the hairpin. In practice, PolyCstem ranges from 1 (not even di-Cytosine in the stem sequences) to hardly ever above 6. In the rare case that a large polyC-track is present in the hairpin sequence, it is very often (partially) located in the central loop of the hairpin, and therefore extreme high values for polyCstem are even more rare than for polyC.
datarange	[0 .. << L/2] — theoretical range<br>[1 .. 11] — observed on all miRNAs (4835)<br>[1 .. 11] — observed on metazoan miRNAs (3896)<br>[1 .. 7] — observed on viridiplantae miRNAs (857)

polyG	longest poly-G stretch (nt)	
explanation	PolyG counts the longest continious stretch of Guanine bases (nt) in the complete hairpin sequence. In practice, PolyG in miRNA hairpins ranges from 1 (not even di-Guanines in the sequence) to hardly ever above 8. In the rare case that a large polyG-track is present, it is very often (partially) located in the central loop of the hairpin.	
datarange	[0 .. << L/2]	theoretical range
	[1 .. 8]	observed on all miRNAs (4835)
	[1 .. 8]	observed on metazoan miRNAs (3896)
	[1 .. 7]	observed on viridiplantae miRNAs (857)

polyGstem	longest poly-G stretch (nt) in the stem	
explanation	PolyGstem counts the longest continious stretch of Guanine bases (nt) in the two stems of the hairpin. In practice, PolyGstem ranges from 1 (not even di-Guanines in the stem sequences) to hardly ever above 6. In the rare case that a large polyG-track is present in the hairpin sequence, it is very often (partially) located in the central loop of the hairpin, and therefore extreme high values for polyGstem are even more rare than for polyG.	
datarange	[0 .. << L/2]	theoretical range
	[1 .. 8]	observed on all miRNAs (4835)
	[1 .. 8]	observed on metazoan miRNAs (3896)
	[1 .. 6]	observed on viridiplantae miRNAs (857)

polyNucHairpin	longest mono-nucleotide stretch (nt) in the hairpin
explanation	PolyNucHairpin counts the longest perfect mono-nucleotide repeat in the sequence of the hairpin. In practice, mono-nucleotide tracks longer than 8 are rarely observed. In the rare case that a large mono-nucleotide track is present in the hairpin sequence, it is very often (partially) located in the central loop of the hairpin.
datarange	

[0 .. << L/2]	theoretical range
[1 .. 20]	observed on all miRNAs (4835)
[1 .. 11]	observed on metazoan miRNAs (3896)
[2 .. 20]	observed on viridiplantae miRNAs (857)

polyNucStem	longest mono-nucleotide stretch (nt) in the stem
explanation	PolyNucStem counts the longest perfect mono-nucleotide repeat in the stem sequences of the hairpin. In practice, mono-nucleotide tracks longer than 6 are rarely observed. In the rare case that a large mono-nucleotide track is present in the hairpin sequence, it is very often (partially) located in the central loop of the hairpin, and therefore extreme high values for polyNucStem are even more rare than for polyNucHairpin.
datarange	

[0 .. << L/2]	theoretical range
[1 .. 14]	observed on all miRNAs (4835)
[1 .. 11]	observed on metazoan miRNAs (3896)
[2 .. 14]	observed on viridiplantae miRNAs (857)

**polyU**	**longest poly-U stretch (nt)**	
**explanation**	PolyA counts the longest continious stretch of Uracil bases (nt) in the complete hairpin sequence. In practice, PolyU in miRNA hairpins ranges from 1 (not even di-Uracil in the sequence) to hardly ever above 8. In the rare case that a large polyU-track is present, it is very often (partially) located in the central loop of the hairpin.	
**datarange**	[0 .. << L/2]	theoretical range
	[1 .. 20]	observed on all miRNAs (4835)
	[1 .. 10]	observed on metazoan miRNAs (3896)
	[1 .. 20]	observed on viridiplantae miRNAs (857)

**polyUstem**	**longest poly-U stretch (nt) in the stem**	
**explanation**	PolyUstem counts the longest continious stretch of Uracil bases (nt) in the two stems of the hairpin. In practice, PolyUstem ranges from 1 (not even di-Uracils in the stem sequences) to hardly ever above 6. In the rare case that a large polyU-track is present in the hairpin sequence, it is very often (partially) located in the central loop of the hairpin, and therefore extreme high values for polyUstem are even more rare than for polyU.	
**datarange**	[0 .. << L/2]	theoretical range
	[1 .. 10]	observed on all miRNAs (4835)
	[1 .. 9]	observed on metazoan miRNAs (3896)
	[1 .. 10]	observed on viridiplantae miRNAs (857)

SCS-di	Di-nucleotide Sequence Complexity (-)
explanation	The dinucleotide Sequence Complexity Score (SCS-di) measures the relative occurrence of the two most occurring dinucleotides. SCS-di ranges from 0.125 for a sequence with a perfectly even dinucleotide composition, to 1.0 for a sequence composed of a perfect dinucleotide repeat. Because miRNA precursors tends to have a *complex* sequence composition, overrepresentation of certain dinucleotides is rare, causing high values for SCS-di to be observed rarely.
datarange	

[0.125 .. 1.0]	theoretical range
[0.145 .. 0.623]	observed on all miRNAs (4835)
[0.145 .. 0.623]	observed on metazoan miRNAs (3896)
[0.161 .. 0.404]	observed on viridiplantae miRNAs (857)

bulgeRatio	ratio asymmetrical bulges vs. stem length
explanation	ratio of asymmetrical bulges relative to the stemalignment length
datarange	

[0.0 .. 0.5]	theoretical range
[0.0 .. 0.432]	observed on all miRNAs (4835)
[0.0 .. 0.432]	observed on metazoan miRNAs (3896)
[0.0 .. 0.336]	observed on viridiplantae miRNAs (857)

SCS-mono	Mono-nucleotide Sequence Complexity (-)
**explanation**	The mononucleotide Sequence Complexity Score (SCS-mono) is a measure for sequence complexity, that rewards base alternation and penalizes single base succession.<br>• A sequence is splitted by each base alternation, resulting in a list of mono-nucleotide tracks of lengths 1,2,3,etc.<br>• Each track of length 1 (base alternation) is rewarded with 1 point<br>• Each track of length 2 (base succession) is rewarded with 0 points<br>• Each longer track with length *tl* is penalized with -(*tl*) points<br>• The sum of all points is SCS-mono. A *complex* sequence will have a high positive score, whereas a non-complex sequence will have a low or even negative score.<br>• Example: the sequence AAUGCGGGCCA:<br>o is splitted into AA - U - G - C - GGG - CC - A<br>o is scored as 0 - 1 - 1 - 1 - -3 - 0 - 1<br>o has a SCS-mono score of 1<br>Because miRNA precursors tends to have a complex sequence composition, base alternation occurs frequently in their sequence, causing low values for SCS-mono to be observed rarely.
**datarange**	[-(L-2) .. L-1] — theoretical range<br>[-25 .. 199] — observed on all miRNAs (4835)<br>[-25 .. 91] — observed on metazoan miRNAs (3896)<br>[-14 .. 199] — observed on viridiplantae miRNAs (857)

D	**Averaged base pair distance D**
**explanation**	The Normalized base-pair distance D as defined by (*Moulton et al. 2000, Freyhult et al. 2005, Loong and Mishra 2007*).
**datarange**	

[0.0 .. 0.25]	theoretical range
[0.002 .. 0.295]	observed on all miRNAs (4835)
[0.002 .. 0.295]	observed on metazoan miRNAs (3896)
[0.003 .. 0.144]	observed on viridiplantae miRNAs (857)

gapratio	**gapratio**
**explanation**	The gapratio counts the gap openings in the hairpin structure, divided by the length of the structure. In the *stem alignment* string (see match ratio and max match count), the number of gap openings are counted. In the given example of cel-lin-4, 8 gap openings are present in its *stem alignment* with a length of 46, resulting in a gap ratio of 0.173 (8/46)
**datarange**	

[0 .. << L/4]	theoretical range
[0.0 .. 0.242]	observed on all miRNAs (4835)
[0.0 .. 0.242]	observed on metazoan miRNAs (3896)
[0.0 .. 0.200]	observed on viridiplantae miRNAs (857)

largest bulge	longest bulge in stem (nt)
explanation	Length of the largest (a)symmetrical bulge in the stem of the hairpin precursor in nucleotides. MiRNA hairpins tend to have some, but generally small bulges.<br>The range for largest bulge is 0 .. 29. MiRNA precursors without bulges exist (e.g. hsa-mir-625). The maximum bulge length is limited to 29 by the structural hairpin filter; longest known bulge is 26 (ath-MIR408).
datarange	[0 .. 29] theoretical range
	[0 .. 26] observed on all miRNAs (4835)
	[0 .. 15] observed on metazoan miRNAs (3896)
	[0 .. 26] observed on viridiplantae miRNAs (857)

longest match-stretch	longest match-stretch in stem (nt)
explanation	Longest continious stretch of matches in the miRNA hairpin (nt). A typical miRNA hairpin has at least a single, reasonable long continious stretch of matches, mostly in the part where the mature miRNA is located. The lowest observed value for the longest match-stretch is 4 (observed on several miRNAs).
datarange	[1 .. ∞] theoretical range
	[4 .. 68] observed on all miRNAs (4835)
	[4 .. 39] observed on metazoan miRNAs (3896)
	[5 .. 68] observed on viridiplantae miRNAs (857)

match ratio stem — **match ratio in hairpin stem**

explanation

Ratio of unmatched positions in the stem structure of the miRNA hairpin precursor. Hairpin structure is as defined by Nam and coworkers (*Nam et al. 2005*). In the example below, the structure of cel-let-7 is visualized, together with its *stem alignment* in string notation.

```
     ---   g    uu-   u    c    a       u   - u
ugcuu   ccg ccug   ccc gaga cuca gugugag gua c a
|||||   ||| ||||   ||| |||| |||| ||||||| ||| |
acgag   ggc ggac   ggg cucu gggu cacacuu cgu g u
     uuu   a    cau   c    c    c       -   a u
```

mmmmgBBBmmmxmmmmxxBmmmxmmmmxmgmmxmmmmmmmgbmgmBm

From the *stem alignment*, the match ratio is calculated as the number of matched positions (m, g) divided by the length of the stem alignment string. In the above example of cel-lin-4, the match ratio is 0.74. Match ratio ranges from 0.45 to 1.0 for a miRNA hairpin without mismatches or bulges. The minimum value (0.45) is set as constrain in our structural hairpin model, whereas the lowest observed value on known miRNAs is 0.53 (ath-MIR408)

datarange

[0.45 .. 1.0]	theoretical range
[0.525 .. 1.00]	observed on all miRNAs (4835)
[0.537 .. 1.00]	observed on metazoan miRNAs (3896)
[0.525 .. 1.00]	observed on viridiplantae miRNAs (857)

max match count	matches in 24nt
**explanation**	Max match count is the highest number of matches in 24 positions in the *stem alignment* string of the hairpin structure, as defined by Nam and coworkers (*Nam et al. 2005*). In the example below, the structure of cel-let-7 is visualized, together with its *stem alignment* in string notation.

```
     ---   g    uu-   u    c    a       u   - u
ugcuu   ccg ccug   ccc gaga cuca gugugag gua c a
|||||   ||| ||||   ||| |||| |||| ||||||| ||| |
acgag   ggc ggac   ggg cucu gggu cacacuu cgu g u
     uuu   a    cau   c    c    c       -   a u
```

mmmmgBBBmmmxmmmmxxBmmmxmmmmxmgmmxmmmmmmgbmgmBm

A window of 24 positions is slided with steps of 1 over the *stem alignment* string, and the number of matched positions (m, g) is counted. The highest observed number is the max match count. In the above example of cel-lin-4, max match count is 20; its corresponding window is marked in blue.
Max match count ranges from 14 to 24. The minimum value (14) is set as constrain in our structural hairpin model.

datarange

[14 .. 24]	theoretical range
[15 .. 24]	observed on all miRNAs (4835)
[15 .. 24]	observed on metazoan miRNAs (3896)
[15 .. 24]	observed on viridiplantae miRNAs (857)

Q	Normalized Shannon entropy (Q)	
explanation	Normalized Shannon entropy (Q) characterizes the base-pairing probability distribution (BPPD) per base in a sequence s as a chaotic dynamical system (*Huynen et al. 1997, Schultes et al. 1999, Freyhult et al. 2005*).	
datarange	[0.0 .. 1.0]	theoretical range
	[0.004 .. 0.963]	observed on all miRNAs (4835)
	[0.005 .. 0.963]	observed on metazoan miRNAs (3896)
	[0.004 .. 0.502]	observed on viridiplantae miRNAs (857)

stem symmetry	stem length symmetry	
explanation	Stem symmetry is defined as **the absolute difference in stem length, divided by the total length of both stems.** Stem lengths are measured in nucleotides, not in structural positions. This results in a value of 0.0 for the perfect symmetrical hairpin.<br>Typical miRNA hairpin precursor lack large or many side-bulges, and as a consequence have rather symmetrical stems. When gaps and bulges do appear, the amount of mismatches is to a large extend balanced over both stems of the hairpin.	
datarange	[0.0 .. << 1.0]	theoretical range
	[0.0 .. 0.207]	observed on all miRNAs (4835)
	[0.0 .. 0.207]	observed on metazoan miRNAs (3896)
	[0.0 .. 0.123]	observed on viridiplantae miRNAs (857)
