# Supplementary material for: In silico miRNA prediction in metazoan genomes: balancing between sensitivity and specificity
Source: BMC Genomics. 2009 Apr 30;10:204. doi: 10.1186/1471-2164-10-204 (PMC2688010; doi:10.1186/1471-2164-10-204)
Supplement: Additional file 3 — Descriptor interdependency. Correlation among descriptors in their S<1 fractions, assessed by Cohen's kappa coefficient κ of all 780 possible pairs of descriptors, using the miRNA hairpins of the taxonomic set Metazoa. [file 1471-2164-10-204-S3.pdf]

Additional File 3: Descriptor interdependency.

Cohen's kappa coefficient of all 780 pairs of 40 descriptors from the taxonomicset *Metazoa* (3902 miRNA hairpins). S<1: number of miRNA hairpins in the S<1 fraction of a descriptor. Union; number of miRNA hairpins with either or both descriptor in the S<1 fraction. Intersection: number of miRNAs with both descriptors in their S<1 fractions. S<1 cut-off is taken at 95% of the *CDF* from the taxonomic set *Metazoa* (3902 miRNA hairpins). κ: Cohen’s κ coefficient (*Cohen 1960*).

**Descriptor1**	**S<1**	**Descriptor2**	**S<1**	**Union**	**Intersection**	**κ**
GCratio	197	GsurplusC	195	197	195	0.995
polyC	153	polyCstem	146	153	146	0.976
polyG	230	polyGstem	217	230	217	0.969
polyNucHairpin	186	polyNucStem	138	186	138	0.846
D	189	Q	189	217	161	0.844
polyA	266	polyAstem	194	266	194	0.834
P	112	Z	171	171	112	0.784
MFEahl	165	MFEasl	167	207	125	0.742
MFEahl index	181	MFEasl index	183	233	131	0.706
MFEahl index	181	Z	171	235	117	0.649
minimal base occurence	190	MaxDiBaseRatio	187	254	123	0.635
GC-content	416	MaxDiBaseRatio	187	417	186	0.59
P	112	MFEahl index	181	206	87	0.579
MFEasl index	183	Z	171	249	105	0.574
SCS-di	190	MaxDiBaseRatio	187	268	109	0.557
polyU	71	polyNucHairpin	186	186	71	0.54
SCS-di	190	minimal base occurence	190	281	99	0.497
P	112	MFEasl index	183	220	75	0.49
MFEindex	204	MFEahl index	181	287	98	0.484
MFE	182	MFEindex	204	288	98	0.482
max match count	189	match ratio stem	174	272	91	0.477
polyU	71	polyNucStem	138	159	50	0.466
GC-content	416	minimal base occurence	190	455	151	0.462

Descriptor1	S<1	Descriptor2	S<1	Union	Intersection	κ
Z	171	Q	189	278	82	0.429
GC-content	416	SCS-di	190	465	141	0.427
MFE	182	MFEahl	165	269	78	0.424
match ratio stem	174	dP	182	277	79	0.417
stem symmetry	120	bulgeRatio	177	232	65	0.416
MFEindex	204	MFEasl index	183	301	86	0.416
stem length	217	hairpin length	418	493	142	0.404
Z	171	D	189	283	77	0.4
P	112	Q	189	238	63	0.397
MFE	182	MFEasl	167	276	73	0.391
P	112	D	189	240	61	0.383
stem length	217	MFEindex	204	334	87	0.38
MFEahl	165	MFEahl index	181	276	70	0.377
MFEahl index	181	Q	189	296	74	0.37
MFEindex	204	Z	171	301	74	0.364
MFEahl index	181	D	189	299	71	0.353
match ratio stem	174	bulgeRatio	177	285	66	0.347
max match count	189	MFEahl index	181	300	70	0.347
match ratio stem	174	largest bulge	115	236	53	0.343
max match count	189	dP	182	302	69	0.341
longest match-stretch	96	gapratio	216	256	56	0.336
MFEahl	165	Z	171	275	61	0.334
MFEasl	167	MFEasl index	183	288	62	0.324
SCS-mono	187	SCS-di	190	311	66	0.317
MFE	182	stem length	217	330	69	0.311
P	112	MFEindex	204	263	53	0.31
max match count	189	MFEindex	204	326	67	0.306
match ratio stem	174	MFEahl index	181	296	59	0.301
P	112	max match count	189	252	49	0.3

Descriptor1	S<1	Descriptor2	S<1	Union	Intersection	κ
polyC	153	polyNucStem	138	244	47	0.297
MFEasl	167	MFEahl index	181	291	57	0.296
max match count	189	Z	171	301	59	0.295
gapratio	216	MFEasl index	183	333	66	0.295
MFE	182	MFEahl index	181	304	59	0.292
polyCstem	146	polyNucStem	138	239	45	0.291
largest bulge	115	stem symmetry	120	199	36	0.285
MFE	182	Z	171	297	56	0.285
MFEasl	167	Z	171	285	53	0.283
max match count	189	bulgeRatio	177	308	58	0.283
MFEahl	165	MFEasl index	183	294	54	0.278
MFEasl index	183	D	189	314	58	0.277
longest match-stretch	96	MFEahl index	181	236	41	0.273
longest match-stretch	96	MFEasl index	183	238	41	0.27
match ratio stem	174	Z	171	293	52	0.269
longest match-stretch	96	Z	171	228	39	0.269
MFEasl index	183	Q	189	316	56	0.266
P	112	MFEahl	165	237	40	0.264
hairpin length	418	MFEindex	204	524	98	0.263
largest bulge	115	bulgeRatio	177	250	42	0.261
match ratio stem	174	stem symmetry	120	252	42	0.259
MFEindex	204	MFEahl	165	315	54	0.258
match ratio stem	174	MFEahl	165	291	48	0.251
polyC	153	polyNucHairpin	186	291	48	0.251
P	112	match ratio stem	174	247	39	0.246
MFEindex	204	MFEasl	167	320	51	0.239
polyCstem	146	polyNucHairpin	186	287	45	0.239
MFE	182	MFEasl index	183	315	50	0.238
MFE	182	P	112	255	39	0.238

Descriptor1	S<1	Descriptor2	S<1	Union	Intersection	κ
max match count	189	Q	189	326	52	0.238
polyGstem	217	polyNucStem	138	307	48	0.237
largest bulge	115	dP	182	258	39	0.235
MFEahl index	181	dP	182	314	49	0.234
polyG	230	polyNucStem	138	320	48	0.227
MFEahl	165	dP	182	302	45	0.225
polyU	71	polyUstem	363	381	53	0.221
longest match-stretch	96	MFEindex	204	263	37	0.221
P	112	MFEasl	167	245	34	0.217
match ratio stem	174	Q	189	317	46	0.217
max match count	189	stem symmetry	120	271	38	0.216
polyGstem	217	polyNucHairpin	186	352	51	0.213
max match count	189	D	189	331	47	0.21
polyG	230	polyNucHairpin	186	364	52	0.208
SCS-di	190	polyNucStem	138	289	39	0.205
gapratio	216	Z	171	340	47	0.204
max match count	189	MFEahl	165	312	42	0.201
MFEindex	204	Q	189	346	47	0.199
max match count	189	MFEasl index	183	328	44	0.198
polyAstem	194	polyUstem	363	488	69	0.196
dP	182	GAsurplusCU	358	474	66	0.195
polyA	266	polyNucHairpin	186	398	54	0.194
match ratio stem	174	D	189	321	42	0.194
stem length	217	looplength	130	308	39	0.191
P	112	longest match-stretch	96	186	22	0.19
max match count	189	stem length	217	359	47	0.19
polyC	153	MaxDiBaseRatio	187	302	38	0.189
minimal base occurence	190	polyCstem	146	299	37	0.186
gapratio	216	MFEahl index	181	352	45	0.186

Descriptor1	S<1	Descriptor2	S<1	Union	Intersection	κ
minimal base occurence	190	polyC	153	305	38	0.186
MFE	182	hairpin length	418	529	71	0.184
SCS-di	190	polyNucHairpin	186	334	42	0.184
polyCstem	146	MaxDiBaseRatio	187	297	36	0.182
max match count	189	GAsurplusCU	358	483	64	0.182
MFE	182	Q	189	330	41	0.182
match ratio stem	174	GAsurplusCU	358	471	61	0.18
MFEindex	204	D	189	350	43	0.177
polyUstem	363	polyNucStem	138	446	55	0.177
max match count	189	largest bulge	115	273	31	0.174
bulgeRatio	177	D	189	327	39	0.174
P	112	GU-match contribution	184	266	30	0.173
MFEahl	165	Q	189	317	37	0.172
MFEahl	165	D	189	317	37	0.172
longest match-stretch	96	MFEasl	167	237	26	0.172
GsurplusC	195	GAsurplusCU	358	491	62	0.171
SCS-di	190	GAsurplusCU	358	487	61	0.17
SCS-di	190	polyG	230	375	45	0.17
polyA	266	polyUstem	363	555	74	0.17
GCratio	197	GAsurplusCU	358	493	62	0.169
SCS-mono	187	polyNucHairpin	186	334	39	0.169
SCS-di	190	polyGstem	217	364	43	0.168
P	112	dP	182	265	29	0.168
SCS-mono	187	polyG	230	373	44	0.167
SCS-di	190	Z	171	324	37	0.167
longest match-stretch	96	MFEahl	165	236	25	0.166
polyUstem	363	polyNucHairpin	186	489	60	0.166
dP	182	Z	171	317	36	0.166
SCS-mono	187	polyGstem	217	362	42	0.165

Descriptor1	S<1	Descriptor2	S<1	Union	Intersection	κ
GU-match contribution	184	Z	171	319	36	0.165
MFE	182	D	189	333	38	0.165
GU-match contribution	184	Q	189	335	38	0.164
bulgeRatio	177	Z	171	313	35	0.164
MFEahl index	181	bulgeRatio	177	322	36	0.163
SCS-mono	187	polyNucStem	138	293	32	0.163
match ratio stem	174	MFEasl index	183	321	36	0.163
MFEahl index	181	GsurplusC	195	338	38	0.162
MFEahl index	181	GCratio	197	340	38	0.16
MFE	182	longest match-stretch	96	252	26	0.16
stem length	217	dP	182	359	40	0.158
GU-match contribution	184	D	189	336	37	0.158
MFEasl	167	D	189	321	35	0.158
GU-match contribution	184	MFEahl index	181	329	36	0.158
SCS-mono	187	MaxDiBaseRatio	187	337	37	0.157
bulgeRatio	177	Q	189	330	36	0.157
polyC	153	polyGstem	217	334	36	0.156
largest bulge	115	MFEahl	165	254	26	0.156
longest match-stretch	96	Q	189	259	26	0.155
P	112	SCS-di	190	274	28	0.155
MFE	182	max match count	189	335	36	0.154
polyG	230	polyC	153	346	37	0.153
MaxDiBaseRatio	187	bulgeRatio	177	329	35	0.153
MFEasl	167	Q	189	322	34	0.152
gapratio	216	MFEasl	167	346	37	0.152
MFEahl index	181	GAsurplusCU	358	484	55	0.152
MaxDiBaseRatio	187	Z	171	324	34	0.151
P	112	gapratio	216	298	30	0.151
minimal base occurence	190	bulgeRatio	177	332	35	0.151

Descriptor1	S<1	Descriptor2	S<1	Union	Intersection	κ
SCS-di	190	polyC	153	311	32	0.15
MFEindex	204	dP	182	349	37	0.15
longest match-stretch	96	D	189	260	25	0.148
MFEindex	204	GsurplusC	195	361	38	0.147
MFEindex	204	GCratio	197	363	38	0.146
GU-match contribution	184	MFEasl index	183	333	34	0.145
P	112	bulgeRatio	177	264	25	0.143
SCS-mono	187	polyAstem	194	346	35	0.142
SCS-di	190	polyCstem	146	306	30	0.142
SCS-mono	187	polyU	71	237	21	0.14
SCS-mono	187	polyA	266	411	42	0.137
GC-content	416	Z	171	531	56	0.137
P	112	MaxDiBaseRatio	187	274	25	0.136
match ratio stem	174	MFEindex	204	345	33	0.133
minimal base occurence	190	Z	171	330	31	0.132
polyGstem	217	polyCstem	146	332	31	0.132
GC-content	416	SCS-mono	187	547	56	0.128
gapratio	216	D	189	370	35	0.128
P	112	minimal base occurence	190	278	24	0.127
dP	182	Q	189	340	31	0.126
SCS-di	190	Q	189	347	32	0.126
polyG	230	polyCstem	146	345	31	0.125
gapratio	216	MFEindex	204	384	36	0.124
MFE	182	GC-content	416	544	54	0.124
GC-content	416	MFEasl index	183	545	54	0.123
GC-content	416	polyC	153	520	49	0.122
SCS-mono	187	minimal base occurence	190	346	31	0.122
MFEasl index	183	GsurplusC	195	347	31	0.122
MFEasl index	183	GCratio	197	349	31	0.12

Descriptor1	S<1	Descriptor2	S<1	Union	Intersection	κ
GC-content	416	polyCstem	146	515	47	0.118
polyNucStem	138	MaxDiBaseRatio	187	300	25	0.118
max match count	189	GsurplusC	195	353	31	0.118
gapratio	216	Q	189	372	33	0.117
max match count	189	GCratio	197	355	31	0.117
stem symmetry	120	Q	189	286	23	0.116
MFEasl index	183	bulgeRatio	177	332	28	0.115
SCS-di	190	D	189	349	30	0.115
SCS-di	190	polyU	71	243	18	0.114
match ratio stem	174	MFEasl	167	315	26	0.114
match ratio stem	174	hairpin length	418	542	50	0.113
GU-match contribution	184	minimal base occurence	190	345	29	0.113
MFEahl	165	bulgeRatio	177	316	26	0.113
GC-content	416	MFEahl	165	533	48	0.111
polyG	230	Z	171	370	31	0.11
largest bulge	115	GAsurplusCU	358	438	35	0.108
polyGstem	217	MaxDiBaseRatio	187	373	31	0.108
minimal base occurence	190	GAsurplusCU	358	503	45	0.107
GC-content	416	MFEasl	167	536	47	0.107
polyG	230	MaxDiBaseRatio	187	385	32	0.106
polyGstem	217	Z	171	359	29	0.106
looplength	130	dP	182	290	22	0.106
polyG	230	D	189	387	32	0.105
max match count	189	SCS-di	190	351	28	0.104
largest bulge	115	polyAstem	194	288	21	0.103
SCS-mono	187	polyUstem	363	506	44	0.103
GC-content	416	MFEahl index	181	549	48	0.103
MaxDiBaseRatio	187	GCratio	197	356	28	0.102
max match count	189	hairpin length	418	558	49	0.102

Descriptor1	S<1	Descriptor2	S<1	Union	Intersection	κ
polyNucHairpin	186	MaxDiBaseRatio	187	346	27	0.102
max match count	189	GU-match contribution	184	346	27	0.102
MFE	182	dP	182	338	26	0.101
polyGstem	217	D	189	376	30	0.101
polyAstem	194	polyNucStem	138	309	23	0.101
max match count	189	MaxDiBaseRatio	187	349	27	0.1
P	112	GC-content	416	491	37	0.099
GU-match contribution	184	polyG	230	384	30	0.098
polyAstem	194	MFEahl	165	334	25	0.098
GC-content	416	MFEindex	204	570	50	0.098
minimal base occurence	190	polyNucStem	138	306	22	0.097
max match count	189	longest match-stretch	96	267	18	0.097
MFEindex	204	GAsurplusCU	358	518	44	0.096
SCS-mono	187	polyC	153	317	23	0.096
GC-content	416	polyNucStem	138	514	40	0.096
minimal base occurence	190	polyGstem	217	378	29	0.096
MaxDiBaseRatio	187	Q	189	350	26	0.095
MaxDiBaseRatio	187	D	189	350	26	0.095
polyC	153	bulgeRatio	177	308	22	0.095
minimal base occurence	190	polyG	230	390	30	0.095
polyG	230	Q	189	389	30	0.095
Z	171	GAsurplusCU	358	490	39	0.094
polyAstem	194	polyNucHairpin	186	354	26	0.093
hairpin length	418	GC-content	416	755	79	0.092
polyA	266	MFEahl	165	401	30	0.092
match ratio stem	174	GU-match contribution	184	334	24	0.092
MaxDiBaseRatio	187	GsurplusC	195	356	26	0.092
dP	182	D	189	346	25	0.092
GC-content	416	GCratio	197	566	47	0.091

Descriptor1	S<1	Descriptor2	S<1	Union	Intersection	κ
MFEasl index	183	GAsurplusCU	358	501	40	0.091
hairpin length	418	GAsurplusCU	358	706	70	0.091
GU-match contribution	184	MFEahl	165	326	23	0.091
polyGstem	217	Q	189	378	28	0.091
hairpin length	418	MFEahl	165	541	42	0.089
largest bulge	115	MFEahl index	181	278	18	0.089
stem symmetry	120	D	189	290	19	0.089
max match count	189	MFEasl	167	333	23	0.088
GU-match contribution	184	polyGstem	217	374	27	0.088
SCS-mono	187	polyCstem	146	312	21	0.088
MFEasl	167	bulgeRatio	177	322	22	0.088
bulgeRatio	177	GAsurplusCU	358	497	38	0.087
MFEindex	204	bulgeRatio	177	356	25	0.087
polyU	71	MFEahl	165	223	13	0.087
MFEahl index	181	MaxDiBaseRatio	187	344	24	0.087
GC-content	416	polyNucHairpin	186	558	44	0.086
SCS-di	190	MFEindex	204	368	26	0.086
polyCstem	146	bulgeRatio	177	303	20	0.086
largest bulge	115	Q	189	286	18	0.085
hairpin length	418	MaxDiBaseRatio	187	561	44	0.085
GC-content	416	GsurplusC	195	566	45	0.085
stem length	217	MFEahl index	181	372	26	0.084
MFE	182	gapratio	216	372	26	0.084
SCS-di	190	GCratio	197	362	25	0.084
GU-match contribution	184	SCS-di	190	350	24	0.084
GC-content	416	bulgeRatio	177	551	42	0.083
polyA	266	dP	182	418	30	0.083
dP	182	GsurplusC	195	353	24	0.083
MaxDiBaseRatio	187	GAsurplusCU	358	507	38	0.082

Descriptor1	S<1	Descriptor2	S<1	Union	Intersection	κ
dP	182	GCratio	197	355	24	0.082
polyUstem	363	MFEasl	167	494	36	0.082
MFEasl index	183	MaxDiBaseRatio	187	347	23	0.081
hairpin length	418	SCS-di	190	565	43	0.08
polyG	230	GAsurplusCU	358	545	43	0.08
largest bulge	115	GU-match contribution	184	282	17	0.08
hairpin length	418	minimal base occurence	190	565	43	0.08
hairpin length	418	bulgeRatio	177	554	41	0.079
minimal base occurence	190	MFEasl index	183	350	23	0.079
GU-match contribution	184	MFEindex	204	364	24	0.078
MFEahl	165	MaxDiBaseRatio	187	331	21	0.078
GU-match contribution	184	MFEasl	167	330	21	0.078
polyAstem	194	dP	182	353	23	0.078
stem symmetry	120	Z	171	275	16	0.077
GC-content	416	D	189	563	42	0.077
minimal base occurence	190	D	189	356	23	0.077
minimal base occurence	190	Q	189	356	23	0.077
gapratio	216	MFEahl	165	358	23	0.076
P	112	stem symmetry	120	220	12	0.076
stem length	217	GAsurplusCU	358	535	40	0.075
P	112	polyG	230	323	19	0.075
stem symmetry	120	MFEindex	204	306	18	0.075
GU-match contribution	184	GAsurplusCU	358	506	36	0.075
SCS-mono	187	GAsurplusCU	358	509	36	0.074
largest bulge	115	MFEasl	167	267	15	0.074
GC-content	416	Q	189	564	41	0.074
P	112	polyGstem	217	311	18	0.074
polyAstem	194	MFEasl	167	340	21	0.074
SCS-di	190	GsurplusC	195	362	23	0.074

Descriptor1	S<1	Descriptor2	S<1	Union	Intersection	κ
polyU	71	polyAstem	194	252	13	0.073
MFEahl	165	GAsurplusCU	358	490	33	0.073
largest bulge	115	Z	171	271	15	0.072
max match count	189	SCS-mono	187	354	22	0.072
largest bulge	115	polyA	266	360	21	0.072
match ratio stem	174	polyNucStem	138	295	17	0.072
minimal base occurence	190	polyNucHairpin	186	354	22	0.072
polyNucHairpin	186	MFEahl	165	331	20	0.072
stem symmetry	120	MFEahl index	181	285	16	0.072
polyA	266	polyNucStem	138	381	23	0.071
minimal base occurence	190	MFEahl	165	335	20	0.071
MFE	182	GU-match contribution	184	345	21	0.071
max match count	189	polyGstem	217	382	24	0.07
max match count	189	polyG	230	394	25	0.07
polyNucHairpin	186	GAsurplusCU	358	509	35	0.07
P	112	largest bulge	115	216	11	0.07
match ratio stem	174	GsurplusC	195	348	21	0.07
polyU	71	MFEasl	167	227	11	0.069
match ratio stem	174	GCratio	197	350	21	0.069
GC-content	416	polyG	230	601	45	0.069
GC-content	416	polyUstem	363	716	63	0.069
polyNucStem	138	GAsurplusCU	358	467	29	0.069
polyGstem	217	GAsurplusCU	358	537	38	0.068
GC-content	416	polyGstem	217	590	43	0.068
GC-content	416	GAsurplusCU	358	712	62	0.068
match ratio stem	174	SCS-di	190	344	20	0.066
MFEasl index	183	dP	182	345	20	0.066
GU-match contribution	184	polyUstem	363	513	34	0.066
MFEindex	204	MaxDiBaseRatio	187	369	22	0.066

Descriptor1	S<1	Descriptor2	S<1	Union	Intersection	κ
largest bulge	115	hairpin length	418	504	29	0.066
P	112	GAsurplusCU	358	445	25	0.066
SCS-di	190	bulgeRatio	177	347	20	0.065
MFEasl	167	MaxDiBaseRatio	187	335	19	0.065
polyGstem	217	bulgeRatio	177	372	22	0.065
polyG	230	bulgeRatio	177	384	23	0.065
stem length	217	MFEahl	165	361	21	0.065
SCS-di	190	MFEasl	167	338	19	0.064
MFE	182	match ratio stem	174	337	19	0.064
SCS-di	190	MFEahl index	181	351	20	0.063
P	112	stem length	217	313	16	0.062
GU-match contribution	184	bulgeRatio	177	342	19	0.062
polyA	266	GAsurplusCU	358	582	42	0.061
match ratio stem	174	polyGstem	217	370	21	0.061
MFEasl	167	dP	182	331	18	0.061
Q	189	GAsurplusCU	358	514	33	0.061
match ratio stem	174	polyG	230	382	22	0.061
stem length	217	Q	189	384	22	0.06
polyUstem	363	GAsurplusCU	358	668	53	0.06
longest match-stretch	96	SCS-di	190	273	13	0.06
max match count	189	minimal base occurence	190	359	20	0.06
polyUstem	363	MFEahl	165	498	30	0.059
hairpin length	418	D	189	570	37	0.059
match ratio stem	174	longest match-stretch	96	258	12	0.059
minimal base occurence	190	MFEindex	204	373	21	0.059
max match count	189	polyNucStem	138	311	16	0.059
MFE	182	MaxDiBaseRatio	187	350	19	0.058
largest bulge	115	D	189	290	14	0.058
D	189	GAsurplusCU	358	515	32	0.057

Descriptor1	S<1	Descriptor2	S<1	Union	Intersection	κ
GU-match contribution	184	stem symmetry	120	290	14	0.057
polyA	266	MFEasl	167	410	23	0.057
stem symmetry	120	GAsurplusCU	358	454	24	0.057
polyA	266	polyU	71	323	14	0.056
polyC	153	GsurplusC	195	331	17	0.056
max match count	189	GC-content	416	569	36	0.056
polyNucStem	138	MFEahl	165	289	14	0.056
hairpin length	418	polyG	230	607	41	0.055
stem length	217	SCS-mono	187	383	21	0.055
hairpin length	418	dP	182	565	35	0.055
polyAstem	194	GAsurplusCU	358	520	32	0.055
polyC	153	GCratio	197	333	17	0.055
match ratio stem	174	minimal base occurence	190	346	18	0.055
polyNucHairpin	186	MFEasl	167	336	17	0.054
polyCstem	146	GsurplusC	195	325	16	0.053
P	112	SCS-mono	187	286	13	0.053
polyCstem	146	GCratio	197	327	16	0.053
SCS-di	190	MFEahl	165	338	17	0.053
stem length	217	GsurplusC	195	391	21	0.052
minimal base occurence	190	MFEasl	167	340	17	0.052
minimal base occurence	190	MFEahl index	181	353	18	0.052
hairpin length	418	Q	189	572	35	0.052
match ratio stem	174	polyNucHairpin	186	343	17	0.051
SCS-di	190	MFEasl index	183	355	18	0.051
stem length	217	Z	171	369	19	0.051
largest bulge	115	MaxDiBaseRatio	187	289	13	0.051
stem length	217	GCratio	197	393	21	0.051
match ratio stem	174	stem length	217	372	19	0.05
P	112	polyNucStem	138	240	10	0.05

Descriptor1	S<1	Descriptor2	S<1	Union	Intersection	κ
largest bulge	115	polyG	230	330	15	0.05
stem symmetry	120	MFEahl	165	273	12	0.05
polyU	71	GAsurplusCU	358	412	17	0.05
hairpin length	418	polyGstem	217	597	38	0.05
stem symmetry	120	minimal base occurence	190	297	13	0.048
hairpin length	418	MFEasl	167	554	31	0.048
largest bulge	115	polyGstem	217	318	14	0.048
match ratio stem	174	polyAstem	194	351	17	0.048
polyC	153	Z	171	310	14	0.047
stem length	217	GC-content	416	596	37	0.047
longest match-stretch	96	GAsurplusCU	358	435	19	0.047
hairpin length	418	stem symmetry	120	513	25	0.047
gapratio	216	polyCstem	146	346	16	0.046
minimal base occurence	190	GCratio	197	369	18	0.046
MFE	182	SCS-di	190	355	17	0.046
hairpin length	418	Z	171	558	31	0.046
SCS-mono	187	Z	171	342	16	0.046
minimal base occurence	190	GsurplusC	195	367	18	0.046
dP	182	bulgeRatio	177	343	16	0.045
polyU	71	MaxDiBaseRatio	187	249	9	0.045
stem symmetry	120	MFEasl index	183	291	12	0.044
stem length	217	D	189	387	19	0.044
P	112	polyC	153	255	10	0.044
SCS-mono	187	D	189	359	17	0.044
SCS-mono	187	MFEindex	204	373	18	0.044
GU-match contribution	184	hairpin length	418	570	32	0.044
SCS-di	190	polyUstem	363	524	29	0.044
GC-content	416	polyU	71	469	18	0.044
gapratio	216	polyC	153	353	16	0.043

Descriptor1	S<1	Descriptor2	S<1	Union	Intersection	κ
stem symmetry	120	MFEasl	167	276	11	0.042
hairpin length	418	MFEahl index	181	568	31	0.041
longest match-stretch	96	dP	182	268	10	0.041
gapratio	216	MaxDiBaseRatio	187	385	18	0.04
P	112	hairpin length	418	508	22	0.04
bulgeRatio	177	GCratio	197	358	16	0.04
polyNucStem	138	Z	171	297	12	0.04
bulgeRatio	177	GsurplusC	195	356	16	0.04
match ratio stem	174	SCS-mono	187	346	15	0.039
max match count	189	polyNucHairpin	186	359	16	0.039
looplength	130	MFEahl	165	284	11	0.039
P	112	polyCstem	146	249	9	0.039
stem length	217	stem symmetry	120	324	13	0.039
P	112	polyNucHairpin	186	287	11	0.039
MFE	182	GCratio	197	363	16	0.038
polyG	230	MFEahl index	181	393	18	0.038
polyGstem	217	MFEahl index	181	381	17	0.037
max match count	189	looplength	130	307	12	0.037
stem length	217	MFEasl	167	368	16	0.037
stem symmetry	120	dP	182	291	11	0.037
largest bulge	115	minimal base occurence	190	294	11	0.037
looplength	130	polyU	71	195	6	0.037
MFE	182	stem symmetry	120	291	11	0.037
polyCstem	146	Z	171	305	12	0.037
SCS-mono	187	MFEahl index	181	353	15	0.036
largest bulge	115	GC-content	416	510	21	0.035
largest bulge	115	GCratio	197	301	11	0.035
polyNucStem	138	MFEasl	167	294	11	0.035
stem symmetry	120	polyG	230	337	13	0.035

Descriptor1	S<1	Descriptor2	S<1	Union	Intersection	κ
largest bulge	115	GsurplusC	195	299	11	0.035
GC-content	416	gapratio	216	599	33	0.034
MFEahl	165	GsurplusC	195	346	14	0.034
match ratio stem	174	polyA	266	421	19	0.034
match ratio stem	174	MaxDiBaseRatio	187	347	14	0.033
MFEahl	165	GCratio	197	348	14	0.033
stem length	217	minimal base occurence	190	390	17	0.033
SCS-mono	187	Q	189	361	15	0.033
stem symmetry	120	polyC	153	264	9	0.033
match ratio stem	174	polyC	153	315	12	0.033
largest bulge	115	polyNucStem	138	245	8	0.032
gapratio	216	polyNucStem	138	341	13	0.032
polyNucStem	138	GsurplusC	195	321	12	0.032
GCratio	197	Z	171	354	14	0.031
Z	171	GsurplusC	195	352	14	0.031
polyNucStem	138	GCratio	197	323	12	0.031
GU-match contribution	184	dP	182	352	14	0.031
gapratio	216	polyG	230	427	19	0.03
polyNucHairpin	186	dP	182	354	14	0.03
largest bulge	115	SCS-di	190	295	10	0.03
max match count	189	gapratio	216	389	16	0.029
MFE	182	GAsurplusCU	358	516	24	0.029
stem symmetry	120	MaxDiBaseRatio	187	297	10	0.029
gapratio	216	polyGstem	217	415	18	0.029
GU-match contribution	184	polyNucStem	138	311	11	0.029
stem length	217	SCS-di	190	391	16	0.028
minimal base occurence	190	gapratio	216	390	16	0.028
MFE	182	bulgeRatio	177	346	13	0.028
polyNucHairpin	186	Z	171	344	13	0.028

Descriptor1	S<1	Descriptor2	S<1	Union	Intersection	κ
hairpin length	418	SCS-mono	187	577	28	0.028
polyG	230	MFEasl index	183	397	16	0.027
stem symmetry	120	polyCstem	146	258	8	0.027
MFE	182	GsurplusC	195	363	14	0.027
stem symmetry	120	polyGstem	217	326	11	0.027
GU-match contribution	184	GC-content	416	573	27	0.026
stem symmetry	120	GCratio	197	307	10	0.026
SCS-mono	187	GsurplusC	195	368	14	0.026
largest bulge	115	MFEindex	204	309	10	0.026
hairpin length	418	MFEasl index	183	574	27	0.026
stem symmetry	120	GsurplusC	195	305	10	0.026
polyGstem	217	MFEasl index	183	385	15	0.025
polyNucStem	138	bulgeRatio	177	305	10	0.025
longest match-stretch	96	MaxDiBaseRatio	187	275	8	0.025
MFE	182	largest bulge	115	288	9	0.025
longest match-stretch	96	polyNucHairpin	186	274	8	0.025
stem length	217	MFEasl index	183	385	15	0.025
SCS-mono	187	GCratio	197	370	14	0.025
stem length	217	MaxDiBaseRatio	187	389	15	0.024
SCS-mono	187	MFEasl	167	342	12	0.024
match ratio stem	174	polyU	71	239	6	0.024
GU-match contribution	184	MaxDiBaseRatio	187	358	13	0.024
SCS-mono	187	dP	182	356	13	0.024
polyNucStem	138	MFEahl index	181	309	10	0.024
polyNucStem	138	MFEasl index	183	311	10	0.023
P	112	GsurplusC	195	298	9	0.023
SCS-di	190	gapratio	216	391	15	0.023
match ratio stem	174	polyCstem	146	310	10	0.023
MFE	182	polyU	71	247	6	0.022

Descriptor1	S<1	Descriptor2	S<1	Union	Intersection	κ
max match count	189	polyC	153	331	11	0.022
P	112	GCratio	197	300	9	0.022
polyNucStem	138	D	189	317	10	0.021
polyNucStem	138	Q	189	317	10	0.021
SCS-di	190	stem symmetry	120	301	9	0.021
SCS-mono	187	bulgeRatio	177	352	12	0.02
polyNucHairpin	186	GsurplusC	195	368	13	0.02
polyNucHairpin	186	GCratio	197	370	13	0.02
GC-content	416	stem symmetry	120	518	18	0.02
max match count	189	polyAstem	194	370	13	0.02
looplength	130	MFEahl index	181	302	9	0.02
max match count	189	polyU	71	254	6	0.02
gapratio	216	GAsurplusCU	358	549	25	0.019
polyG	230	MFEindex	204	418	16	0.019
GC-content	416	polyAstem	194	584	26	0.019
gapratio	216	polyNucHairpin	186	388	14	0.019
MFE	182	polyNucHairpin	186	356	12	0.019
longest match-stretch	96	GU-match contribution	184	273	7	0.018
polyUstem	363	MaxDiBaseRatio	187	528	22	0.018
max match count	189	polyCstem	146	325	10	0.018
longest match-stretch	96	polyGstem	217	305	8	0.018
polyGstem	217	MFEindex	204	406	15	0.018
hairpin length	418	polyC	153	550	21	0.017
stem length	217	bulgeRatio	177	381	13	0.017
largest bulge	115	polyNucHairpin	186	293	8	0.017
polyNucStem	138	dP	182	311	9	0.017
polyNucHairpin	186	D	189	363	12	0.017
gapratio	216	GsurplusC	195	397	14	0.016
polyC	153	D	189	332	10	0.016

Descriptor1	S<1	Descriptor2	S<1	Union	Intersection	κ
match ratio stem	174	GC-content	416	567	23	0.016
gapratio	216	GCratio	197	399	14	0.016
polyAstem	194	polyGstem	217	397	14	0.016
polyC	153	Q	189	332	10	0.016
largest bulge	115	stem length	217	323	9	0.016
longest match-stretch	96	polyG	230	318	8	0.015
longest match-stretch	96	GC-content	416	498	14	0.015
GU-match contribution	184	stem length	217	388	13	0.015
hairpin length	418	GsurplusC	195	588	25	0.014
hairpin length	418	GCratio	197	590	25	0.014
longest match-stretch	96	polyNucStem	138	229	5	0.014
polyNucHairpin	186	MFEahl index	181	356	11	0.014
polyA	266	MFEahl index	181	432	15	0.013
MFE	182	SCS-mono	187	358	11	0.013
match ratio stem	174	gapratio	216	378	12	0.013
polyNucHairpin	186	MFEasl index	183	358	11	0.013
GU-match contribution	184	polyU	71	250	5	0.013
GU-match contribution	184	polyNucHairpin	186	359	11	0.013
polyG	230	polyAstem	194	410	14	0.013
SCS-mono	187	MFEahl	165	342	10	0.012
GC-content	416	polyA	266	650	32	0.012
polyC	153	GAsurplusCU	358	494	17	0.012
MFE	182	minimal base occurence	190	361	11	0.012
polyC	153	MFEahl index	181	325	9	0.012
polyCstem	146	Q	189	326	9	0.012
polyCstem	146	D	189	326	9	0.012
looplength	130	polyNucHairpin	186	308	8	0.012
MFE	182	polyA	266	433	15	0.012
SCS-di	190	dP	182	361	11	0.012

Descriptor1	S<1	Descriptor2	S<1	Union	Intersection	κ
polyAstem	194	MFEahl index	181	364	11	0.011
polyNucHairpin	186	Q	189	364	11	0.011
largest bulge	115	MFEasl index	183	291	7	0.011
looplength	130	minimal base occurence	190	312	8	0.011
looplength	130	Q	189	311	8	0.011
match ratio stem	174	polyUstem	363	518	19	0.011
gapratio	216	dP	182	386	12	0.01
largest bulge	115	SCS-mono	187	295	7	0.01
longest match-stretch	96	minimal base occurence	190	280	6	0.01
largest bulge	115	polyU	71	183	3	0.01
longest match-stretch	96	GsurplusC	195	285	6	0.009
polyNucHairpin	186	bulgeRatio	177	353	10	0.009
looplength	130	Z	171	294	7	0.009
hairpin length	418	polyCstem	146	546	18	0.009
stem symmetry	120	polyU	71	188	3	0.009
longest match-stretch	96	GCratio	197	287	6	0.008
looplength	130	hairpin length	418	532	16	0.008
polyCstem	146	MFEahl index	181	319	8	0.008
match ratio stem	174	looplength	130	297	7	0.008
MFE	182	polyUstem	363	526	19	0.008
SCS-mono	187	stem symmetry	120	300	7	0.008
GU-match contribution	184	SCS-mono	187	361	10	0.007
longest match-stretch	96	hairpin length	418	502	12	0.007
polyCstem	146	GAsurplusCU	358	489	15	0.007
largest bulge	115	polyUstem	363	466	12	0.006
polyU	71	polyG	230	296	5	0.006
stem symmetry	120	polyNucStem	138	253	5	0.006
polyU	71	dP	182	249	4	0.006
longest match-stretch	96	bulgeRatio	177	268	5	0.005

Descriptor1	S<1	Descriptor2	S<1	Union	Intersection	κ
polyC	153	MFEasl index	183	328	8	0.005
max match count	189	polyA	266	441	14	0.005
looplength	130	GAsurplusCU	358	475	13	0.005
looplength	130	D	189	312	7	0.005
minimal base occurence	190	polyU	71	257	4	0.004
P	112	polyUstem	363	464	11	0.003
polyU	71	GsurplusC	195	262	4	0.003
longest match-stretch	96	SCS-mono	187	278	5	0.003
longest match-stretch	96	polyA	266	355	7	0.003
polyU	71	GCratio	197	264	4	0.003
looplength	130	polyNucStem	138	263	5	0.003
longest match-stretch	96	polyCstem	146	238	4	0.003
MaxDiBaseRatio	187	dP	182	360	9	0.002
longest match-stretch	96	polyC	153	245	4	0.002
P	112	polyA	266	370	8	0.002
P	112	looplength	130	238	4	0.002
stem symmetry	120	polyNucHairpin	186	300	6	0.002
stem length	217	polyNucStem	138	347	8	0.002
hairpin length	418	polyNucStem	138	541	15	0.001
polyCstem	146	dP	182	321	7	0.001
polyA	266	polyGstem	217	468	15	0.001
polyA	266	polyG	230	480	16	0.001
polyCstem	146	MFEasl index	183	322	7	0.001
polyU	71	polyGstem	217	284	4	0.0
longest match-stretch	96	stem symmetry	120	213	3	0.0
P	112	polyU	71	181	2	0.0
stem length	217	polyU	71	284	4	0.0
polyC	153	dP	182	328	7	-0.001
polyNucStem	138	MFEindex	204	335	7	-0.001

Descriptor1	S<1	Descriptor2	S<1	Union	Intersection	κ
GU-match contribution	184	gapratio	216	390	10	-0.001
polyU	71	bulgeRatio	177	245	3	-0.002
longest match-stretch	96	stem length	217	308	5	-0.002
polyAstem	194	MaxDiBaseRatio	187	372	9	-0.002
MFEasl	167	GCratio	197	356	8	-0.002
MFEasl	167	GsurplusC	195	354	8	-0.002
max match count	189	polyUstem	363	535	17	-0.002
polyA	266	MFEasl index	183	437	12	-0.002
stem length	217	polyNucHairpin	186	393	10	-0.002
hairpin length	418	polyU	71	482	7	-0.003
hairpin length	418	polyNucHairpin	186	585	19	-0.003
looplength	130	GsurplusC	195	319	6	-0.003
hairpin length	418	polyAstem	194	592	20	-0.003
MFE	182	polyNucStem	138	314	6	-0.003
looplength	130	GCratio	197	321	6	-0.004
polyNucHairpin	186	MFEindex	204	381	9	-0.004
SCS-mono	187	MFEasl index	183	362	8	-0.004
largest bulge	115	polyC	153	264	4	-0.004
stem length	217	polyG	230	435	12	-0.004
MFEasl	167	GAsurplusCU	358	511	14	-0.005
stem symmetry	120	polyUstem	363	473	10	-0.005
polyUstem	363	bulgeRatio	177	525	15	-0.006
polyGstem	217	dP	182	390	9	-0.006
polyU	71	polyC	153	222	2	-0.007
MFE	182	looplength	130	307	5	-0.007
SCS-di	190	polyAstem	194	376	8	-0.008
looplength	130	MaxDiBaseRatio	187	312	5	-0.008
polyA	266	Z	171	427	10	-0.008
largest bulge	115	longest match-stretch	96	209	2	-0.008

Descriptor1	S<1	Descriptor2	S<1	Union	Intersection	κ
minimal base occurence	190	polyAstem	194	376	8	-0.008
hairpin length	418	polyA	266	658	26	-0.008
looplength	130	stem symmetry	120	247	3	-0.008
polyG	230	dP	182	403	9	-0.009
polyU	71	Z	171	240	2	-0.009
longest match-stretch	96	polyU	71	166	1	-0.009
GC-content	416	dP	182	581	17	-0.009
stem length	217	polyGstem	217	424	10	-0.01
looplength	130	polyA	266	389	7	-0.01
polyCstem	146	MFEindex	204	344	6	-0.01
polyU	71	MFEahl index	181	250	2	-0.011
P	112	polyAstem	194	302	4	-0.011
polyU	71	Q	189	258	2	-0.011
minimal base occurence	190	dP	182	365	7	-0.011
polyAstem	194	MFEasl index	183	370	7	-0.012
stem length	217	polyCstem	146	357	6	-0.012
polyC	153	MFEindex	204	351	6	-0.012
longest match-stretch	96	polyAstem	194	287	3	-0.013
longest match-stretch	96	polyUstem	363	453	6	-0.013
polyA	266	MaxDiBaseRatio	187	443	10	-0.013
polyA	266	MFEindex	204	459	11	-0.013
MFE	182	polyG	230	404	8	-0.014
minimal base occurence	190	polyA	266	446	10	-0.014
polyAstem	194	Z	171	359	6	-0.014
minimal base occurence	190	polyUstem	363	539	14	-0.014
stem length	217	polyC	153	364	6	-0.014
largest bulge	115	looplength	130	243	2	-0.015
looplength	130	polyG	230	355	5	-0.015
MFE	182	polyGstem	217	392	7	-0.016

Descriptor1	S<1	Descriptor2	S<1	Union	Intersection	κ
looplength	130	polyAstem	194	320	4	-0.016
polyUstem	363	MFEasl index	183	533	13	-0.016
polyU	71	polyCstem	146	216	1	-0.016
polyUstem	363	Z	171	522	12	-0.016
polyAstem	194	bulgeRatio	177	365	6	-0.016
MFE	182	polyAstem	194	370	6	-0.017
hairpin length	418	gapratio	216	616	18	-0.017
looplength	130	MFEindex	204	330	4	-0.017
polyUstem	363	D	189	539	13	-0.018
GU-match contribution	184	polyAstem	194	372	6	-0.018
largest bulge	115	polyCstem	146	259	2	-0.018
SCS-di	190	polyA	266	447	9	-0.018
D	189	GsurplusC	195	378	6	-0.019
GCratio	197	D	189	380	6	-0.019
polyU	71	D	189	259	1	-0.019
polyUstem	363	dP	182	533	12	-0.019
polyU	71	MFEasl index	183	253	1	-0.019
longest match-stretch	96	looplength	130	225	1	-0.02
looplength	130	bulgeRatio	177	304	3	-0.02
stem symmetry	120	polyAstem	194	311	3	-0.02
polyGstem	217	GsurplusC	195	405	7	-0.02
polyUstem	363	GsurplusC	195	545	13	-0.02
polyGstem	217	GCratio	197	407	7	-0.02
polyU	71	MFEindex	204	274	1	-0.02
polyUstem	363	GCratio	197	547	13	-0.02
gapratio	216	polyA	266	472	10	-0.021
GU-match contribution	184	polyA	266	442	8	-0.021
looplength	130	SCS-mono	187	314	3	-0.021
gapratio	216	polyU	71	286	1	-0.021

Descriptor1	S<1	Descriptor2	S<1	Union	Intersection	κ
looplength	130	SCS-di	190	317	3	-0.022
polyAstem	194	MFEindex	204	392	6	-0.022
polyG	230	GsurplusC	195	418	7	-0.022
polyC	153	MFEahl	165	315	3	-0.023
looplength	130	polyC	153	281	2	-0.023
stem symmetry	120	polyA	266	382	4	-0.023
polyUstem	363	polyGstem	217	566	14	-0.023
stem symmetry	120	gapratio	216	333	3	-0.023
polyG	230	GCratio	197	420	7	-0.023
Q	189	GsurplusC	195	379	5	-0.024
hairpin length	418	polyUstem	363	751	30	-0.025
polyUstem	363	Q	189	541	11	-0.025
GCratio	197	Q	189	381	5	-0.025
gapratio	216	bulgeRatio	177	388	5	-0.026
stem length	217	polyA	266	474	9	-0.026
polyA	266	D	189	448	7	-0.027
polyG	230	polyUstem	363	579	14	-0.027
stem length	217	polyUstem	363	567	13	-0.027
polyUstem	363	MFEahl index	181	534	10	-0.027
polyCstem	146	MFEahl	165	309	2	-0.028
polyA	266	bulgeRatio	177	437	6	-0.029
looplength	130	polyCstem	146	275	1	-0.029
polyC	153	MFEasl	167	318	2	-0.03
stem length	217	polyAstem	194	406	5	-0.03
looplength	130	polyUstem	363	488	5	-0.03
polyAstem	194	GCratio	197	387	4	-0.031
polyUstem	363	polyCstem	146	503	6	-0.031
polyAstem	194	GsurplusC	195	385	4	-0.031
looplength	130	MFEasl	167	296	1	-0.032

Descriptor1	S<1	Descriptor2	S<1	Union	Intersection	κ
polyA	266	polyC	153	415	4	-0.032
polyA	266	Q	189	449	6	-0.032
polyC	153	polyUstem	363	510	6	-0.034
GU-match contribution	184	looplength	130	313	1	-0.034
stem length	217	gapratio	216	428	5	-0.034
gapratio	216	polyUstem	363	568	11	-0.034
polyAstem	194	D	189	380	3	-0.035
polyAstem	194	Q	189	380	3	-0.035
polyCstem	146	MFEasl	167	312	1	-0.035
polyA	266	polyCstem	146	409	3	-0.035
gapratio	216	polyAstem	194	406	4	-0.035
polyG	230	MFEahl	165	392	3	-0.036
polyG	230	MFEasl	167	394	3	-0.036
MFE	182	polyCstem	146	327	1	-0.037
GU-match contribution	184	polyCstem	146	329	1	-0.037
looplength	130	polyGstem	217	346	1	-0.037
looplength	130	gapratio	216	345	1	-0.037
MFE	182	polyC	153	334	1	-0.038
polyAstem	194	polyCstem	146	339	1	-0.038
SCS-mono	187	gapratio	216	400	3	-0.038
looplength	130	GC-content	416	542	4	-0.038
GU-match contribution	184	polyC	153	336	1	-0.039
polyGstem	217	MFEahl	165	380	2	-0.039
largest bulge	115	gapratio	216	331	0	-0.04
polyC	153	polyAstem	194	346	1	-0.04
polyGstem	217	MFEasl	167	382	2	-0.04
looplength	130	MFEasl index	183	313	0	-0.041
polyA	266	GCratio	197	459	4	-0.043
polyA	266	GsurplusC	195	457	4	-0.043

Descriptor1	S<1	Descriptor2	S<1	Union	Intersection	κ
polyUstem	363	MFEindex	204	560	7	-0.045
GU-match contribution	184	GCratio	197	381	0	-0.051
GU-match contribution	184	GsurplusC	195	379	0	-0.051
