# Supplementary material for: In silico miRNA prediction in metazoan genomes: balancing between sensitivity and specificity
Source: BMC Genomics. 2009 Apr 30;10:204. doi: 10.1186/1471-2164-10-204 (PMC2688010; doi:10.1186/1471-2164-10-204)
Supplement: Additional file 4 — Taxonomic sets with at least 100 miRNA sequences. MiRNA sequences were divided in hierarchically organized subsets based on their taxonomic relationships. A total of 23 taxonomic sets comprised at least 100 sequences and were used for analysis. [file 1471-2164-10-204-S4.pdf]

Additional File 4: Taxonomic sets with at least 100 miRNA sequences (23).

Name	number of miRNA hairpin precursors
Kingdom : metazoa	3902
Taxonomy : Vertebrata	3394
Taxonomy : Mammalia	2458
Taxonomy : Primates	1584
Taxonomy : Hominidae	1287
Organism : Homo sapiens	718
Taxonomy : Rodentia	607
Taxonomy : Pisces	600
Organism : Mus musculus	373
Organism : Danio rerio	337
Organism : Pan troglodytes	310
Taxonomy : Arthropoda	234
Organism : Rattus norvegicus	234
Taxonomy : Nematoda	211
Taxonomy : Amphibia	184
Organism : Xenopus tropicalis	177
Organism : Gallus gallus	152
Taxonomy : Cercopithecidae	146
Organism : Tetraodon nigroviridis	132
Organism : Caenorhabditis elegans	132
Organism : Fugu rubripes	131
Organism : Monodelphis domestica	106
Taxonomy : Ruminantia	101
