# Supplementary material for: In silico miRNA prediction in metazoan genomes: balancing between sensitivity and specificity
Source: BMC Genomics. 2009 Apr 30;10:204. doi: 10.1186/1471-2164-10-204 (PMC2688010; doi:10.1186/1471-2164-10-204)
Supplement: Additional file 5 — Hairpins identified in Epstein-Barr Virus. Details of 23 hairpins with L score = 1.0, identified in Epstein-Barr Virus [EMBL: AJ507799] for the scoring model Metazoa. [file 1471-2164-10-204-S5.pdf]

Additional File 5: Identified hairpins in Epstein-Barr Virus with *L* = 1.0

List of 23 identified hairpins in Epstein-Barr Virus [EMBL:AJ507799] with *L* score=1.0 for the scoring model *Metazoa*, of which eight are known miRNAs (denoted with !! and !!- when the hairpin is opposite to the known miRNA). These 23 hairpins collapse on 20 unique loci; candidates 28941 and 30018 are alternative structures for candidates 30009 and 28561, candidate 33319 is on the opposite strand of candidate 27827.

In the text of the paper two hairpins are mentioned that are located upstream and amidst a cluster of eleven known miRNAs in an intronic region of the BART gene (*Cai et al. 2006*). These hairpins are marked in the figure below by a green bar. The color coding of the miRNA and genomic hairpins represents their *L* score: dark green for *L* = 1 and continuous lower *L* scores for the color gradient light green, yellow, orange, red, brown and black.

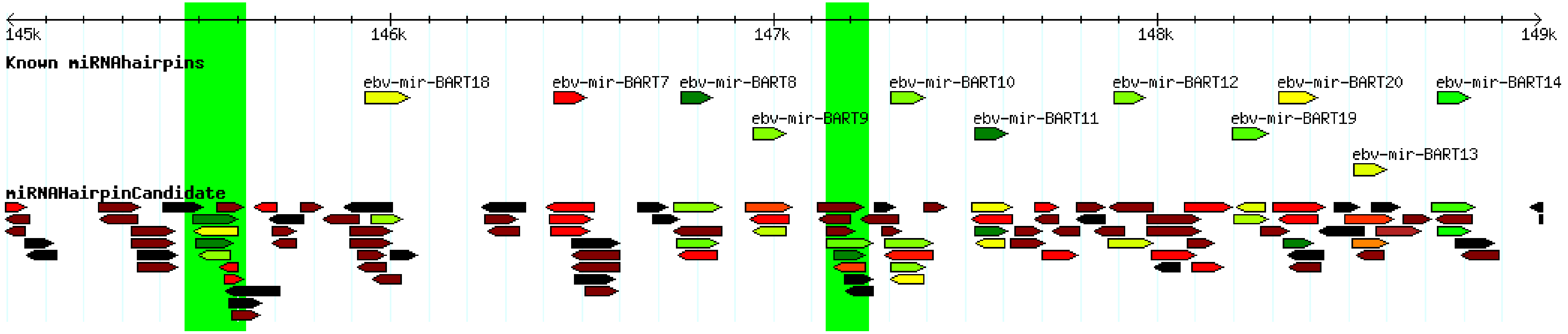

All S scores for the 18 descriptors are 1.0, resulting in a combined *L* score of 1.0. The 18 descriptors, from left to right: MFEahl, MFEahl index, Q, max match count, bulgeRatio, GU-match contribution, largest bulge, longest match-stretch, looplength, stem length, dP, SCS-mono, SCS-di, polyA, polyU, polyNucHairpin, GsurplusC, GasurplusCU.

‘Genpos’ denotes the genomic position of the hairpin:

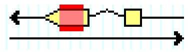 overlapping with an exon

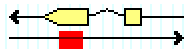 opposite to an exon

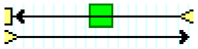 intergenic or intronic

Id	Position	Strand	Locus																			Lscore	Genpos
29419	7406..7483	+	1																			1.000	
33135	41461..41544 !!-	-	2																			1.000	
27343	42825..42938 !!	+	3																			1.000	
33383	68762..68887	-	4																			1.000	
30260	87663..87740	+	5																			1.000	
33093	103516..103588	-	6																			1.000	
32868	134006..134065	-	7																			1.000	
29668	137869..137939	+	8																			1.000	
28731	139065..139171 !!	+	9																			1.000	
30009	139208..139298 !!	+	10																			1.000	
28941	139214..139300 !!	+	10																			1.000	
29081	139507..139584 !!	+	11																			1.000	
28641	139673..139738 !!	+	12																			1.000	
31765	139777..139873 !!-	-	13																			1.000	
27827	139894..139995 !!	+	14																			1.000	
33319	139900..139989	-	14																			1.000	
29664	140361..140441	+	15																			1.000	
28561	145484..145602	+	16																			1.000	
30018	145494..145589	+	16																			1.000	
29857	147155..147236	+	17																			1.000	
29968	147524..147609 !!	+	18																			1.000	
29929	148328..148405 !!	+	19																			1.000	
32018	150845..150927	-	20																			1.000	

**id**	29419
**genomic position**	7405-7483 on AJ507799 (+) in Epstein Barr virus
**L score**	1.0
**structure**	cac acua u a g a<br>ucc ucu cugggua cauaugcug cu uau u<br>||| ||| ||||||| ||||||||| || ||| agg aga ggcccau guauacgau gg gua g<br>auu caua c a a c<br><br>mmmxxxmmmxxxxmgmmmmmxmmmmmmmmgxmgxgmm
**sequence**	(((...(((....(((((((.(((((((((.((.(((....))).)).))))))))).)))))))....)))...)))<br>ucccacucuacuacugggguaucauaugcugacuguauaugcaugaggauagcauaugcuacccggauacagauuagga<br><br>folding energy of structure: **-26.8** kcal/mol

**id**	33135
**genomic position**	41460-41544 on AJ507799 (-) in Epstein Barr virus
**L score**	1.0
**Structure**	c- c cu g a ga<br>gac cuucuugu aaccu ucag cccggguu gu ugg<br>||| |||||||| ||||| |||| ||||||||| || || a<br>cug gaggaaua uugga aguc gggccucaa cg aca<br>cc a cu g - g-<br><br>mmmxBmmgmmmgmxmmmmmxxmmmmxmmmmmgmmmbmgxbmm
**Sequence**	(((.((((((((.(((((..((((.(((((((((.((..((...)).)))))))))))).))))..))))).))))))))..)))<br>gacccuucuugucaaccucuucaggcccggguuagugaugaaacaggcaacuccggggcugaucagguuaauaaggagccguc<br><br>folding energy of structure: **-40.1** kcal/mol

**id**	27343
**genomic position**	42824-42938 on AJ507799 (+) in Epstein Barr virus
**L score**	1.0

structure

Candidate miRNA precursor

```
    g     ----  ccc    cc         a           c       ugau
acau ccaau    ga   ggcc  cacuuuuaa uucuguugcag agauagc    a
|||| |||||    ||   ||||  ||||||||| ||||||||||| |||||||    c
ugug gguua    cu   ccgg  gugaaaguu aagacggcguu ucuauug    c
    -     acau  aua    uc         a           u       uaac

mmmgbmmmmmBBBBmmxxxmmmmxxmmmmmmgmmxmmmmmggmmmgxmmmmmgm
```

Known miRNA precursor (MI0001065)

```
       a           c       ugau
cuuuuaa uucuguugcag agauagc    a
||||||| ||||||||||| |||||||    c
gaaaguu aagacggcguu ucuauug    c
       a           u       uaac

mmmmgmmxmmmmmggmmmgxmmmmmgm
```

sequence

```
((((.((((((((...((((..(((((((((.(((((((((((.(((((((..........))))))).))))))))))).)))))))))..)))).
.))....)))))))))
acaugccaaugacccggcccccacuuuuaaauucuguugcagcagauagcugauacccaaugu*uaucuuuugcggcagaaauugaa*agugcuggccau
aucuacaauugggugu
```

folding energy of structure: **-46.1** kcal/mol

**id**	33383
**genomic position**	68761-68887 on AJ507799 (-) in Epstein Barr virus
**L score**	1.0

structure

```
       g  a      a  c    -         ca    cu  uu  a gag   a--- g  u
ggaggcc ag cagaag ca aggc ccccaggga  ggug  gc  gc c   aac    u gu g
||||||| || |||||| || |||| |||||||||  ||||  ||  || |   |||    | || u
ccuccgg uc gucuuc gu uucg gggguucuu  ucac  cg  cg g   uug    a ua u
       -  c      -  c    u         --    uu  uc  - aaa   gggg g  a

mmmmmmmbmmxmmmmmmbmmxmgmmBmmmmmgmgmbbgmmmxxmmxxmmbmxxxmmmxBBBmxgm
```

sequence

```
(((((((.((.((((((.((.(((((((((((((..((((..((..((.(...(((.(.((.....)).)....)))...)))..))..)))))))))
)))).)))).)))))))).)))))))))
ggaggccgagacagaagacacaggcccccagggacaggugcugcuugcacgagaacaugguguuaaaugagggggguuaaaggccugcuucacuuucuu
ggggugcuucugcuucugccuggccucc
```

folding energy of structure: **-57.0** kcal/mol

**id**	30260
**genomic position**	87662-87740 on AJ507799 (+) in Epstein Barr virus
**L score**	1.0

structure

```
  u      -   -        u   auaga    a  gau
ug guugug uac gacgauga guc     ggug uu   g
|| |||||| ||| |||||||| |||     |||| ||
ac cgacac gug cugcuacu uag     ccac aa   u
  -      u   a        -   aag--    c  agu

mmbmgmmmmBgmmBmmmmmmmmbgmmxxxbbmmmmxmm
```

sequence

```
((.((((((((((((((((.(((.....((((.((........)).))))...))))))))))).))).))))))))
uguguuguguacgacgaugaugucauagaggugauugauguugaaaccaccgaagauucaucgucagugucacagcca
```

folding energy of structure: **-27.8** kcal/mol

**id**	33093
**genomic position**	103515-103588 on AJ507799 (-) in Epstein Barr virus
**L score**	1.0

structure

```
 u     a        c   -   c  cacg     gc
c ccggu gagcucgu gau gau uc    augcc  c
| ||||| |||||||| ||| ||| ||    |||||
g ggcca cuugagca cug cua ag    ugcgg  u
 u     c        -   u   c  a---     ga

mxmmmmmxmmgmmmmmbmmgBmmmxmmxbbbmgmmm
```

sequence

```
(.(((((.((((((((.((((((.((....(((((......))))).)).))).))))))))))).)))))).)
cuccgguagagcucgucgaugaucuccacgaugccuaggguggcguagacaucugucacgaguuccaccggug
```

folding energy of structure: **-32.5** kcal/mol

**id**	32868
**genomic position**	134005-134065 on AJ507799 (-) in Epstein Barr virus
**L score**	1.0
**structure**	`   -        g g    u   g   g  u`<br>`gaa ugugccgg u gaua gcc ccu uc c`<br>`||| |||||||| | |||| ||| ||| ||`<br>`cuu gcgcgguc a uuau cgg gga ag g`<br>`   c        - g    -   -   g  g`<br><br>mmmBgmgmmmgmbmxgmmmbmmmbmmmxmm
**sequence**	((((((((((.(.((((.(((.(((.((....)).))))))))).))))))))).)))<br>gaaugugccggguggauaugccgccugucgguugagaggggcuauugacuggcgcgcuuc<br><br>folding energy of structure: **-27.1** kcal/mol

**id**	29668
**genomic position**	137868-137939 on AJ507799 (+) in Epstein Barr virus
**L score**	1.0
**structure**	`   caau          c  a-    a       u`<br>`cug    uucucagugu ac  uugc cguguag a`<br>`|||    |||||||||| ||  |||| |||||||`<br>`gac    aggggguuaca ug  aacg guacguc a`<br>`   uauu          c  gg    c       c`<br><br>mmmxxxxmgmgmmgmmmxmmxBmmmmxmgmmgmm
**sequence**	(((....((((((((((.((.((((.(((((((....))))))).))))..)).))))))))))....)))<br>cugcaauuucucagugucacauugcacguguaguaaccugcaugcgcaagggucacauuggggauuaucag<br><br>folding energy of structure: **-28.7** kcal/mol

**id**	28731
**genomic position**	139064-139171 on AJ507799 (+) in Epstein Barr virus
**L score**	1.0

structure

Candidate miRNA precursor

```
       u--   ----         ga    a   -      u       uaaau
ggcauug   uaa    ccuuuggug  accu gug uuagug ugugcug     a
|||||||   |||    |||||||||  |||| ||| |||||| |||||||
ccgugac   guu    ggaggccac  ugga cac gaucac acgcgac     a
       ucc   cauc         ug    c   u      c       cugug
```

mmmmgmmxBBgmmBBBBmmmggmmmmxxmmmmxmmmBgmmmmmxmmgmmmm

Known miRNA precursor (MI0003725)

```
         ga    a   -      u       uaaau
ccuuuggug  accu gug uuagug ugugcug     a
|||||||||  |||| ||| |||||| |||||||
ggaggccac  ugga cac gaucac acgcgac     a
         ug    c   u      c       cugug
```

mmmggmmmmxxmmmmxmmmBgmmmmmxmmgmmmm

sequence

```
(((((((.((((((((((((..((((.(((((((((.(((((((............))))))).)))))).))).))))..)))))))))....)))...)))))))
ggcauuguuaaccuuuggugg*aaccuaguguuaguguugugcu*guaaauaaguguccag*cgcaccacuagucaccaggugu*caccggaggcuacuugccucagugcc
```

folding energy of structure: **-48.7** kcal/mol

**id**	30009
**genomic position**	139207-139298 on AJ507799 (+) in Epstein Barr virus
**L score**	1.0

structure

Candidate miRNA precursor

```
       ua-          gg-  c     c      u ug  gu
ggcucug   acauuuggug   ac ugaug ugcugg g  cu  a
|||||||   ||||||||||   || ||||| |||||| |  ||  a
ucgggac   uguggaccac   ug acuac acgauc c  ga  a
       cac          gga  c     -      - gu  au
```

gmmgmmmxxBmmmggmmmmmxxBmmxmmmmmbmmmmgmbmxxmm

Known miRNA precursor (MI0003726)

```
       g     a   -   -         --   aa
uuggugg accug ugc ugc uggugugcu   gua  u
||||||| ||||| ||| ||| |||||||||   |||
gaccacu uggac acg aug acuacacga   cgu  a
       g     c   g   c         uc   ga
```

gmmmmmgxmmmmmxmmmBmgmBmmgmmmmmmBBmgm

sequence

```
(((((((..((((((((((..((.(((((.((((((.(..((.......))..))))))))))))).))...))))))))))...)))))))
ggcucuguaacauuuggugg*gaccugaugcugcuggugugcu*guaaauaagugccuagcacaucacguaggcaccaggugucaccagggcu
```

folding energy of structure: **-44.3** kcal/mol

**id**	28941
**genomic position**	139213-139300 on AJ507799 (+) in Epstein Barr virus
**L score**	1.0

structure

Candidate miRNA precursor

```
   aca        g     a   -   -         --   aa
gua   uuuggugg accug ugc ugc uggugugcu  gua  u
|||   |||||||| ||||| ||| ||| |||||||||  |||
cau   ggaccacu uggac acg aug acuacacga  cgu  a
   cg-        g     c   g   c         uc   ga

mmmxxbggmmmmmgxmmmmmxmmmBmgmBmmgmmmmmmBBmgm
```

Known miRNA precursor (MI0003726)

```
       g     a   -   -         --   aa
uuggugg accug ugc ugc uggugugcu  gua  u
||||||| ||||| ||| ||| |||||||||  |||
gaccacu uggac acg aug acuacacga  cgu  a
       g     c   g   c         uc   ga

gmmmmmgxmmmmmxmmmBmgmBmmgmmmmmmBBmgm
```

sequence

```
(((...((((((((.(((((.((((((((((((((((((......)))..))))))))).))).))).))))).))))))))..)))
guaacauuuggugggaccugaugcugcuggugugcuguaaauaagugccuagcacaucacguaggcaccaggugucaccagggcuac
```

folding energy of structure: **-39.8** kcal/mol

**id**	29081
**genomic position**	139506-139584 on AJ507799 (+) in Epstein Barr virus
**L score**	1.0

structure

Candidate miRNA precursor

```
    c g  ugg         u      c   ag   u
ugug c cu   agggaaaca gaccac uga  ucug u
|||| | ||   ||||||||| |||||| |||  |||| a
acac g ga   uuccuuugu uuggug acu  ggac a
    a a  uag         u      -   --   c

mmmmxmxmmxxxmgmmmmmmmxgmmmmmbmmmbbgmmm
```

Known miRNA precursor (MI0004988)

```
   c g  ugg         u      c   ag   u
gug c cu   agggaaaca gaccac uga  ucug u
||| | ||   ||||||||| |||||| |||  |||| a
cac g ga   uuccuuugu uuggug acu  ggac a
   a a  uag         u      -   --   c

mmmxmxmmxxxmgmmmmmmmxgmmmmmbmmmbbgmmm
```

sequence

```
((((.(.((...(((((((((.((((((.(((..((((.....))))))))))))).)))))))))...)).).))))
ugugccgcuuggagggaaacaugaccaccugaagucuguuaaccaggucagugguuuuguuccuugauagagacaca
```

folding energy of structure: **-29.1** kcal/mol

**id**	28641
**genomic position**	139672-139738 on AJ507799 (+) in Epstein Barr virus
**L score**	1.0

structure

Candidate miRNA precursor

```
  ca      au     u        ac  a  g
cu  agguga  auagc gcccaucg  gu uc c
||  ||||||  ||||| ||||||||  || ||
ga  uccacu  ugucg cggguggc  ca ag u
  aa      --     c        --  a  g
```

mmxxmmmmmmbbmgmmmxmmmmmgmmbbmmxmm

Known miRNA precursor (MI0003727)

```
  uc    g   cuca      au      u        ac  a  g
gc  ugug cac    agguga  auagc gcccaucg  gu uc c
||  |||| |||    ||||||  ||||| ||||||||  || ||
ug  acgc gug    uccacu  ugucg cggguggc  ca ag u
  ga    a   aaa-      --      c        --  a  g
```

gmxxmmgmxmmmxxxbmmmmmmbbmgmmmxmmmmmgmmbbmmxmm

sequence

((..((((((..(((((.((((((((..((.((....)).)))))))))).)))))))))))..))

c*ucaaggugaauauagcugcccaucg*acguaucgcuggaaaccgguggccgcuguucaccuaaag

folding energy of structure: **-29.8** kcal/mol

**id**	31765
**genomic position**	139776-139873 on AJ507799 (-) in Epstein Barr virus
**L score**	1.0

structure

```
     a   au       a        --     -   a     guaa
gguuu uca  ugugggau uggauaga  gggug gug ucuug    u
||||| |||  |||||||| ||||||||  ||||| ||| |||||    u
ccgaa agu  acaccuua aucuaucu  cccac cac agaac    a
     -   cc       a        ca     a   g     acaa
```

mmgmmbmmmxxmmmmmgmmxmgmmmmmmBBmmmmmBmmmxmmmmm

sequence

(((((.(((..((((((((.((((((((((((((((.(((((..........))))).))).)))))..)))))))).))))))))..))))))))

gguuuaucaauugugggauauggauagagggugguga ucuugguguaauuaaacaagagcacacacccacucuaucuaaauuccacaccugaaagcc

folding energy of structure: **-44.5** kcal/mol

id	27827
genomic position	139893-139995 on AJ507799 (+) in Epstein Barr virus
L score	1.0
structure	**Candidate miRNA precursor**<br>`    aac   a-    gca  -     a      -            uuauu `<br>`guug   agg  ugug   cc cuaag ggacgc aggcauacaagg     a`<br>`||||   |||  ||||   || ||||| |||||| ||||||||||||      `<br>`cgau   ucc  acgc   gg gauuc ccugug uccguauguucc     c`<br>`    gga   gg    ag-  u     c      g            ugacc `<br><br>mgmgxxxmmmxBmmgmxxbmmBmmmmmxmmmmgmBmmmmmmmmmmmm
	**Known miRNA precursor (MI0004990)**<br>`    aac   a-    gca  -     a      -            uuauu `<br>`guug   agg  ugug   cc cuaag ggacgc aggcauacaagg     a`<br>`||||   |||  ||||   || ||||| |||||| ||||||||||||      `<br>`cgau   ucc  acgc   gg gauuc ccugug uccguauguucc     c`<br>`    gga   gg    ag-  u     c      g            ugacc `<br><br>mgmgxxxmmmxBmmgmxxbmmBmmmmmxmmmmgmBmmmmmmmmmmmm
sequence	((((...(((.((((...((((((.((((((((((((((((((............)))))))))))).)))))).))))).))..)))).)))...))))<br>guugaacaggauguggcacccu*aagaggacgcaggcauacaagg*uuauuacccaguccu*uguaugccuggugucccccuua*gugggacgcaggccuagguagc<br><br>folding energy of structure: **-49.6** kcal/mol

<table>
<tr><th>id</th><td>33319</td></tr>
<tr><th>genomic position</th><td>139899-139989 on AJ507799 (-) in Epstein Barr virus</td></tr>
<tr><th>L score</th><td>1.0</td></tr>
<tr><th>structure</th><td><pre>
    cc- c uc a   ac   ggacu
uagg   ug g cc cuaaggggac caggcauacaagg   g
|||| || | || |||||||||| |||||||||||||
gucc   ac c gg gauucuccug guccguauguucc   g
    uac - gu -   c-   uaaug
</pre>
gmmmxxBmmbmxxmmbmmmmmgmmmmxbmmmmmmmmmmmmm</td></tr>
<tr><th>sequence</th><td>((((..((.(..((.((((((((((..(((((((((((((...........))))))))))))).))))))))))))..)))...))))
uaggccugcgucccacuaaggggacaccaggcauacaaggacuggguaauaaccuuguaugccugcguccucuuagggugccacauccug

folding energy of structure: -45.3 kcal/mol</td></tr>
</table>

<table>
<tr><th>id</th><td>29664</td></tr>
<tr><th>genomic position</th><td>140360-140441 on AJ507799 (+) in Epstein Barr virus</td></tr>
<tr><th>L score</th><td>1.0</td></tr>
<tr><th>structure</th><td><pre>
     --   aug   cu   cg   a   gu
gaccu gcu   ugg aga uauggccuaccc agac u
||||| ||| ||| ||| |||||||||||| |||| g
uugga cgg   acc ucu guaccggauggg ucug g
     ua   ---   u-   ua   c   gg
</pre>
gmmmmBBmmgbbbmmmxbmmmxxgmmmmmmmmmmmxmmmm</td></tr>
<tr><th>sequence</th><td>((((((((...(((..(((..((((((((((((.((((.......)))).))))))))))))..))).))))))..)))))
gaccugcuauguggcuagacguauggccuacccaagacguuggggucucggguaggccaugauucuuccaggcauagguu

folding energy of structure: -39.7 kcal/mol</td></tr>
</table>

<table>
<tr><th>id</th><td>28561</td></tr>
<tr><th>genomic position</th><td>145483-145602 on AJ507799 (+) in Epstein Barr virus</td></tr>
<tr><th>L score</th><td>1.0</td></tr>
<tr><th>structure</th><td><pre>
         a---   ua- a   -   uu   aag   a   agu
cuuuguguu   ggu   gu uggg cugggua cacuagug   gcaacua cac   u
||||||||| ||| || |||| ||||||| |||||||| ||||||| |||
gggacacaa   cca   ua accc ggccuau guggucac   uguugau gug   a
         gaca   cug a   u   uu   ccg   c   cag

mggmmmmmmxBBBmmmxxBgmxmmmmBmgmmgmmxxmmmgmmmmxxxgmmmmmmxmmm
</pre></td></tr>
<tr><th>sequence</th><td><pre>
(((((((((.(((..((.(((((((((((..((((((((...(((((((.(((........))).)))))))...))))))))..))))))).)))
).))...)))....)))))))))
cuuuguguuagguuaguaugggcuggguauucacuagugaaggcaacuaacacaguuagacgugcuaguugugcccacugguguuuauccggucccc
aaaugucaccacagaacacaggg
</pre>
folding energy of structure: -53.0 kcal/mol</td></tr>
</table>

<table>
<tr><th>id</th><td>30018</td></tr>
<tr><th>genomic position</th><td>145493-145589 on AJ507799 (+) in Epstein Barr virus</td></tr>
<tr><th>L score</th><td>1.0</td></tr>
<tr><th>structure</th><td><pre>
   ua- a   -   uu   aag   a   agu
ggu   gu uggg cugggua cacuagug   gcaacua cac   u
||| || |||| ||||||| |||||||| ||||||| |||
cca   ua accc ggccuau guggucac   uguugau gug   a
   cug a   u   uu   ccg   c   cag

mmmxxBgmxmmmmBmgmmgmmxxmmmgmmmmxxxgmmmmmmxmmm
</pre></td></tr>
<tr><th>sequence</th><td><pre>
(((..((.(((((((((((..((((((((...(((((((.(((........))).)))))))...))))))))..))))))).)))).))...)))
gguuaguaugggcuggguauucacuagugaaggcaacuaacacaguuagacgugcuaguugugcccacugguguuuauccggucccaaaugucacc
</pre>
folding energy of structure: -43.2 kcal/mol</td></tr>
</table>

**id**	29857
**genomic position**	147154-147236 on AJ507799 (+) in Epstein Barr virus
**L score**	1.0

structure

```
    uu       g         c    ag    -    -  u
gcgg  gucacag ugcuagacc ugg  uug aacc ag a
||||  ||||||| ||||||||| |||  ||| |||| || c
cguc  caguguu augaucugg acu  aac uugg uc c
    c-       g         u    ga    a    c  a

mmgmxbmmmmmmgxmgmmmmmmmxmmgxxmmmBmmmmBmm
```

sequence

```
((((..(((((((.(((((((((.(((..(((((((((.....)).)))).)))..))).))))))))).))))))).))))
gcgguugucacaggugcuagacccuggaguugaaccaguaccacucgguuacaaagucauggucuaguaguugugacccugc
```

folding energy of structure: **-40.5** kcal/mol

**id**	29968
**genomic position**	147523-147609 on AJ507799 (+) in Epstein Barr virus
**L score**	1.0

structure

Candidate miRNA precursor

```
    u--        u   a          c  ua     g
ggcu   cuguuggg cag caguuuggug gc  guugu ugcu
||||   |||||||| ||| |||||||||| ||  ||||| ||| u
ccgg   gacgauuc guc gucggaccac cg  caacg acga
    ugu        c   a          a  --     -

mmmgxBBmmmgmggmxmmmxmmmggmmmmmxmmbbmmmmgbmmm
```

Known miRNA precursor (MI0003733)

```
    u--        u   a          c  ua     g
ggcu   cuguuggg cag caguuuggug gc  guugu ugcu
||||   |||||||| ||| |||||||||| ||  ||||| ||| u
ccgg   gacgauuc guc gucggaccac cg  caacg acga
    ugu        c   a          a  --     -

mmmgxBBmmmgmggmxmmmxmmmggmmmmmxmmbbmmmmgbmmm
```

sequence

((((.((((((((.(((.((((((((((.((..(((((.(((...)))))))))).)))))))))).))).))))))))...))))

ggcuucuguuggguca*gacaguuuggugcgcuaguugu*gugcuuagcagca*acgcacaccaggcugacugcc*uuagcagugugcc

folding energy of structure: **-43.5** kcal/mol

**id**	29929
**genomic position**	148327-148405 on AJ507799 (+) in Epstein Barr virus
**L score**	1.0

structure

Candidate miRNA precursor

```
   -   u  u         a-         u  ug-
agg gcc au guagcaggc  ugucuucau cc   cgu
||| ||| || |||||||||  ||||||||| ||   || a
ucc cgg ua cauuguccg  acggaagua gg   gcc
   a   u  c         ac         c  uaa

mmmBmmmxmmxmmmgmmmmmxBmmgmmmmmmxmmxxBmm
```

Known miRNA precursor (MI0004993)

```
    -  cgu--   -   u  u         a-         u  ug-
uaca gg     agg gcc au guagcaggc  ugucuucau cc   cgu
|||| ||     ||| ||| || |||||||||  ||||||||| ||   || a
augu cc     ucc cgg ua cauuguccg  acggaagua gg   gcc
    a  uuuuu   a   u  c         ac         c  uaa

mmmmBmmxxxBBmmmBmmmxmmxmmmgmmmmmxBmmgmmmmmmxmmxxBmm
```

sequence

```
((((((.((.(((((((((.((((((((.((..((...))...)).)))))))))..)))))))).)).))).)))
agggccuauuguagcaggcaugucuucauuccugcguaccgaaugggcaugaaggcacagccuguuaccauuggcaccu
```

folding energy of structure: **-37.7** kcal/mol

**id**	32018
**genomic position**	150844-150927 on AJ507799 (-) in Epstein Barr virus
**L score**	1.0

structure

```
      -   c--    g a a  g         -   -  a
gcgcgg ccu   ggcug g c cg gacgugguc ggc ug g
|||||| |||   ||||| | | || ||||||||| ||| ||
cgugcc ggg   ccgac c g gc cuguaccgg ccg ac g
      u   ccu    g a a  a         u   g  c

mmgmmmBmmgxBBmmmmmxmxmxmmxmmmgmmmgmBmmmBmm
```

sequence

```
((((((((.(((((.(.(.((.(((((((((((((((....)).))).))))))))).)).).).)))))...))).))))))
gcgcggccucggcugggacacgggacguggucggcuggcaccaggccuggccaugucacgagacgcagccuccggguccgugc
```

folding energy of structure: **-47.8** kcal/mol
