# Supplementary material for: In silico miRNA prediction in metazoan genomes: balancing between sensitivity and specificity
Source: BMC Genomics. 2009 Apr 30;10:204. doi: 10.1186/1471-2164-10-204 (PMC2688010; doi:10.1186/1471-2164-10-204)
Supplement: Additional file 6 — Hairpins identified in Mareks disease Virus. Details of 18 hairpins with L >= 0.30 identified in Mareks Disease Virus [EMBL: AF243438] for the scoring model Metazoa. [file 1471-2164-10-204-S6.pdf]

Additional File 6: Identified hairpins in Mareks Disease Virus with *L* >= 0.30

List of 18 identified hairpins in Mareks Disease Virus [EMBL:AF243438] with *L* >= 0.30, of which eigth are known miRNAs (denoted with !! when present in miRBase version 9.0, denoted with [Yao ea 2008] when described in this publication.). These 18 hairpins collapse on 13 unique loci; candidates 25615, 22600 and 24833 are alternative structures for candidates 26918, 26073 and 26711, candidates 26788 and 57118 are on the opposite strand of candidates 27073 and 26711. *L* >= 0.30 corresponds to the 1% best scoring identified hairpins on MDV (53 candidates). These 53 are further limited for non-overlap with annotated exons (17) and redundant hairpins in an ~15kb long (exactly) inverted repeated region (18). Between brackets the number of candidates that were removed by the filtering step.

In the publication three hairpins are mentioned: one located in the transcribed strand of an intron (hairpin 25899 / locus 2) and two others closely flanked (0.3 kb) the mdv1-mir-M1 gene in the same orientation (hairpin 25847 / locus 7 and hairpin 26298 / locus 9).

Not all S scores for the 18 descriptors are 1.0, resulting in combined *L* scores smaller than 1.0. Descriptors with S scores < 1.0 are recognizable by a lighter green or yellow color and their colored fields lack a border. The 18 descriptors, from left to right: MFEahl, MFEahl index, Q, max match count, bulgeRatio, GU-match contribution, largest bulge, longest match-stretch, looplength, stem length, dP, SCS-mono, SCS-di, polyA, polyU, polyNucHairpin, GsurplusC, GasurplusCU.

‘Genpos’ denotes the genomic position of the hairpin:

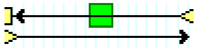 intergenic or intronic

Id	Position	Strand	Locus	Lscore	Genpos
26084	128661..128719	-	1	0.495	
25899	130360..130434	+	2	0.832	
25615	133864..133975 [Yao ea 2008]	+	3	0.526	
26918	133868..133968 [Yao ea 2008]	+	3	1.000	
22600	134338..134455 !!	+	4	0.809	
26073	134355..134436 !!	+	4	1.000	
26788	134400..134461	-	4	0.495	
26433	136037..136128 [Yao ea 2008]	+	5	1.000	
26824	136102..136188	+	6	0.482	
25847	136532..136614	+	7	0.500	
22133	136855..136947 !!	+	8	1.000	
26298	137115..137192	+	9	0.781	
25737	142172..142315 !!	+	10	0.315	
22011	142270..142344 [Yao ea 2008]	+	11	0.357	
24833	142481..142591 !!	+	12	0.537	
26711	142495..142579 !!	+	12	0.537	
27118	142505..142571	-	12	0.339	
24751	142609..142706 [Yao ea 2008]	+	13	1.000	

**Id**	26084
**genomic_position**	128660-128719 on AF243438 (-) in Mareks disease virus
**default_lscore**	0.49472342099
**structure**	** uu a g- u - u**<br>**gaug g cggu guc g cggucccgg c**<br>**|||| | |||| ||| | |||||||||**<br>**cuac c gcca cag c gucagggcu g**<br>** uu - ag c a u**<br><br>mmmmxxmbmmmmxBmmmxmBmgmmmmmmg
**sequence**	((((..(.((((.(((.((((((((((....))))))))).).)))..)))))..))))<br>gauguugacgguggucugcggucccggaucgucgggacugaccgacgaaccgcuucauc<br><br>folding energy of structure: **-28.0** kcal/mol

**id**	25899
**genomic_position**	130359-130434 on AF243438 (+) in Mareks disease virus
**default_lscore**	0.831529838222
**structure**	** aa a a uc-- u**<br>**agg gcggggga ggggu uguu augagacag a**<br>**||| |||||||| ||||| |||| ||||||||| g**<br>**uuc ugcucccu ccuca acag uacucuguu a**<br>** gg - - cuac u**<br><br>mgmxxgmmgmmmmbmmgmmbmmmgxxBBmmmmmmmmg
**sequence**	(((..((((((((.(((((.((((..(((((((((.....)))))))))....)))))))))))))))))..)))<br>aggaagcgggggaagggguauguuucaugagacaguagauuugucucaucaucgacaacuccucccucguggcuu<br><br>folding energy of structure: **-36.2** kcal/mol

id: 25615

genomic_position: 133863-133975 on AF243438 (+) in Mareks disease virus

default_lscore: 0.526422856804

structure:

```
   a--- ag- g   aau   -    cc     ag           a        g c
ugg    g   g gau   gcc cucg  gauca  gcccuccguau auguaaau u c
|||    |   | |||   ||| ||||  |||||  ||||||||||| |||||||| |
acc    c   u cua   cgg gagc  cuagu  ugggaggcaua uacguuug a a
   ggag gca g   gc-   u    --     cu           a        g a

mmmxBBBmxxBgxmmmxxbmmmBmmmmbbmmmmmxxgmmmmmmmmmmxmmmgmmmgxm
```

sequence:

```
(((.(..(.(((...(((((((..(((((..(((((((((((.((((((((.(....).)))))))).)))))))))))..))))))))).)))..))).)
...)....)))
uggagaggggauaaugcccucgccgaucaaggcccuccguauaauguaaauguccaaagguuugcauaauacggaggguucugauccgaguggccgaucgu
acgcgaggcca
```

folding energy of structure: **-51.3** kcal/mol

id: 26918

genomic_position: 133867-133968 on AF243438 (+) in Mareks disease virus

default_lscore: 1.0

structure:

```
 ag- g   aau   -    cc     ag           a        g c
g   g gau   gcc cucg  gauca  gcccuccguau auguaaau u c
|   | |||   ||| ||||  |||||  ||||||||||| |||||||| |
c   u cua   cgg gagc  cuagu  ugggaggcaua uacguuug a a
 gca g   gc-   u    --     cu           a        g a

mxxBgxmmmxxbmmmBmmmmbbmmmmmxxgmmmmmmmmmmxmmmgmmmgxm
```

sequence:

```
(..(.(((...(((((((..(((((..(((((((((((.((((((((.(....).)))))))).)))))))))))..))))))))).)))..))).)...)
gaggggauaaugcccucgccgaucaaggcccuccguauaauguaaauguccaaagguuugcauaauacggaggguucugauccgaguggccgaucguacgc
```

folding energy of structure: **-48.9** kcal/mol

**id**	22600
**genomic_position**	134337-134455 on AF243438 (+) in Mareks disease virus
**default_lscore**	0.808613808398

structure

Candidate miRNA precursor

```
   gc   g uu   aug--      gu    uuua       ua         c     uc g
ugg  uu u  uga     uccccu  gaag    augcug  ucggaacc uucgu  g u
|||  || |  |||     ||||||  ||||    ||||||  |||||||| |||||  |
acc  aa g  gcu     aggggg  uuuc    uacgac  agucuugg aagca  c g
   aa   g uc   aguaa      uc    cag-       --         u     c- a

mmmxxmmxgxxgmmxxxBBmmmmmgxxgmmmxxxbmmmmmmbbmmgmmmmmxmmmmmxbm
```

Known miRNA precursor (MI0005096)

```
    uuua       ua         c     uc g
gaag    augcug  ucggaacc uucgu  g u
||||    ||||||  |||||||| |||||  |
uuuc    uacgac  agucuugg aagca  c g
    cag-       --         u     c- a

gmmmxxxbmmmmmmbbmmgmmmmmxmmmmmxbm
```

sequence

(((..((.(..(((...((((((..((((....((((((..((((((((.(((((..(....).))))).)))))))))))))))...))))..))))))...
..)))..).))..)))

ugggcuuguuuugaaugucccugugaagu*uuaaugcuguaucggaacccuuc*guucggugaccacgaugguucugacagcaugaccuuucuggggggaaau
gaucgcuggaaaacca

folding energy of structure: **-45.5** kcal/mol

**id**	26073
**genomic_position**	134354-134436 on AF243438 (+) in Mareks disease virus
**default_lscore**	1.0

structure

Candidate miRNA precursor

```
      gu    uuua       ua         c     uc g
uccccu  gaag    augcug  ucggaacc uucgu  g u
||||||  ||||    ||||||  |||||||| |||||  |
aggggg  uuuc    uacgac  agucuugg aagca  c g
      uc    cag-       --         u     c- a

mmmmmgxxgmmmxxxbmmmmmmbbmmgmmmmmxmmmmmxbm
```

Known miRNA precursor (MI0005096)

```
    uuua       ua         c     uc g
gaag    augcug  ucggaacc uucgu  g u
||||    ||||||  |||||||| |||||  |
uuuc    uacgac  agucuugg aagca  c g
    cag-       --         u     c- a

gmmmxxxbmmmmmmbbmmgmmmmmxmmmmmxbm
```

sequence

((((((..((((....((((((..((((((((.(((((..(....).))))).)))))))))))))))...))))..))))))
uccccugugaagu*uuaaugcuguaucggaacccuuc*guucggugaccacgaugguucugacagcaugaccuuucuggggga

	folding energy of structure: **-40.1** kcal/mol

**id**	26788
**genomic_position**	134399-134461 on AF243438 (-) in Mareks disease virus
**default_lscore**	0.494855140146

structure

```
      -       c-    --    a    c
cuacga ugguuuu  cagc  gauc uuuc a
|||||| |||||||  ||||  |||| |||| g
ggugcu accaaga  gucg  cugg aaag a
      u       cu    ua    -    a

mgmmmmBmmmmmgmxBmmmmBBmmgmbmmmm
```

sequence

```
((((((((((((.((((((((.((((.....))))))))..))))..))))))).))))))
cuacgaugguuuuccagcgaucauuucccccagaaaggucaugcugucagaaccauucgugg
```

folding energy of structure: **-23.1** kcal/mol

**id**	26433
**genomic_position**	136036-136128 on AF243438 (+) in Mareks disease virus
**default_lscore**	1.0

structure

```
 cu       -      u uu   u               c----  g
c  cuggggu aacaaa u  ccu accguguagcuuaga     uc g
|  ||||||| |||||| |  ||| |||||||||||||||     ||
g  gaccucg uuguuu a  gga ugguacauugaguuu     ag a
 ag       g      - gg   c               uauca  a

mxxmmmmgmgBmmmmmmbmxxmmmxmmmgmmmmgmmgmgmxBBBBmm
```

sequence

```
(..(((((((((((((.(..(((.(((((((((((((((.((....)).....))))))))))))))).)))..))))))).)))))))..)
ccucuggggguaacaaauuuuccuuaccguguagcuuagacucggaagaacuauuuugaguuacauggucaggggauuuguuggcuccaggag
```

folding energy of structure: **-44.1** kcal/mol

**id**	26824
**genomic_position**	136101-136188 on AF243438 (+) in Mareks disease virus
**default_lscore**	0.481564753951

structure

```
    ag    uu     g          --    a--   a    a
gguc  ggga  uguug cuccaggagu  uccg   agu ugag u
||||  ||||  ||||| ||||||||||  ||||   ||| |||| a
ccgg  cucu  acaac ggggucuuca  aggu   ucg auuc a
    cu    --     -          aa    gua   -    a

mmgmxxmgmmbbmmmmmbmgmmmmgmmmBBmmmgxBBmmgbmgmm
```

sequence

```
((((..((((..(((((.(((((((((((((.(((.((((.....)))))))...))))..))))))))))))))))))..))))
ggucaggggauuuguuggcuccaggaguuccgaaguaugagauaaacuuagcuauguggaaaacuucuggggcaacaucucucggcc
```

folding energy of structure: **-37.3** kcal/mol

**id**	25847
**genomic_position**	136531-136614 on AF243438 (+) in Mareks disease virus
**default_lscore**	0.500185258809

structure

```
     uc   ug  gc a      u      -     gugc
guccc  ucc  ug  c gcucac ggcugu gcacu    g
|||||  |||  ||  | |||||| |||||| |||||    a
cgggg  agg  ac  g cgagug cugaca cguga    u
     cu   ua  ua a      -      u     aucu

mgmmmxxmmmxxmmxxmxmmmmmmbmgmmmmBmmmmm
```

sequence

```
(((((..(((..((..(.((((((.(((((((((((..........))))).))))))))))))).)..))..)))..)))))
gucccucuccuguggccagcucacuggcugugcacugugcgauucuaagugcuacagucgugagcagaucaauggaucggggc
```

folding energy of structure: **-39.4** kcal/mol

**id**	22133
**genomic_position**	136854-136947 on AF243438 (+) in Mareks disease virus
**default_lscore**	1.0

structure

Candidate miRNA precursor

```
   a u   -    ccu       g    c              acau
gag c cuc uucc   uuugcuu uuca ugugcggcauuauu    u
||| | ||| ||||   ||||||| |||| ||||||||||||||    u
cuc g gag aagg   aagcgag aagu acgcgucguaaugg    a
   - -   c    c--       a    -              ccac

mmmbmbmmmBmmmmxbbmmgmmmgxmmmmbmmgmmgmmmmmmgg
```

Known miRNA precursor (MI0005093)

```
  u       g    c              acau
cc uuugcuu uuca ugugcggcauuauu    u
|| ||||||| |||| ||||||||||||||    u
gg aagcgag aagu acgcgucguaaugg    a
  c       a    -              ccac

mmxmmgmmmgxmmmmbmmgmmgmmmmmmgg
```

sequence

```
(((.(.(((((((...(((((((.((((.((((((((((((((...........))))))))))))))))).))))))).)))).)))))))
gagacucucuuccccuuuugcuuguucacugugcggcauuauuacauuuacaccgguaaugcugcgcaugaaagagcgaacggaacgaggcuc
```

folding energy of structure: **-38.3** kcal/mol

**id**	26298
**genomic_position**	137114-137192 on AF243438 (+) in Mareks disease virus
**default_lscore**	0.78111982283

structure

```
        cu       ug   g       c
cccuuccc  uuuacuu  uuu augugug cacuguugu
||||||||  |||||||  ||| ||||||| ||||||||  a
gggaaggg  aagugag  aaa ugcgcgc gugauagcc
        c-       --   g       c

mmmmmmmmxbmmgmmmgbbmmmxmgmgmgmxmmmmgmgm
```

sequence

```
((((((((..((((((((..(((.(((((((.((((((((...)))))))).))))))).))))))))))).))))))))
cccuuccccuuuuacuuuguuugaugugugccacuguuguaccgauagugccgcgcgugaaagagugaacgggaaggg
```

folding energy of structure: **-38.8** kcal/mol

**id**	25737
**genomic_position**	142171-142315 on AF243438 (+) in Mareks disease virus
**default_lscore**	0.315352525584

structure

Candidate miRNA precursor

```
   gauac   a     uuu   u  caua     ac     c              uu    u  aucuau  u
ugu     gcg ucgag   ucg gg    uuccu  ggaaa cuauuguucugugg  gguu  cg     cg u
|||     ||| |||||   ||| ||    |||||  ||||| ||||||||||||||  ||||  ||     ||
acg     cgu agcuu   ggc cc    gagga  ccuuu gauaacaaggcauc  ccag  gc     gc c
   aagc-   c     u--   -  uuua     --     u              u-    u  gucau-  u
```

mmgxxxxbmmgxmmmmgxbbgmmbmmxxxxgmmmmbbmmmmmxmmmmmmmmmmgmmgmxbmmmgxmmxxxxxbmm

Known miRNA precursor (MI0005100)

```
     ac     c              uu    u  aucuau  u
uuccu  ggaaa cuauuguucugugg  gguu  cg     cg u
|||||  ||||| ||||||||||||||  ||||  ||     ||
gagga  ccuuu gauaacaaggcauc  ccag  gc     gc c
     --     u              u-    u  gucau-  u
```

gmmmmbbmmmmmxmmmmmmmmmmgmmgmxbmmmgxmmxxxxxbmm

sequence

(((.....(((.(((((...(((.((....(((((..(((((.((((((((((((((..((((.((......((....)).....)).)))).)))))))))
))))).))))))))))....))))).))))).)))....)))

ugugauacgcgaucgaguuuucguggcauauuccuacggaaaccuauuguucugugguugguuucgaucuaucguucucguacugc*gugaccucuacggaac aauagu*uuuccaggagauuucccgguuucgacugccgaagca

folding energy of structure: **-50.8** kcal/mol

**id**	22011
**genomic_position**	142269-142344 on AF243438 (+) in Mareks disease virus
**default_lscore**	0.357330031926

structure

```
         --          ga     cg      a
gaacaauag  uuuuccagga  uuucc  guuucg c
|||||||||  ||||||||||  |||||  |||||| u
cuuguuguc  aaaggguccu  aaagg  cgaagc g
         ua          gc     ua      c

mmmmmmgmmBBmmmgmmmmmmxxmmmmmxxmgmmmm
```

sequence

((((((((((((((((((..(((((..((((((.....))))))..)))))..))))))))))..)))))))))
gaacaauaguuuuccaggagauuucccgguuucgacugccgaagcauggaaacguccugggaaaaucuguuguuc

folding energy of structure: **-36.6** kcal/mol

**id**	24833
**genomic_position**	142480-142591 on AF243438 (+) in Mareks disease virus
**default_lscore**	0.53734455228

structure

Candidate miRNA precursor

```
   a c    aacg    aag         u      gg      c   ucu-  cc
gag g cgcg    cucc   gagaacugu aucucg  gagauc cga    cu  u
||| | ||||    ||||   ||||||||| ||||||  |||||| |||    ||
cuc c gugu    gggg   uuuuugaca uagagc  cucuag gcu    ga  a
   - u    aaa-    g--         u      au      a   caac  cc

mmmbmxmgmgxxxbmgmmxbbgmgmmmmmmxmmmmmmxxmmmmmmxmmmxxxBmm
```

Known miRNA precursor (MI0005099)

```
         u      gg      c   ucu-  cc
gagaacugu aucucg  gagauc cga    cu  u
||||||||| ||||||  |||||| |||    ||
uuuuugaca uagagc  cucuag gcu    ga  a
         u      au      a   caac  cc

gmgmmmmmmxmmmmmmxxmmmmmmxmmmxxxBmm
```

sequence

(((.(.((((....((((...(((((((((.((((((..((((((.(((...((......))....))).))))))..)))))).))))))))).)))).
..)))).))))
gagagccgcgaacgcuccaaggagaacuguuaucucggggagaucccgaucucuccuaccagcaac*ucgagaucucuacgagauuacag*uuuuuggggga
aauguguccuc

folding energy of structure: **-42.4** kcal/mol

**id**	26711
**genomic_position**	142494-142579 on AF243438 (+) in Mareks disease virus
**default_lscore**	0.53734455228

structure

Candidate miRNA precursor

```
    aag         u      gg      c   ucu-  cc
cucc   gagaacugu aucucg  gagauc cga    cu  u
||||   ||||||||| ||||||  |||||| |||    ||
gggg   uuuuugaca uagagc  cucuag gcu    ga  a
    g--         u      au      a   caac  cc

mgmmxbbgmgmmmmmmxmmmmmmxxmmmmmmxmmmxxxBmm
```

Known miRNA precursor (MI0005099)

```
         u      gg      c   ucu-  cc
gagaacugu aucucg  gagauc cga    cu  u
||||||||| ||||||  |||||| |||    ||
uuuuugaca uagagc  cucuag gcu    ga  a
         u      au      a   caac  cc

gmgmmmmmmxmmmmmmxxmmmmmmxmmmxxxBmm
```

sequence

```
((((...(((((((((.((((((..((((((.(((...((......))....))).))))))..)))))).))))))))).))))
cuccaaggagaacuguuaucucggggagaucccgaucucuccuaccagcaacucgagaucucuacgagauuacaguuuuugggg
```

folding energy of structure: **-35.8** kcal/mol

**id**	27118
**genomic_position**	142504-142571 on AF243438 (-) in Mareks disease virus
**default_lscore**	0.339291811307

structure

```
      a      ua          guug  ug
aacugu aucucg  gagaucucga    cu  g
|||||| ||||||  ||||||||||    ||
uugaca uagagc  cucuagggcu    ga  u
      a      cc          aga-  ga

mmmmmmxmmmmmmxxmmmmmmgmmmxxxbmm
```

sequence

```
((((((.((((((..((((((((((....((......))...))))))))))..)))))).))))))
aacuguaaucucguagagaucucgaguugcugguaggagagaucgggaucuccccgagauaacaguu
```

folding energy of structure: **-32.7** kcal/mol

**id**	24751
**genomic_position**	142608-142706 on AF243438 (+) in Mareks disease virus
**default_lscore**	1.0

structure

```
    c       - gg  ggc                cca  ucucu  u
gaag uuccuag u  au   guugucucguagaggu   ga     cc g
|||| ||||||| |  ||   ||||||||||||||||   ||     ||
uuuc aaggauc g  ug   caauagagcaucucua   cu     gg u
    a       u uu  a--                aag  caac-  u

gmmmxmmmmmmmBgxxmgxbbmmmgmmmmmmmmmmmgmxxxmmxxxxbmm
```

sequence

```
((((.((((((((..((...(((((((((((((((...((.....((....))....))...))))))))))))))).))..).))))))).))))
gaagcuuccuaguggauggcguugucucguagagguccagaucucuccuguuggcaacucgaaaucucuacgagauaacaguuugucuaggaaacuuu
```

folding energy of structure: **-38.9** kcal/mol
