# Supplementary material for: In silico miRNA prediction in metazoan genomes: balancing between sensitivity and specificity
Source: BMC Genomics. 2009 Apr 30;10:204. doi: 10.1186/1471-2164-10-204 (PMC2688010; doi:10.1186/1471-2164-10-204)
Supplement: Additional file 7 — Hairpin loci in C. elegans obtained by the filtering protocol "Clustered". Filtering on L score was combined with filtering on genomic context, a protocol referred to as "Clustered". [file 1471-2164-10-204-S7.pdf]

Additional File 7: Hairpin loci in *C. elegans* obtained by the filtering protocol "Clustered"

List of 30 identified hairpins on 20 loci in *C. elegans* with *L* score = 1.0 and less than 5kb up- or downstream from one of the 132 currently known *C. elegans* miRNAs. Further filtering criteria are no overlap with annotated exons, less than 40nt overlap with a by Tandem Repeats Finder (*Benson 1999*) detected repeat (default settings) and removal of the known miRNAs themselves. *L* scores are obtained from the scoring model *Metazoa*.

The 30 remaining genomic hairpins are located on 20 unique loci, as shown in the figure on the next page. Hairpins were grouped into unique loci when the centers of their loops were less than 20 nt apart, regardless of the strand on which the hairpins were located.

For each hairpin, all S scores for the 18 descriptors are 1.0, resulting in a combined *L* score of 1.0. The 18 descriptors, from left to right: MFEahl, MFEahl index, Q, max match count, bulgeRatio, GU-match contribution, largest bulge, longest match-stretch, looplength, stem length, dP, SCS-mono, SCS-di, polyA, polyU, polyNucHairpin, GsurplusC, GasurplusCU.

‘Genpos’ denotes the genomic position of the hairpin:

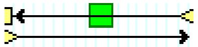 intergenic

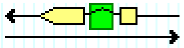 intronic, on the same strand as the exons

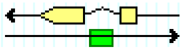 intronic but opposite to the strand of the exons

Id	Position	Strand	Locus#																			Lscore	Genpos
1880443	II 7035046..7035118	+	1																			1.000	
1948311	II 11450711..11450781	+	2																			1.000	
1966903	II 11532611..11532687	+	3																			1.000	
1972296	II 11532611..11532687	-	3																			1.000	
1965421	II 11532912..11532980	+	4																			1.000	
1988720	II 12519798..12520032	+	5																			1.000	
2003357	II 12519798..12520032	-	5																			1.000	
2157900	III 3145225..3145336	+	6																			1.000	
2246908	III 3145241..3145320	-	6																			1.000	
2246732	III 3145992..3146089	-	7																			1.000	
2158072	III 3146692..3146787	+	8																			1.000	
2182845	III 3146699..3146781	+	8																			1.000	
2246304	III 3146704..3146775	-	8																			1.000	
2631469	IV 3258104..3258214	-	9																			1.000	
4356809	IV 3261925..3262012	+	10																			1.000	
4590820	IV 4270555..4270641	-	11																			1.000	
4576692	IV 4272131..4272199	+	12																			1.000	
2915574	IV 14032858..14032960	-	13																			1.000	
4324818	V 5441651..5441724	-	14																			1.000	
3800893	X 2365036..2365147	-	15																			1.000	
3791358	X 2365040..2365143	+	15																			1.000	
3848323	X 3003185..3003251	-	16																			1.000	
3877152	X 5801240..5801380	+	17																			1.000	
3898593	X 5801272..5801349	+	17																			1.000	
4064562	X 11003779..11003882	-	18																			1.000	
4069058	X 11003789..11003876	-	18																			1.000	
4047686	X 11003867..11003974	+	19																			1.000	
4058842	X 11003874..11003965	+	19																			1.000	
4051955	X 11003883..11003985	+	19																			1.000	
4063259	X 11003928..11003992	-	20																			1.000	

<table>
<tr><td>id</td><td>1880443</td></tr>
<tr><td>genomic_position</td><td>7035045-7035118 on CEL150_II (+) in Caenorhabditis elegans</td></tr>
<tr><td>L score</td><td>1.0</td></tr>
<tr><td>structure</td><td><pre>
     g   c gc   -   a
aucag ggugugcggcaaa uu cgaa uuugcug g
||||| ||||||||||||| || |||| |||||||
ugguc cuacacgccguuu ag guuu aaacggc c
     a   - a-   c   u

mgmmmxmgmmmmmmmmmmmmbmgxbmgmmBmmmmmgm
</pre></td></tr>
<tr><td>sequence</td><td><pre>
(((((.(((((((((((((.((..(((((((((((....))))))).)))).))))))))))))))).)))))
aucaggggugugcggcaaacuugccgaauuugcugagcucggcaaacuuugagauuugccgcacaucacuggu
</pre>
folding energy of structure: -37.3 kcal/mol</td></tr>
</table>

<table>
<tr><td>id</td><td>1948311</td></tr>
<tr><td>genomic_position</td><td>11450710-11450781 on CEL150_II (+) in Caenorhabditis elegans</td></tr>
<tr><td>L score</td><td>1.0</td></tr>
<tr><td>structure</td><td><pre>
 g   uga   -   a   g   c
u acg   gcc aaacuuuucag uaccgua ugu g
| ||| ||| ||||||||||| ||||||| ||| c
g ugc   ugg uuugaaaaguc auggcau gca a
 g   ---   c   g   g   u

gxmmmbbbgmmBmmmmmmmmmmmxmmmmmmmxgmm
</pre></td></tr>
<tr><td>sequence</td><td><pre>
(.(((...(((((((((((((.(((((((.(((.....))).))))))).))))))))))).)))))).)
ugacgugagccaaacuuuucagauaccguagugucgcauacgguacgguagcugaaaaguuucggucgugg
</pre>
folding energy of structure: -31.0 kcal/mol</td></tr>
</table>

<table>
<tr><td>id</td><td>1966903</td></tr>
<tr><td>genomic_position</td><td>11532610-11532687 on CEL150_II (+) in Caenorhabditis elegans</td></tr>
<tr><td>L score</td><td>1.0</td></tr>
<tr><td>structure</td><td><pre>
                - c   - u
ggcaacuuccugcca ca uuuggcaacuuauuu ag g
||||||||||||||| || ||||||||||||||| || c
ccguugaaggacggu gu aagccguugaauaaa uc c
                c u   a a

mmmmmmmmmmmmmmmmBmmxmmgmmmmmmmmmmmmmBmm
</pre></td></tr>
<tr><td>sequence</td><td><pre>
(((((((((((((((((.(((((((((((((((((.....)).))))))))))))))).)).)))))))))))))))
ggcaacuuccugccacacuuuggcaacuuauuuagugccacuaaaauaaguugccgaauugcuggcaggaaguugcc
</pre>
folding energy of structure: -51.0 kcal/mol</td></tr>
</table>

<table>
<tr><td>id</td><td>1972296</td></tr>
<tr><td>genomic_position</td><td>11532610-11532687 on CEL150_II (-) in Caenorhabditis elegans</td></tr>
<tr><td>L score</td><td>1.0</td></tr>
<tr><td>structure</td><td><pre>
                g a c   u a
ggcaacuuccugcca ca uu ggcaacuuauuu ag g
||||||||||||||| || || |||||||||||| || u
ccguugaaggacggu gu aa ccguugaauaaa uc g
                - g a   - g

mmmmmmmmmmmmmmmmbmmxmmxmmmmmmmmmmmmmbmm
</pre></td></tr>
<tr><td>sequence</td><td><pre>
(((((((((((((((.((.((.((((((((((((.((.....)))))))))))))).)).)))))))))))))))))
ggcaacuuccugccagcaauucggcaacuuauuuuaguggcacuaaauaaguugccaaaguguggcaggaaguugcc
</pre>
folding energy of structure: -47.2 kcal/mol</td></tr>
</table>

**id**	1965421
**genomic_position**	11532911-11532980 on CEL150_II (+) in Caenorhabditis elegans
***L* score**	1.0
**structure**	`           g  a  c            u  u`<br>`acuuccugcca ca uu ggcaacuuauuu ag g`<br>`||||||||||| || || |||||||||||| || g`<br>`ugaaggacggu gu aa ccguugaauaaa uc c`<br>`           -  g  a            -  a`<br><br>mmmmmmmmmmmbmmxmmxmmmmmmmmmmmmbmm
**sequence**	(((((((((((.((.((.((((((((((((.((.....)))))))))))))).)).)))))))))))))<br>acuuccugccagcaauucggcaacuuauuuuaguggcacuaaauaaguugccaaagugugcaggaagu<br><br>folding energy of structure: **-37.0** kcal/mol

**id**	1988720
**genomic_position**	12519797-12520032 on CEL150_II (+) in Caenorhabditis elegans
***L* score**	1.0
**structure**	`  a a                      a                         a                                   ---    g              uuuccuc`<br>`cg c auauuaccgguacagagagugu gauaguuagagagugacagacaucc ggauccaauggggcggggcgcgcggaagagacgau   uugu ucgauuuacgaaau       g`<br>`|| | |||||||||||||||||||||| ||||||||||||||||||||||||| |||||||||||||||||||||||||||||||||||   |||| ||||||||||||||`<br>`gc g uauaauggccaugucucucaca cuaucaaucucucacugucuguagg ccuggguuaccccgccccgcgcgccuucucugcug   aaca agcuaaaugcuuua       u`<br>`  a c                      a                         c                                   cua    g              cuacugu`<br><br>mmxmxmmmmmmmmmmmmmmmmmmmmmmxmmmmmmmmmmmmmmmmmmmmmmmmmxmmmgmmmmmmmmmmmmmmmmmmmmmmmmmmmmmmmmmgBBBmmmmxmmmmmmmmmmmmmm
**sequence**	((.(.((((((((((((((((((((((.(((((((((((((((((((((((((.(((((((((((((((((((((((((((((((((((((((.((((((((<br>(((((((................)))))))))))))).))))...))))))))))))))))))))))))))))))))))).))))))))))))))))))))<br>))))).)))))))))))))))))))))).).))<br>cgacaauauuaccgguacagagaguguagauaguuagagagugacagacauccaggauccaauggggcggggcgcgcggaagagacgauuugugucgauuu<br>acgaaauuuuccucguugucaucauuucguaaaucgagacaaaucgucgucucuuccgcgcgccccgccccauugggucccggaugucugucacucucuaa<br>cuaucaacacucucuguaccgguaauaucgacg<br><br>folding energy of structure: **-191.19** kcal/mol

**id**	2003357
**genomic_position**	12519797-12520032 on CEL150_II (-) in Caenorhabditis elegans
***L* score**	1.0
**structure**	`  u                        u                             c                             cga     c              gauga u`<br>`cg cgauauuaccgguacagagagugu gauaguuagagagugacagacauccggga ccaauggggcggggcgcgcggaagagacga   uuugu ucgauuuacgaaau     c g`<br>`|| |||||||||||||||||||||| ||||||||||||||||||||||||||||| ||||||||||||||||||||||||||||||   ||||| ||||||||||||||     | a`<br>`gc guuauaauggccaugucucucaca cuaucaaucucucacugucuguagguccu gguuaccccgccccgcgcgccuucucugcu   aaaca agcuaaaugcuuua     g c`<br>`  u                        u                             a                             ---     c              aaag- a`<br><br>mmxmgmmmmmmmmmmmmmmmmmmmmmmxmmmmmmmmmmmmmmmmmmmmmmmmmmmgmxmmmmmmmmmmmmmmmmmmmmmmmmmmmmmmbbbmmmmmxmmmmmmmmmmmmmmxxxxbm
**sequence**	((.((((((((((((((((((((((((.(((((((((((((((((((((((((((((.((((((((((((((((((((((((((((((...(((((.((((<br>((((((((((.....(.....)....)))))))))))))).))))))))))))))))))))))))))))))))))).))))))))))))))))))))))))<br>))))).)))))))))))))))))))))))).))<br>cgucgauauuaccgguacagagaguguugauaguuagagagugacagacauccgggacccaauggggcggggcgcgcggaagagacgacgauuugucucga<br>uuuacgaaaugaugacaacgaggaaaauuucguaaaucgacacaaaucgucucuuccgcgcgccccgccccauuggauccuggaugucugucacucucuaa<br>cuaucuacacucucuguaccgguaauauugucg<br><br>folding energy of structure: **-195.2** kcal/mol

**id**	2157900
**genomic_position**	3145224-3145336 on CEL150_III (+) in Caenorhabditis elegans
***L* score**	1.0
**structure**	`             aga    gaa       a                    aacg`<br>`gcguuugaaguug   cgac   agacugu gagggaggggagacgcagac    c`<br>`|||||||||||||   ||||   ||||||| ||||||||||||||||||||`<br>`cgcaaacuucgau   guug   ucugaca cuuccuccccucugcgucug    g`<br>`             caa    auc       c                    ccgc`<br><br>mmmmmmmmmmgmgxxxmgmmxxxmmmmmmmxmmgmmmmmmmmmmmmmmmmm
**sequence**	(((((((((((((...((((...(((((((.((((((((((((((((((((..........))))))))))))))))))))).)))))))...))))...)))<br>))))))))))<br>gcguuugaaguugagacgacgaaagacuguagagggaggggagacgcagacaacgcgcgccgucugcgucuccccuccuuccacagucucuaguugaacuag<br>cuucaaacgc<br><br>folding energy of structure: **-75.5** kcal/mol

**id**	2246908
**genomic_position**	3145240-3145320 on CEL150_III (-) in Caenorhabditis elegans
***L* score**	1.0
**structure**	a u a g<br>c ac agagacugugga ggaggggagacgcagacggc c<br>| || |||||||||||| |||||||||||||||||||| g ug uuucugacaucu ccuccccucugcgucuguug g<br>c c c c<br><br>mxmmxmgmmmmmmmgmmxmmmmmmmmmmmmmmmmmmggm
**sequence**	(.((.((((((((((((.((((((((((((((((((((....)))))))))))))))))))).)))))))))))).)).)<br>caacuagagacuguggaaggaggggagacgcagacggcgcgcguugucugcgucuccccucccucuacagucuuucgucg<br><br>folding energy of structure: **-62.6** kcal/mol

**id**	2246732
**genomic_position**	3145991-3146089 on CEL150_III (-) in Caenorhabditis elegans
***L* score**	1.0
**structure**	a-- a g g gaa g u u u<br>gagag gag ga agaga ugug gga gggagacgcag g gc c<br>||||| ||| || ||||| |||| ||| ||||||||||| | || cuuuc uuc cu uuucu acac ucu cccucugcguc c cg g<br>aac c g g acg a u c c<br><br>mmgmmxBBgmmxmmxmgmmmxmmmmxxxgmmxmmmmmmmmmmmmxmxmm
**sequence**	(((((.(((.((.(((((.((((...(((.(((((((((((.(.((....)).).))))))))))).)))...)))).))))).)).)))...)))))<br>gagagagagagagagagagaguguggaaggaggggagacgcagugugcgcucgcccucugcgucucccaucugcacacagucuuugucccuucaacuuuc<br><br>folding energy of structure: **-50.3** kcal/mol

<table>
<tr><th>id</th><td>2158072</td></tr>
<tr><th>genomic_position</th><td>3146691-3146787 on CEL150_III (+) in Caenorhabditis elegans</td></tr>
<tr><th>L score</th><td>1.0</td></tr>
<tr><th>structure</th><td><pre>
   acau gu-   c ac a   g   g
guu   ag   ugagagaguguggg g g ggagacgcagg ggc c
||| || |||||||||||||| | | ||||||||||| |||
cga   uc   gcucucucacaucc c c ccucugugucu cug a
   cuu- auu   a cu c   g   c

mgmxxxbmmxxBgmmmmmmmmmmgmmxmxxmxmmmmmmgmmmgxmgm
</pre></td></tr>
<tr><th>sequence</th><td><pre>
(((....((..((((((((((((((.(..(.(((((((((((.(((....))).))))))))))).)..).))))))))))))))...))...)))
guuacauagguugagagaguguggggcgacgaggagacgcagggggcgcacgucgucugugucuccccuccaccuacacucucucguuacuuucagc
</pre>
folding energy of structure: -50.9 kcal/mol</td></tr>
</table>

<table>
<tr><th>id</th><td>2182845</td></tr>
<tr><th>genomic_position</th><td>3146698-3146781 on CEL150_III (+) in Caenorhabditis elegans</td></tr>
<tr><th>L score</th><td>1.0</td></tr>
<tr><th>structure</th><td><pre>
  gu-   c ac a   g   g
ag   ugagagaguguggg g g ggagacgcagg ggc c
|| |||||||||||||| | | ||||||||||| |||
uc   gcucucucacaucc c c ccucugugucu cug a
  auu   a cu c   g   c

mmxxBgmmmmmmmmmmgmmxmxxmxmmmmmmgmmmgxmgm
</pre></td></tr>
<tr><th>sequence</th><td><pre>
((..((((((((((((((.(..(.(((((((((((.(((....))).))))))))))).)..).))))))))))))))...))
agguugagagaguguggggcgacgaggagacgcagggggcgcacgucgucugugucuccccuccaccuacacucucucguuacu
</pre>
folding energy of structure: -50.6 kcal/mol</td></tr>
</table>

**id**	2246304
**genomic_position**	3146703-3146775 on CEL150_III (-) in Caenorhabditis elegans
***L* score**	1.0

structure

```
          a    ga        a   a---  u
gagagagugu ggug  ggggagac cag    cg g
|||||||||| ||||  |||||||| |||    || c
cucucucaca ccgc  cuccucug guc    gc g
          c    ug        c   cccc  c

mmmmmmmmmmxmmgmxxmgmmmmmmxmmmxBBBmm
```

sequence

```
((((((((((.((((..((((((((.(((.((.....))....))).))))))))..)))).))))))))))
gagagaguguagguggaggggagacacagacgacgugcgcccccugcgucuccucgucgcccacacucucuc
```

folding energy of structure: **-42.8** kcal/mol

**id**	2631469
**genomic_position**	3258103-3258214 on CEL150_IV (-) in Caenorhabditis elegans
***L* score**	1.0

structure

```
         -   cc  a  a  u   a           ua   -   -   - ua
guaggcaug aga  gu ag ca gca guagguaggcg  agc aug uag c  c
||||||||| |||  || || || ||| |||||||||||  ||| ||| ||| |  g
cgucuguac ucu  ca uc gu cgu cauccguccgu  ucg ugc guc g  u
         a   au  g  c  c   a           ca   c   c   u uc

mgmmgmmmmBmmmxxmmxmmxmmxmmmxmmmmmgmmmmgxxmmmBmgmBgmmBm
```

sequence

```
(((((((((((..((.((.((.(((.(((((((((((..((((((((((.......).))).))).)))..))))))))))).))).))
.)).))..))).)))))))))
guaggcaugagaccguaagacaugcaaguagguaggcguaagcauguagcuauauacgucugccgucgcuacugccugccuacaugccug
ccugacuaucuacaugucugc
```

folding energy of structure: **-46.8** kcal/mol

id	4356809
genomic_position	3261924-3262012 on CEL150_IV (+) in Caenorhabditis elegans
L score	1.0

structure

```
  g   c    c       - --           --   caacc
cu uga caau agcgauu c  gcuccgcccacu  uuc     a
|| ||| |||| ||||||| |  ||||||||||||  |||     a
ga gcu guua uugcuga g  ugaggcgggugg  aag     u
  g   c    u       a uu           ug   cagac

mmxgmmxmmmmxmgmmmgmBmBBgmmmmmmmmmmgBBmmm
```

sequence

```
((.(((.((((.(((((((((((((((((((((((.............)))..))))))))))))..).))))))).)))).))).))
cugugaccaaucagcgauucgcuccgcccacuuuccaaccaaucagacgaaguggugggcggaguuugaagucguuuauugcucggag
```

folding energy of structure: **-35.52** kcal/mol

id	4590820
genomic_position	4270554-4270641 on CEL150_IV (-) in Caenorhabditis elegans
L score	1.0

structure

```
                agcu   ug  g          caca
cuguagugguacugug    uau  ua gauuauugua    c
||||||||||||||||    |||  || ||||||||||    c
gacaucaccaugacau    aug  au cugaugacau    u
                cauu   cg  g          caag

mmmmmmmmmmmmmmmmgxxxxmmgxxmmxmmgmmgmmmm
```

sequence

```
((((((((((((((((....(((..((.((((((((((...........)))))))))).))..)))....))))))))))))))))
cuguagugguacugugagcuuauuguaggauuauuguacacaccugaacuacaguagucguagcguauuacuacaguaccacuacag
```

folding energy of structure: **-36.7** kcal/mol

**id**	4576692
**genomic_position**	4272130-4272199 on CEL150_IV (+) in Caenorhabditis elegans
***L* score**	1.0
**structure**	** g c gc g guu c**<br>**ac cuug cga cuugucgacuu uua cu u**<br>**|| |||| ||| ||||||||||| ||| ||**<br>**ug gaac gcu ggacagcugag aau ga c**<br>** a u ga - auu c**<br><br>mmxmmmmxmmmxxmgmmmmmmmmmgbmmmxxxmm
**sequence**	((.((((.(((..((((((((((((.(((...((....))...)))))))))))))))..))).)))).))<br>acgcuugccgagccuugucgacuuguuaguucucuccaguuauaagagucgacaggagucgucaagagu<br><br>folding energy of structure: **-24.7** kcal/mol

**id**	2915574
**genomic_position**	14032857-14032960 on CEL150_IV (-) in Caenorhabditis elegans
***L* score**	1.0
**structure**	** cu cu c ----- g - g cuu**<br>**cgu uagaca cg gaga aa agagagaga gagugu uguguu g**<br>**||| |||||| || |||| || ||||||||| |||||| |||||| a**<br>**gca aucugu gc uucu uu ucucucucu uucaca acgcag g**<br>** u- u- c cccac g g g gca**<br><br>mmmxbmmmmmmxbmmxgmmmBBBBBmmxmmmmmmmmmBgmmmmmxmmgmmg
**sequence**	(((..((((((..((.((((((.((((((((((((((.((((((.........)))))).)))))).))))))))).)).....))))<br>.)).))))))).)))<br>cgucuuagacacucgcgagaaagagagagagagagugugugguugacccuugagacgcagacacuugucucucucuguucacccucuu<br>ccguugucuauacg<br><br>folding energy of structure: **-42.2** kcal/mol

<table>
<tr><td>id</td><td>4324818</td></tr>
<tr><td>genomic_position</td><td>5441650-5441724 on CEL150_V (-) in Caenorhabditis elegans</td></tr>
<tr><td>L score</td><td>1.0</td></tr>
<tr><td>structure</td><td><pre>
       c   c   uac   a   c u
acuguag gcuugug ugauu   gcggg ucg uu u
||||||| ||||||| ||||| ||||| ||| ||
ugauguc cgaacac gcuaa   ugccc agu aa u
       u   a   u--   a   a u

mmmggmmxmmmmmmmxgmmmmxbbgmmmmxmmgxmm
</pre></td></tr>
<tr><td>sequence</td><td><pre>
(((((((.(((((((.(((((...(((((.(((.((....)).))).))))).))))).))))))).)))))))
acuguagcgcuugugcugauuuacgcgggaucgcuuuucgaaaugaacccguuaaucgacacaagcucuguagu
</pre>
folding energy of structure: -29.0 kcal/mol</td></tr>
</table>

<table>
<tr><td>id</td><td>3800893</td></tr>
<tr><td>genomic_position</td><td>2365035-2365147 on CEL150_X (-) in Caenorhabditis elegans</td></tr>
<tr><td>L score</td><td>1.0</td></tr>
<tr><td>structure</td><td><pre>
                        c   a   c   ac   uuuu
ucuuacauuauuucguugauuaag uauauuua agc gauaggua caa   a
|||||||||||||||||||||||| |||||||| ||| |||||||| |||
agaguguaauaaaguaacuaauuc auguaaau ucg cuauccgu guu   a
                        c   g   u   ga   uacu

mmmgmmmmmmmmmmgmmmmmmmmmxmmgmmmmmxmmmxmmmmmmgmxxmmm
</pre></td></tr>
<tr><td>sequence</td><td><pre>
((((((((((((((((((((((((.((((((((.(((.((((((((..(((..........)))..)))))))).))).)))))))).)))))
)))))))))))))))))))
ucuuacauuauuucguugauuaagcuauauuuaaagccgauagguaaccaauuuuaaucauuugagugccuaucugcuguaaauguaccuuaa
ucaaugaaauaaugugaga
</pre>
folding energy of structure: -54.6 kcal/mol</td></tr>
</table>

<table>
<tr><td>id</td><td>3791358</td></tr>
<tr><td>genomic_position</td><td>2365039-2365143 on CEL150_X (+) in Caenorhabditis elegans</td></tr>
<tr><td>L score</td><td>1.0</td></tr>
<tr><td>structure</td><td>   a   g c   c   a   c cu   a ga
acauuauuuc uugauuaag ua auuua agc gauagg a caa u u
|||||||||| ||||||||| || ||||| ||| |||||| | ||| |
uguaauaaag aacuaauuc au uaaau ucg cuaucc u guu a u
          c   g a   u   g   a ug   a aa

mmmmmmmmmmxmmmmmmmmmxmmxmmmmmxmmmxmmmmmmxmxxmmmxm</td></tr>
<tr><td>sequence</td><td>((((((((((.(((((((((.((.(((((.(((.((((((.(..(((.(......).)))..).)))))).))).))))).)).)))
)))))).))))))))))
acauuauuucauugauuaagguacauuuacagcagauaggcacucaaaugauuaaaauugguuaccuaucggcuuuaaauauagcuu
aaucaacgaaauaaugu

folding energy of structure: -37.5 kcal/mol</td></tr>
</table>

<table>
<tr><td>id</td><td>3848323</td></tr>
<tr><td>genomic_position</td><td>3003184-3003251 on CEL150_X (-) in Caenorhabditis elegans</td></tr>
<tr><td>L score</td><td>1.0</td></tr>
<tr><td>structure</td><td> g - a   a c- ug
uc cg ca uggucucgccacgcgc uc cg a
|| || || |||||||||||||||| || ||
ag gc gu accagagcggugcgcg ag gu u
  g a a   - uu uu

mmxmmBmmxmmmmmmmmmmmmmmmbmmxBmg</td></tr>
<tr><td>sequence</td><td>((.((((.((((((((((((((((.((.((......))..)))))))))))))))))).)).)).))
ucgcgcaauggucucgccacgcgcaucccguugauuuguugagcgcguggcgagaccaaugacggga

folding energy of structure: -41.1 kcal/mol</td></tr>
</table>

**id**	3877152
**genomic_position**	5801239-5801380 on CEL150_X (+) in Caenorhabditis elegans
***L* score**	1.0

structure

```
   g   cu       c  ga  cg   guua    g    c    --  -       -     -     ucc
cgu cuu  guuucua ua  cu  ugu    uucc cuca uuuc  ca acuucuc ccuuu uacac   a
||| |||  ||||||| ||  ||  |||    |||| |||| ||||  || ||||||| ||||| |||||
gca gag  uaaagau gu  gg  gca    aagg gagu agag  gu ugaagag gggag augug   u
   -   ag       a  aa  aa   aaag    -    a    aa  a       a     u     uuu

mmmbmmgxxgmmmmmmxgmxxmgxxgmmxxxxmmmmbmmmmxmgmmBBmmBmmmmmmmBmmgmgBmmmmm
```

sequence

```
(((.(((..(((((((.((..((..(((....((((.((((.(((((((((((((((((((((((........))))).))))).)))
)))).))..)))).))))))))....)))..))..)).)))))))..))))))

cgugcuucuguuucuacuagacucguguguuauuccgcucacuuuccaacuucucccuuuuacacuccauuuuguguaugagggagag
aaguaugaagagaaugagggaagaaaacgaaggaaugauagaaaugagagacg
```

folding energy of structure: **-47.9** kcal/mol

**id**	3898593
**genomic_position**	5801271-5801349 on CEL150_X (+) in Caenorhabditis elegans
***L* score**	1.0

structure

```
    g    c    --  -       -     -     ucc
uucc cuca uuuc  ca acuucuc ccuuu uacac   a
|||| |||| ||||  || ||||||| ||||| |||||
aagg gagu agag  gu ugaagag gggag augug   u
    -    a    aa  a       a     u     uuu

mmmmbmmmmxmgmmBBmmBmmmmmmmBmmgmgBmmmmm
```

sequence

```
((((.((((.(((((((((((((((((((((((........))))).))))).))))))).))..)))).))))))))
uuccgcucacuuuccaacuucucccuuuuacacuccauuuuguguaugagggagagaaguaugaagagaaugagggaa
```

folding energy of structure: **-31.2** kcal/mol

**id**	4064562
**genomic_position**	11003778-11003882 on CEL150_X (-) in Caenorhabditis elegans
***L* score**	1.0

structure

```
   a  ----  a      auu--     a  c                   cg
guu gc    cg guuucg     uuagc ga auuagccgaguuagccgaa  u
||| ||    || ||||||     ||||| || |||||||||||||||||||  u
caa cg    gc cgaggu     aaucg cu uaaucggcuuaaucggcuu  c
   a  cuua  a      aacac     g  -                   gg

mmmxmmBBBBmmxmgmgmgxxxBBmmmmmxmmbmmmmmmmmmgmmmmmmmmm
```

sequence

```
(((.((((.((((((...(((((.((.(((((((((((((((((((.......))))))))))))))))))))).))))))..
...)))))).))....)).)))

guuagccgaguuucgauuuuagcagacauuagccgaguuagccgaaauauucguucggcuaauucggcuaauucggcuaaca
caauggagcacgauucgcaaac
```

folding energy of structure: **-43.2** kcal/mol

**id**	4069058
**genomic_position**	11003788-11003876 on CEL150_X (-) in Caenorhabditis elegans
***L* score**	1.0

structure

```
  a      auu--     a  c                   ua
cg guuucg     uuagc ga auuagccgaguuagccgaa  u
|| ||||||     ||||| || |||||||||||||||||||  u
gc cgaggu     aaucg cu uaaucggcuuaaucggcuu  c
  a      aacac     g  -                   ug

mmxmgmgmgxxxBBmmmmmxmmbmmmmmmmmmgmmmmmmmmm
```

sequence

```
((.((((((...(((((.((.(((((((((((((((((((.......))))))))))))))))))))).))))).....)))))).))
cgaguuucgauuuuagcagacauuagccgaguuagccgaaauauucguucggcuaauucggcuaauucggcuaacacaauggagcacg
```

folding energy of structure: **-41.4** kcal/mol

**id**	4047686
**genomic_position**	11003866-11003974 on CEL150_X (+) in Caenorhabditis elegans
***L* score**	1.0
**structure**	** ac-- uaa - a aug cg cgg**<br>**ucgaa ucggc cggcuaauuuggcuaau cggu aa gu gcuga c**<br>**||||| ||||| ||||||||||||||||| |||| || || |||||**<br>**ggcuu agcug gccgauuaaaccgauua gccg uu ua cggcu u**<br>** aauc --- a a a-- au uaa**<br><br>gmmmmxxBBmmmgmbbbmmmmmmmmmmmmmmmmmBmmmgxmmxbbgmxxmmgmm
**sequence**	(((((..(((((...(((((((((((((((((((((.((...((..(((((........)))))..)).)).)))).))))))))))))<br>))))))))))....)))))<br>ucgaaacucggcuaacggcuaauuuggcuaaucgguaaaauggucggcugacggcuaauucggcuaauauuagccgaauuagccaaauua<br>gccggucgacuaauucgg<br><br>folding energy of structure: **-48.2** kcal/mol

**id**	4058842
**genomic_position**	11003873-11003965 on CEL150_X (+) in Caenorhabditis elegans
***L* score**	1.0
**structure**	** uaa - a aug cg cgg**<br>**ucggc cggcuaauuuggcuaau cggu aa gu gcuga c**<br>**||||| ||||||||||||||||| |||| || || |||||**<br>**agcug gccgauuaaaccgauua gccg uu ua cggcu u**<br>** --- a a a-- au uaa**<br><br>mmmgmbbbmmmmmmmmmmmmmmmmmBmmmgxmmxbbgmxxmmgmm
**sequence**	(((((...(((((((((((((((((((((.((...((..(((((........)))))..)).)).)))).))))))))))))))))))))))<br>ucggcuaacggcuaauuuggcuaaucgguaaaauggucggcugacggcuaauucggcuaauauuagccgaauuagccaaauuagccggucga<br><br>folding energy of structure: **-45.0** kcal/mol

<table>
<tr><th>id</th><td>4051955</td></tr>
<tr><th>genomic_position</th><td>11003882-11003985 on CEL150_X (+) in Caenorhabditis elegans</td></tr>
<tr><th>L score</th><td>1.0</td></tr>
<tr><th>structure</th><td><pre>
   ua u   -   cg aaaa gu   c--   a
ggc a uuggcu aau gu   ug cggcuga   ggcuaauucggcua u
||| | |||||| ||| || || ||||||| ||||||||||||||
ucg u aaucgg uua ca   gc gccgauu   ccgauuaagccgau a
   gc u   c   au ---- ug   aaa   u
</pre>
gmmxxmxmmgmmgBmmmxxmmbbbbgmxxmmmmmgmxBBmmmmmmmmmmmmmmm</td></tr>
<tr><th>sequence</th><td>(((..(.(((((((((..((....((..(((((((.((((((((((((((....))))))))))))))...))))))).))))
..))).)))))))).)..)))
ggcuaauuuggcuaaucgguaaaauggucggcugacggcuaauucggcuaauauuagccgaauuagccaaauuagccggucgac
uaauucggcuaauucggcu

folding energy of structure: -50.5 kcal/mol</td></tr>
</table>

<table>
<tr><th>id</th><td>4063259</td></tr>
<tr><th>genomic_position</th><td>11003927-11003992 on CEL150_X (-) in Caenorhabditis elegans</td></tr>
<tr><th>L score</th><td>1.0</td></tr>
<tr><th>structure</th><td><pre>
      ---   c
ggcuaa   uagccgaauuagccgaauuaguc g
|||||| |||||||||||||||||||||||
ccgauu   aucggcuuaaucgguuuaaucgg g
      aua   c
</pre>
mmmmmmBBBmmmmmmmmmmmmmmmgmmmmmmgm</td></tr>
<tr><th>sequence</th><td>((((((((((((((((((((((((((((((....)))))))))))))))))))))))))...))))))
ggcuaauagccgaauuagccgaauuagucgaccggcuaauuuggcuaauucggcuaauauuagcc

folding energy of structure: -44.1 kcal/mol</td></tr>
</table>
