# Supplementary material for: In silico miRNA prediction in metazoan genomes: balancing between sensitivity and specificity
Source: BMC Genomics. 2009 Apr 30;10:204. doi: 10.1186/1471-2164-10-204 (PMC2688010; doi:10.1186/1471-2164-10-204)
Supplement: Additional file 8 — Hairpin loci in C. elegans obtained by the filtering protocol "Similar". Filtering on L score was combined with filtering on a similarity threshold to known metazoan mature miRNAs, a protocol referred to as "Similar". [file 1471-2164-10-204-S8.pdf]

Additional File 8: Hairpin loci in *C. elegans* obtained by the filtering protocol "Similar"

List of 41 identified loci in *C. elegans* of genomic hairpins with *L* score >= $1e^{-5}$, not overlapping with exons, having similarity to a known metazoan mature miRNA (at most 3 three mismatches on 19 nt length) and binary filtered on seven individual descriptors: four based on structure (stem length <= 55, loop length <= 40, largest bulge <= 8, max match count >= 17) and three based on sequence complexity (polyNucHairpin <= 8, SCS-mono >= -10, SCS-di <= 0.40). Except for stem length, all individual thresholds fall within the *S*<1 fractions of the descriptors and separately reject at most three *C. elegans* miRNA hairpins. After removal of genomic hairpins that represent known miRNA hairpins, 64 loci remain.

The 84 hairpins of these 64 loci are further filtered for the position of the similarity within the hairpin. The similarity with the mature miRNA may have at most 3 nt overlap with the hairpin loop coordinates and at least 8 nt of stem must separate the similarity from the end of the hairpin. These criteria are set according to the biological model of miRNA maturation from hairpins (*Zeng et al. 2005*). This final filtering step reduced the list to 51 genomic hairpins on 41 loci. Below, the hairpin with the highest *L* score for each locus is given. *L* scores are obtained from the scoring model *Metazoa*.

The 18 descriptors in the figures, from left to right: MFEahl, MFEahl index, Q, max match count, bulgeRatio, GU-match contribution, largest bulge, longest match-stretch, looplength, stem length, dP, SCS-mono, SCS-di, polyA, polyU, polyNucHairpin, GsurplusC, GasurplusCU.

‘Genpos’ denotes the genomic position of the hairpin:

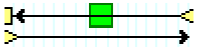 intergenic

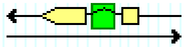 intronic, on the same strand as the exons

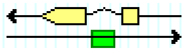 intronic but opposite to the strand of the exons

Highest scoring hairpins from the loci obtained by the filtering protocol “Similar” on chromosomes I-III

Id	Position	Strand																			Lscore	Genpos
1652826	I 103237..103347	-																			1.1e-04	
1165306	I 1733470..1733572	+																			0.030	
1760974	I 1733608..1733684	-																			0.044	
1184736	I 3531370..3531472	+																			3.7e-05	
1280658	I 9372758..9372852	+																			0.089	
4284206	I 10325772..10325844	+																			2.9e-04	
1715134	I 11708848..11708926	-																			2.6e-05	
1446507	II 2633349..2633441	+																			1.5e-05	
1891777	II 9403659..9403769	+																			6.7e-04	
1959550	II 11841755..11841841	+																			2.0e-04	
2004295	II 12609234..12609323	-																			0.048	
1991116	II 12941938..12942051	+																			4.9e-05	
2043412	II 13173538..13173633	+																			0.102	
2064086	II 13502012..13502095	+																			1.8e-04	
2047661	II 13515555..13515672	+																			7.2e-03	
1976550	II 15256703..15256813	+																			9.5e-05	
4515471	III 1101498..1101616	+																			2.4e-03	
4546124	III 2916984..2917057	+																			0.016	
2229988	III 3596986..3597083	+																			0.011	
2196306	III 3876769..3876860	+																			0.023	
2414207	III 10524895..10524964	+																			0.537	

Highest scoring hairpins from the loci obtained by the filtering protocol “Similar” on chromosomes IV-X

Id	Position	Strand	Lscore	Genpos
2490824	IV 245875..245960	+	0.051	
2875398	IV 12806506..12806596	+	0.018	
2981916	IV 15168041..15168155	+	5.6e-03	
3083314	V 600115..600211	-	8.2e-03	
3130412	V 1304648..1304762	+	6.5e-03	
3148670	V 2851038..2851136	+	0.097	
3179049	V 2851151..2851221	-	0.035	
3240609	V 4703559..4703654	-	5.7e-05	
3227648	V 4844054..4844172	+	1.7e-05	
3411064	V 11066747..11066854	-	0.102	
3450047	V 12302101..12302170	-	2.8e-05	
3561947	V 16348305..16348419	+	0.015	
3714507	V 20590843..20590916	-	2.2e-03	
3729314	X 539532..539646	-	4.4e-04	
3731460	X 624955..625048	-	1.1e-04	
3846390	X 3912837..3912933	-	9.3e-03	
4048106	X 11051158..11051250	+	3.5e-04	
4147031	X 14917325..14917447	+	7.2e-05	
4167805	X 14917516..14917627	-	0.035	
4181655	X 15447707..15447814	-	2.7e-03	

**id**	1652826
**genomic_position**	103236-103347 on CEL150_I (-) in Caenorhabditis elegans
***L* score**	0.000113029645737

structure

```
    g--    c      u  aaac   gauuu        u      -     u--  a
gcau   guac auuucu ga    gua     uguuuucg agaucu ccaaa   ag g
||||   |||| |||||| ||    |||     |||||||| |||||| |||||   || u
cgug   caug uagagg cu    cgu     gcaaaagu ucuaga gguuu   uc a
    aga    -      c  c---   ggu--        -      u     ugu  g

mmmgxBBmmmmbmmgmmgxmmxbbbmgmxxxbbgmmmmmmgbmmmmmmBmmmmmxBBmm
```

sequence

```
((((.((((.((((((.((....(((.....((((((((.(((((((((((.((.....))...))))).))))))))))))))...))).)
).)))))))))))...))))
```

gcaugguaccauuucuug*aaaacguagauuuuguuuu*cguagaucuccaaauaguagaucuuguuuugguagaucuugaaaacguggugccu
ccggagauguacagagugc

folding energy of structure: **-32.8** kcal/mol
1 known mature miRNA(s) exhibit sequence similarity to this candidate hairpin

**id**	1165306
**genomic_position**	1733469-1733572 on CEL150_I (+) in Caenorhabditis elegans
***L* score**	0.0300871112455

structure

```
        c   a   g g     c     c   a     a  cg   gaa
acuugggu aga guu g caaga uuugg gaa cuugg uu  ggc   a
|||||||| ||| ||| | ||||| ||||| ||| ||||| ||  |||   u
ugaaccca uuu caa c guucu aaacc cuu gaacc ga  uug   c
        -   g   a g     c     a   g     -  au   aaa

mmmmmmmmbmgmxmmmxmxmmmmmxmmmmmxmmmxmmmmmbgmxxggm
```

sequence

```
((((((((.(((.(((.(.(((((.(((((.(((.(((((.((..(((.........)))..))))))).))).))))).))))).).))).))
))))))))
```

acuugggucagaaguug*ggcaagacuuuggcgaaacu*uggauucgggcgaaaucaaaguuuaagccaagguucaccaaacucuuggcaaacguu
uacccaagu

folding energy of structure: **-40.6** kcal/mol
1 known mature miRNA(s) exhibit sequence similarity to this candidate hairpin

<table>
<tr><th>id</th><td>1760974</td></tr>
<tr><th>genomic_position</th><td>1733607-1733684 on CEL150_I (-) in Caenorhabditis elegans</td></tr>
<tr><th>L score</th><td>0.0442810979858</td></tr>
<tr><th>structure</th><td> ga   ca   u   a-   g   cg
ca aguuggg agacuu ggc aaacuu gauu g
|| ||||||| |||||| ||| |||||| |||| g
gu ucaaccc uuugaa ccg uuugaa cuaa u
  uc   ac   -   aa   a   ag

mmxxmmmmmmmxxmgmmmmbmmmxBmmmmmmxmmmm</td></tr>
<tr><th>sequence</th><td>((..(((((((..((((((.(((.((((((.((((.......)))).))))))..)))))))))..))))))).))
cagaaguugggcaagacuuuggcaaaacuuggauucgggugaaaucaaaguuuaagccaaguuucacccaacucuug

folding energy of structure: -27.2 kcal/mol
2 known mature miRNA(s) exhibit sequence similarity to this candidate hairpin</td></tr>
</table>

<table>
<tr><th>id</th><td>1184736</td></tr>
<tr><th>genomic_position</th><td>3531369-3531472 on CEL150_I (+) in Caenorhabditis elegans</td></tr>
<tr><th>L score</th><td>3.69668875878e-05</td></tr>
<tr><th>structure</th><td>   c g   aaaa   ca   ca   - auuca
ggcuaaa uu ggcaa   uagg agacuuggg aaacuu gg   g
||||||| || ||||| |||| ||||||||| |||||| ||
ccgguuu aa ccguu   aucc uuugaaccc uuugaa cc   g
       c a   caca   ac   ac   a aauuc

mmmgmmmxmmxmmmmmxxxxmmmmxxmgmmmmmmmxxmmmmmmBmm</td></tr>
<tr><th>sequence</th><td>(((((((.((.(((((....((((..(((((((((..((((((((............)).))))))..)))))))))..))))....))))).)).)))))))
ggcuaaacuugggcaaaaaauaggcaagacuugggcaaaacuuggauucaggcuuaaccaaaguuucacccaaguuucaccuaacacuugccaaacuuuggcc

folding energy of structure: -38.4 kcal/mol
2 known mature miRNA(s) exhibit sequence similarity to this candidate hairpin</td></tr>
</table>

**id**	1280658
**genomic_position**	9372757-9372852 on CEL150_I (+) in Caenorhabditis elegans
***L* score**	0.0892294092686

structure

```
      ca     c   a         --   cug    a  aaa u  u
acagug  acagg agc caucaaagc  ugg   ugau ug   g ca a
||||||  ||||| ||| |||||||||  |||   |||| ||   | || a
ugucau  ugucu ucg guaguuucg  acc   acua ac   c gu u
      a-     u   -         cc   a--    c  aa- -  u

mmmmmgxbmmmmgxmmmbmmmmmmmmmBBmmmxbbmmmmxmmxxbmbmm
```

sequence

```
((((((..(((((.(((.((((((((((((...((((.((...(.((.....)))..)).)))).)))..)))))))))))).))))).))))))
acagugcaacaggcagcacaucaaagcuggcugugauaugaaagucauaauuugcaacacaucaaccaccgcuuugauggcuuucuguauacugu
```

acagugcaacaggca*gcacaucaaagcuggcugugaua*ugaaagucauaauuugcaacacaucaaccaccgcuuugauggcuuucuguauacugu

folding energy of structure: **-32.4** kcal/mol

2 known mature miRNA(s) exhibit sequence similarity to this candidate hairpin

**id**	4284206
**genomic_position**	10325771-10325844 on CEL150_I (+) in Caenorhabditis elegans
***L* score**	0.000290946981613

structure

```
    aa      aaaac     --          u  ag
ucag  cuuggc     uuugg  caaaacuugg ua  g
||||  ||||||     |||||  |||||||||| ||
gguu  gaaccg     gaacc  guuuugaacc gu  c
    c-      a----     ua          c  gu

gmmgxbmmmmmmxbbbbgmmmmBBmmmmmmmmmmxgm
```

sequence

```
((((..((((((.....((((((((((((((.((......)).))))))))))..))))).)))))).))))
ucagaacuuggcaaaacuuuggcaaaacuugguuaaggcugugcccaaguuuugauccaagagccaagcuugg
```

ucagaacuu*ggcaaaacuuuggcaaaacu*ugguuaaggcugugcccaaguuuugauccaagagccaagcuugg

folding energy of structure: **-22.4** kcal/mol

1 known mature miRNA(s) exhibit sequence similarity to this candidate hairpin

**id**	1715134
**genomic_position**	11708847-11708926 on CEL150_I (-) in Caenorhabditis elegans
***L* score**	2.61725789981e-05

structure

```
    ga          ca      u   a-    g-   c
uugg  aaaaguuggg  agacuu ggc  aagc  ugg u
||||  ||||||||||  |||||| |||  ||||  ||| g
aacc  uuuucaacuu  uuugaa ccg  uuug  acc c
    g-          ac      -   aa    aa   a
```

mmmmxbmmmmmmmmggxxmgmmmmbmmmxBmmgmxBmmm

sequence

((((..((((((((((..((((((.(((.((((.(((.....)))..))))..)))))))))..))))))))))).))))
uugggaaaaaguu*gggcaagacuuuggcaaagc*guggcugcaccaaaguuuaagccaaguuucauucaacuuuugccaa

folding energy of structure: **-25.5** kcal/mol
1 known mature miRNA(s) exhibit sequence similarity to this candidate hairpin

**id**	1446507
**genomic_position**	2633348-2633441 on CEL150_II (+) in Caenorhabditis elegans
***L* score**	1.4530952118e-05

structure

```
    uguaguuu  -   a         a     a   uua    ag
gguu        ga gca cugacaguu acggg auu   gggu  c
||||        || ||| ||||||||| ||||| |||   ||||  a
ccaa        cu cgu ggcuguuaa ugccc uag   cuua  a
    uuuguuuu  a   c         g     a   ---    aa
```

mmmmxxxxxxxxmmBmmmxmgmmmmgmmxmmmmmxmmgbbbmggm

sequence

((((........(((((.(((((((((.(((((.(((...((((.......))))))).))))).))))))))).))).))........))))
gguuuguaguuugagcaacugacaguuaacgggaauuuuaggguagcaaaaauucgaua*cccgugaauugucggcugca*ucuuuuguuuaacc

folding energy of structure: **-27.39** kcal/mol
1 known mature miRNA(s) exhibit sequence similarity to this candidate hairpin

<table>
<tr><td>id</td><td>1891777</td></tr>
<tr><td>genomic_position</td><td>9403658-9403769 on CEL150_II (+) in Caenorhabditis elegans</td></tr>
<tr><td>L score</td><td>0.000671559878891</td></tr>
<tr><td>structure</td><td><pre>
  u   a   a   c u ca   a ca c   a
uc aaacuugggc aaaguu ggcaaga uu gg aaacuugg uu ag cuu c
|| |||||||||| |||||| ||||||| || || |||||||| || || |||
ag uuugaacccg uuucaa ccguucu aa cc uuugaacc aa uc gaa c
  -          g   a   c c ac   c -- u   a
</pre>mmbmmmmmmmmmmxmmmmmmxmmmmmmmxmmxmmxxmmmmmmmmxmmbbmmxmmm</td></tr>
<tr><td>sequence</td><td>((.((((((((((.((((((.(((((((.((.((..((((((((.((..((.(((....))).)))).))))))))..)).)).)))))
)).)))))).)))))))))))
ucuaaacuugggcaaaaguuaggcaagacuuuggcaaaacuuggauucaagccuuaccaaagucuaacccaaguuucacccaacucuug
ccaaacuuuggcccaaguuuga

folding energy of structure: -46.5 kcal/mol
2 known mature miRNA(s) exhibit sequence similarity to this candidate hairpin</td></tr>
</table>

<table>
<tr><td>id</td><td>1959550</td></tr>
<tr><td>genomic_position</td><td>11841754-11841841 on CEL150_II (+) in Caenorhabditis elegans</td></tr>
<tr><td>L score</td><td>0.000204673248929</td></tr>
<tr><td>structure</td><td><pre>
   au-   - acac-   g   -   a aa----
ggc   gcagc cg   cugaca cuuc cgg ga   guaca
||| ||||| || |||||| |||| ||| || |||| a
ccg   cgucg gc   ggcugu gaag gcc cu   cauga
   aau   a acgcc   -   u   - gguuga
</pre>mmmxxBmmmmmBmmxxxxBmgmmmmbmmmmBmmmbmmxxBBBBmmmm</td></tr>
<tr><td>sequence</td><td>(((..(((((((....((((((.(((((((.((..((((...))))......))))).))))))))))).....)).))))))...)))
ggcaugcagccgacaccugacagcuuccggagaaaguacaaaguacaguuggucccgugaagugucggccgcacgagcugcuaagcc

folding energy of structure: -32.3 kcal/mol
1 known mature miRNA(s) exhibit sequence similarity to this candidate hairpin</td></tr>
</table>

**id**	2004295
**genomic_position**	12609233-12609323 on CEL150_II (-) in Caenorhabditis elegans
***L* score**	0.0481020993305

structure

```
    aa           uc  u  c        ga  cagg    gg
ggca  aguuuggcaag  uu gg aaaacuug  uu    agac  a
||||  |||||||||||  || || ||||||||  ||    ||||
ccgu  ucaaaccguuc  aa cc uuuugaac  aa    uuug  g
    --           uc  c  a        uc  ----    ca

mmmmbbmmmmmmmmmmmxxmmxmmxmmmmmmmmxxmmbbbbmgmm
```

sequence

```
((((..(((((((((((..((.((.((((((((..((....((((......))))))..)))))))).)).))..)))))))))))))))
ggcaaaaguuuggcaagucuuuggcaaaacuuggauucaggagacaucaaaguuuaacucaaguuuuacccaacucuugccaaacuugcc
```

folding energy of structure: **-33.6** kcal/mol

1 known mature miRNA(s) exhibit sequence similarity to this candidate hairpin

**id**	1991116
**genomic_position**	12941937-12942051 on CEL150_II (+) in Caenorhabditis elegans
***L* score**	4.86890627252e-05

structure

```
     ag  a-     gg    a    c  ca         caau    gaauucc
aaguu  cc  aacuu  gcaa aguu gg  agacuuugg    acuu       g
|||||  ||  |||||  |||| |||| ||  |||||||||    ||||       g
uucaa  gg  uugag  cguu ucaa cc  uuugaaacc    ugaa       c
     aa  gg     aa    c    c  cc         aauu    accacgu

mmmmmxxmmxBmmmmgxxmmmmxmmmmxmmxxmgmmmmmmmxxxxmmmm
```

sequence

```
(((((..((.(((((..((((.((((.((..(((((((((....((((.................))))....)))))))))..)).)
))).)))).))))))..))..)))))
aaguuagccaaacuugggcaaaaguucggcaagacuuuggcaauacuugaauuccggcugcaccaaaguuuaaccaaaguuuccccca
acucuugcaagaguugggaaaacuu
```

folding energy of structure: **-34.83** kcal/mol

1 known mature miRNA(s) exhibit sequence similarity to this candidate hairpin

**id**	2043412
**genomic_position**	13173537-13173633 on CEL150_II (+) in Caenorhabditis elegans
***L* score**	0.102327407922

structure

```
    c    c-     c    ga     a         caaaa    g
uugg uaag  uuggg gaaa  ugggu agacuuugg     cuug u
|||| ||||  ||||| ||||  ||||| |||||||||     ||||
agcc auuc  aaccc cuuu  accca uuugaaacu     ggac u
    -    uc     a    ga     a         acagc    u
```

mgmmbmmmmxBmmmmmxmmmmxxmmmmmxmgmmmmmmgxxxxxmgmm

sequence

((((.((((.(((((.((((..(((((.(((((((((.....((((....)))).....))))))))).))))).)))).)))))..))))))))
uuggcuaagcuugggcgaaagaug*gguaagacuuuggcaaaacu*ugguuucaggcgacaucaaaguuuaacccaaguuucacccaacucuuaccga

folding energy of structure: **-35.3** kcal/mol
1 known mature miRNA(s) exhibit sequence similarity to this candidate hairpin

**id**	2064086
**genomic_position**	13502011-13502095 on CEL150_II (+) in Caenorhabditis elegans
***L* score**	0.000178408489523

structure

```
      uug-----    a      aaa          aagaaa
aguuga        agga cccgug   ugucggcugc      a
||||||        |||| ||||||   ||||||||||
ucggcu        uccu gggcac   acagccgacg      u
      uuagaaaa    -      guc          aaauau
```

mmggmmxxxBBBBBmmmmbmmmmmmxxxmmmmmmmmmm

sequence

((((((...((((.((((((...((((((((((..............))))))))))...))))))))))........))))))
aguugauugaggaa*cccgugaaaugucggcugca*agaaaauuauaaagcagccgacacugcacggguccuaaaagauuucggcu

folding energy of structure: **-39.64** kcal/mol
1 known mature miRNA(s) exhibit sequence similarity to this candidate hairpin

id: 2047661

genomic_position: 13515554-13515672 on CEL150_II (+) in Caenorhabditis elegans

***L* score**: 0.00717506840493

structure

```
  ugg          accaac   g       c  u  ca        a  c    gac
gu   cuaaacuugg      guu ggcaaga uu gg  aaacuugg uu gggc   a
||   ||||||||||      ||| ||||||| || ||  |||||||| || ||||   u
ca   gauuuggacc      caa ccguucu aa cc  uuugaacc aa uuug   c
  a--          cagcuu   a       c  c  ac        c  -    aaa

mmxbbmmmmmmgmmmxxxxxxmmmxmmmmmmmxmmxmmxxmmmmmmmmxmmbgggm
```

sequence

```
((...((((((((((......(((.(((((((.((.((..((((((((.((.((((........)))))).))))))))..))
.)).))))))).)))......)))))))))).))
```

guuggcuaaacuuggaccaacguug*ggcaagacuuuggcaaaacu*uggauucgggcgacaucaaaguuuaacccaaguuucacc
caacucuugccaaacuucgacccagguuuagaac

folding energy of structure: **-39.0** kcal/mol

2 known mature miRNA(s) exhibit sequence similarity to this candidate hairpin

id: 1976550

genomic_position: 15256702-15256813 on CEL150_II (+) in Caenorhabditis elegans

***L* score**: 9.45151064191e-05

structure

```
    c-       auauu---                      uagaa   a  gc
cuaa  aacuagg        agcagccgacacguuacgggcu     uuu cu  u
||||  |||||||        ||||||||||||||||||||||     ||| ||  a
gauu  uuggucu        ucgucggcuguguaaugcccga     aaa ga  u
    uu       agggccau                      uagua   a  ag

mmmmxBmmmgmmgxxxxxBBBmmmmmmmmmmmmgmmmmmmmmmxxxxxmmmxmm
```

sequence

```
((((.(((((((.....((((((((((((((((((((((.....(((.((.......)).))).....))))))))))))))))))))))
).......))))))).))))
```

cuaacaacuaggauauuagcagccgacacguuacgggcuuagaauuuacugcuaugaagaaaaauga*uagcccguaaugugucggcug*c
uuaccgggaucugguuuuuuag

folding energy of structure: **-49.92** kcal/mol

2 known mature miRNA(s) exhibit sequence similarity to this candidate hairpin

**id**	4515471
**genomic_position**	1101497-1101616 on CEL150_III (+) in Caenorhabditis elegans
***L* score**	0.00242345988778

structure

```
    gua   uuuu  a       cg   cu  cu          g---    gc    acaa
uuuu   cca    gu ccgaguu  gcu  ag  ucuugcucaa    uuug  ccaa    u
||||   |||    || |||||||  |||  ||  ||||||||||    ||||  ||||    a
aaaa   ggu    ua gguucaa  cgg  uc  agaacggguu    agac  gguu    u
    ag-   u---  -       aa   uu  --          gaga    ua    caau
```

mmmmxxbmmmxbbbgmbmmgmmmmxxmmgxxmmbbmmmmmmgmmmxBBBmgmmxxmmmm

sequence

((((...(((....((.(((((((..(((..((..((((((((((.((((..((((...........))))..)))).....))
))))))))))..)))..)))))))))).)))..))))

uuuuguaccauuuuguaccgaguucggcucuagcuucuugcucaaguuuggcccaaacaauauuaacuuggaucagaagaguu
g*ggcaagacuuuggcaaaacu*uggauuugggaaaaa

folding energy of structure: **-36.9** kcal/mol

2 known mature miRNA(s) exhibit sequence similarity to this candidate hairpin

**id**	4546124
**genomic_position**	2916983-2917057 on CEL150_III (+) in Caenorhabditis elegans
***L* score**	0.0155916136128

structure

```
    ca   g      a         caaaa    a
uggg  aaa uugggc agacuuugg     cuug a
||||  ||| |||||| |||||||||     ||||
accc  uuu aacccg uuugaaacu     ggac u
    ac   g      a         acagc    u
```

mmmmxxmmmxmmmmmmxmgmmmmmmgxxxxxmgmm

sequence

((((..(((.((((((.(((((((((.....((((....)))).....))))))))).)))))).)))..))))

ugggcaaaaguug*ggcaagacuuuggcaaaacu*ugaauucaggcgacaucaaaguuuagcccaaguuucaccca

folding energy of structure: **-30.7** kcal/mol

2 known mature miRNA(s) exhibit sequence similarity to this candidate hairpin

**id**	2229988
**genomic_position**	3596985-3597083 on CEL150_III (+) in Caenorhabditis elegans
***L* score**	0.0114687741443

structure

```
      a      -   c   ac     ca       aa  c     gc
uugggc agaguu ggc aag  uuugg  aaacuug  uu aggcu  a
|||||| |||||| ||| |||  |||||  |||||||  || |||||  c
aaccug uuucaa ccg uuc  aaacc  uuugaac  aa uuuga  c
      g      a   -   ac     ac       cc  -     aa

mmmmgmxmgmmmmBmmmbmmmxxmmmmmxxmmmmmmmxxmmbmggmm
```

sequence

```
((((((.(((((((((.(((..(((((..(((((((..((.(((((.......)))))))..)))))))..))))).)..))))
)).)))))).))))))
uugggcaagaguuggccaagacuuuggcaaaacuugaauucaggcugcaccaaaguuuaacccaaguuucaccaaacacuug
ccaaacuuugguccaa
```

folding energy of structure: **-32.7** kcal/mol

1 known mature miRNA(s) exhibit sequence similarity to this candidate hairpin

**id**	2196306
**genomic_position**	3876768-3876860 on CEL150_III (+) in Caenorhabditis elegans
***L* score**	0.0225865595926

structure

```
           gaugg   uau      guu         agc       a
aauggcgccau     ucg   ugaugg   ugaugauga   ugaugau a
|||||||||||     |||   ||||||   |||||||||   |||||||
uuaccgcggug     agc   acuacu   acuacuacu   acuacua c
           a----   u--      ---         ---       u

mmmmmmmmmmmgxbbbbmmmxbbmmmmmgbbbmmmmmmmmmbbbmmmmmmm
```

sequence

```
(((((((((((.....(((...((((((...(((((((((...(((((((....))))))))))))))))))))).))).)))))))))))
aauggcgccaugauggucguauugaugguuugaugaugaagcugaugauaacuaucaucaucaucaucaucaucaucgaaguggcgccauu
```

folding energy of structure: **-45.1** kcal/mol

1 known mature miRNA(s) exhibit sequence similarity to this candidate hairpin

<table>
<tr><td>id</td><td>2414207</td></tr>
<tr><td>genomic_position</td><td>10524894-10524964 on CEL150_III (+) in Caenorhabditis elegans</td></tr>
<tr><td>L score</td><td>0.53734455228</td></tr>
<tr><td>structure</td><td>   c-   -   uuua   g
acu cau gauu   gcagccgacguuuuacg g
||| ||| |||| |||||||||||||||||
uga gua cuaa   cgucggcugugaagugc c
   uc   g   ccaa   c

mmmxBmmmBmmmmxxxxmmmmmmmmmggmmgmmm</td></tr>
<tr><td>sequence</td><td>(((.(((((((....((((((((((((((((((....)))))))))))))))))....)))).)))..)))
acuccaugauuuuuagcagccgacguuuuacgggcccgugaagugucggcugcaaccaaucgaugcuagu

folding energy of structure: -30.8 kcal/mol
1 known mature miRNA(s) exhibit sequence similarity to this candidate hairpin</td></tr>
</table>

<table>
<tr><td>id</td><td>2490824</td></tr>
<tr><td>genomic_position</td><td>245874-245960 on CEL150_IV (+) in Caenorhabditis elegans</td></tr>
<tr><td>L score</td><td>0.0511826610145</td></tr>
<tr><td>structure</td><td>   ug   g   a   gca acuuu   aa   g
ggc aacuu ggcaa aguugg   ag   ggcag cuug a
||| ||||| ||||| |||||| || ||||| ||||
ccg uugaa ccguu ucaacc   uc   ccguc ggac u
   gu   a   c   aaa c----   --   u

mmmxxmmmmmxmmmmmxmmmmmmxxxmmxbbbbmmmmmbbmgmm</td></tr>
<tr><td>sequence</td><td>(((..(((((.(((((.((((((...((.....(((((..((((....)))))))))).))...)))))).))))).)))))..)))
ggcugaacuugggcaaaaguugggcaagacuuuggcagaacuuggauucaggcugccccuaaaccaacucuugccaaaguuuggcc

folding energy of structure: -38.8 kcal/mol
1 known mature miRNA(s) exhibit sequence similarity to this candidate hairpin</td></tr>
</table>

**id**	2875398
**genomic_position**	12806505-12806596 on CEL150_IV (+) in Caenorhabditis elegans
***L* score**	0.0177509172603

structure

```
       c  u                 u      u     gca
uguuuuu gu guuuuagguacguuuua guugau ggcau   a
||||||| || ||||||||||||||||| |||||| |||||   a
gcgaaaa cg caaaauucauguaaaau caacua cugug   a
       u  u                 u      u     uua

gmgmmmmxmgxmmmmmmgmmmmgmmmmmxmmmmmmxmgmmg
```

sequence

```
(((((((.((.(((((((((((((((((.((((((.(((((........))))).)))))).))))))))))))))))).)).)))))))
```

uguuuuucguuguuu*uagguacguuuuauguugauugg*caugcaaaaauugugucuaucaacuuaaaauguacuuaaaacugcuaaaagcg

folding energy of structure: **-37.5** kcal/mol

1 known mature miRNA(s) exhibit sequence similarity to this candidate hairpin

**id**	2981916
**genomic_position**	15168040-15168155 on CEL150_IV (+) in Caenorhabditis elegans
***L* score**	0.00561533942048

structure

```
     uua   ga     g   -  ---     ga-       ac a     --    ag
aggag   uuc  gauag aug cc   uagca   ccugccu  c ccuau  uugu  g
|||||   |||  ||||| ||| ||   |||||   |||||||  | |||||  ||||
uuuuu   aag  cuauu uac gg   aucgu   ggacgga  g ggaug  aaca  u
     gcg   aa     a   a  ucg     aac       gc c     ca    ca

mggmgxxxmmmxxmmmmgxmmmBmmBBBmmmmmxxBmmmmmmmxxmxmmmmgBBmmmm
```

sequence

```
(((((...(((..(((((.((((((((((..(((((((..(.(((((((((......))))..))))).)..)))))))...)))))...)).
))).))))))..)))...)))))
```

aggaguuauucgagauaggaugccuagcagaccugccuaccaccuauuuguagguacacaaacguaggcgcg*aggcaggcaaugcuagcugg*a
cauauuaucaagaagcguuuuu

folding energy of structure: **-36.0** kcal/mol

1 known mature miRNA(s) exhibit sequence similarity to this candidate hairpin

<table>
<tr><td>id</td><td>3083314</td></tr>
<tr><td>genomic_position</td><td>600114-600211 on CEL150_V (-) in Caenorhabditis elegans</td></tr>
<tr><td>L score</td><td>0.00822253846695</td></tr>
<tr><td>structure</td><td>   a   a   a u   ca-   c   ca   cu
uugggc aagguua gcaag c uugg   aaa uuggauu ggcu a
|||||| ||||||| ||||| | |||| ||| ||||||| |||| c
aacucg uuucaau cguuc g aacc   uuu aacuuaa uuga a
      g   c   - c   acc   a   --   cu

mmmgmmxmmgmmmmxmmmmmbmxmmmmxxBmmmxmmmgmmmbbggmm</td></tr>
<tr><td>sequence</td><td>((((((.(((((((.(((((.(.((((..(((.(((((((..((((.......)))))))))))).)))...)))).)))))).))))))).))))))
uugggcaaagguuaagcaagacuuuggcaaaacuuggauucaggcuacaucaaaguuaauucaaauuuccaccaacgcuugccuaacuuuggcucaa

folding energy of structure: -29.5 kcal/mol
2 known mature miRNA(s) exhibit sequence similarity to this candidate hairpin</td></tr>
</table>

<table>
<tr><td>id</td><td>3130412</td></tr>
<tr><td>genomic_position</td><td>1304647-1304762 on CEL150_V (+) in Caenorhabditis elegans</td></tr>
<tr><td>L score</td><td>0.00653196564705</td></tr>
<tr><td>structure</td><td>   au   a-   a c   ca   u   a   uug   u a
gacuugg cagaa uuggcaa a uuggg agacuu ggc aaau   ggu c g
||||||| ||||| ||||||| | ||||| |||||| ||| |||| ||| | a
uugagcc gucuu aaccguu u aaucc uuugaa ccg uuug   cca g c
       c-   ca   c c   ac   c   a   uua   c u

gmmmgmmxbmmmmmxBmmmmmmmxmxmmgmmxxmgmmmmxmmmxmmmgxxxmmmxm</td></tr>
<tr><td>sequence</td><td>(((((((..(((((.(((((((.(.(((((..((((((.(((.((((...(((.(.....).)))...)))).)))
.))))))..))))).).)))))))..))))).)))))))
gacuuggaucagaaauuggcaaaacuugggcaagacuuuggcaaaauuuggguucagacugcaccauuguuuagcc
caaguuucaccuaacucuugccaaacuucugcccgaguu

folding energy of structure: -42.4 kcal/mol
1 known mature miRNA(s) exhibit sequence similarity to this candidate hairpin</td></tr>
</table>

**id**	3148670
**genomic_position**	2851037-2851136 on CEL150_V (+) in Caenorhabditis elegans
***L* score**	0.0966069330178
**structure**	a g cu c- auuc gc<br>uuggaucaga guu ggcaaga uugg aaaacuugg aggcu a |||||||||| ||| ||||||| |||| ||||||||| ||||| c<br>aaccuaguuu caa ccguucu aacc uuuugaacc uuuga c<br>- a c- aa cau- aa<br><br>mmmmmmmmmgmbmmmxmmmmmmmxbmmmmxBmmmmmmmmmxxxbmggmm
**sequence**	((((((((((.(((.(((((((..((((.(((((((((....(((((.......)))))...)))))))))..)))).))<br>))))).)))))))))))))<br>uuggaucagaaguug*ggcaagacuuuggcaaaacu*uggauucaggcugcaccaaaguuuuacccaaguuuuaaccaacuc<br>uugccaaacuuugauccaa<br><br>folding energy of structure: **-40.9** kcal/mol<br>2 known mature miRNA(s) exhibit sequence similarity to this candidate hairpin

**id**	3179049
**genomic_position**	2851150-2851221 on CEL150_V (-) in Caenorhabditis elegans
***L* score**	0.0349718376886
**structure**	a- g ca c- aaa a<br>ggc aaa uuggg aga uuuggcg cuug u ||| ||| ||||| ||| ||||||| |||| cug uuu aaccc ucu aaaccgc ggac u<br>ag g a- ua gcc c<br><br>mgmxBmmmxmmmmmxbmmmxBmmmmmmmxxxmgmm
**sequence**	(((.(((.(((((..(((.(((((((...((((....))))...)))))))..))).))))).)))..)))<br>ggcaaaaguug*ggcaagacuuuggcgaaacu*uggauucaggccgcgccaaaauucuacccaaguuugaguc<br><br>folding energy of structure: **-24.4** kcal/mol<br>1 known mature miRNA(s) exhibit sequence similarity to this candidate hairpin

**id**	3240609
**genomic_position**	4703558-4703654 on CEL150_V (-) in Caenorhabditis elegans
***L* score**	5.74977015012e-05

structure

```
     a     cg       c  u  ca       aauucau   ug
ugggc aaagu  ggcaaga uu gg  aaacuug       gcu  c
||||| |||||  ||||||| || ||  |||||||       |||  a
acccg uuuca  ccguucu aa cc  uuuggac       uga  c
     g     aa       c  c  ac       ccaauu-   ac

mmmmmxmmmmmxxmmmmmmmxmmxmmxxmmmmgmmxxxxxxbgmm
```

sequence

```
(((((.(((((..(((((((.((.((..(((((((.......(((.......)))......)))))))..)).)).)))))))..))))).)))))
```

ugggcaaaaguc*gggcaagacuuuggcaaaacu*ugaauucaugcugcaccaaaguuuaacccagguuucacccaacucuugccaaacuuuggccca

folding energy of structure: **-35.92** kcal/mol

2 known mature miRNA(s) exhibit sequence similarity to this candidate hairpin

**id**	3227648
**genomic_position**	4844053-4844172 on CEL150_V (+) in Caenorhabditis elegans
***L* score**	1.70886568918e-05

structure

```
    auu         c---    -------   a   -            aaauaa      ag
cggc   uggaucuag    ugcu       ugc cca guuuugcccaaa      uaacuu  u
||||   |||||||||    ||||       ||| ||| ||||||||||||      ||||||  g
gucg   acuuagguu    acgg       acg ggu caaaacggguuu      guugaa  c
    g--         caaa    uuucaaa   -   u            aaauca      ga

mgmmxbbmmgmmmgmgxBBBmmmgBBBBBBmmmbmmmBmmmmmmmmmmmmxxxxxxgmmmmm
```

sequence

```
((((...(((((((((.(((((((.((((((((((((((......((((((.......))))))......)))))))))))
)).)))))).......)))).....)))))))))).))))
```

cggcauuuggaucuagcugcuugcaccaguuuugcccaaaaaauaauaacuuagugcagaaguugacuaaauuugggcaaa
acuug*ggcaaaacuuuggcaaaacu*uggauucaggcug

folding energy of structure: **-41.31** kcal/mol

1 known mature miRNA(s) exhibit sequence similarity to this candidate hairpin

**id**	3411064
**genomic_position**	11066746-11066854 on CEL150_V (-) in Caenorhabditis elegans
***L* score**	0.102327407922

structure

```
           a  g   a       c  u  c        ga  c    caa
uaaacuugggc aa guu ggcaaga uu gg gaaacuug  uu aggc   u
||||||||||| || ||| ||||||| || || ||||||||  || ||||   a
guuugaacccg uu uaa ucguucu aa cc cuuugaac  aa uuug   u
           g  g   a       c  c  a        uc  -    aac
```

gmmmmmmmmmmxmmxgmmxgmmmmmmxmmxmmxmmmmmmmmxxmmbmggm

sequence

((((((((((.((.(((.(((((((.((.((.((((((((..((.((((.........))))))..)))))))).)).)).))
))))).))).)).)))))))))))
uaaacuugggcaaagguu*aggcaagacuuuggcgaaacu*uggauucaggcaauaucaaaguuuaacucaaguuucacccaacuc
uugcuaaauguuggcccaaguuug

folding energy of structure: **-44.7** kcal/mol
2 known mature miRNA(s) exhibit sequence similarity to this candidate hairpin

**id**	3450047
**genomic_position**	12302100-12302170 on CEL150_V (-) in Caenorhabditis elegans
***L* score**	2.76249354171e-05

structure

```
   c    uuaau      a  aaaa  -       g
uga gaug     aggaga ga    ga gaagaug a
||| ||||     |||||| ||    || |||||||
acu cuac     uucucu cu    cu cuucuac u
   a    -----      a  acua  a       g
```

mmmxmmmmbbbbmgmmmmxmmxxxxmmBmmmmmmm

sequence

(((.((((.....((((((.((....(((((((((....)))))))).))....)).)))))))))).)))
ugacgauguuaauaggagaagaaaaagagaagauguua*ucaucuucaucaucaucauc*ucuucaucauca

folding energy of structure: **-19.6** kcal/mol
1 known mature miRNA(s) exhibit sequence similarity to this candidate hairpin

**id**	3561947
**genomic_position**	16348304-16348419 on CEL150_V (+) in Caenorhabditis elegans
***L* score**	0.0153853123148

structure

```
   c    a-     ua-  c   u      g     ca         caaaa    g
gau agaa  uuggc   ag uug guaaaa uuggg  agacuuugg     cuug a
||| ||||  |||||   || ||| |||||| |||||  |||||||||     ||||
cua ucuu  aacug   uc aac cauuuu aaccc  uuugaaacu     gggc u
   a    ca     ucc  -   c      g     ag         acaag    u

mmmxmmmmxBmmmgmxxBmmbmmmxmmmmmmxmmmmmxxmgmmmmmmgxxxxxmggm
```

sequence

(((.((((.(((((..((.(((.((((((.(((((..(((((((((.....((((....))))).....)))))))))..))))).)))))
).))))))...))))))..))))).)))
gaucagaaauuggcuaagcuuguguaaaaguug*ggcaagacuuuggcaaaacu*uggauucggggaacaucaaaguuugacccaaguuuua
cccaacuccugucaaacuucuaauc

folding energy of structure: **-35.7** kcal/mol
2 known mature miRNA(s) exhibit sequence similarity to this candidate hairpin

**id**	3714507
**genomic_position**	20590842-20590916 on CEL150_V (-) in Caenorhabditis elegans
***L* score**	0.00222552655899

structure

```
     u   a-  auc   a          cacg
aaagc cgc  uc   auc ucaucaucau    gagg
||||| |||  ||   ||| ||||||||||    ||| g
uuuug gug  ag   uag aguaguagua    cucc
     -   ac  guu   c          aua-

mmmgmbmgmxBmmxxxmmmxmmmmmmmmmmxxxbmmm
```

sequence

(((((.(((.((...(((.((((((((((....(((...)))...)))))))))).)))...))..))))))))
aaagcucgca*ucaucaucaucaucaucauca*cggaggcucucauaaugaugaugacgauuuggacagugguuuu

folding energy of structure: **-21.4** kcal/mol
1 known mature miRNA(s) exhibit sequence similarity to this candidate hairpin

**id**	3729314
**genomic_position**	539531-539646 on CEL150_X (-) in Caenorhabditis elegans
***L* score**	0.000440348779386

structure

```
       ag     uaaacu    caa       - c          caaaa   ga
gaucaga  uugac      uggg   aaauuug g aggacuuugg     cuu  u
|||||||  |||||      ||||   ||||||| | ||||||||||     |||
cugguuu  aacug      accc   uuugaac c uuuugaaacc     gga  u
       cu     uugucu    cac       u a          acguc   ac

mmgmmgmxxmmmmmxxxxxxmmmmxxxmmmgmmmBmxmggmmmmmmmxxxxxmgm
```

sequence

```
(((((((..(((((......((((...((((((((.((((((((((.....(((......))).....)))))))))).).))))
)))...))))......)))))..)))))))
```

gaucagaaguugacuaaacuugggcaaaaauuu*ggcaggacuuuggcaaaacu*uugauucaggcugcaccaaaguuuuacucaag
uuucaccccaucuguugucaaucuuugguc

folding energy of structure: **-34.7** kcal/mol

1 known mature miRNA(s) exhibit sequence similarity to this candidate hairpin

**id**	3731460
**genomic_position**	624954-625048 on CEL150_X (-) in Caenorhabditis elegans
***L* score**	0.000109205356242

structure

```
   -    gc     u  c     c  g-         aaaa    u
gca uuug  ucuaa uu uugcc aa  uuugcccaa    ugua a
||| ||||  ||||| || ||||| ||  |||||||||    |||| a
cgu ggac  agguu aa aacgg uu  gaacggguu    acau c
   c    uu     c  -     u  ga         gaaa    u

mmmBggmmxxmmgmmxmmbmmmmmxmmxBgmmmmmmmmxxxxmmmm
```

sequence

```
(((((((..(((((.((.(((((.((.(((((((((....((((.....))))....)))))))))..)).))))))).)))))..)))).)))
```

gcauuuggcucuaauuucuugcccaaguuugcccaaaaaauguauuaacuacaaaaguug*ggcaagaguuuggcaaaacu*uggauucaggcugc

folding energy of structure: **-30.4** kcal/mol

1 known mature miRNA(s) exhibit sequence similarity to this candidate hairpin

<table>
<tr><td>id</td><td>3846390</td></tr>
<tr><td>genomic_position</td><td>3912836-3912933 on CEL150_X (-) in Caenorhabditis elegans</td></tr>
<tr><td>L score</td><td>0.00931295735244</td></tr>
<tr><td>structure</td><td>   a   ag   c   cu   c-   a uaug cu
uugg caaa uuggg aaga uugg caaacuugg uu   cu g
|||| |||| ||||| |||| |||| ||||||||| || || c
ggcc guuu aaccc uucu aacc guuugaacc aa   ga g
    g   ca   -   c-   ua   c uua- cc

ggmmxmmmmxxmmmmmbmmmmxbmmmmxBmmmmmmmmmxmmxxxbmm</td></tr>
<tr><td>sequence</td><td>((((.((((..(((((.((((..((((.(((((((((.((....((.......))...)).)))))))))..)))).)))))))))..)))).))))
uuggacaaaaguugggcaagacuuuggccaaacuuggauuuaugcugcgccaaagauuaacccaaguuugauccaacucuucccaaacuuuggccgg

folding energy of structure: -31.6 kcal/mol
1 known mature miRNA(s) exhibit sequence similarity to this candidate hairpin</td></tr>
</table>

<table>
<tr><td>id</td><td>4048106</td></tr>
<tr><td>genomic_position</td><td>11051157-11051250 on CEL150_X (+) in Caenorhabditis elegans</td></tr>
<tr><td>L score</td><td>0.000347862126048</td></tr>
<tr><td>structure</td><td>   a   a   aacu   ca-   au   ag auu
ggccaaa guu ggcaa   uugg   aaacuugg uca c   a
||||||| ||| ||||| |||| |||||||| ||| | c
ccgguuu caa ccguu   aacc   uuugaacc agu g   c
       -   a   cuc-   cac   c-   cu aaa

mmmmmmmbmmmxmmmmmxxxbmmmmxxBmmmmmmmmxbmmmxxm</td></tr>
<tr><td>sequence</td><td>(((((((.(((.(((((....((((..((((((((..(((..(.........)..))).))))))))...))))...))))).))))))))))
ggccaaaaguuaggcaaaacuuuggcaaaacuuggauucaagcauuaccaaagucugacccaaguuucacccaacucuugccaaacuuuggcc

folding energy of structure: -33.3 kcal/mol
2 known mature miRNA(s) exhibit sequence similarity to this candidate hairpin</td></tr>
</table>

**id**	4147031
**genomic_position**	14917324-14917447 on CEL150_X (+) in Caenorhabditis elegans
***L* score**	7.22734328364e-05

structure

```
   uuc  g  a       cu  cu          -     g u    aaa  -       gg
gcc   uc ga uuuugcu  ag  ucuugcccaa guuug c caaa   ua uuaacuu  a
|||   || || |||||||  ||  |||||||||| ||||| | ||||   || |||||||  u
cgg   ag uu aaaacgg  uc  agaacggguu caaac g guuu   au gguugaa  c
   cau  g  c       uu  --          g     - -    caa  c       ga
```

mmmxxxmmxgmxmmmmmmgxxmmbbmmmmmmmmmmBmmmmmbmbmmmmxxxmmBggmmmmm

sequence

(((...((.((.(((((((..((..((((((((((((((.(.((((...((((((((.......))))))).))...)))))
))))).)))))))))))))..))))))).)).))...)))

gccuucucggaauuuugcucuagcuucuugcccaaguuuggcucaaaaaauauuaacuuggaucagaaguuggcuaaacuuugg
caaacguug*ggcaagacuuuggcaaaacu*uggauacggc

folding energy of structure: **-40.3** kcal/mol

2 known mature miRNA(s) exhibit sequence similarity to this candidate hairpin

**id**	4167805
**genomic_position**	14917515-14917627 on CEL150_X (-) in Caenorhabditis elegans
***L* score**	0.0351579811802

structure

```
 c          caa    ug       cu    cac-       a  c    caa
g uaaacuuggg   aagu  ggcaaga  uugg    aacuugg uu gggc   c
| ||||||||||   ||||  |||||||  ||||    ||||||| || ||||   a
c guuugaaccc   uuca  ccguucu  aacc    uugaacc aa uuug   u
 a          auc    ua       c-    uacu       c  -    aac
```

mxgmmmmmmmmmxxxmmmmxxmmmmmmmxbmmmmxxxBmmmmmmmxmmbgggm

sequence

(.((((((((((...((((..(((((((..((((...(((((((.((.((((.........)))))).))))))).....)))).))
)))))..))))...)))))))))).)

gcuaaacuugggcaaaaguug*ggcaagacuuuggcacaacu*uggauucgggcaacaucaaaguuuaacccaaguuucauccaacuc
uugccauacuucuacccaaguuugac

folding energy of structure: **-38.1** kcal/mol

1 known mature miRNA(s) exhibit sequence similarity to this candidate hairpin

<table>
<tr><td>id</td><td>4181655</td></tr>
<tr><td>genomic_position</td><td>15447706-15447814 on CEL150_X (-) in Caenorhabditis elegans</td></tr>
<tr><td>L score</td><td>0.00266635069795</td></tr>
<tr><td>structure</td><td>   ag   a u   caa   ca-   aaa-   gg
caga uuggcaa a uuggg   aaauuggg   agacuuuggc   acu a
|||| ||||||| | ||||| |||||||| |||||||||| ||| u
guuu aaccguu u aaccc   uuugaccc   uuugaaacug   uga u
    ca   c c   ac-   cag   cagc   ac

mmgmxxmmmmmmmxmxmmmmmxxbmmmgmmmmxxBmgmmmmmmgmxxxBmmm</td></tr>
<tr><td>sequence</td><td>((((..(((((((.(.(((((...((((((((..((((((((((...(((.......)))....))))))))))...))))
))))..))))).).))))))))..))))
cagaaguuggcaaaauuugggcaaaaauugggcaagacuuuggcaaaacuuggauucagucgacgucaaaguuugacccca
guuucacccaacucuugccaaacuuug

folding energy of structure: -38.3 kcal/mol
2 known mature miRNA(s) exhibit sequence similarity to this candidate hairpin</td></tr>
</table>
