# Supplementary material for: In silico miRNA prediction in metazoan genomes: balancing between sensitivity and specificity
Source: BMC Genomics. 2009 Apr 30;10:204. doi: 10.1186/1471-2164-10-204 (PMC2688010; doi:10.1186/1471-2164-10-204)
Supplement: Additional file 9 — Performance of the scoring model Metazoa. Analysis of the performance of the scoring model Metazoa on sets of genomic hairpins other than those derived for the C. elegans genome. [file 1471-2164-10-204-S9.pdf]

Additional File 9: Performance of the scoring model *Metazoa* in other metazoan organisms

The default scoring model Metazoa is based on all known metazoan miRNAs. Parameterization was optimized for miRNA hairpin classification in *C. elegans*. This was done by selection of non-correlated descriptors that give optimal separation between known miRNAs and genomic hairpins from *C. elegans* and by assigning a weight proportional equal to the square root of a descriptor's discriminative power as measured at a sensitivity of 95%.

Here, we show examples of the performance of this scoring model in other Metazoan organisms, represented by the relatively miRNA-rich chromosomes X from *D. melanogaster* (drosoX) and 22 from human (hsa22) and provide a comparison with the performance on the *C. elegans* genome and separately on the *C. elegans* X chromosome. Prediction of genomic hairpins in drosoX and hasa22, which were used as negative sets in performance assessment, descriptor scoring and *L*-score calculation were performed as described in the paper.

The number of detected hairpins per Mb of (double stranded) sequence ranges from 2.21E4 and 3.56E4 (Table 1), and is dependent on the complexity and repetitiveness of the sequence.

Table 1. Retrieval of genomic hairpins

	Size (Mb)	N's (Mb)	Remaining (Mb)	Detected hairpins	Hairpins per ds Mb
4 viruses [1]	0.72	0.00	0.72	25599	3.56E4
C. elegans [1]	100.29	1.4e-03	100.29	3,525,115	3.51E4
celX [1]	17.72	7.8e-04	17.72	583,302	3.29E4
hsa22	49.69	14.84	34.85	1,096,254	2.21E4
drosoX	22.42	0.09	22.33	660,821	2.96E4

[1] data as in the paper

For assessment of performance, the count of the number of high scoring hairpins (*L* = 1.0) and the ROC area of miRNAs versus genomic hairpins are most relevant (Table 2). The human chromosome contains nearly 15 Mb of N nucleotides, the vast majority of which is placed in the otherwise highly repetitive telomeric and centromeric regions of the chromosome. This resulted in an underrepresentation of poorly scoring, overlapping hairpins in the hsa22 dataset and accounts for the much higher frequency of hairpin loci with L=1.

Except for the viral genomes, between 20 and 50 *L*= 1 hairpins per Mb of double stranded sequence were found for each dataset. The prediction that viral genomes contain many more hairpin loci with *L* = 1.0 per Mb reflects the fact that viruses are more miRNA-dense. For *D. melanogaster* chromosome X we searched the predicted hairpins for the occurrence of 27 known miRNAs (miRBase 12.0); 26 of these were present in our predictions (detection sensitivity of 96%), eight of which had an *L*-score of 1.0 (data not shown).

The Area Under the Curve (AUC) of the ROC plot can be considered as a measure of how well miRNA and genomic hairpin sets are separated from each other in the prediction. The lower AUC values for the drosoX and has22 hairpin sets thus reflect a poorer separation between miRNAs and genomic hairpins (relatively more genomic hairpin get a high *L* score compared with *C. elegans*). Although the difference in AUC

of nearly 2.5% between *C. elegans* and hsa22 seems small, expressed in number of remaining candidate miRNAs at a certain threshold for *L* this represents a considerable drop in performance.

Table 2. Number of L=1 hairpins / MB and AUC performance

	Detected hairpins	Hairpins with L=1	Hairpin loci with L=1	Hairpin loci [2] with L=1 per Mb	AUC area [3]
4 viruses [1]	25599	96	85	118	0.9601
C. elegans [1]	3525115	4104	3099	31	0.9872
celX [1]	583302	408	321	18	0.9896
hsa22	1096254	1839	1642	47	0.9633
drosoX	660821	593	540	24	0.9799

[1] data as in the paper
[2] definition of a unique hairpin locus is described in the paper
[3] AUC (Area Under the Curve) of a ROC-plot (Receiver Operating Characteristic) obtained with the scoring model Metazoa as described in the paper

In Table 3, we show the square root of the discriminative power of descriptors calculated for 250,000 random hairpins from the hairpin sets of hsa22 and drosoX in comparison with those obtained from *C. elegans* (which are used in the default scoring model). Indeed, the descriptor's discriminative powers vary, although the changes for most descriptors are small. For a few descriptors large differences are observed, most importantly for the descriptor with highest selectivity in the *C. elegans* hairpin set (MFEahl). The fact that this descriptor has a lower discriminative power in the hsa22 and drosoX hairpin sets explains the drop in AUC performance for the hsa22 and drosoX datasets.

The scoring model Metazoa has a parameterization (weighting and descriptor selection) that was optimized for performance on the *C. elegans* dataset of genomic hairpins. The *C. elegans* dataset serves as an example to demonstrate the usability and possibilities of the scoring model for miRNA classification and prediction in Metazoan genomes. The data presented above shows that this scoring model can be used directly for prediction in other genomes. However, when optimal performance for another taxonomic group or organism is desired, a dedicated, fine-tuned scoring model for that particular taxon can be expected to perform better than the model developed for miRNA classification in *C. elegans*. A random set of several hundreds of thousands genomic hairpins is required to perform this fine-tuning.

Table 3. Discriminative power of descriptors measured in three different sets of genomic hairpins

descriptor	SQRT(dp) Cel [1]	SQRT(dp) hsa22 [1]	SQRT(dp) drosX [1]	hsa22 difference (%) [2]	drosoX difference (%) [2]
**MFEahl**	3.651	1.444	2.146	**-60.5**	**-41.2**
**MFEahlindex**	2.179	2.097	2.589	**-3.7**	**18.8**
**Q**	1.735	1.516	1.660	**-12.6**	**-4.3**
**maxmatchcount**	1.658	1.756	1.762	**5.9**	**6.3**
**dP**	1.510	1.431	1.517	**-5.2**	**0.5**
**SCS-di**	1.330	1.047	1.111	**-21.3**	**-16.5**
**largestbulge**	1.319	1.188	1.208	**-9.9**	**-8.4**
**polyNucHairpin**	1.281	1.041	1.076	**-18.7**	**-16.0**
**SCS-mono**	1.265	1.021	1.050	**-19.3**	**-17.0**
**polyA**	1.257	1.028	1.081	**-18.2**	**-14.0**
**polyU**	1.237	1.005	1.032	**-18.7**	**-16.6**
**bulgeRatio**	1.204	1.276	1.231	**6.0**	**2.3**
**stemlength**	1.136	1.119	1.139	**-1.5**	**0.2**
**GU-matchcontribution**	1.131	1.121	1.174	**-0.9**	**3.8**
**longestmatch-stretch**	1.095	1.084	1.076	**-1.0**	**-1.7**
**looplength**	1.082	1.066	1.074	**-1.5**	**-0.7**
**GsurplusC**	1.072	1.081	1.058	**0.8**	**-1.4**
**GAsurplusCU**	1.058	1.090	1.055	**3.0**	**-0.3**

[1] SQRT(dp): square root of the discriminative power of a descriptor on the indicated set of hairpins. As positive set, in each case the set of all Metazoan miRNAs (3902) from miRBase 12.0 were used.

[2] percent difference relative to discriminative power on the complete *C. elegans* genomic hairpin set
