# Supplementary material for: In silico miRNA prediction in metazoan genomes: balancing between sensitivity and specificity
Source: BMC Genomics. 2009 Apr 30;10:204. doi: 10.1186/1471-2164-10-204 (PMC2688010; doi:10.1186/1471-2164-10-204)
Supplement: Additional file 10 — MiRNAs that excluded from the analyses. The hairpin structure of six sequences deviated considerably from the predicted characteristics of miRNA hairpins and were excluded from all subsequent analyses. [file 1471-2164-10-204-S10.pdf]

Additional File 10: miRNAs excluded from the analyses (6).

Accession	Fref	Organism	Rnafold energy
MI0001606	aga-mir-133	A. gambiae	-18.3
MI0005056	bta-mir-7	B. taurus	-20.7
MI0005299	mdo-mir-137	M. domestica	-11.6
None	PT_50	P. troglodytes	-41.0
None	PT_340	P. troglodytes	-24.44
None	PT_208	P. troglodytes	-34.54

**fref**	aga-mir-133
**accession**	MI0001606
**organism**	Anopheles gambiae
**rnafold_string**	((((((.(((((.........((((...((((.(((((........))))).))))))))..)))))..))))))..
**rnafold_energy**	-18.3
**source**	miRNA registry
**remark**	The reported mature miRNA from aga-mir-133 (MI0001606) is fully positioned in the loop of the hairpin precursor. Moreover, it fully spans the loop. According to current knowledge about interaction and substrate recognition by Dicer, this reported mature miRNA can impossible be cleaved out by Dicer from this precursor hairpin.

**fref**	bta-mir-7
**accession**	MI0005056
**organism**	Bos taurus
**rnafold_string**	((((((((.((....)).)))))).))............((((((.((((((.((......)).))))))))))))...
**rnafold_energy**	-20.7
**source**	miRNA registry
**remark**	MiRNA hairpin precursor bta-mir-7 (MI0005056) has an experimentally verified mature miRNA (MI0005056). Moreover, the mir-7 gene family has members in many metazoan species. However, the precursor sequence that was submitted to miRBase can not represent the true miRNA precursor sequence. Most likely a large part of the 3' end of the precursor sequence is missed in the experimental cloning effort. As a consequence, this precursor failed our hairpin structure concept and was abandoned in the analysis of known miRNA precursors.

**fref**	mdo-mir-137
**accession**	MI0005299
**organism**	Monodelphis domestica
**rnafold_string**	((((((..(.(((((((.....)).))))))..))))))....(.((....)).).
**rnafold_energy**	-11.6
**source**	miRNA registry
**remark**	MiRNA hairpin precursor mdo-mir-137 (MI0005299) is recognized based on similarity to the fully identical mmu-mir-137 (MI0000163). Moreover, the mir-137 gene family has members in several metazoan species. However, the precursor sequence that was submitted to mirBase can not represent the true miRNA precursor sequence. Most likely a large part of the 5' end of the precursor sequence is missed*. As a consequence, this precursor failed our hairpin structure concept and was abandoned in the analysis of known miRNA precursors.<br><br>* The central part of the precursor, directly around the loop, is recognizable and fully indentical to the precursor sequence and structure of mmu-mir-137.

**fref**	PT_50
**accession**	None
**organism**	Pan troglodytes
**rnafold_string**	((..((((((....((((((.((..(((.(((....(((..((..(((((.....)))))..))..)))......))).)))..))....))))))))))))..))
**rnafold_energy**	-41.0
**source**	Berezikov ea 2006
**remark**	miRNA precursor PT_50 (experimentally identified in a large-scale deep-sequencing project) is rejected by our structural model because it lacks at least one position with 15 matches in 23 positions*. Because all other properties and the experimental evidence of the hairpin point to a true miRNA precursor, this can be classified as a minor flaw of our structural hairpin concept.<br><br>* only 5 out of almost 5000 (0.1%) miRNA precursors are rejected by this constrain.

**fref**	PT_340
**accession**	None
**organism**	Pan troglodytes
**rnafold_string**	(((((........((((((...(((((((..((((..((((.............)))).....)))))))))....))....)))))))))))
**rnafold_energy**	-24.44
**source**	Berezikov ea 2006
**remark**	miRNA precursor PT_340 (experimentally identified in a large-scale deep-sequencing project) is rejected by our structural model because it lacks at least one position with 15 matches in 23 positions*. A second abbarent feature of this precursor, is that the mature miRNA sequences are not nicely mirrored on both sides of the stem with a characteristic 2-nt staggered cut, a signature of cleavage by the DCL family.<br><br>* only 5 out of almost 5000 (0.1%) miRNA precursors are rejected by this constrain.

**fref**	PT_208
**accession**	None
**organism**	Pan troglodytes
**rnafold_string**	(((((((((((((.......((((.(((((((((........)))))..))))))))).......))))))).....)))))))
**rnafold_energy**	-34.54
**source**	Berezikov ea 2006
**remark**	miRNA precursor PT_208 (experimentally identified in a large-scale deep-sequencing project) is rejected by our structural model because it lacks at least one position with 15 matches in 23 positions*. Because all other properties and the experimental evidence of the hairpin point to a true miRNA precursor, this can be classified as a minor flaw of our structural hairpin concept.

* only 5 out of almost 5000 (0.1%) miRNA precursors are rejected by this constrain.
